# Chemoselective Heterogeneous Hydrogenation of Sulfur Containing Quinolines under Mild Conditions

**DOI:** 10.1021/jacs.3c11163

**Published:** 2024-02-20

**Authors:** Lukas Lückemeier, Thijs De Vos, Lisa Schlichter, Christian Gutheil, Constantin G. Daniliuc, Frank Glorius

**Affiliations:** Universität Münster, Organisch-Chemisches Institut, Corrensstraße 36, Münster 48149, Germany

## Abstract

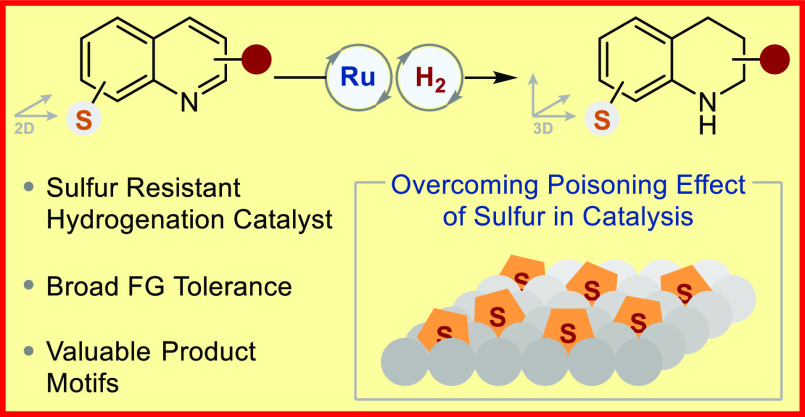

Sulfur, alongside oxygen and nitrogen, holds a prominent position as one of the key heteroatoms in nature and medicinal chemistry. Its significance stems from its ability to adopt different oxidation states, rendering it valuable as both a polarity handle and a hydrogen bond donor/acceptor. Nevertheless, the poisonous nature of its free electron pairs makes sulfur containing substrates inaccessible for many catalytic protocols. Strong and (at low temperatures) irreversible chemisorption to the catalyst’s surface is in particular detrimental for heterogeneous catalysts, possessing only few catalytically active sites. Herein, we present a novel heterogeneous Ru–S catalyst that tolerates multiple sulfur functionalities, including thioethers, thiophenes, sulfoxides, sulfones, sulfonamides, and sulfoximines, in the hydrogenation of quinolines. The utility of the products was further demonstrated by subsequent diversifications of the sulfur functionalities.

## Introduction

**Figure 1:**
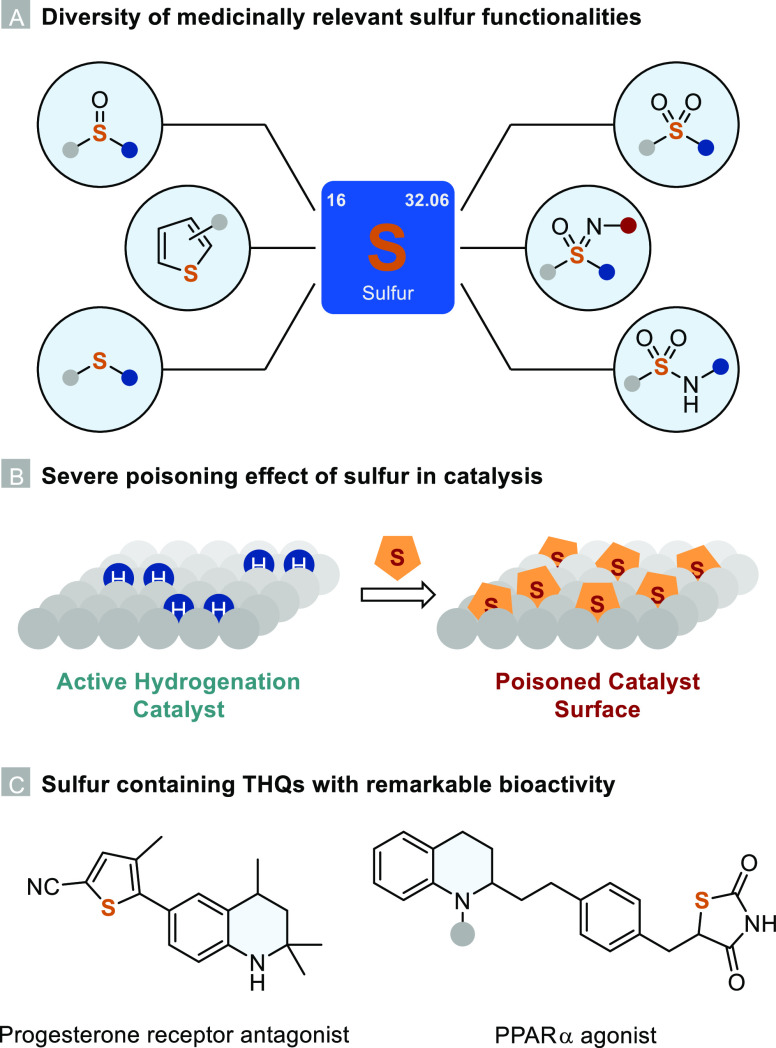
(A) Versatility of sulfur in organic chemistry. (B) Poisonous nature of sulfur in heterogeneous transition metal catalysis. (C) Examples of sulfur containing 1,2,3,4-tetrahydroquinolines with remarkable bioactive properties.

Sulfur is, next to oxygen and nitrogen, one of the most important heteroatoms in nature and ubiquitous in a plethora of natural products.^1−3^ Due to its many different oxidation states and functionalities, it is essential for medicinal chemistry or drug discovery, serving as a hydrogen bond donor/acceptor or as a polarity handle (Figure 1A).^4−6^ This is also represented by the fact that up until 2018, about 288 FDA approved drugs and 36 out of the top 100 marketed drugs in 2021 contained at least one sulfur atom.^7,8^ However, its free electron pairs in lower oxidation states render sulfur very Lewis basic and therefore a catalyst poison, in particular, for heterogeneous ones. It strongly chemisorbs to transition metal surfaces and blocks catalytically active sites (Figure 1B).^9,10^ Even concentrations of sulfur as low as 10 ppb can lead to substantial surface coverage and consequently to catalyst deactivation.^11^

**Scheme 1:**
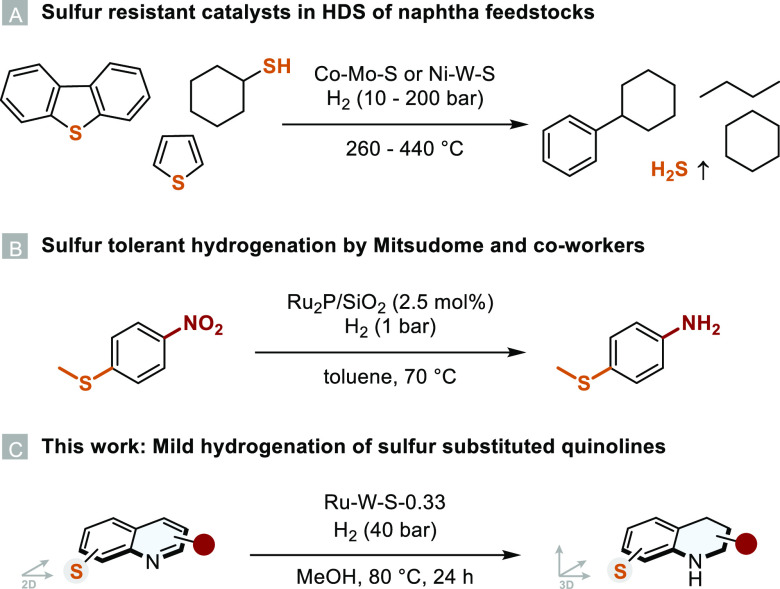
(A) Relevant Examples for the HDS of Naphtha Feedstocks; (B) Sulfur Tolerant Hydrogenation of Nitroarenes; (C) Sulfur Tolerant Hydrogenation of Quinolines

Heterogeneous, sulfur-resistant hydrogenation catalysts are scarce and usually employed in the hydrodesulfurization (HDS) of naphtha feedstocks for the petrochemical industry. At high temperatures and hydrogen pressures, sulfur impurities in the crude feedstock undergo hydrogenolysis to yield H_2_S and sulfur-free hydrocarbons,^12,13^ which are further refined in catalytic reforming processes (Scheme 1A).^14^ Supported cobalt–molybdenum-sulfide and nickel–tungsten-sulfide catalysts are the most common materials used for this process,^15^ but recently, research has been focused on the development of hydrogenation reactions catalyzed by these unsupported materials.^16,17^ Sulfur tolerant catalysts are usually metal sulfides or metal nanoparticles doped with heteroatoms to reduce the binding affinity of sulfur to the metal surface.^18−21^ A recent example of such a catalyst was published by Mitsudome and co-workers.^22^ By doping ruthenium nanoparticles with phosphorus, they were able to increase the catalyst’s sulfur tolerance in the hydrogenation of nitroarenes to anilines significantly (Scheme 1B). Employing only 1 bar of hydrogen pressure at 70 °C, they did not observe any HDS and showcased a broad functional group tolerance by the synthesis of many complex drug precursors.

Saturated N-heterocycles are crucial structural elements in a variety of natural products and pharmaceuticals.^23−25^ Tetrahydroquinolines (THQs) in particular frequently appear as key building blocks in ligands and compounds with intriguing bioactive properties (Figure 1C). Consequently, the synthesis of these motifs with various substitution patterns and functional groups is sought after and has been of interest in recent years.^26,27^ Arguably, the most straightforward way to synthesize THQs is the hydrogenation of the corresponding quinoline precursors. Quinolines are widely available and can be modified easily employing classical arene chemistry,^28,29^ while the construction and modification of THQs is still a challenging task. Therefore, hydrogenation can be used as a green and sustainable platform to bridge the gap between known 2D chemical space and complex 3D structures,^30,31^ enabling access to a broad variety of THQs. Inspired by the results of Mitsudome^22^ and the catalyst design of Corma,^16^ we set out to explore the hydrogenation of sulfur substituted quinolines to THQs using unsupported, binary metal sulfide catalysts (Scheme 1C). This catalyst type recently showed promising hydrogenation activity and bears the potential to tolerate poisonous sulfur functionalities.^16,17^ The central aspect to achieving this transformation is to fine-tune the reaction conditions to balance reactivity and minimize HDS.

## Results and Discussion

### Synthesis and Characterization of Catalysts

**Figure 2:**
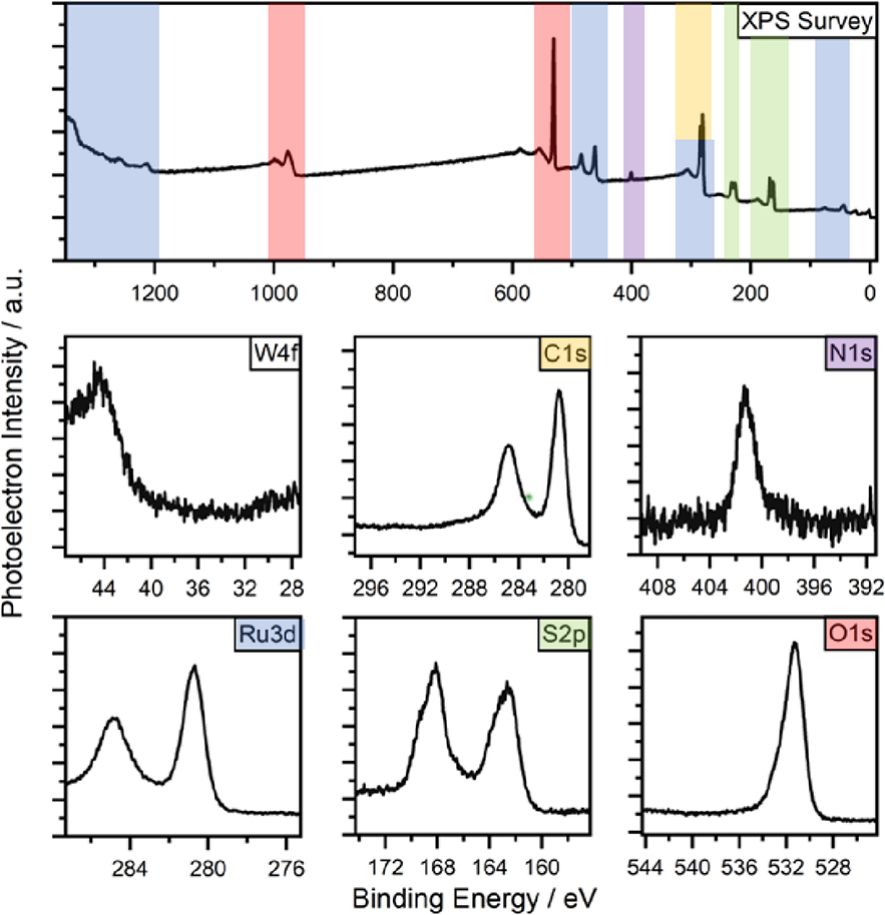
XPS measurement of Ru–W–S-0.33.

The metal sulfide catalysts utilized in this study were obtained via hydrothermal synthesis according to a procedure by Corma and co-workers.^16^ The corresponding metal precursor, either ammonium molybdate or sodium tungstate, and sulfur were reacted with an aqueous hydrazine solution in an autoclave at 180 °C. The resulting catalysts were denoted as [M]–Mo/W–S-X; X = [M]/([M] + Mo/W) molar ratio of the metal salts. The optimized ruthenium catalysts were analyzed by XPS measurements to gain insight into the surface structure and composition of the catalysts. To our surprise, the XPS analysis revealed that the final Ru–W–S-0.33 catalyst contained no tungsten (Figure 2): a W4f signal in the respective binding energy region between 31 and 36 eV could not be detected. S2p XP signals are usually detected at lower binding energies of ∼162 eV, whereas oxidized sulfur species, such as sulfites or sulfates, are detected at higher energies of ∼169 eV. It can be seen that Ru–W–S-0.33 consists of both sulfide and higher oxidized sulfite and sulfate species, even though the catalyst was synthesized under highly reducing conditions. Furthermore, a catalyst synthesized in a similar manner without the addition of any sodium tungstate (Ru–S) showed no signals of oxidized sulfur species in the respective S2p spectrum (see Figure S3). We therefore hypothesize that the tungstate acts more as an oxidant, partially oxidizing the sulfur and therefore not being reduced to WS_2_-layers, as observed in previous works.^16,17^ ICP-OES measurements of Ru–W–S-0.33 also confirm the absence of any significant amount of tungsten (see the Supporting Information for further details).

**Figure 3:**
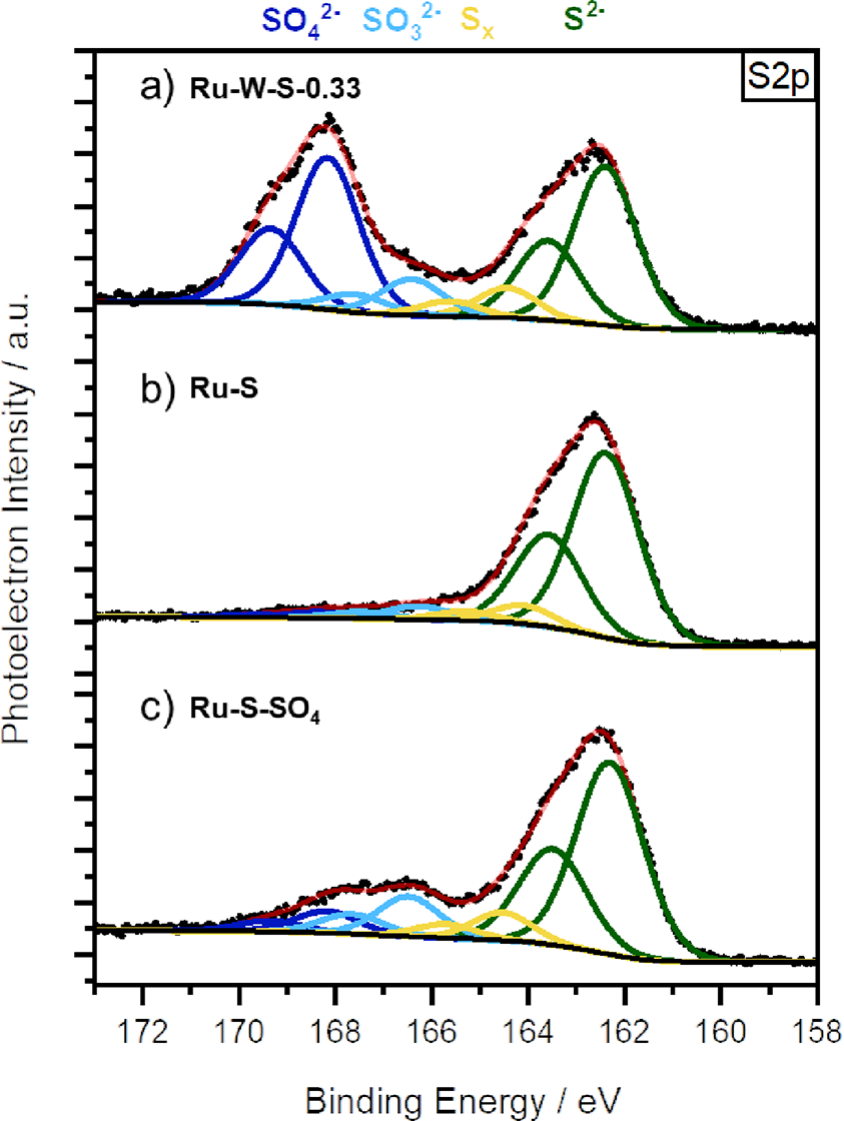
Comparison of the XP spectra of Ru–W–S-0.33, Ru–S, and Ru–S–SO_4_ focusing on the different sulfur species.

In order to further test our hypothesis of surface-bound sulfates, we synthesized an additional ruthenium catalyst. This time, we omitted sodium tungstate and added sodium sulfate instead, introducing the coordinating sulfur species directly into the catalyst synthesis. The resulting catalyst, Ru–S–SO_4_, was then also analyzed by XPS and the catalytic performance was compared to Ru–W–S-0.33 and Ru–S. Similar to Ru–W–S-0.33, Ru–S–SO_4_ also shows a mixture of oxidized and reduced sulfur species on the catalyst’s surface (Figure 3C, see Figure S4 for whole spectrum), therefore proving that sulfates can indeed coordinate to the catalyst’s surface and that they are produced during the synthesis of Ru–W–S-0.33. The amount of oxidized sulfur in the catalysts also differs significantly. While Ru–S does not contain any sulfates or sulfites, Ru–W–S-0.33 consists of visibly more oxidized sulfur than Ru–S–SO_4_ does (Figure 3). The hydrogenation activities of the different catalysts will be the subject of discussion later.

**Figure 4:**
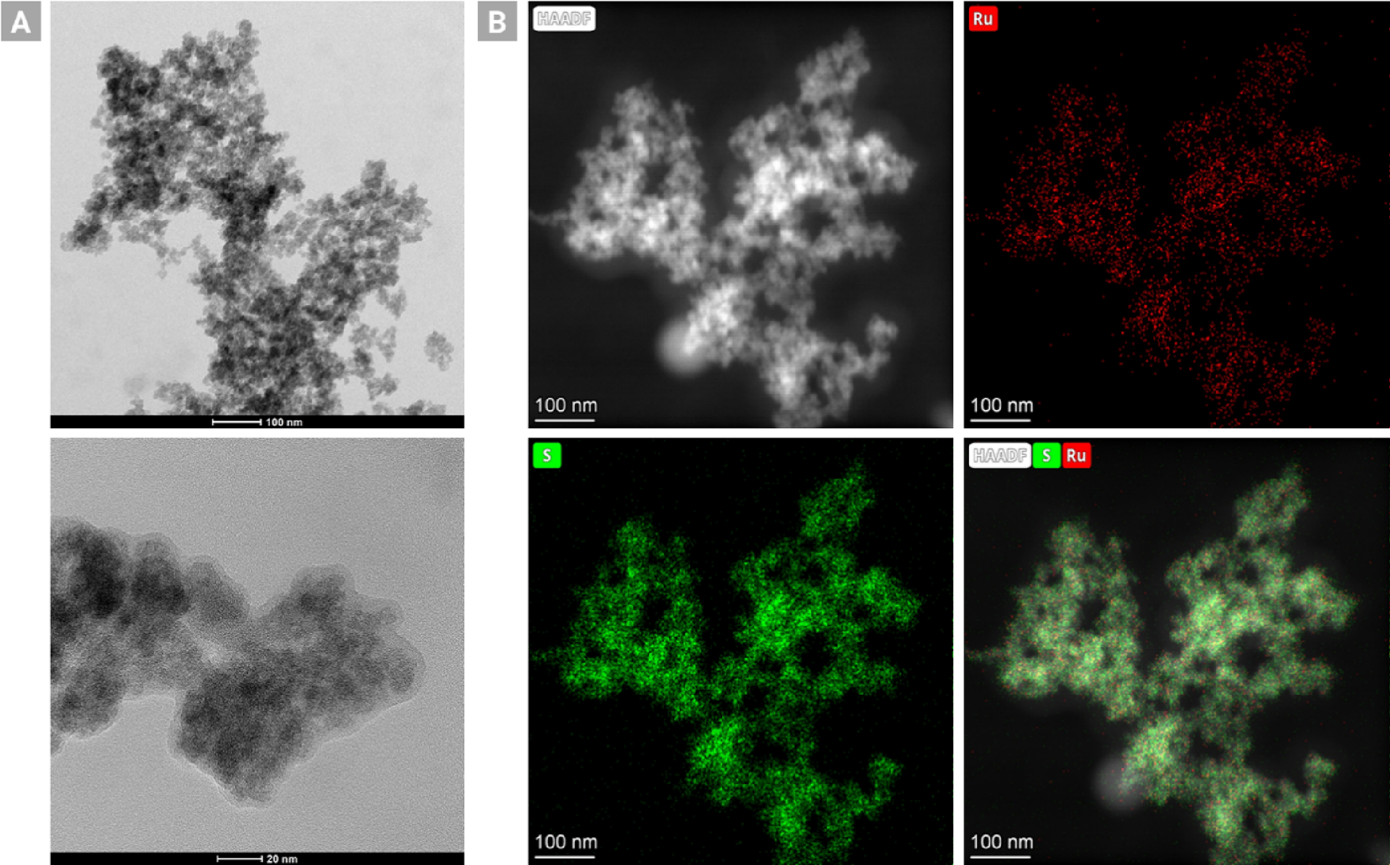
(A) TEM micrographs of Ru–W–S-0.33. (B) High-angle annular dark-field scanning transmission electron microscopy (HAADF-STEM) images and energy-dispersive X-ray (EDX) elemental mapping of Ru–W–S-0.33.

In order to analyze the catalyst’s nanostructure in more detail, DLS, TEM, STEM-HAADF, and STEM-EDX measurements were performed. While DLS measurements of Ru–W–S-0.33 confirm a nanoparticle structure of the catalyst in solution with an average particle size of ∼69 nm (including solvent shell, see Figure S4), TEM pictures show no evident formation of nanoparticles (Figure 4A). This might be caused by agglomeration of the unsupported nanoparticles under dry conditions. EDX elemental mapping again confirms the absence of tungsten and reveals that Ru–W–S-0.33 mostly constitutes of ruthenium and sulfur, which are evenly dispersed on the catalyst surface (Figure 4B). It also indicates that the sulfur content of the catalyst is significantly higher than the ruthenium content.

### Catalytic Results

To test the activity of the catalysts, we selected the hydrogenation of 2-methyl-8-(methylthio)quinoline as the benchmark reaction. Initial experiments were performed with the Ru–Mo–S-0.33 catalyst in toluene at 150 °C. To our delight, the catalyst gave full conversion, yet also a substantial amount of HDS was observed (Table 1, entry 1).

**Table 1:**
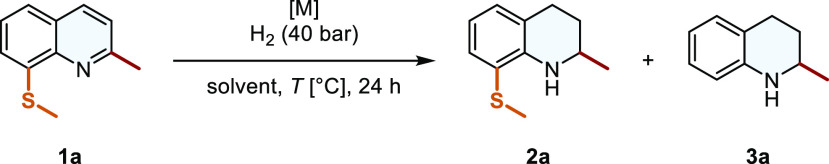
Investigation of Reaction Conditions^a^

**entry**	**catalyst**	**solvent**	*T***[°C]**	**yield 2a**	**yield 3a**
1	Ru–Mo–S-0.33	toluene	150	71%	29%
2	Ru–Mo–S-0.33	toluene	120	89%	4%
3	Rh–Mo–S-0.33	toluene	120	39%	1%
4	Co–Mo–S-0.33	toluene	120	79%	2%
5	Pd–Mo–S-0.33	toluene	120	44%	3%
6	Ru–Mo–S-0.33	EtOH	100	96%	3%
7	Ru–Mo–S-0.33	MeOH	80	96%	4%
8	Ru–Mo–S-0.33	MeOH	60	51%	0%
9	Ru–Mo–S-0.50	MeOH	60	90%	3%
10	Ru–W–S-0.33	MeOH	60	96%	2%
11	Ru–S	MeOH	60	32%	0%
12	Ru–S–SO_4_	MeOH	60	76%	0%

a Reaction conditions: **1a** (0.1 mmol), cat. (2.2 mg), H_2_ (40 bar), solvent (0.66 mL), and reaction time = 24 h. Yields were determined by GC-FID using mesitylene as internal standard. [M] = metal catalyst.

After our initial discovery, we studied the influence of the reaction conditions on the yield and HDS of the product (Table 1, for more detailed information, see the Supporting Information). A screen of different metal sulfide catalysts revealed that the employed primary metal is essential for the reaction outcome. While chromium and iron yielded hardly any product (Table S2), cobalt, rhodium, palladium, and ruthenium gave good conversions to the desired THQ, with ruthenium giving the best results (Table 1, entries 2–5).

Switching the solvents indicated that alcohols are essential for good conversions at lower temperatures (100 °C; Table 1, entry 6). MeOH proved to be optimal, giving full conversion even at temperatures of 80 °C (Table 1, entry 7). The addition of additives (Lewis and Bro̷nsted acids) gave no significant improvements (Table S6), while the composition of the catalyst was essential for the reaction outcome. Higher amounts of ruthenium in the catalyst also correlated to higher reactivity. Additionally, it was observed that Ru–W–S-0.33 showed higher reactivity than Ru–Mo–S-0.50, highlighting the importance of the employed secondary metal salt (Table 1, entries 9–10). To investigate the reproducibility of the reaction we also conducted a reaction-condition-based sensitivity screen, which underlined the robustness of this method (Table 2; see the Supporting Information for details).^32^

An assessment of the catalytic activity of Ru–W–S-0.33, Ru–S–SO_4_, and Ru–S revealed an interesting trend. While Ru–S gave only a poor yield of 32%, Ru–S–SO_4_ showed improved activity and yielded **2a** in 76% under optimized conditions, Ru–W–S-0.33 gave 96% yield (see Table 1). This underlines the significance of the oxidized sulfur species coordinating to the catalyst surface and dramatically improving the catalytic activity. It is particularly interesting to note that the catalytic activity increases with the amount of oxidized sulfur present in the catalyst, as higher levels of sulfates or sulfites appear to correlate with higher catalytic activity.

**Figure 5:**
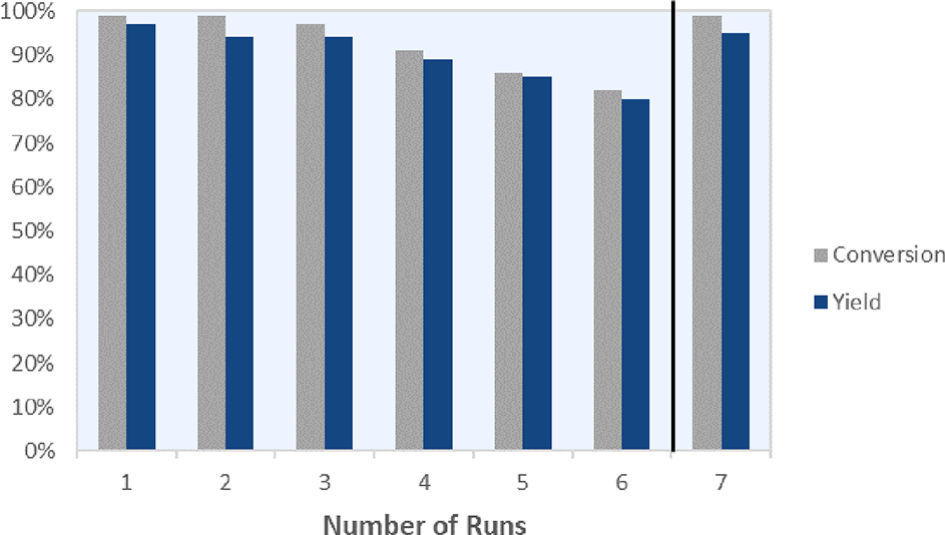
Catalyst recycling experiments for the hydrogenation of quinoline **1a** to THQ **2a** with conversions and yields for seven runs. The reaction time was prolonged to 48 h for the seventh run.

As these metal-sulfide-catalysts are susceptible to catalyst leaching during hydrogenation reactions, resulting in a significant decline in catalyst activity,^17^ we wanted to examine the recyclability of our newly synthesized Ru–W–S-0.33 catalyst (Figure 5). To our delight, no evident catalyst poisoning and only a slight reduction in catalytic activity was observed after multiple reaction cycles. Even after 6 cycles, the catalyst maintained good conversion and yielded **2a** in 80%. Additionally, full conversion of **1a** could be achieved again by extending the reaction time to 48 h.

Having the optimized conditions in hand, we wanted to explore the scope of this catalytic protocol (Table 2). Although we established a reaction temperature of 60 °C for substrate **1a**, a temperature of 80 °C provided optimal results for more challenging quinolines. To our delight, the introduction of sterically more demanding substituents on the N-heterocycle did not diminish the reaction yield (**2a**–**2c**). When varying the steric bulk of the thioether substituent (**2d**) or the substitution pattern of the alkyl and thioether (**2e** + **2f**), we observed no significant impact on the reaction outcome, and the corresponding THQs were obtained in high yields. Additionally, disubstituted THQs were successfully synthesized in high yields, albeit at higher reaction temperatures of 100 °C (**2g**–**2i**). We were particularly pleased that our method was capable of chemoselectively hydrogenating the quinoline core and simultaneously preserving other unsaturated moieties highly susceptible to reduction. The tolerance of an alkene in arene hydrogenation has been rarely demonstrated,^33,34^ yet our conditions retained the unsaturated bond, affording **2h** in 72% yield. Furthermore, the catalyst selectively reduced the N-heterocycle while maintaining the aromaticity of other aromatic ring systems (**2i**–**2o**). In particular, rings with low aromaticity such as furans (**2o**) and thiophenes (**2n**) are well conserved, with **2n** even possessing two Lewis basic sulfur atoms, again highlighting the catalyst’s remarkable sulfur resistance. Although halogens constitute useful synthetic handles, hydrogenation of halogen substituted substrates tends to be challenging due to competing hydrodehalogenation pathways.^35^ Utilizing our catalytic protocol, substrates **2k**–**2m** and **2p** could be obtained without any loss in yield or dehalogenation observed.

**Table 2:**
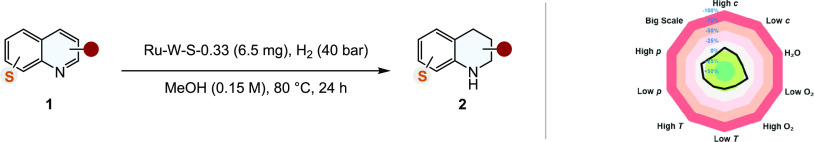

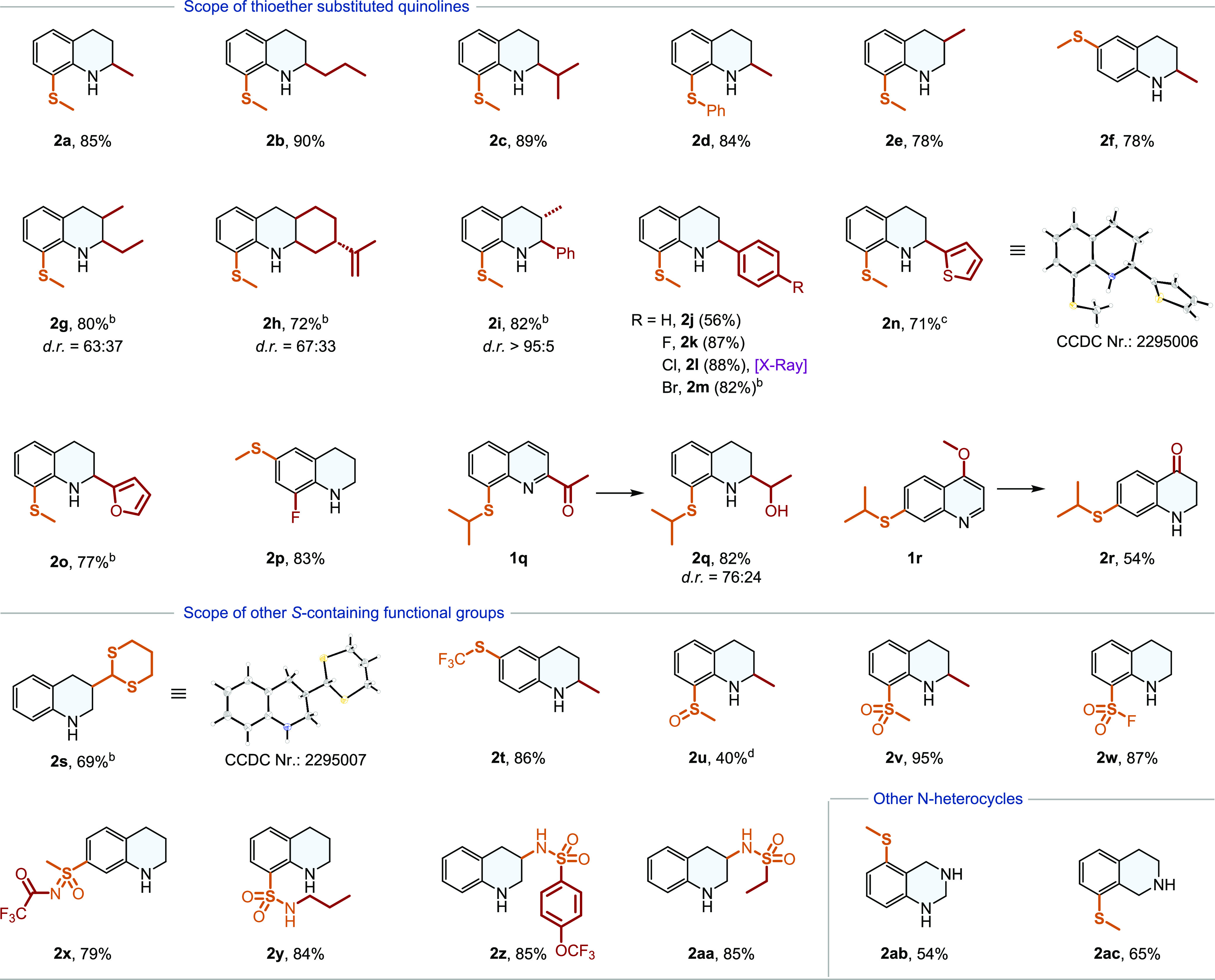
Substrate Scope for the Hydrogenation of Sulfur-Containing Quinolines under Optimized Conditions^a^ and the Reaction Condition-Based Sensitivity Assessment

a Reaction conditions: 1 (0.3 mmol), Ru–W–S-0.33 (6.5 mg), H_2_ (40 bar), T = 80 °C, MeOH (2 mL) and reaction time = 24 h. Isolated yields are given, the diastereomeric ratio (d.r.) was determined by NMR.

b *T* = 100 °C.

c *T* = 120 °C.

d *T* = 60 °C.

However, it is worth mentioning that a carbonyl group directly attached to the N-heterocycle was not tolerated and reduced to the corresponding alcohol, yielding **2q** in good yield. Interestingly, the hydrogenation of **1r** did not yield the methoxy substituted THQ but rather the tetrahydroquinolinone **2r**. This outcome can likely be attributed to the deoxygenation of the methoxy methyl during hydrogenation, followed by a tautomerization to yield **2r** in moderate yield. Other unsuccessful substrates include electron withdrawing substituents such as fluorine or trifluoromethyl groups directly attached to the N-heterocycle. These motifs showed no conversion under the standard conditions. In addition, highly sensitive nitrile, nitro, and alkyne groups were not tolerated and were reduced to their respective saturated moieties. Sulfur moieties directly attached to the N-heterocycle were also cleaved during the hydrogenation reaction. Further details of unsuccessful substrates or substrates that did not yield the desired THQ are given in the Supporting Information.

Intrigued by the high level of chemoselectivity and sulfur tolerance of the catalyst, we subjected different sulfur functionalities to the hydrogenation conditions. Dithianes, which bear two Lewis basic sulfur atoms and are protecting groups for easily reducible aldehydes, are tolerated well (**2s**). Sulfoxides are known to undergo reduction to thioethers under a hydrogen atmosphere.^36^ Although we also observed significant sulfoxide reduction at 80 °C, we successfully obtained **2u** in a moderate yield at 60 °C. THQs containing medicinally relevant sulfone (**2v**) and sulfonamide (**2y**-**2aa**) moieties were synthesized in excellent yields ranging from 84% to 95%. Sulfonyl fluoride substituted quinolines reacted smoothly, giving THQ **2w** in 87% yield. Other noteworthy motifs in medicinal chemistry are sulfoximines,^5^ and pleasantly, this functional group was also well tolerated (**2x**) without any reduction of the sulfoximine observed. Isoquinolines and quinazolines, despite being closely related to quinolines, tend to be more challenging to hydrogenate due to their lower reactivity and strong coordination to the catalyst.^37^ To our delight, our method could be further extended to these challenging N-heteroarenes, affording **2ab** + **2ac** in good yields.

**Scheme 2:**
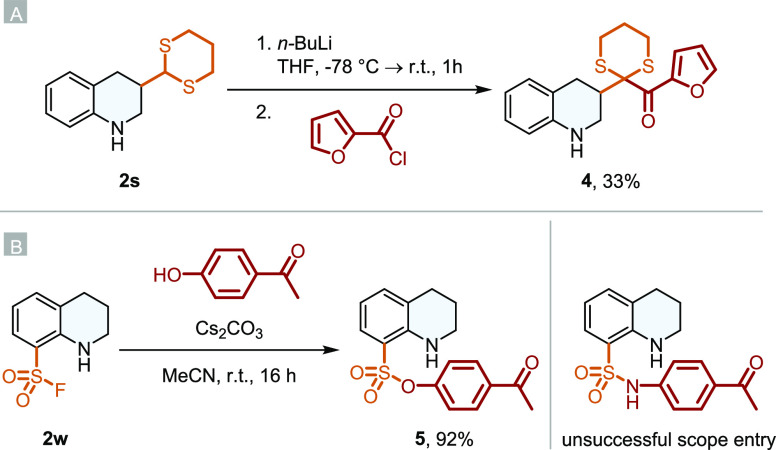
Downstream Product Modifications: (A) Corey–Seebach Type Umpolung of Dithianes; (B) SuFEx Click Reaction of **2w**

Finally, we wanted to demonstrate the utility of the sulfur functionalities by providing some downstream product modifications (Scheme 2). Dithianes can be seen as protecting groups for carbonyl compounds and can be used for Corey–Seebach-type umpolung reactions.^38^ Since carbonyls are often labile under hydrogenation conditions (see **2q**), the tolerance of dithianes offers new possibilities to retain carbonyl moieties under a hydrogen atmosphere and offers the chance to do further umpolung reactions to obtain more complex structures. By adding *n*-BuLi to **2s** and subsequently 2-furoyl chloride, THQ **4** was successfully obtained in moderate yield due to a competing reaction with the nitrogen (Scheme 2A). Further known deprotection methods with, e.g., mercury result in the formation of a dicarbonyl compound, a structural motif highly vulnerable to reducing conditions. Additional functionalization of the sulfonyl fluoride **2w** utilizing SuFEx click-chemistry afforded THQ **5** in excellent yield (Scheme 2B). This simple reaction can therefore be exploited to introduce reductively labile groups into the THQ.

## Conclusions

In summary, we developed the first sulfur tolerant hydrogenation of quinolines under mild conditions for the facile synthesis of valuable 1,2,3,4-tetrahydroquinolines. A novel unsupported Ru–S catalyst was synthesized and characterized by DLS, XPS, ICP-OES, TEM, HAADF-STEM, and EDX elemental mapping. The chemoselectivity and sulfur resistance of the catalytic protocol was demonstrated by a diverse substrate scope. Mild reaction conditions enable a broad functional group tolerance, including reductively labile groups like (hetero)arenes, halides, and olefins. Also, the tolerance of various poisoning (e.g., thioethers, dithianes, thiophenes, and sulfoxides) and medicinally relevant (e.g., sulfones, sulfonyl fluorides, sulfoximines, and sulfonamides) sulfur functionalities was shown. Further downstream product modifications underline the utility of the method, synthesizing product motifs, which are otherwise inaccessible under standard hydrogenation conditions. As the relevance of sulfur in medicinal chemistry increases, catalytic systems that are tolerant to diverse sulfur functionalities become more sought after. We see huge potential in the development of novel catalysts that combine resistance to sulfur poisoning with hydrogenation activity to increase molecular complexity in a single step.

## Supporting Information Available

All experimental procedures, characterizations, and crystallographic data (PDF)
