# Supplementary material for: Chemoselective Heterogeneous Hydrogenation of Sulfur Containing Quinolines under Mild Conditions
Source: J Am Chem Soc. 2024 Feb 20;146(9):5864–71. doi: 10.1021/jacs.3c11163 (PMC10921411; doi:10.1021/jacs.3c11163)

Supporting Information

Chemoselective Heterogeneous Hydrogenation of Sulfur Containing Quinolines Under Mild Conditions

Lukas Lückemeier[‡,1], Thijs De Vos[‡,1], Lisa Schlichter[2], Christian Gutheil[1], Constantin G. Daniliuc[1] and Frank Glorius[*,1]

[1]Universität Münster, Organisch-Chemisches Institut, Corrensstraße 36, 48149 Münster (Germany).

[2]Universität Münster, Center for Soft Nanoscience (SoN) and Organisch-Chemisches Institut, Busso-Peus-Straße 10, 48149 Münster (Germany).

Corresponding author: glorius@uni-muenster.de

Table of Contents

1. General 2
2. Synthesis and Analysis of Catalysts 3
   2.1 Synthesis of Catalysts 3
   2.2 X-Ray Photoelectron Spectroscopy (XPS) 4
   2.3 Dynamic Light Scattering (DLS) Measurements 7
   2.4 Transmission Electron Microscopy (TEM), High-Angle Annular Dark-Field Scanning Transmission Electron Microscopy (HAADF-STEM) and EDX Elemental Mapping 8
   2.5 ICP-OES Measurement of Tungsten 12
3. Synthesis of Starting Materials 13
4. Investigation of Reaction Parameters 25
   4.1 Screen of Different Catalysts 25
   4.2 Screen of Different Solvents 26
   4.3 Screen of Different Additives 27
   4.4 Screen of Different Ru-catalysts 28
5. Catalyst Recycling Experiments 28
6. Reaction-Condition-Based Sensitivity Assessment 29
7. Catalytic Hydrogenation Reactions 31
8. Unsuccessful Substrates 41
9. Diversifications of Products 2s and 2w 42
   9.1 Corey-Seebach Type Umpolung of 2s 42
   9.2 SuFEx Click Reaction of 2w 42
10. Crystallographic Data 43
11. References 45
12. NMR Spectra 48

1. General

All reagents were obtained commercially unless otherwise noted. Unless otherwise noted, all reactions were carried out under an atmosphere of air. The employed solvents were either dried by distillation over standard drying agents and stored under argon over molecular sieves or directly used from a solvent purification system (HPLC grade, dried via an alumina/molecular sieves column under positive argon pressure; *n*-hexane, dichloromethane, toluene, DMF, diethyl ether, MeCN, THF, MeOH). Catalytic hydrogenation reactions were prepared under air, unless otherwise noted, and carried out in Berghof High Pressure Reactors using hydrogen gas. Reaction temperatures are reported as the temperature of the bath surrounding the vessel unless otherwise stated.

Analytical thin layer chromatography (TLC) was performed on silica gel 60 F254 aluminum plates (Merck). TLC plates were visualized by exposure to short wave ultraviolet light (254nm, 366nm) or by staining with a solution of $KMnO_4$. Flash chromatography was performed on Acros Organics silica gel (35-70 mesh) under a positive pressure of compressed air, eluting with the specified solvent system.

GC-MS spectra were recorded on an Agilent Technologies 7890A GC-system with an Agilent 5975C VL MSD or an Agilent 5975 inert Mass Selective Detector (EI) and a HP-5MS column (0.25 mm x 30 m, film: 0.25 μm). ESI mass spectra were recorded on a Bruker Daltonics MicroTof spectrometer. APCI mass spectra were recorded on a Thermo Fisher Scientific Orbitrap LTQ XL. ^{1}H, ^{13}C, ^{19}F spectra were recorded on a Bruker Avance II300 or Avance II400, AgilentDD2 500 or AgilentDD2 600 in the indicated solvents. Chemical shifts (δ) are given in ppm relative to TMS. The residual solvent signals were used as references and the chemical shifts converted to the TMS scale ($CDCl_3$: δ_H = 7.26 ppm, δ_C = 77.16 ppm; DMSO-d_6: δ_H = 2.50 ppm and δ_C = 39.52 ppm). ^{19}F-NMR spectra were not calibrated by an internal reference and the chemical shift δ (ppm) is given relative to CCl_3F. Multiplicities are reported using the following abbreviations: s = singlet, d = doublet, t = triplet, q = quartet, quint = quintet, hept = heptet, m = multiplet, br = broad resonance.

Transmission electron microscopy (TEM) experiments were performed using a Thermo Fisher Scientific FEI Themis G3 60-300 transmission electron microscope (Thermo Fisher, Waltham, Massachusetts, US) equipped with a high brightness field emission gun (X-FEG), a monochromator, a quadrupole energy-dispersive X-ray system, a high-angle annular dark field detector (Fishione Model 3000), an image Cs-corrector, and a fast CMOS camera (Ceta 2 speed upgrade 4k x 4k). The microscope was operated at an acceleration voltage of 300 kV. Samples were measured on carbon coated copper grids (Plano EM, S160). Images were analyzed using TIA version 4.5 (FEI) and ImageJ version 1.50i (National Institutes of Health, US, Java 1.8.0_77).

DLS measurements were performed on a Nano ZS Zetasizer (MalvernInstruments LTD., Worcestershire, UK) in a 1 mL quartz glass cuvette with a path length of 1 cm. The samples were measured at 20 °C, equilibrated at this temperature for 2 minutes prior to the measurement. Measurements were done in triplicate with 10-15 runs per single measurement as optimized by the instrument.

2. Synthesis and Analysis of Catalysts

2.1 Synthesis of Catalysts

General Procedure 1 (GP1): According to a literature procedure by Corma and coworkers,[1] the metal salt, $(NH_4)_6Mo_7O_{24}$ · $4H_2O$ or Na_2WO_4 · $2H_2O$ and sulfur were dispersed in H_2O (55 mL) in a stainless steel autoclave. N_2H_4·H_2O (80%, 6.75 mL, 0.110 mol) was added and the autoclave was sealed tightly. The mixture was stirred at 180 °C for 22 h and after cooling down, the autoclave was depressurized carefully. The black precipitate was filtered off and washed with H_2O, EtOH and Et_2O (three times each). The resulting black powder was dried under vacuum and stored under argon.

The denotation of the catalysts follows the rule: [M]-Mo-S-X or [M]-W-S-X, with X = [M] / ([M]+Mo) mole ratio or X = [M] / ([M]+W) mole ratio. [M] = metal of the employed metal salt.

Catalyst **Ru-Mo-S-0.33** was synthesized according to **GP1** from $RuCl_3$ · xH_2O (479 mg, 2.12 mmol, 1.0 equiv.), $(NH_4)_6Mo_7O_{24}$ (750 mg, 0.610 mmol 2.0 equiv.), sulfur (284 mg, 8.84 mmol, 4.0 equiv.) and N_2H_4·H_2O (80%, 6.75 mL, 0.110 mol).

Catalyst **Ru-Mo-S-0.50** was synthesized according to **GP1** from $RuCl_3$ · xH_2O (479 mg, 2.12 mmol, 1.0 equiv.), $(NH_4)_6Mo_7O_{24}$ (375 mg, 0.300 mmol, 1.0 equiv.), sulfur (142 mg, 4.42 mmol, 2.0 equiv.) and N_2H_4·H_2O (80%, 3.38 mL, 55.0 mmol).

Catalyst **Ru-W-S-0.33** was synthesized according to **GP1** from $RuCl_3$ · xH_2O (479 mg, 2.12 mmol, 1.0 equiv.), Na_2WO_4 · H_2O (1.40 g, 4.24 mmol, 2.0 equiv.), sulfur (284 mg, 8.84 mmol, 4.0 equiv.) and N_2H_4·H_2O (80%, 6.75 mL, 0.110 mol).

Catalyst **Co-Mo-S-0.50** was synthesized according to **GP1** from $Co(NO_3)_2$ · $6H_2O$ (1.31 g, 4.50 mmol, 1.0 equiv.), $(NH_4)_6Mo_7O_{24}$ (750 mg, 0.610 mmol 2.0 equiv.), sulfur (284 mg, 8.84 mmol, 2.0 equiv.) and N_2H_4·H_2O (80%, 6.75 mL, 0.110 mol).

Catalyst **Rh-Mo-S-0.33** was synthesized according to **GP1** from $[Rh(COD)Cl]_2$ (261 mg, 0.530 mmol, 1.0 equiv.), $(NH_4)_6Mo_7O_{24}$ (375 mg, 0.300 mmol, 1.0 equiv.), sulfur (142 mg, 4.42 mmol, 4.0 equiv.) and N_2H_4·H_2O (80%, 3.38 mL, 55.0 mmol).

Catalyst **Pd-Mo-S-0.33** was synthesized according to **GP1** from $Pd(OAc)_2$ (476 mg, 2.12 mmol, 1.0 equiv.), $(NH_4)_6Mo_7O_{24}$ (750 mg, 0.610 mmol 2.0 equiv.), sulfur (284 mg, 8.84 mmol, 4.0 equiv.) and N_2H_4·H_2O (80%, 6.75 mL, 0.110 mol).

Catalyst **Cr-Mo-S-0.50** was synthesized according to **GP1** from $CrCl_2$ (543 mg, 4.42 mmol, 1.0 equiv.), $(NH_4)_6Mo_7O_{24}$ (750 mg, 0.610 mmol 2.0 equiv.), sulfur (284 mg, 8.84 mmol, 2.0 equiv.) and N_2H_4·H_2O (80%, 6.75 mL, 0.110 mol).

Catalyst **Fe-Mo-S-0.33** was synthesized according to **GP1** from $FeCl_3$ · $6H_2O$ (573 mg, 2.12 mmol, 1.0 equiv.), $(NH_4)_6Mo_7O_{24}$ (750 mg, 0.610 mmol 2.0 equiv.), sulfur (284 mg, 8.84 mmol, 4.0 equiv.) and N_2H_4·H_2O (80%, 6.75 mL, 0.110 mol).

Catalyst **Ru-S** was synthesized according to **GP1** from $RuCl_3$ · xH_2O (956 mg, 4.24 mmol, 1.0 equiv.), sulfur (284 mg, 8.84 mmol, 2.0 equiv.) and N_2H_4·H_2O (80%, 6.75 mL, 0.110 mol).

Catalyst **Ru-S-SO_4** was synthesized according to **GP1** from $RuCl_3$ · xH_2O (239 mg, 1.06 mmol, 1.0 equiv.), sulfur (68 mg, 2.12 mmol, 2.0 equiv.), Na_2SO_4 (301 mg, 2.12 mmol, 2.0 equiv.) and N_2H_4·H_2O (80%, 2.70 mL, 55.5 mmol).

2.2 X-Ray Photoelectron Spectroscopy (XPS)

XPS measurements were performed on a Thermo Fisher Scientific K-Alpha instrument (Organisch-Chemisches Institut, Corrensstr. 36, 48149 Münster). The instrument parameters are summarized in Table S1. All spectra were referenced to adventitious C1s (284.8 eV). The spectra were analyzed by use of Avantage 5.9925 (Thermo Fisher Scientific), and fitting of S2p spectra was done by comparison to XPS database sets[2] and further literature examples.[3,4] Data visualization was done in Origin Pro 2023.

Table S1: XPS measurement parameters.

XPS	X-Ray Source	Spot and Detection	Charge Compensation	Pass Energy / eV	Step Size / eV	Dwell Time / ms	Number of Scans
Survey	Al Kα, 12 kV filament	100 μm (72W max.). 60° X-ray incidence angle, 0° take-off angle (rel. to surface normal)	Flood gun	200	0.9	100	2
Ru3d				50	0.05	50	5
W4f							
Mo3d							
C1s							
N1s							
O1s							
S2p							

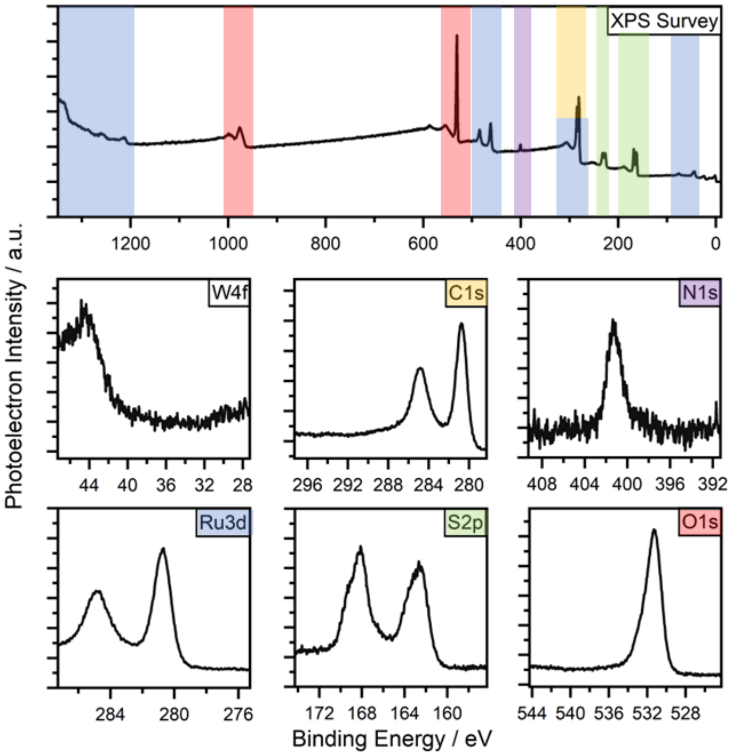

Figure S1: XPS measurement of Ru-W-S-0.33.

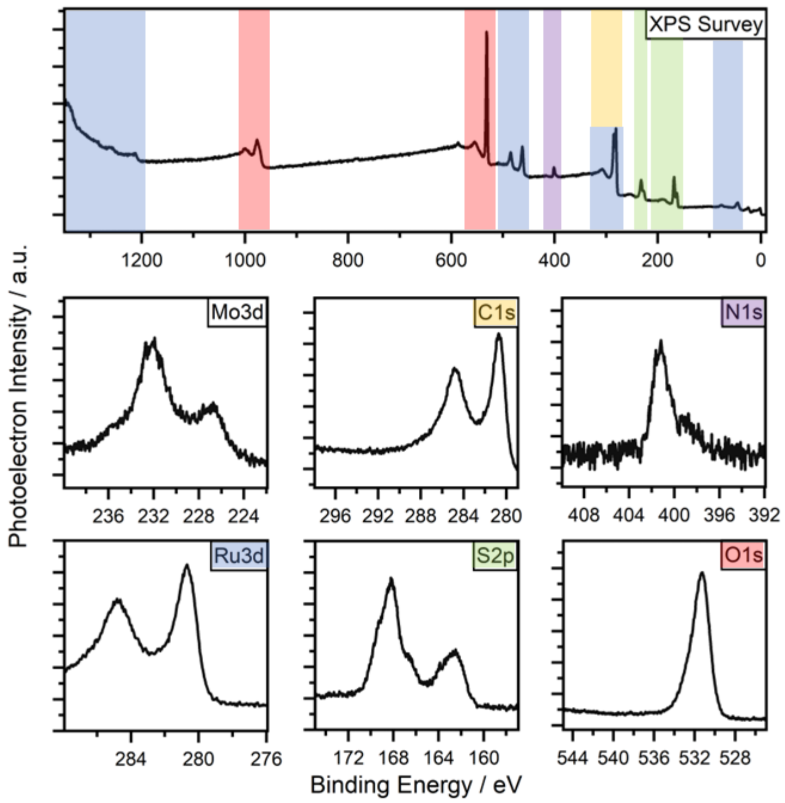

Figure S2: XPS measurement of Ru-Mo-S-0.50.

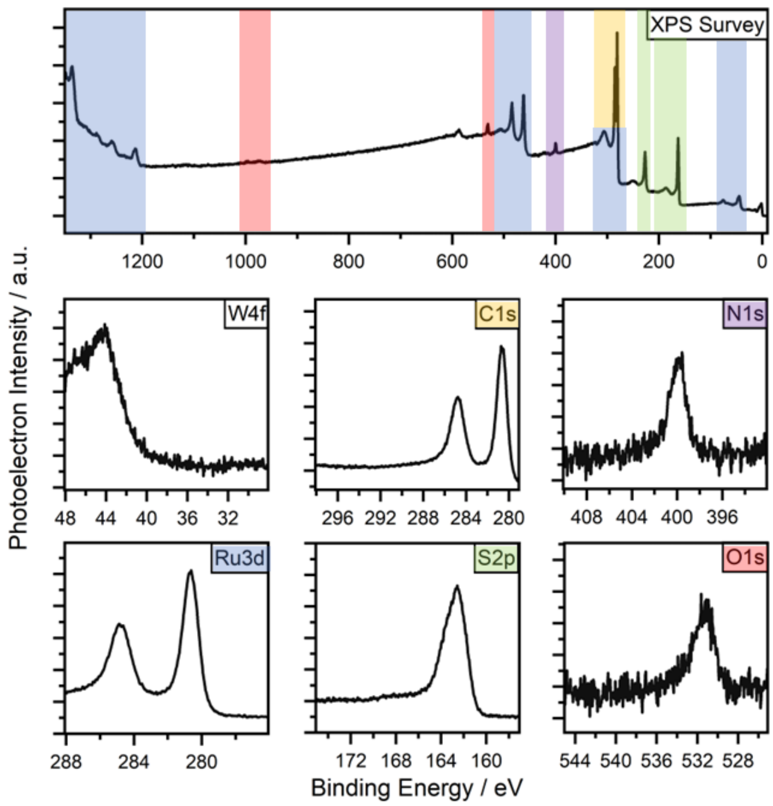

Figure S3: XPS measurement of Ru-S.

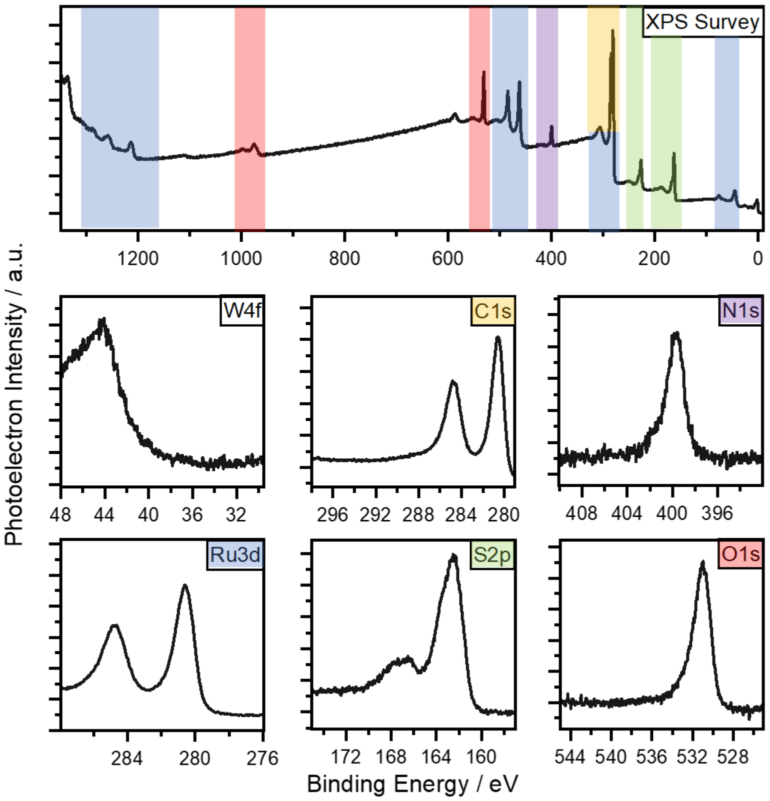

Figure S4: XPS measurement of Ru-S-SO_4.

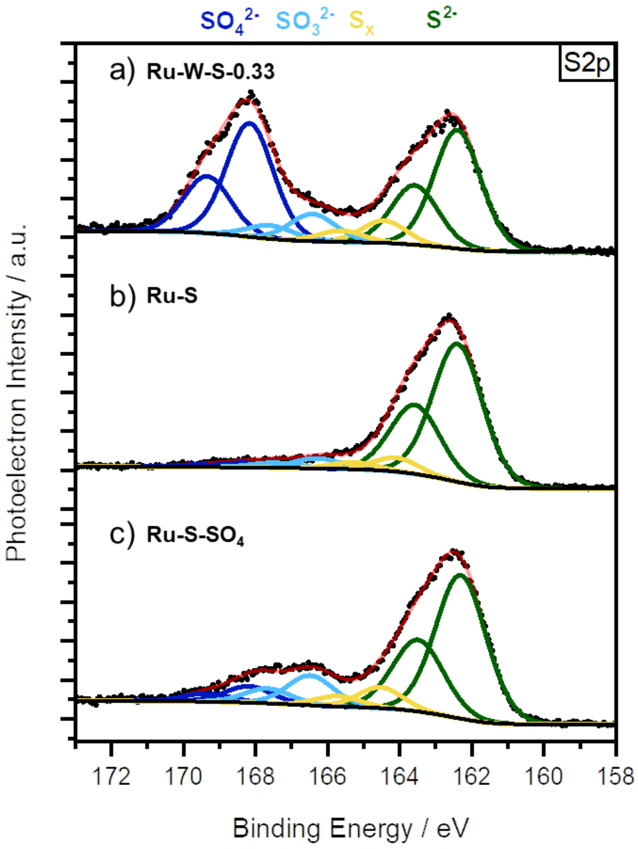

Figure S5: A comparison of the XPS spectra in the sulfur region of Ru-W-S-0.33, Ru-S and Ru-S-SO_4.

XPS analysis of selected ruthenium catalysts revealed that neither Ru-W-S-0.33 nor Ru-Mo-S-0.50 contain tungsten or molybdenum. Typically, the W4f electron binding energy can be detected between 31 and 36 eV, yet no signal was detected in this region for Ru-W-S-0.33 (Figure S1). The Mo3d electron binding energy is expected to occur between 227-235 eV. Although two signals can be detected in this region, their relative intensities do not match the expected ratios corresponding to the Mo($3d_{3/2}$) and Mo($3d_{5/2}$) electron binding energies (Figure S2). The signals in this region can therefore more likely be related to sulfur, since overlap between the signals of the S2s and Mo3d electron binding energies is known to occur. S2p electron binding energies of sulfides are usually detected at lower energies of ~162 eV, whereas oxidized sulfur species, such as sulfites or sulfates, are detected at higher energies of ~169 eV. It can be seen that both catalysts, Ru-W-S-0.33 and Ru-Mo-S-0.50, consist of sulfides as well as higher oxidized sulfites/sulfates. In comparison to that, a Ru-S catalyst, synthesized the same way but without ammonium molybdate or sodium tungstate, does not contain any oxidized sulfur species (Figure S3). Hence, we hypothesize that the molybdate/tungstate rather serves as an oxidant that partially oxidizes the sulfur and therefore does not get reduced to MoS_2/WS_2-layers (as can be seen in the work of Corma and co-workers).[1]

2.3 Dynamic Light Scattering (DLS) Measurements

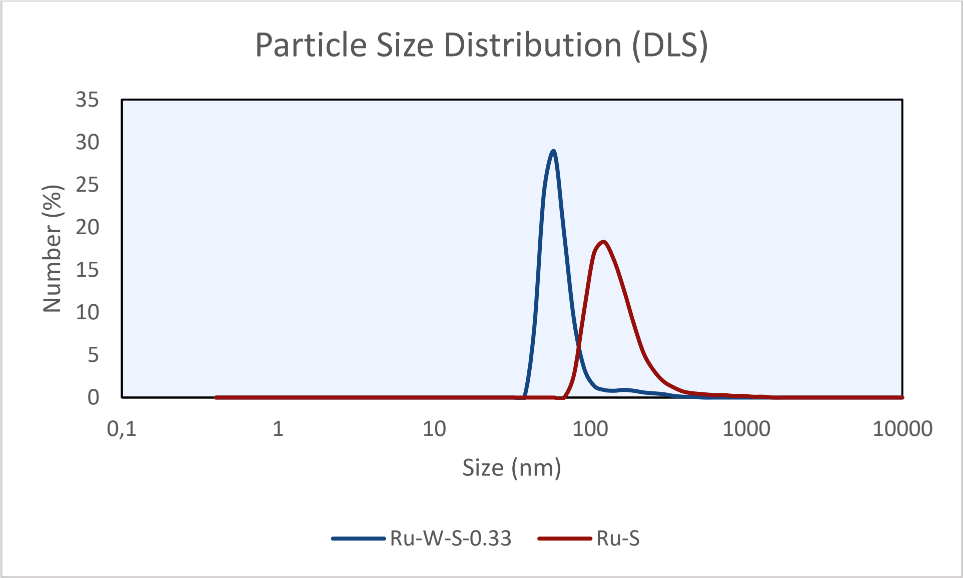

Figure S6: DLS measurements of Ru-W-S-0.33 and Ru-S.

Further analysis of the Ru-catalysts was performed *via* DLS measurements (Figure S6). The mean particle size of the Ru-W-S-0.33 catalyst in solution is ~69 nm (Figure S6, blue line). In comparison to that, the Ru-S catalyst has a mean particle size of ~159 nm, which is significantly higher (Figure S6, red line). A reason for that could be that the oxidized sulfur species of Ru-W-S-0.33 bind to the surface of the nanoparticles, which leads to stabilization and prevents agglomeration. The Ru-S catalyst, in contrast, does not contain any stabilizing ligands which leads to more agglomeration and therefore bigger nanoparticles. Also, it is to note that DLS only measures the size of the particles in solution including the solvation shell of the particles. Consequently, the measured size is significantly higher than the actual size of the nanoparticles, which can be determined by transmission electron microscopy (TEM).

2.4 Transmission Electron Microscopy (TEM), High-Angle Annular Dark-Field Scanning Transmission Electron Microscopy (HAADF-STEM) and EDX Elemental Mapping

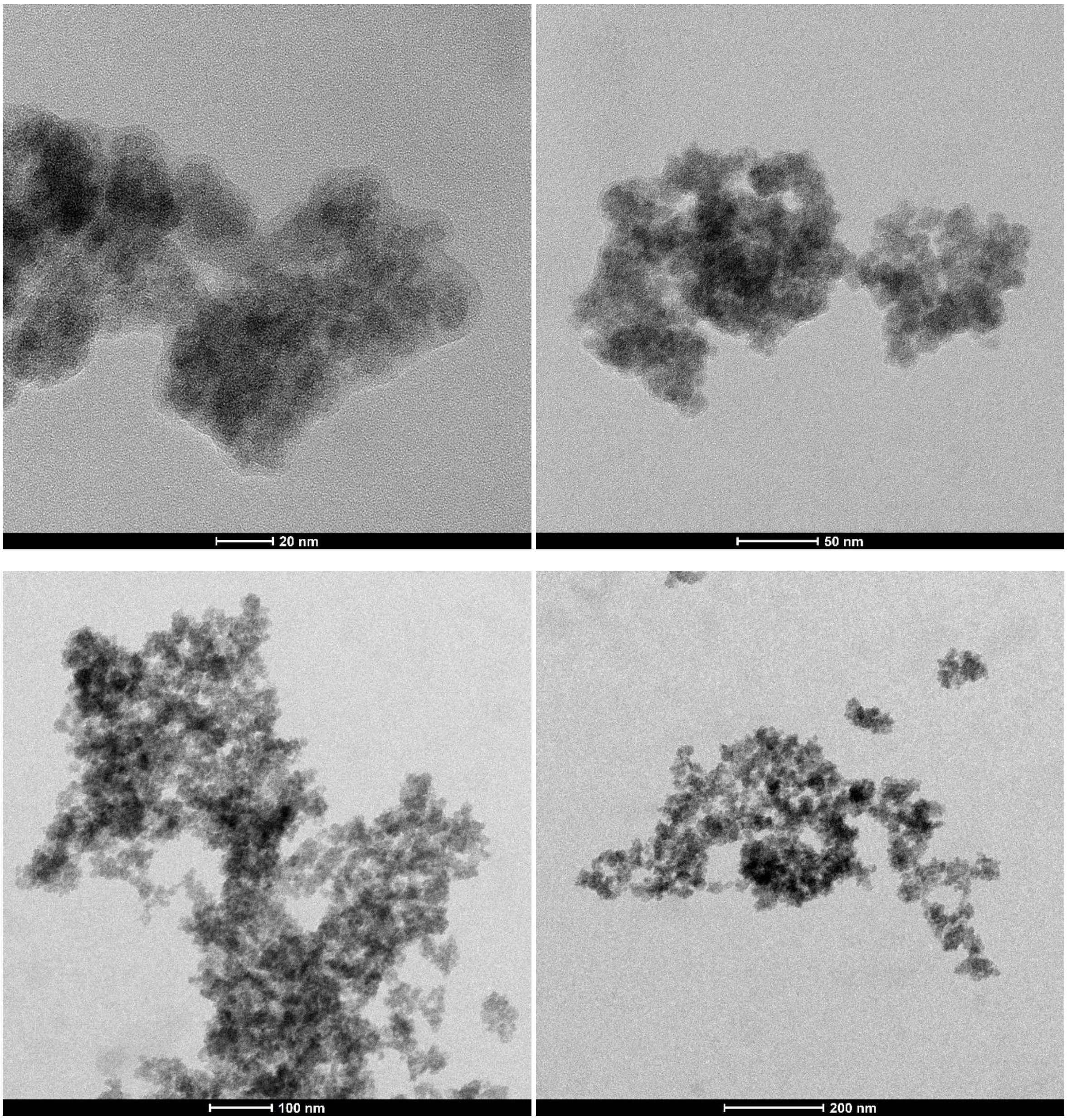

Figure S7: TEM images of Ru-W-S-0.33 at different magnifications.

Neither the TEM images of Ru-W-S-0.33 (Figure S7) nor the images of Ru-S (Figure S8) show fringe structures, which are typical for nanoparticles. This is either caused by agglomeration of the nanoparticles in dry conditions or the limited resolution of the TEM images. Nevertheless, the DLS measurements confirmed that both catalysts consist of nanoparticles, which might be stabilized in solution by solvent molecules.

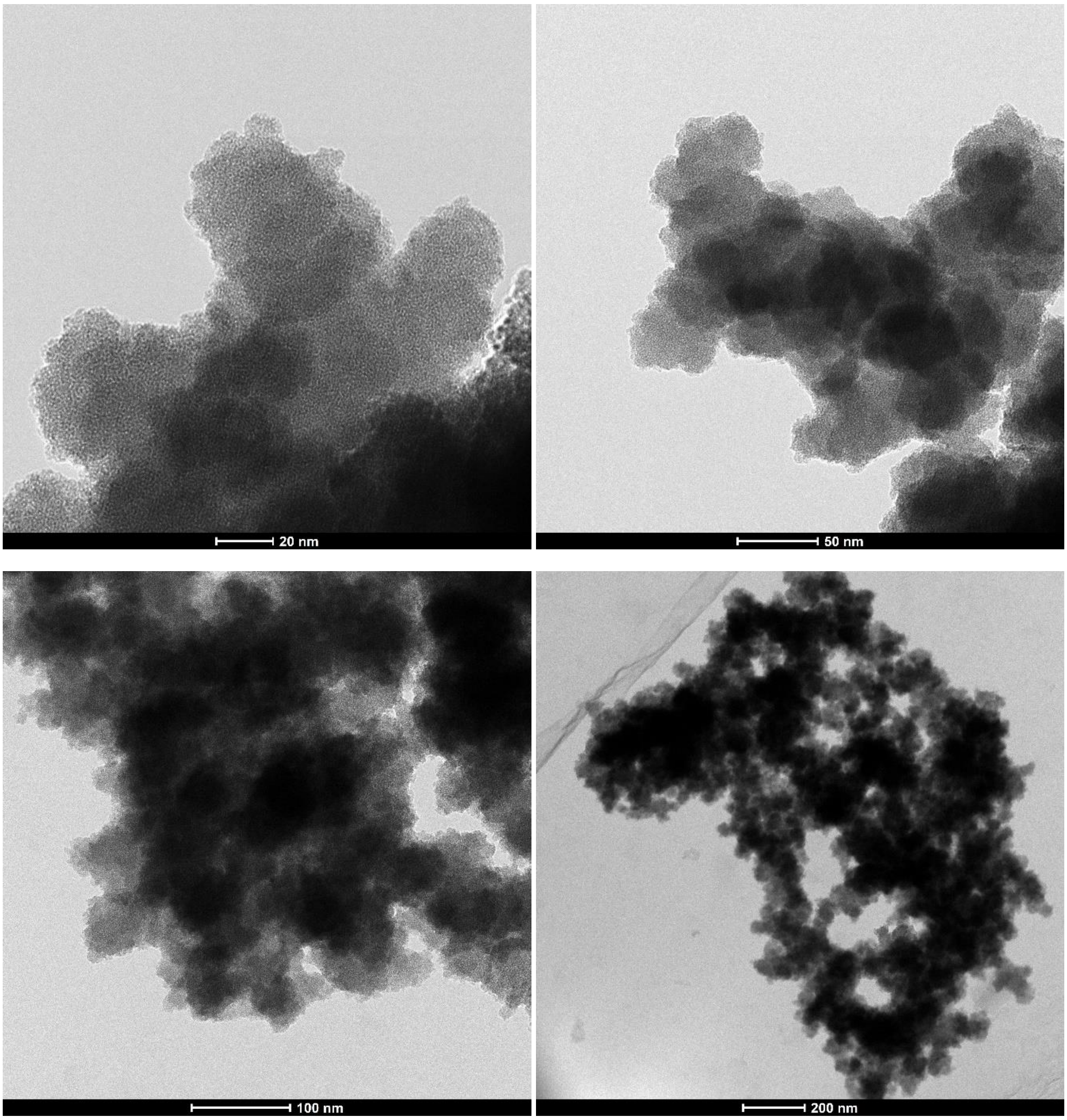

Figure S8: TEM images of Ru-S at different magnifications.

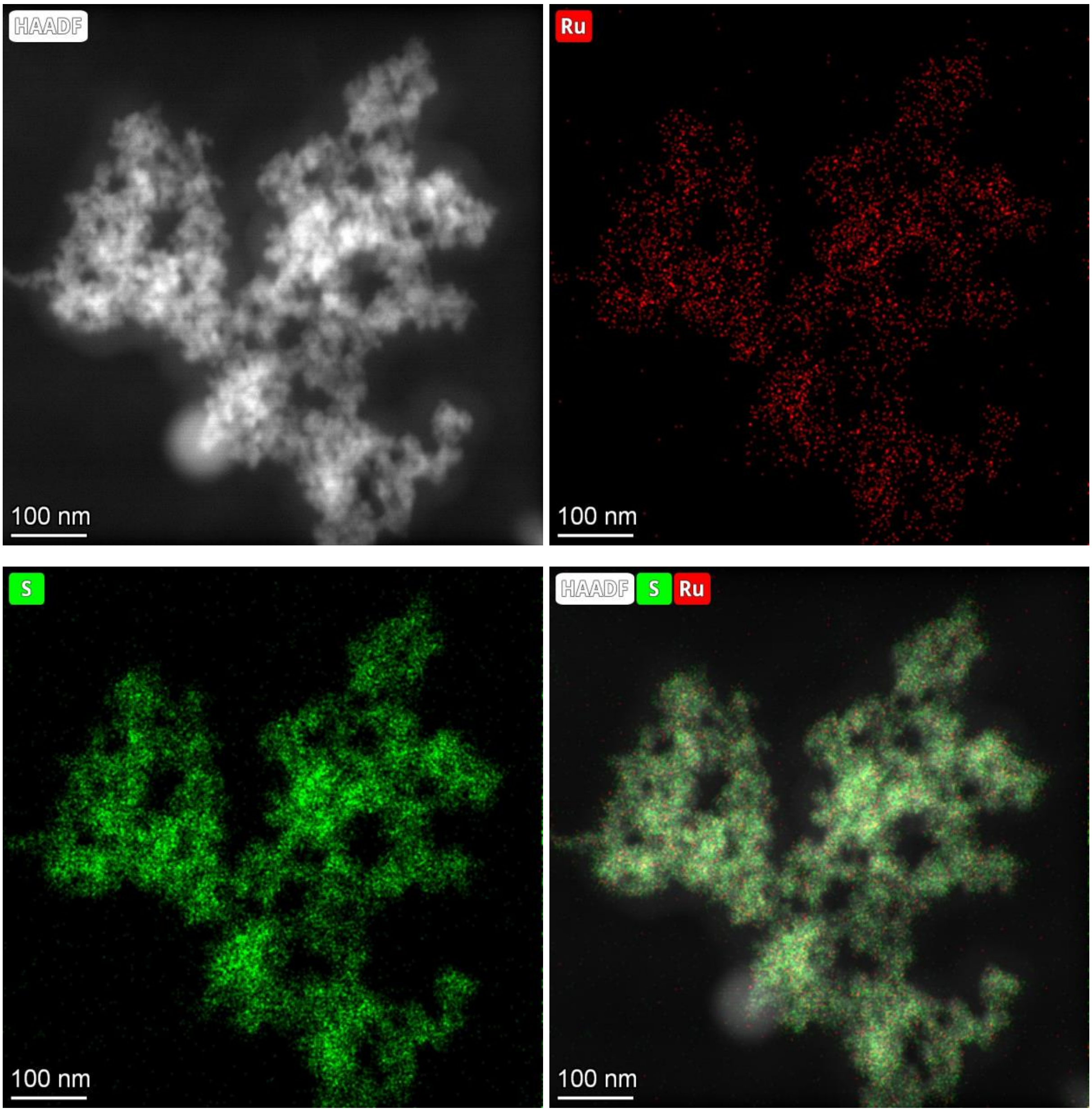

Figure S9: HAADF-STEM and EDX elemental mapping of Ru-W-S-0.33.

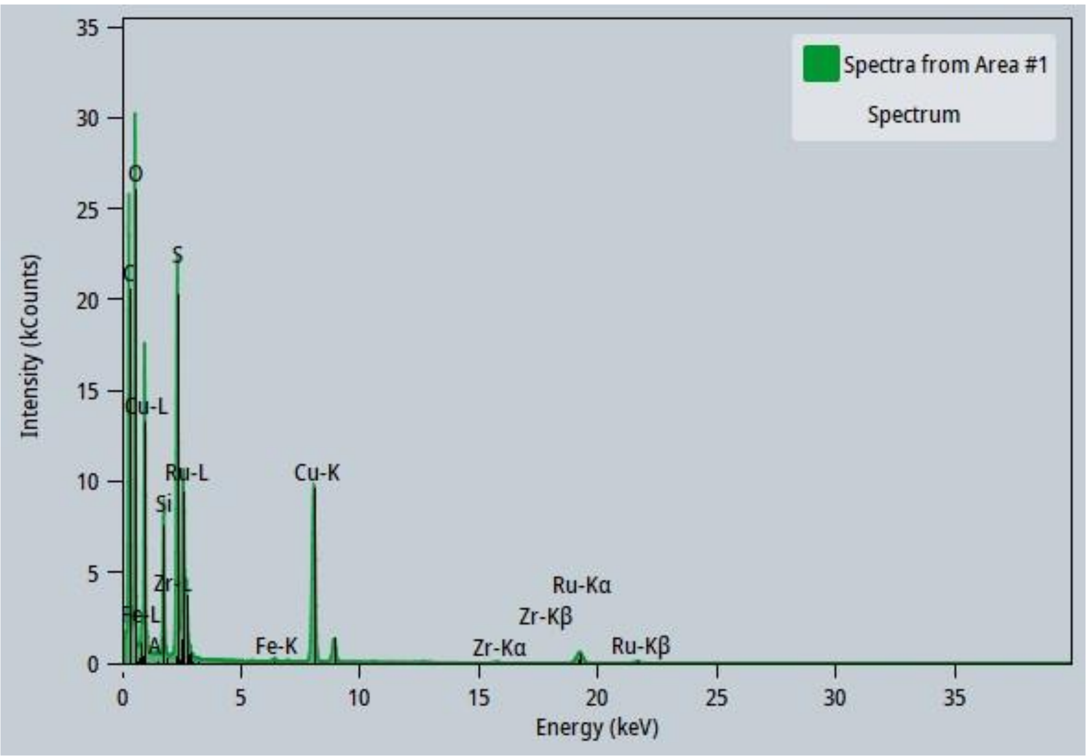

Figure S10: Energy diagram of the EDX elemental mapping of Ru-W-S-0.33.

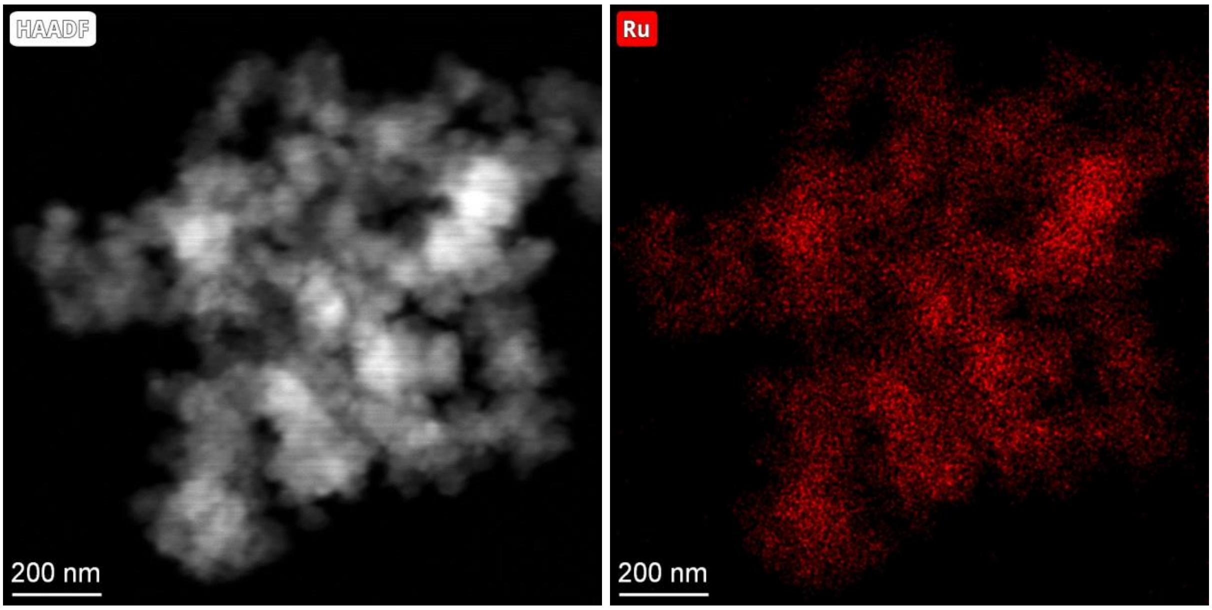

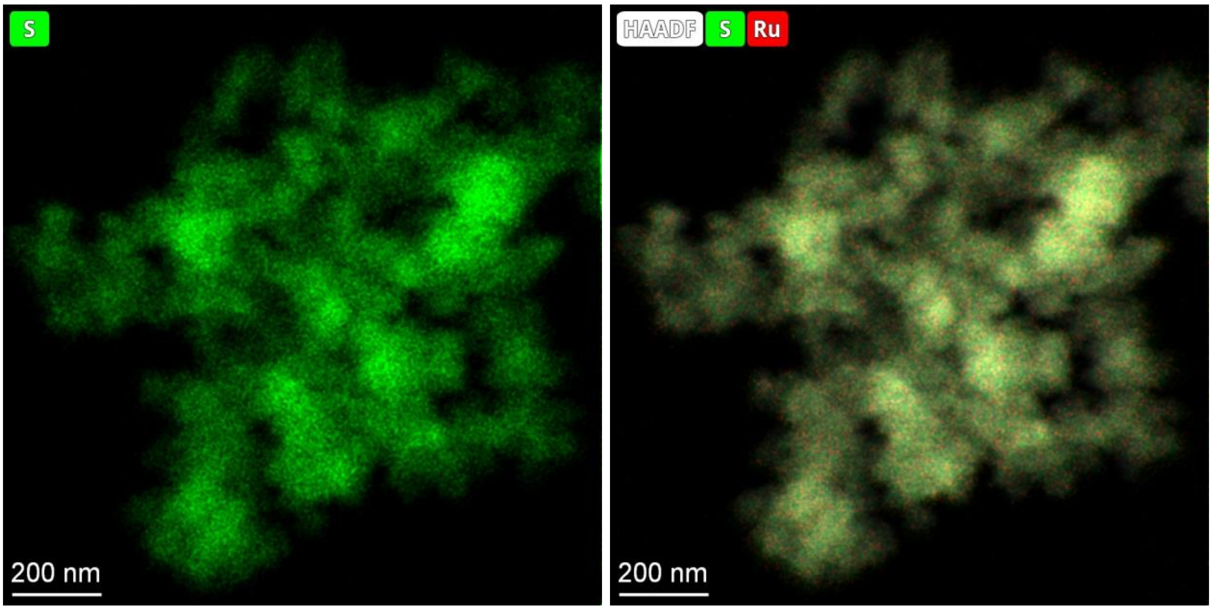

Figure S11: HAADF-STEM and EDX elemental mapping of Ru-S.

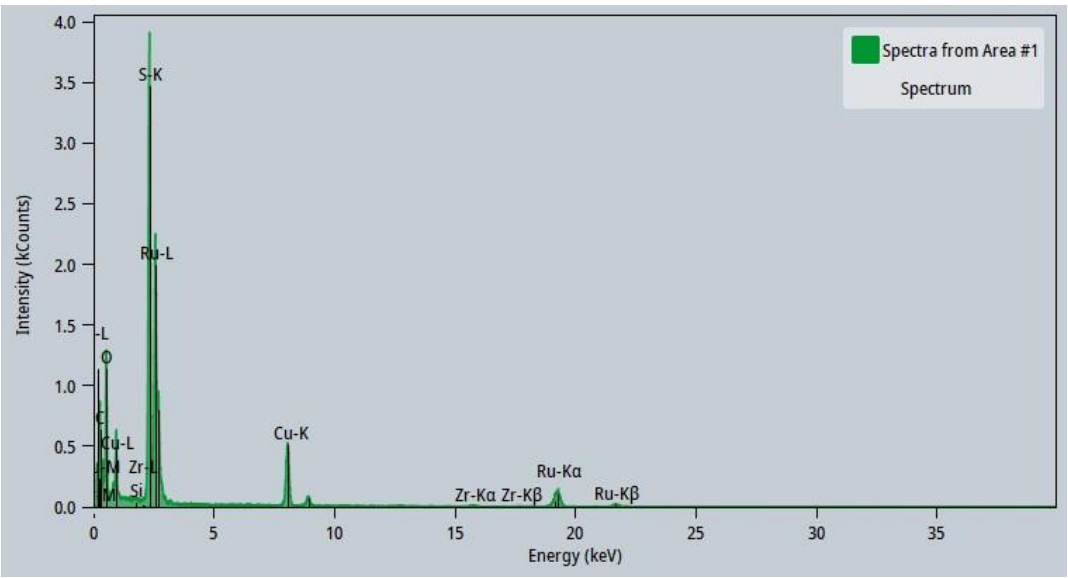

Figure S12: Energy diagram of the EDX elemental mapping of Ru-S.

2.5 ICP-OES Measurement of Tungsten

Three separately synthesized batches of Ru-W-S-0.33 were analyzed via ICP-OES to determine whether the catalyst contains a significant amount of tungsten. The measurements show that only background noise and no tungsten can be observed with ICP-OES (Figure S13) and the catalysts are therefore free of any tungsten.

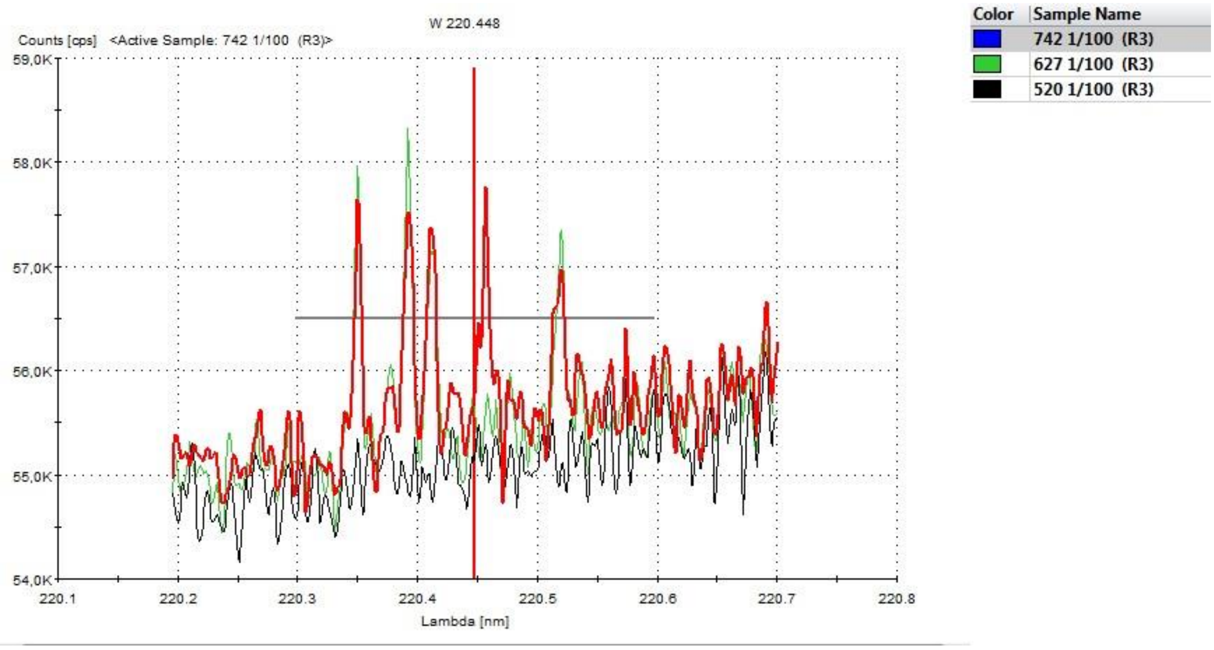

Figure S13: ICP-OES measurement of three different batches of Ru-W-S-0.33.

3. Synthesis of Starting Materials

General Procedure 2: Synthesis of Quinolines from Anilines and Acroleins (GP2)

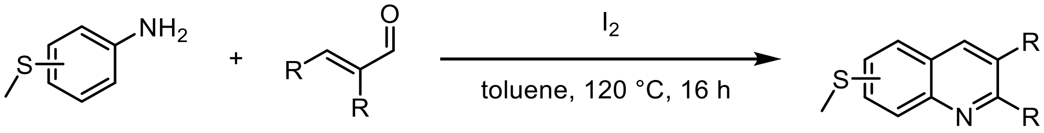

According to a literature procedure by Denmark *et al.*[5] In an oven-dried Schlenk tube the corresponding aniline (1.0 equiv.), acrolein (2.0 equiv.) and iodine (5 mol%) were dissolved in dry toluene (1.0 M) under argon and the reaction was refluxed at 120 °C for 16 h. The dark brown mixture was concentrated *in vacuo* and the crude was purified by column chromatography (cc) to yield the corresponding quinoline.

General Procedure 3: Synthesis of Quinolines from the Corresponding Bromoquinoline (GP3)

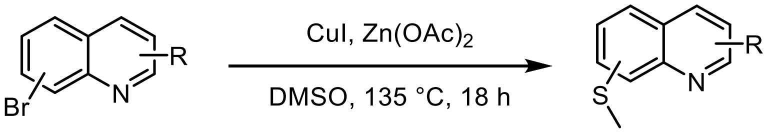

According to a literature procedure by Kantam and co-workers.[6] In an oven-dried Schlenk tube the corresponding quinoline (1.0 equiv.), $Zn(OAc)_2$ (2.0 equiv.) and CuI (25 mol%) were suspended under argon in dry DMSO (0.33 M) and the mixture was stirred at 135 °C for 18 h. The suspension was filtered over Celite®, the filtrate washed with H_2O and the aqueous phase was further extracted with Et_2O. The combined organics were dried over $MgSO_4$, concentrated *in vacuo* and the crude was purified by column chromatography to yield the corresponding quinoline.

General Procedure 4: Synthesis of Sulfonamides from Anilines and Sulfonyl Chloride (GP4)

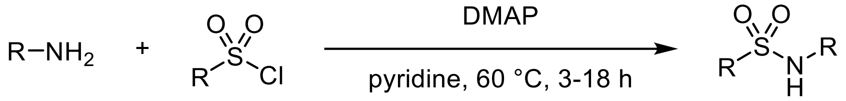

In an oven-dried Schlenk tube the corresponding sulfonyl chloride (1.1 equiv.), aniline (1.0 equiv.) and DMAP (10 mol%) were dissolved in pyridine (0.2 M) under argon and the mixture was stirred at 60 °C for 3–18 h. H_2O and brine were added, the organic layer was separated and the aqueous phase was extracted with EtOAc. The combined organic layers were dried over anhydrous $MgSO_4$ and the solvent was removed under reduced pressure. The residue was purified by column chromatography (and recrystallisation) to yield the corresponding sulfonamide.

2-Methyl-8-(methylthio)quinoline (1a)

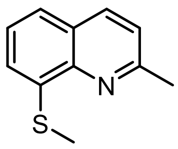

The title compound was synthesized from 2-(methylthio)aniline (2.5 mL, 20 mmol, 1.0 equiv.) and *trans*-crotonaldehyde (3.3 mL, 40 mmol, 2.0 equiv.) according to **GP2**. The product was obtained after column chromatography (*n*-pentane/Et_2O = 19:1, *v*:*v*) as pale yellow solid (1.25 g, 6.39 mmol, 33%).

^{1}H-NMR (400 MHz, $CDCl_3$) δ 8.00 (d, *J* = 8.4 Hz, 1H), 7.51 (dd, *J* = 8.0, 1.4 Hz, 1H), 7.42 (t, *J* = 7.7 Hz, 1H), 7.35 (dd, *J* = 7.3, 1.4 Hz, 1H), 7.31 (d, *J* = 8.4 Hz, 1H), 2.77 (s, 3H), 2.56 (s, 3H).

^{13}C{^{1}H}-NMR (101 MHz, $CDCl_3$) δ 158.3, 145.1, 139.2, 136.4, 126.2, 125.8, 123.4, 122.7, 122.7, 25.6, 14.3.

HRMS (ESI) m/z calc. for $C_{11}H_{11}NSNa$ $[M+Na]^+$ 212.0504, found 212.0502.

8-(Methylthio)-2-propylquinoline (1b)

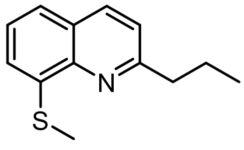

The title compound was synthesized from 2-(methylthio)aniline (0.62 mL, 5.0 mmol, 1.0 equiv.) and *trans*-2-hexenal (1.15 mL, 10.0 mmol, 2.0 equiv.) according to **GP2**. The product was obtained after column chromatography (*n*-pentane/Et_2O = 29:1, *v*:*v*) as yellow oil (164 mg, 0.755 mmol, 15%).

^{1}H-NMR (400 MHz, $CDCl_3$) δ 8.01 (d, *J* = 8.4 Hz, 1H), 7.51 (dd, *J* = 8.0, 1.4 Hz, 1H), 7.42 (t, *J* = 7.7 Hz, 1H), 7.35 (dd, *J* = 7.4, 1.4 Hz, 1H), 7.31 (d, *J* = 8.4 Hz, 1H), 2.98 (t, *J* = 7.6 Hz, 2H), 2.55 (s, 3H), 1.90 (h, *J* = 7.4 Hz, 2H), 1.04 (t, *J* = 7.4 Hz, 3H).

^{13}C{^{1}H}-NMR (101 MHz, $CDCl_3$) δ 161.9, 145.1, 139.5, 136.2, 126.4, 125.8, 123.3, 122.6, 122.1, 41.1, 22.9, 14.3, 14.2.

HRMS (ESI) m/z calc. for $C_{13}H_{15}NSNa$ $[M+Na]^+$ 240.0817, found 240.0816.

2-Isopropyl-8-(methylthio)quinoline (1c)

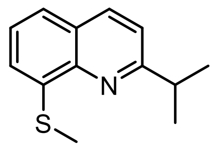

The title compound was synthesized from 2-(methylthio)aniline (0.62 mL, 5.0 mmol, 1.0 equiv.) and 4-methyl-2-pentenal (1.17 ml, 10.0 mmol, 2.0 equiv.) according to **GP2**. The product was obtained after column chromatography (*n*-pentane/Et_2O = 29:1, *v*:*v*) as yellow oil (175 mg, 0.805 mmol, 16%).

^{1}H-NMR (400 MHz, $CDCl_3$) δ 8.03 (d, J = 8.5 Hz, 1H), 7.51 (dd, J = 8.0, 1.4 Hz, 1H), 7.42 (t, J = 7.7 Hz, 1H), 7.38 – 7.33 (m, 2H), 3.29 (hept, J = 7.0 Hz, 1H), 2.55 (s, 3H), 1.42 (d, J = 6.9 Hz, 6H).

^{13}C{^{1}H}-NMR (101 MHz, $CDCl_3$) δ 166.5, 144.8, 139.8, 136.4, 126.6, 125.8, 123.2, 122.5, 120.3, 37.1, 22.7, 14.3.

HRMS (ESI) m/z calc. for $C_{13}H_{15}NSNa$ [M+Na]$^+$ 240.0817, found 240.0817.

2-Methyl-8-(phenylthio)quinoline (1d)

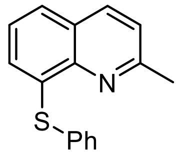

The title compound was synthesized from 2-(phenylthio)aniline (1.01 g, 5.00 mmol, 1.0 equiv.) and *trans*-crotonaldehyde (0.83 mL, 10 mmol, 2.0 equiv.) according to **GP2**. Purification by column chromatography on silica gel (*n*-pentane/EtOAc = 98:2, *v*:*v*), followed by recrystallisation from MeOH afforded the product as a yellow solid (317 mg, 1.26 mmol, 25%).

^{1}H-NMR (400 MHz, $CDCl_3$) δ 8.01 (d, J = 8.4 Hz, 1H), 7.73 – 7.63 (m, 2H), 7.53 – 7.40 (m, 4H), 7.34 (d, J = 8.4 Hz, 1H), 7.23 (t, J = 7.8 Hz, 1H), 6.93 (dd, J = 7.5, 1.3 Hz, 1H), 2.81 (s, 3H).

^{13}C{^{1}H}-NMR (101 MHz, $CDCl_3$) δ 158.6, 144.4, 139.6, 136.4, 136.1, 132.1, 129.8, 129.1, 126.4, 125.9, 124.9, 124.1, 122.9, 25.6.

HRMS (ESI) m/z calc. for $C_{16}H_{13}NSNa$ [M+Na]$^+$ 274.0661, found 274.0660.

3-Methyl-8-(methylthio)quinoline (1e)

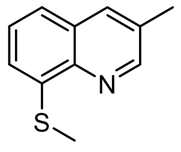

The title compound was synthesized from 2-(methylthio)aniline (1.25 mL, 10.0 mmol, 1.0 equiv.) and methacrylaldehyde (1.66 mL, 20.0 mmol, 2.0 equiv.) according to **GP2**. Purification by column chromatography on silica gel (*n*-pentane/EtOAc = 95:5, *v*:*v*), followed by recrystallisation from MeOH afforded the product as a white solid (89 mg, 0.47 mmol, 5%).

^{1}H-NMR (400 MHz, $CDCl_3$) δ 8.77 (d, J = 2.2 Hz, 1H), 7.88 – 7.86 (m, 1H), 7.49 – 7.42 (m, 2H), 7.31 (dd, J = 6.8, 1.9 Hz, 1H), 2.56 (s, 3H), 2.50 (s, 3H).

^{13}C{^{1}H}-NMR (101 MHz, $CDCl_3$) δ 151.1, 143.8, 139.7, 135.1, 131.3, 128.0, 126.8, 123.1, 122.0, 18.8, 14.3.

HRMS (ESI) m/z calc. for $C_{11}H_{11}NSNa$ [M+Na]$^+$ 212.0504, found 212.0504.

2-Methyl-6-(methylthio)quinoline (1f)

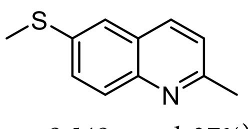

The title compound was synthesized from 6-bromo-2-methylquinoline (444 mg, 2.00 mmol, 1.0 equiv.) according to **GP3**. Purification by column chromatography on silica gel (*n*-pentane/EtOAc = 4:1, *v*:*v*) afforded the product as a light brown oil (104 mg, 0.549 mmol, 27%).

^{1}H-NMR (400 MHz, $CDCl_3$) δ 7.95 (d, *J* = 8.6 Hz, 2H), 7.56 (dd, *J* = 8.9, 2.1 Hz, 1H), 7.50 (d, *J* = 2.2 Hz, 1H), 7.29 – 7.23 (m, 1H), 2.72 (s, 3H), 2.58 (s, 3H).

^{13}C{^{1}H}-NMR (101 MHz, $CDCl_3$) δ 158.3, 145.8, 136.5, 135.4, 129.3, 128.6, 127.2, 122.8, 122.8, 25.0, 15.9.

HRMS (ESI) m/z calc. for $C_{11}H_{12}NS$ $[M+H]^+$ 190.0685, found 190.0678.

2-Ethyl-3-methyl-8-(methylthio)quinoline (1g)

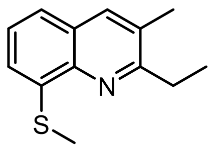

The title compound was synthesized from 2-(methylthio)aniline (1.24 mL, 10.0 mmol, 1.0 equiv.) and 2-methyl-2-pentenal (2.28 mL, 20.0 mmol, 2.0 equiv.) according to **GP2**. The product was obtained after column chromatography (*n*-pentane/EtOAc = 49:1, *v*:*v*) as yellow solid (318 mg, 1.46 mmol, 15%).

^{1}H-NMR (400 MHz, $CDCl_3$) δ 7.78 (d, *J* = 1.1 Hz, 1H), 7.45 (dd, *J* = 8.1, 1.4 Hz, 1H), 7.39 (dd, *J* = 8.1, 7.2 Hz, 1H), 7.29 (dd, *J* = 7.3, 1.4 Hz, 1H), 2.99 (q, *J* = 7.4 Hz, 2H), 2.55 (s, 3H), 2.46 (d, *J* = 1.0 Hz, 3H), 1.45 (t, *J* = 7.4 Hz, 3H).

^{13}C{^{1}H}-NMR (101 MHz, $CDCl_3$) δ 161.9, 143.7, 139.3, 135.6, 130.3, 126.9, 125.8, 122.6, 121.7, 29.2, 19.1, 14.3, 12.1.

HRMS (ESI) m/z calc. for $C_{13}H_{16}NS$ $[M+H]^+$ 218.0998, found 218.0995.

(*S*)-5-(Methylthio)-3-(prop-1-en-2-yl)-1,2,3,4-tetrahydroacridine (1h)

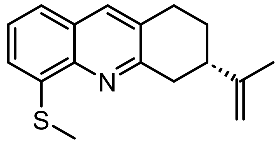

The title compound was synthesized from 2-(methylthio)aniline (0.62 mL, 5.0 mmol, 1.0 equiv.) and perillaldehyde (1.55 mL, 10.0 mmol, 2.0 equiv.) according to **GP2**. The product was obtained after column chromatography (*n*-pentane/Et_2O = 19:1, *v*:*v*) as yellow solid (116 mg, 0.431 mmol, 9%).

^{1}H-NMR (400 MHz, $CDCl_3$) δ 7.79 (s, 1H), 7.45 (dd, *J* = 8.1, 1.4 Hz, 1H), 7.38 (dd, *J* = 8.1, 7.3 Hz, 1H), 7.28 (dd, *J* = 7.3, 1.4 Hz, 1H), 4.85 – 4.79 (m, 2H), 3.39 (ddd, *J* = 17.4, 4.8, 1.9 Hz, 1H), 3.16 – 2.93 (m, 3H), 2.58 – 2.49 (m, 4H), 2.15 – 2.05 (m, 1H), 1.85 (s, 3H), 1.79 – 1.70 (m, 1H).

^{13}C{^{1}H}-NMR (101 MHz, $CDCl_3$) δ 158.3, 148.6, 143.9, 138.8, 135.0, 130.8, 126.9, 125.8, 122.9, 121.8, 109.7, 41.8, 39.1, 28.8, 27.9, 21.1, 14.3.

HRMS (ESI) m/z calc. for $C_{17}H_{19}NSNa$ $[M+Na]^+$ 292.1130, found 292.1129.

3-Methyl-8-(methylthio)-2-phenylquinoline (1i)

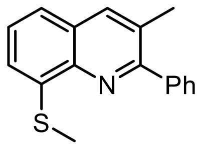

The title compound was synthesized from 2-(methylthio)aniline (0.62 mL, 5.0 mmol, 1.0 equiv.) and (*E*)-2-methyl-3-phenylacrylaldehyde (1.39 mL, 10.0 mmol, 2.0 equiv.) according to **GP2**. The product was obtained after column chromatography (*n*-pentane/Et_2O = 29:1, *v*:*v*) as yellow solid (394 mg, 1.48 mmol, 30%).

^{1}H-NMR (400 MHz, $CDCl_3$) δ 7.98 (d, *J* = 1.0 Hz, 1H), 7.73 – 7.69 (m, 2H), 7.57 – 7.38 (m, 5H), 7.33 (dd, *J* = 7.2, 1.4 Hz, 1H), 2.55 (s, 3H), 2.53 (d, *J* = 1.0 Hz, 3H).

^{13}C{^{1}H}-NMR (101 MHz, $CDCl_3$) δ 158.8, 143.9, 140.7, 140.1, 137.2, 129.9, 129.7, 128.3, 128.2, 127.3, 126.6, 122.5, 122.1, 21.0, 14.3.

HRMS (ESI) m/z calc. for $C_{17}H_{15}NSNa$ $[M+Na]^+$ 288.0817, found 288.0815.

8-(Methylthio)-2-phenylquinoline (1j)

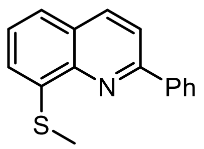

The title compound was synthesized from 2-(methylthio)aniline (0.62 mL, 5.0 mmol, 1.0 equiv.) and cinnamaldehyde (1.26 mmol, 10.0 mmol, 2.0 equiv.) according to **GP2**. The product was obtained after column chromatography (*n*-pentane/Et_2O = 19:1, *v*:*v*) as yellow sticky gum (143 mg, 569 mmol, 11%).

^{1}H-NMR (400 MHz, $CDCl_3$) δ 8.32 – 8.25 (m, 2H), 8.18 (d, *J* = 8.6 Hz, 1H), 7.95 (d, *J* = 8.6 Hz, 1H), 7.57 – 7.50 (m, 3H), 7.49 – 7.43 (m, 2H), 7.39 (dd, *J* = 7.4, 1.3 Hz, 1H), 2.59 (s, 3H).

^{13}C{^{1}H}-NMR (101 MHz, $CDCl_3$) δ 155.6, 145.3, 140.7, 139.2, 137.1, 129.6, 128.9, 127.7, 127.0, 126.5, 123.1, 122.9, 119.1, 14.3.

HRMS (ESI) m/z calc. for $C_{16}H_{13}NSNa$ $[M+Na]^+$ 274.0661, found 274.0659.

2-(4-Fluorophenyl)-8-(methylthio)quinoline (1k)

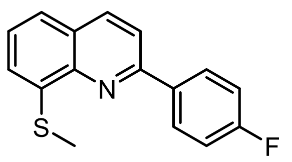

The title compound was synthesized from 2-(methylthio)aniline (0.62 mL, 5.0 mmol, 1.0 equiv.) and *trans*-4-fluorocinnamaldehyde (1.31 mL, 10.0 mmol, 2.0 equiv.) according to **GP2**. The product was obtained after column chromatography (*n*-pentane/Et_2O = 19:1, *v*:*v*) as yellow solid (132 mg, 0.490 mmol, 10%).

^{1}H-NMR (400 MHz, $CDCl_3$) δ 8.32 – 8.22 (m, 2H), 8.17 (d, *J* = 8.6 Hz, 1H), 7.89 (d, *J* = 8.6 Hz, 1H), 7.55 (dd, *J* = 8.0, 1.4 Hz, 1H), 7.47 (dd, *J* = 8.1, 7.3 Hz, 1H), 7.39 (dd, *J* = 7.4, 1.4 Hz, 1H), 7.24 – 7.16 (m, 2H), 2.59 (s, 3H).

^{13}C{^{1}H}-NMR (101 MHz, $CDCl_3$) δ 165.3, 162.8, 154.5, 145.2, 140.6, 137.2, 135.4 (d, *J* = 3.2 Hz), 129.5 (d, *J* = 8.5 Hz), 126.9, 126.6, 123.1 (d, *J* = 9.5 Hz), 118.7, 115.9 (d, *J* = 21.7 Hz), 14.3.

^{19}F{^{1}H}-NMR (376 MHz, $CDCl_3$) δ -112.3.

HRMS (ESI) m/z calc. for $C_{16}H_{12}NSFNa$ $[M+Na]^+$ 292.0567, found 292.0566.

2-(4-Chlorophenyl)-8-(methylthio)quinoline (1l)

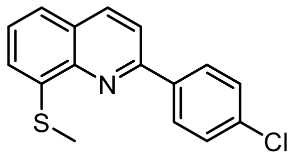

The title compound was synthesized from 2-(methylthio)aniline (0.62 mL, 5.0 mmol, 1.0 equiv.) and *trans*-4-chlorocinnamaldehyde (1.67 g, 10.0 mmol, 2.0 equiv.) according to **GP2**. The product was obtained after column chromatography (*n*-pentane/Et_2O = 19:1, *v*:*v*) as yellow solid (378 mg, 1.32 mmol, 26%).

^{1}H-NMR (400 MHz, $CDCl_3$) δ 8.25 – 8.20 (m, 2H), 8.18 (d, *J* = 8.6 Hz, 1H), 7.90 (d, *J* = 8.6 Hz, 1H), 7.55 (dd, *J* = 8.1, 1.4 Hz, 1H), 7.51 – 7.45 (m, 3H), 7.39 (dd, *J* = 7.4, 1.3 Hz, 1H), 2.59 (s, 3H).

^{13}C{^{1}H}-NMR (101 MHz, $CDCl_3$) δ 154.3, 145.2, 140.7, 137.7, 137.3, 135.8, 129.1, 128.9, 127.1, 126.8, 123.1, 123.1, 118.7, 14.3.

HRMS (ESI) m/z calc. for $C_{16}H_{13}NSCl$ $[M+H]^+$ 286.0452, found 286.0450.

2-(4-Bromophenyl)-8-(methylthio)quinoline (1m)

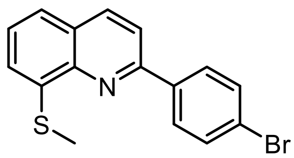

The title compound was synthesized from 2-(methylthio)aniline (0.62 mL, 5.0 mmol, 1.0 equiv.) and *trans*-4-bromocinnamaldehyde (1.48 g, 7.00 mmol, 1.4 equiv.) according to **GP2**. The product was obtained after column chromatography (*n*-pentane/Et_2O = 29:1, *v*:*v*) as yellow solid (306 mg, 0.927 mmol, 19%).

^{1}H-NMR (400 MHz, $CDCl_3$) δ 8.21 – 8.11 (m, 3H), 7.90 (d, *J* = 8.6 Hz, 1H), 7.67 – 7.62 (m, 2H), 7.55 (dd, *J* = 8.1, 1.4 Hz, 1H), 7.48 (t, *J* = 7.7 Hz, 1H), 7.39 (dd, *J* = 7.4, 1.3 Hz, 1H), 2.59 (s, 3H).

^{13}C{^{1}H}-NMR (101 MHz, $CDCl_3$) δ 154.4, 145.2, 140.7, 138.1, 137.3, 132.1, 129.2, 127.1, 126.8, 124.2, 123.1, 123.1, 118.6, 14.3.

HRMS (ESI) m/z calc. for $C_{16}H_{13}NSBr$ $[M+H]^+$ 331.9927, found 331.9926.

8-(Methylthio)-2-(thiophen-2-yl)quinoline (1n)

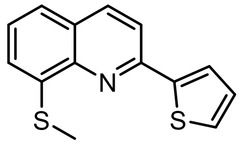

The title compound was synthesized from 2-(methylthio)aniline (0.62 mL, 5.0 mmol, 1.0 equiv.) and (*E*)-3-(thiophen-2-yl)acrylaldehyde (691 mg, 5.00 mmol, 1.0 equiv.) according to **GP2**. The product was obtained after column chromatography (*n*-pentane/Et_2O = 19:1, *v*:*v*) as yellow sticky gum (91 mg, 0.35 mmol, 7%).

^{1}H-NMR (400 MHz, $CDCl_3$) δ 8.09 (d, *J* = 8.6 Hz, 1H), 7.80 (d, *J* = 8.6 Hz, 1H), 7.73 (dd, *J* = 3.7, 1.1 Hz, 1H), 7.50 (dd, *J* = 7.9, 1.5 Hz, 1H), 7.46 (dd, *J* = 5.0, 1.1 Hz, 1H), 7.42 (t, *J* = 7.6 Hz, 1H), 7.37 (dd, *J* = 7.4, 1.5 Hz, 1H), 7.14 (dd, *J* = 5.0, 3.7 Hz, 1H), 2.58 (s, 3H).

^{13}C{^{1}H}-NMR (101 MHz, $CDCl_3$) δ 151.1, 145.7, 145.2, 140.0, 137.0, 129.0, 128.2, 127.0, 126.3, 125.9, 123.4, 123.3, 118.0, 14.4.

HRMS (ESI) m/z calc. for $C_{14}H_{11}NS_2Na$ $[M+Na]^+$ 280.0225, found 280.0225.

2-(Furan-2-yl)-8-(methylthio)quinoline (1o)

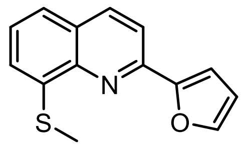

The title compound was synthesized from 2-(methylthio)aniline (0.62 mL, 5.0 mmol, 1.0 equiv.) and *trans*-3-(2-furyl)acrolein (1.22 g, 10.0 mmol, 2.0 equiv.) according to **GP2**. The product was obtained after column chromatography (*n*-pentane/Et_2O = 29:1, *v*:*v*) as yellow sticky gum (63 mg, 0.26 mmol, 5%).

^{1}H-NMR (400 MHz, $CDCl_3$) δ 8.13 (d, *J* = 8.6 Hz, 1H), 7.87 (d, *J* = 8.6 Hz, 1H), 7.60 (dd, *J* = 1.8, 0.8 Hz, 1H), 7.51 (dd, *J* = 7.9, 1.5 Hz, 1H), 7.43 (t, *J* = 7.6 Hz, 1H), 7.37 (dd, *J* = 7.4, 1.5 Hz, 1H), 7.32 (dd, *J* = 3.4, 0.8 Hz, 1H), 6.58 (dd, *J* = 3.4, 1.7 Hz, 1H), 2.58 (s, 3H).

GC-MS (EI) m/z calc. for $[C_{14}H_{11}NOS]^+$ 241.0561, found: 242.10 (17), 241.00 (92), 240.00 (31), 209.00 (18), 208.10 (100), 207.00 (12), 196.00 (17), 195.00 (38), 178.00 (11), 167.00 (15), 166.00 (20), 140.00 (13), 139.00 (15).

8-Fluoro-6-(methylthio)quinoline (1p)

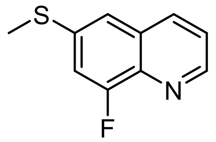

The title compound was synthesized from 6-bromo-8-fluoroquinoline (452 mg, 2.00 mmol, 1.0 equiv.) according to **GP3**. Purification by column chromatography on silica gel (*n*-pentane/EtOAc = 4:1, *v*:*v*) afforded the product as a yellow solid (124 mg, 0.642 mmol, 32%).

^{1}H-NMR (400 MHz, $CDCl_3$) δ 8.87 (dd, *J* = 4.2, 1.6 Hz, 1H), 8.05 (dt, *J* = 8.4, 1.6 Hz, 1H), 7.43 (dd, *J* = 8.4, 4.2 Hz, 1H), 7.35 – 7.29 (m, 2H), 2.59 (s, 3H).

^{13}C{^{1}H}-NMR (101 MHz, $CDCl_3$) δ 157.8 (d, *J* = 259.1 Hz), 149.6 (d, *J* = 1.5 Hz), 138.1 (d, *J* = 8.0 Hz), 136.9 (d, *J* = 12.3 Hz), 134.6 (d, *J* = 3.1 Hz), 130.3 (d, *J* = 2.9 Hz), 122.8 (d, *J* = 1.1 Hz), 118.1 (d, *J* = 4.1 Hz), 113.1 (d, *J* = 21.2 Hz), 15.8.

^{19}F-NMR (376 MHz, $CDCl_3$) δ -125.30.

HRMS (ESI+) m/z calc. for $C_{10}H_8FNSNa$ $[M+Na]^+$ 216.0254, found 216.0251.

1-(8-Bromoquinolin-2-yl)ethan-1-one

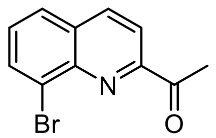

According to the literature,[7] in an oven-dried Schlenk tube, $Fe(ClO_4)_3{\cdot}H_2O$ (372 mg, 1.00 mmol, 10 mol%) and 8-bromoquinoline (2.08 g, 10.0 mmol, 1.0 equiv.) were suspended in triethyl orthoformate (20 mL). To the stirring mixture, *t*BuOOH (70% in H_2O, 5.5 mL, 40 mmol, 4.0 equiv.) was added slowly over 2 min. The mixture was then stirred at 100 °C for 12 h. The reaction was cooled to r.t. and the mixture was extracted with EtOAc. The combined organic layers were washed with H_2O and brine, dried over anhydrous $MgSO_4$ and solvents were removed under reduced pressure. The residue was purified by column chromatography on silica gel (*n*-pentane/EtOAc = 9:1, *v*:*v*) to afford the product as a yellow solid (689 mg, 2.76 mmol, 28%).

^{1}H-NMR (400 MHz, $CDCl_3$) δ 8.28 (d, *J* = 8.5 Hz, 1H), 8.18 (d, *J* = 8.5 Hz, 1H), 8.12 (dd, *J* = 7.5, 1.3 Hz, 1H), 7.84 (dd, *J* = 8.2, 1.3 Hz, 1H), 7.50 (dd, *J* = 8.2, 7.4 Hz, 1H), 2.94 (s, 3H).

^{13}C{^{1}H}-NMR (101 MHz, $CDCl_3$) δ 200.5, 153.6, 144.5, 137.7, 133.9, 131.0, 129.1, 127.6, 126.5, 118.8, 25.6.

HRMS (ESI+) m/z calc. for $C_{11}H_8BrNONa$ $[M+Na]^+$ 271.9682, found 271.9681.

1-(8-(Isopropylthio)quinolin-2-yl)ethan-1-one (1q)

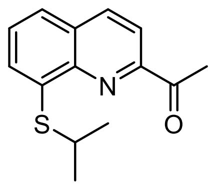

According to the literature,[8] an oven-dried Schlenk tube was charged with Ni(COD)$_2$ (20.6 mg, 0.0750 mmol, 5 mol%), Xantphos (43.4 mg, 0.0750 mmol, 5 mol%) and CsOAc (432 mg, 2.25 mmol, 1.5 equiv.) under argon. 1-(8-bromoquinolin-2-yl)ethan-1-one (375 mg, 1.50 mmol, 1.0 equiv.) and propane-2-thiol (0.211 mL, 1.65 mmol, 1.1 equiv.) were added and the mixture was dissolved in THF (5 mL). The solution was stirred at r.t. for 24 h, after which the mixture was quenched with brine and diluted with EtOAc. After phase separation, the organic layer was washed with brine and the combined aqueous phases were extracted with EtOAc. The combined organic phases were dried over anhydrous $MgSO_4$ and solvents were removed under reduced pressure. The residue was purified by column chromatography on silica gel (*n*-pentane/EtOAc = 98:2, *v*:*v*) to afford the product as a colorless oil (115 mg, 0.469 mmol, 31%).

^{1}H-NMR (400 MHz, $CDCl_3$) δ 8.92 (dd, *J* = 8.8, 0.9 Hz, 1H), 8.16 (d, *J* = 8.8 Hz, 1H), 8.13 (dt, *J* = 8.4, 1.1 Hz, 1H), 7.82 (dd, *J* = 7.2, 1.2 Hz, 1H), 7.72 (dd, *J* = 8.4, 7.2 Hz, 1H), 3.39 (hept, *J* = 6.7 Hz, 1H), 2.87 (s, 3H), 1.30 (d, *J* = 6.7 Hz, 6H).

^{13}C{^{1}H}-NMR (101 MHz, $CDCl_3$) δ 200.7, 153.4, 147.9, 135.4, 134.0, 133.6, 131.2, 130.6, 129.6, 118.3, 39.7, 25.7, 23.4.

HRMS (ESI) m/z calc. for $C_{14}H_{15}NOSNa$ [M+Na]$^+$ 268.0767, found 268.0757.

7-Chloro-4-methoxyquinoline

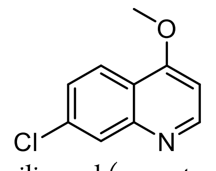

A Schlenk tube was charged with 4,7-dichloroquinoline (1.98 g, 10.0 mmol, 1.0 equiv.), NaOMe (810 mg, 15.0 mmol, 1.5 equiv.) and MeOH (15 mL). The mixture was stirred at 50 °C for 18 h. Afterwards, the mixture was filtered over Celite® and the solvent was removed under reduced pressure. The residue was purified by column chromatography on silica gel (*n*-pentane/EtOAc = 1:1, *v*:*v*) to afford the product as a white solid (1.74 g, 8.99 mmol, 90%).

^{1}H-NMR (400 MHz, $CDCl_3$) δ 8.74 (d, *J* = 5.3 Hz, 1H), 8.12 (d, *J* = 8.9 Hz, 1H), 8.02 (d, *J* = 2.1 Hz, 1H), 7.44 (dd, *J* = 8.9, 2.1 Hz, 1H), 6.72 (d, *J* = 5.2 Hz, 1H), 4.03 (s, 3H).

^{13}C{^{1}H}-NMR (101 MHz, $CDCl_3$) δ 162.5, 152.7, 149.8, 135.9, 128.0, 126.7, 123.5, 120.0, 100.5, 55.9.

HRMS (ESI) m/z calc. for $C_{10}H_9ClNO$ [M+H]$^+$ 194,0367, found 194.0366.

7-(Isopropylthio)-4-methoxyquinoline (1r)

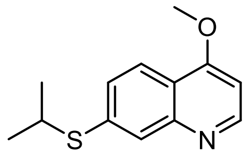

According to the literature,[8] an oven-dried Schlenk tube was charged with Ni(COD)$_2$ (24.1 mg, 0.0875 mmol, 5 mol%), Xantphos (50.6 mg, 0.0875 mmol, 5 mol%) and KOAc (258 mg, 2.63 mmol, 1.5 equiv.) under argon. 7-chloro-4-methoxyquinoline (339 mg, 1.75 mmol, 1.0 equiv.) and propane-2-thiol (0.18 mL, 1.9 mmol, 1.1 equiv.) were added and the mixture was dissolved in THF (5 mL). The solution was stirred at r.t. for 18 h, after which the mixture was quenched with brine and diluted with EtOAc. After phase separation, the organic layer was washed with brine and the combined aqueous phases were extracted with EtOAc. The combined organic phases were dried over anhydrous $MgSO_4$ and solvents were removed under reduced pressure. The residue was purified by column chromatography on silica gel (*n*-pentane/EtOAc = 3:7, *v*:*v*) to afford the product as a colorless oil (147 mg, 0.630 mmol, 36%).

^{1}**H-NMR** (400 MHz, $CDCl_3$) δ 8.70 (d, *J* = 5.3 Hz, 1H), 8.06 (d, *J* = 8.7 Hz, 1H), 7.92 (d, *J* = 1.8 Hz, 1H), 7.40 (dd, *J* = 8.7, 1.8 Hz, 1H), 6.67 (d, *J* = 5.3 Hz, 1H), 4.01 (s, 3H), 3.62 (hept, *J* = 6.7 Hz, 1H), 1.38 (d, *J* = 6.7 Hz, 6H).

^{13}**C{^{1}H}-NMR** (101 MHz, $CDCl_3$) δ 162.4, 151.7, 149.3, 139.4, 127.6, 127.4, 122.0, 119.3, 99.9, 55.7, 37.0, 23.0.

HRMS (ESI) m/z calc. for $C_{13}H_{16}NOS$ $[M+H]^+$ 234.0947, found 234.0946.

3-(1,3-Dithian-2-yl)quinoline (1s)

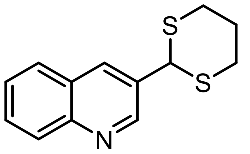

$BF_3{\cdot}OEt_2$ (1.27 mL, 10.0 mmol, 2.0 equiv.) was added under argon to a solution of 3-quinolinecarboxaldehyde (786 mg, 5.00 mmol, 1.0 equiv.) and 1,3-propanedithiol (0.551 mL, 5.50 mmol, 1.1 equiv.) in dry CH_2Cl_2 (20 mL) and the reaction was stirred at r.t. for 18 h. The reaction was quenched with aq. sat. $NaHCO_3$, phases were separated and the organic layer was washed with brine. The organics were dried over anhydrous $MgSO_4$ and concentrated *in vacuo*. The crude was purified by column chromatography (*n*-pentane/EtOAc = 3:2, *v*:*v*) to afford the product as white solid (972 mg, 3.93 mmol, 79%).

^{1}**H-NMR** (400 MHz, $CDCl_3$) δ 8.97 (d, *J* = 2.3 Hz, 1H), 8.28 (d, *J* = 2.3 Hz, 1H), 8.13 – 8.06 (m, 1H), 7.82 (dd, *J* = 8.2, 1.4 Hz, 1H), 7.71 (ddd, *J* = 8.5, 6.9, 1.5 Hz, 1H), 7.55 (ddd, *J* = 8.1, 6.9, 1.2 Hz, 1H), 5.36 (s, 1H), 3.18 – 3.06 (m, 2H), 3.02 – 2.92 (m, 2H), 2.28 – 2.17 (m, 1H), 2.07 – 1.91 (m, 1H).

^{13}**C{^{1}H}-NMR** (101 MHz, $CDCl_3$) δ 150.3, 148.1, 135.0, 132.3, 130.0, 129.4, 128.1, 127.9, 127.1, 48.6, 32.1, 25.0.

HRMS (ESI) m/z calc. for $C_{13}H_{13}NS_2Na$ $[M+Na]^+$ 270.0382, found 270.0381.

2-Methyl-6-((trifluoromethyl)thio)quinoline (1t)

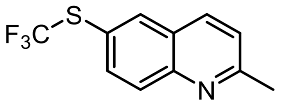

The title compound was synthesized from 4-(trifluoromethylthio)aniline (0.72 mL, 5.0 mmol, 1.0 equiv.) and *trans*-crotonaldehyde (0.825 mL, 10.0 mmol, 2.0 equiv.) according to **GP2**. The product was obtained after column chromatography (*n*-pentane/EtOAc = 9:1, *v*:*v*) as brown solid (127 mg, 0.522 mmol, 10%).

^{1}**H-NMR** (400 MHz, $CDCl_3$) δ 8.13 (d, *J* = 2.1 Hz, 1H), 8.06 (t, *J* = 8.4 Hz, 2H), 7.86 (dd, *J* = 8.7, 2.1 Hz, 1H), 7.37 (d, *J* = 8.5 Hz, 1H), 2.77 (s, 3H).

^{13}**C{^{1}H}-NMR** (101 MHz, $CDCl_3$) δ 161.5, 148.5, 136.7, 136.4, 135.7, 130.2, 129.7 (q, *J* = 308.3 Hz), 126.8, 123.2, 121.7 (q, *J* = 2.2 Hz), 25.7.

^{19}**F{^{1}H}-NMR** (376 MHz, $CDCl_3$) δ -42.5.

HRMS (ESI) m/z calc. for $C_{11}H_9NSF_3$ $[M+H]^+$ 244.0402, found 244.0401.

2-Methyl-8-(methylsulfinyl)quinoline (1u)

According to the literature,[9] a Schlenk tube was charged with 2-methyl-8- (methylthio)quinoline (378 mg, 2.00 mmol, 1.0 equiv.) and EtOH (10 mL). H_2O_2 (30% in H_2O, 0.40 mL, 4.0 mmol, 2.0 equiv.) and Tf_2O (0.17 mL, 1.0 mmol, 50 mol%) were added and the mixture was stirred at r.t. for 1 h. The reaction was quenched

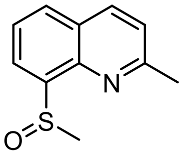

by adding H_2O and the mixture was extracted with EtOAc. The combined organic layers were dried over anhydrous $MgSO_4$ and solvents were removed under reduced pressure. The residue was purified by column chromatography on silica gel (CH_2Cl_2/MeOH = 98:2, *v*:*v*) to afford the product as a brown oil (224 mg, 1.09 mmol, 55%).

^{1}H-NMR (400 MHz, $CDCl_3$) δ 8.24 (dd, *J* = 7.2, 1.4 Hz, 1H), 8.10 (d, *J* = 8.5 Hz, 1H), 7.88 (dd, *J* = 8.1, 1.5 Hz, 1H), 7.67 (dd, *J* = 8.1, 7.3 Hz, 1H), 7.36 (d, *J* = 8.5 Hz, 1H), 3.02 (s, 3H), 2.72 (s, 3H).

^{13}C{^{1}H}-NMR (101 MHz, $CDCl_3$) δ 159.4, 143.7, 142.8, 136.3, 130.0, 126.4, 125.9, 125.7, 123.0, 42.4, 25.5.

HRMS (ESI) m/z calc. for $C_{11}H_{11}NOSNa$ $[M+Na]^+$ 228.0454, found 228.0446.

2-Methyl-8-(methylsulfonyl)quinoline (1v)

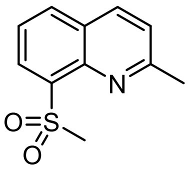

According to the literature,[10] a Schlenk tube was charged with 2-methyl-8-(methylthio)quinoline (235 mg, 1.24 mmol, 1.0 equiv.) and CH_2Cl_2 (1.24 mL). The solution was cooled to 0 °C and *m*CPBA (639 mg, 2.60 mmol, 2.1 equiv.) was added. The mixture was stirred at r.t. for 3 h, after which it was treated with aq. sat. $NaHCO_3$ and extracted with CH_2Cl_2. The combined organic layers were dried over anhydrous $MgSO_4$ and solvents were removed under reduced pressure. The residue was purified by column chromatography on silica gel (CH_2Cl_2/acetone = 98:2, *v*:*v*) to afford the product as a beige solid (209 mg, 0.945 mmol, 76%).

^{1}H-NMR (400 MHz, $CDCl_3$) δ 8.49 (dd, *J* = 7.4, 1.5 Hz, 1H), 8.13 (d, *J* = 8.5 Hz, 1H), 8.04 (dd, *J* = 8.1, 1.5 Hz, 1H), 7.60 (dd, *J* = 8.1, 7.4 Hz, 1H), 7.42 (d, *J* = 8.5 Hz, 1H), 3.65 (s, 3H), 2.82 (s, 3H).

^{13}C{^{1}H}-NMR (101 MHz, $CDCl_3$) δ 161.0, 143.9, 137.0, 136.5, 134.1, 130.9, 127.3, 124.8, 123.2, 44.6, 26.0.

HRMS (ESI) m/z calc. for $C_{11}H_{11}NO_2SNa$ $[M+Na]^+$ 244.0403, found 244.0401.

Quinoline-8-sulfonyl fluoride (1w)

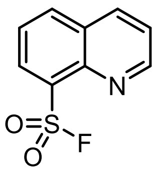

Prepared according to a literature procedure by Grimster and co-workers.[11] A solution of KHF_2 (1.72 g, 22.0 mmol, 11.0 equiv.) in H_2O (11 mL) was added to a solution of quinoline-8-sulfonyl chloride (455 mg, 2.00 mmol, 1.0 equiv.) in MeCN (11 mL). The reaction mixture was stirred vigorously for 16 h at r.t. Phases were separated, the aqueous layer was extracted with CH_2Cl_2 (3x), combined organics dried over $MgSO_4$ and concentrated *in vacuo*. The crude was purified by column chromatography (*n*-pentane/EtOAc = 2:1, *v*:*v*) to afford the product as white solid (103 mg, 0.488 mmol, 24%).

^{1}H-NMR (400 MHz, $CDCl_3$) δ 9.19 (dd, *J* = 4.3, 1.8 Hz, 1H), 8.52 (dd, *J* = 7.5, 1.4 Hz, 1H), 8.32 (dd, *J* = 8.3, 1.8 Hz, 1H), 8.23 (dd, *J* = 8.3, 1.5 Hz, 1H), 7.72 (ddd, *J* = 8.5, 7.4, 1.4 Hz, 1H), 7.63 (dd, *J* = 8.4, 4.2 Hz, 1H).

^{13}C{^{1}H}-NMR (101 MHz, $CDCl_3$) δ 152.9, 144.0 (d, *J* = 1.5 Hz), 136.7, 136.3, 133.2 (d, *J* = 2.1 Hz), 131.6 (d, *J* = 21.1 Hz), 129.2 (d, *J* = 1.2 Hz), 125.4 (d, *J* = 0.8 Hz), 123.2.

^{19}F{^{1}H}-NMR (376 MHz, $CDCl_3$) δ 60.2.

HRMS (ESI) m/z calc. for $C_9H_6NO_2SFNa$ $[M+Na]^+$ 233.9996, found 233.9995.

7-(Methylthio)quinoline

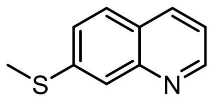

The title compound was synthesized from 7-bromoquinoline (2.08 g, 10.0 mmol, 1.0 equiv.) according to **GP3**. The product was obtained after column chromatography (*n*-pentane/EtOAc = 2:1, *v*:*v*) as yellow oil (683 mg, 3.90 mmol, 39%).

^{1}H-NMR (400 MHz, $CDCl_3$) δ 8.84 (dd, *J* = 4.3, 1.8 Hz, 1H), 8.07 (ddd, *J* = 8.3, 1.8, 0.8 Hz, 1H), 7.80 (d, *J* = 1.9 Hz, 1H), 7.67 (d, *J* = 8.6 Hz, 1H), 7.39 (dd, *J* = 8.6, 1.9 Hz, 1H), 7.31 (dd, *J* = 8.2, 4.3 Hz, 1H), 2.60 (s, 3H).

^{13}C{^{1}H}-NMR (101 MHz, $CDCl_3$) δ 150.8, 148.9, 141.6, 136.0, 127.8, 126.1, 126.0, 123.2, 120.4, 15.2.

HRMS (ESI) m/z calc. for $C_{10}H_{10}NS$ [M+H]$^+$ 176.0529, found 176.0527.

Imino(methyl)(quinolin-7-yl)-λ^6-sulfanone

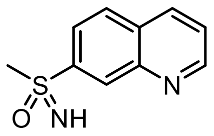

Prepared according to a procedure by Reboul and co-workers.[12] 7-(Methylthio)quinoline (701 mg, 4.00 mmol, 1.0 equiv.) and $PhI(OAc)_2$ (3.22 g, 10.0 mmol, 2.5 equiv.) were dissolved in MeOH (10 mL). A solution of ammonia in MeOH (7 M, 3.43 mL, 24.0 mmol, 6.0 equiv.) was added and the reaction was stirred at r.t. for 3 h. The solvent was removed *in vacuo* and the crude was purified by column chromatography (CH_2Cl_2/MeOH = 97:3, *v*:*v*) to afford the product as off-white solid (723 mg, 3.51 mmol, 88%).

^{1}H-NMR (400 MHz, $CDCl_3$) δ 9.06 (dd, *J* = 4.2, 1.7 Hz, 1H), 8.80 (dt, *J* = 1.8, 0.7 Hz, 1H), 8.26 (ddd, *J* = 8.3, 1.8, 0.8 Hz, 1H), 8.10 (dd, *J* = 8.6, 1.9 Hz, 1H), 8.00 (d, *J* = 8.6 Hz, 1H), 7.57 (dd, *J* = 8.3, 4.2 Hz, 1H), 3.20 (s, 3H), 2.86 (s, 1H).

^{13}C{^{1}H}-NMR (101 MHz, $CDCl_3$) δ 152.4, 147.6, 144.5, 136.1, 130.5, 130.4, 129.7, 123.9, 123.6, 46.1.

HRMS (ESI) m/z calc. for $C_{10}H_{10}N_2OSNa$ [M+Na]$^+$ 229.0406, found 229.0401.

2,2,2-Trifluoro-*N*-(methyl(oxo)(quinolin-7-yl)- λ^6-sulfaneylidene)acetamide (1x)

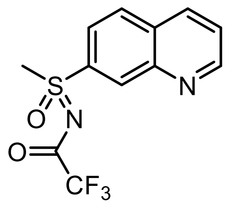

Prepared according to a literature procedure by Bolm and co-workers.[13] Under argon a solution of imino(methyl)(quinolin-7-yl)-λ^6-sulfanone (413 mg, 2.00 mmol, 1.0 equiv.) and dry NEt_3 (0.416 mL, 3.00 mmol, 1.5 equiv.) in dry CH_2Cl_2 (10 mL) was cooled to 0 °C and TFAA (0.334 mL, 2.40 mmol, 1.2 equiv.) was added slowly. The reaction was then stirred at r.t. for 16 h. The solvent was removed *in vacuo* and the crude was purified by column chromatography (*n*-pentane/EtOAc = 1:1, *v*:*v*) to afford the product as white solid (536 mg, 1.77 mmol, 89%).

^{1}H-NMR (400 MHz, $CDCl_3$) δ 9.10 (dd, *J* = 4.2, 1.7 Hz, 1H), 8.82 (d, *J* = 2.0 Hz, 1H), 8.29 (dt, *J* = 8.3, 1.2 Hz, 1H), 8.10 (d, *J* = 8.7 Hz, 1H), 8.01 (dd, *J* = 8.6, 2.0 Hz, 1H), 7.63 (dd, *J* = 8.4, 4.2 Hz, 1H), 3.55 (s, 3H).

^{13}C{^{1}H}-NMR (101 MHz, $CDCl_3$) δ 164.6 (q, *J* = 38.6 Hz), 153.0, 147.3, 137.4, 136.2, 131.4, 130.9, 130.8, 124.6, 122.2, 116.0 (q, *J* = 287.5 Hz), 44.4.

^{19}F{^{1}H}-NMR (376 MHz, $CDCl_3$) δ -75.9.

HRMS (ESI) m/z calc. for $C_{12}H_9N_2O_2SF_3Na$ [M+Na]$^+$ 325.0229, 325.0212.

N-Propylquinoline-8-sulfonamide (1y)

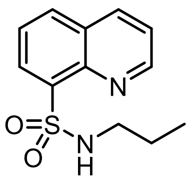

The title compound was synthesized from 8-quinolinesulfonyl chloride (751 mg, 3.30 mmol, 1.1 equiv.) and propylamine (0.246 mL, 3.00 mmol, 1.0 equiv.) according to **GP4**. The reaction was run for 16 h. The crude was purified by column chromatography (*n*-pentane/EtOAc = 1:1, *v*:*v*) to afford the product as off-white solid (169 mg, 0.675 mmol, 22%).

^{1}H-NMR (400 MHz, $CDCl_3$) δ 9.03 (dt, *J* = 4.2, 1.9 Hz, 1H), 8.44 (dt, *J* = 7.3, 1.8 Hz, 1H), 8.29 (dt, *J* = 8.4, 1.9 Hz, 1H), 8.06 (dt, *J* = 8.2, 1.8 Hz, 1H), 7.71 – 7.62 (m, 1H), 7.57 (ddd, *J* = 8.3, 4.3, 2.0 Hz, 1H), 6.32 (t, *J* = 6.4 Hz, 1H), 2.84 (qd, *J* = 6.9, 2.0 Hz, 2H), 1.45 (hd, *J* = 7.3, 1.9 Hz, 2H), 0.81 (td, *J* = 7.4, 2.0 Hz, 3H).

^{13}C{^{1}H}-NMR (101 MHz, $CDCl_3$) δ 151.3, 143.4, 137.2, 136.1, 133.3, 131.4, 128.9, 125.9, 122.4, 45.5, 22.9, 11.3.

HRMS (ESI) m/z calc. for $C_{12}H_{14}N_2O_2SNa$ $[M+Na]^+$ 273.0668, found 273.0666.

N-(Quinolin-3-yl)-4-(trifluoromethoxy)benzenesulfonamide (1z)

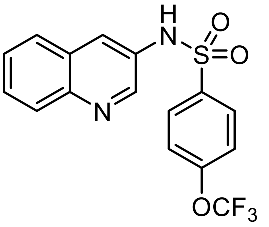

The title compound was synthesized from 4- (trifluoromethoxy)benzenesulfonyl chloride (0.37 mL, 2.2 mmol, 1.1 equiv.) and quinoline-3-amine (288 mg, 2.00 mmol, 1.0 equiv.) according to **GP4**. The reaction was run for 3 h. Purification by column chromatography on silica gel (*n*-pentane/EtOAc = 1:1, *v*:*v*) afforded the product as a white solid (486 mg, 1.32 mmol, 66%).

^{1}H-NMR (400 MHz, $CDCl_3$) δ 8.59 (d, *J* = 2.6 Hz, 1H), 8.09 – 8.00 (m, 2H), 7.89 – 7.83 (m, 2H), 7.78 (dd, *J* = 8.1, 1.4 Hz, 1H), 7.68 (ddd, *J* = 8.4, 6.9, 1.5 Hz, 1H), 7.65 – 7.52 (m, 2H), 7.28 – 7.22 (m, 2H).

^{13}C{^{1}H}-NMR (101 MHz, $CDCl_3$) δ 152.8 (q, *J* = 1.9 Hz), 146.2, 145.7, 137.2, 129.8, 129.6, 129.5, 129.3, 128.0, 127.9, 127.8, 127.3, 121.2 (q, *J* = 1.2 Hz), 120.3 (q, *J* = 259.9 Hz).

^{19}F-NMR (376 MHz, $CDCl_3$) δ -57.73.

HRMS (ESI) m/z calc. for $C_{16}H_{11}N_2O_3SF_3Na$ $[M+Na]^+$ 391.0335, found 391.0334.

N-(Quinolin-3-yl)ethanesulfonamide (1aa)

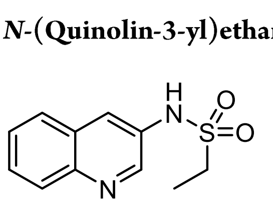

The title compound was synthesized from ethanesulfonyl chloride (0.52 mL, 5.5 mmol, 1.1 equiv.) and quinoline-3-amine (721 mg, 5.00 mmol, 1.0 equiv.) according to **GP4**. The reaction was run for 18 h. Purification by column chromatography on silica gel (*n*-pentane/EtOAc = 1:1, *v*:*v*) afforded the product as a beige solid (838 mg, 3.55 mmol, 71%).

^{1}H-NMR (400 MHz, $CDCl_3$) δ 8.79 (d, *J* = 2.6 Hz, 1H), 8.16 (d, *J* = 2.7, Hz, 1H), 8.07 (d, *J* = 8.4 Hz, 1H), 7.93 (br, 1H), 7.80 (dd, *J* = 8.2, 1.5 Hz, 1H), 7.67 (ddd, *J* = 8.5, 6.9, 1.5 Hz, 1H), 7.56 (ddd, *J* = 8.1, 6.9, 1.2 Hz, 1H), 3.22 (q, *J* = 7.4 Hz, 2H), 1.42 (t, *J* = 7.4 Hz, 3H).

^{13}C{^{1}H}-NMR (101 MHz, $CDCl_3$) δ 145.6, 144.8, 130.9, 129.2, 129.1, 128.2, 127.9, 127.7, 124.9, 46.7, 8.4.

HRMS (ESI) m/z calc. for $C_{11}H_{11}N_2O_2S$ $[M-H]^-$ 235.0547, found 235.0544.

5-(Methylthio)quinazoline (1ab)

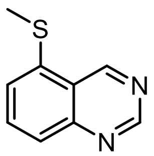

The title compound was synthesized from 5-bromoquinazoline (134 mg, 0.641 mmol, 1.0 equiv.) according to **GP3**. The product was obtained after column chromatography (*n*-pentane/EtOAc = 3:2, *v*:*v*) as yellow solid (68 mg, 0.39 mmol, 60%).

^{1}H-NMR (400 MHz, $CDCl_3$) δ 9.79 (s, 1H), 9.34 (s, 1H), 7.87 – 7.77 (m, 2H), 7.45 (dd, *J* = 6.1, 2.3 Hz, 1H), 2.63 (s, 3H).

^{13}C{^{1}H}-NMR (101 MHz, $CDCl_3$) δ 157.2, 155.7, 150.8, 138.2, 134.1, 125.5, 124.6, 123.7, 16.0.

HRMS (ESI) m/z calc. for $C_9H_9N_2S$ $[M+H]^+$ 177.0481, found 177.0480.

8-(Methylthio)isoquinoline (1ac)

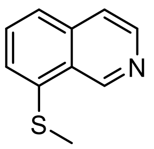

The title compound was synthesized from 8-bromoisoquinoline (416 mg, 2.00 mmol, 1.0 equiv.) according to **GP3**. Purification by column chromatography on silica gel (n-pentane/EtOAc = 4:1, *v*:*v*) afforded the product as a yellow oil (164 mg, 0.936 mmol, 47%).

^{1}H-NMR (400 MHz, $CDCl_3$) δ 9.68 (s, 1H), 8.56 (d, *J* = 5.7 Hz, 1H), 7.64 – 7.58 (m, 3H), 7.44 – 7.40 (m, 1H), 2.61 (s, 3H).

^{13}C{^{1}H}-NMR (101 MHz, $CDCl_3$) δ 149.3, 143.6, 137.8, 136.5, 130.3, 126.8, 124.3, 124.0, 120.8, 16.0.

HRMS (ESI) m/z calc. for $C_{10}H_9NSNa$ $[M+Na]^+$ 198.0348, found 198.0347.

4. Investigation of Reaction Parameters

General Procedure for the Hydrogenation of Sulfur substituted Quinolines:

A 4 mL glass vial (screw-cap) equipped with a stir bar was charged with the catalyst (2.2 mg) and 2-methyl-8-(methylthio)quinoline (19 mg, 0.10 mmol, 1.0 equiv.). Solvent was added (0.66 mL) and the glass vial was placed in a 150 mL stainless steel autoclave under air. The autoclave was pressurized and depressurized four times with hydrogen gas before the final hydrogen pressure of 40 bar was set. The reaction mixture was stirred at the indicated temperature for 24 h. After the autoclave was carefully depressurized, mesitylene (13.9 µL, 0.100 mmol) was added as internal standard and the product yield was determined using GC-FID.

4.1 Screen of Different Catalysts

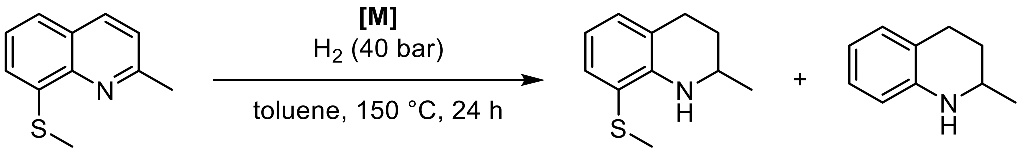

1a **2a** **3a**

Table S2: Evaluation of heterogeneous catalysts.

entry	catalyst	SM	yield 2a	yield 3a
1	Ru-Mo-S-0.33	0%	71%	29%
2	Rh-Mo-S-0.33	10%	87%	3%
3	Co-Mo-S-0.33	0%	69%	31%
4	Cr-Mo-S-0.50	99%	1%	0%
5	Fe-Mo-S-0.33	35%	62%	2%
6	Pd-Mo-S-0.33	1%	87%	12%

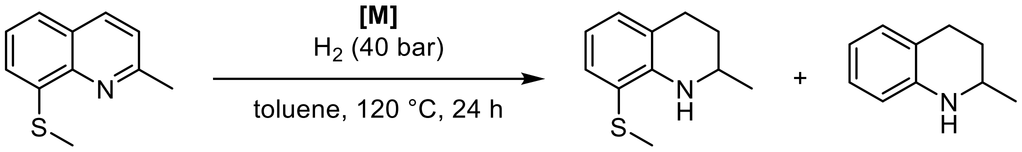

1a **2a** **3a**

Table S3: Evaluation of heterogeneous catalysts.

entry	catalyst	SM	yield 2a	yield 3a
1	Ru-Mo-S-0.33	3%	89%	4%
2	Rh-Mo-S-0.33	60%	39%	1%
3	Co-Mo-S-0.33	17%	79%	2%
4	Pd-Mo-S-0.50	51%	44%	3%

4.2 Screen of Different Solvents

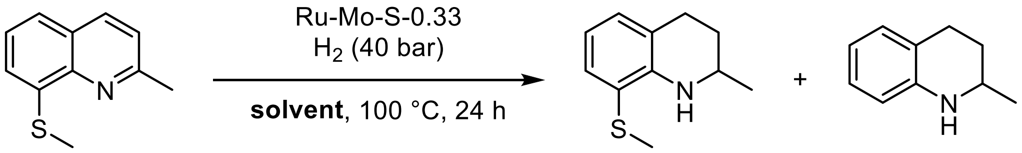

1a **2a** **3a**

Table S4: Evaluation of different solvents

entry	solvent	SM	yield 2a	yield 3a
1	toluene	37%	62%	1%
2	EtOH	0%	96%	3%
3	hexane	54%	45%	1%
4	THF	29%	70%	1%
5	DCE	15%	76%	1%
6	PhCl	29%	70%	1%
7	TFE	30%	63%	4%
8	$CHCl_3$	57%	6%	0%

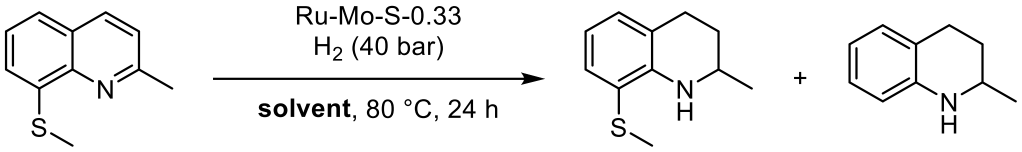

1a **2a** **3a**

Table S5: Evaluation of different alcohols as solvent.

entry	solvent	SM	yield 2a	yield 3a
1	EtOH	43%	51%	1%
2	MeOH	0%	96%	4%
3	*i*PrOH	33%	58%	1%
4	*n*-propanol	16%	80%	1%

4.3 Screen of Different Additives

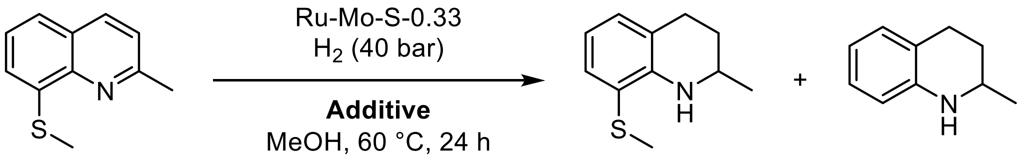

1a **2a** **3a**

Table S6: Evaluation of different Lewis and Brønsted acids.

entry	additive	SM	yield 2a	yield 3a
1	none	53%	47%	0%
2	$Sc(OTf)_3$	51%	48%	0%
3	$Bi(OTf)_3$	45%	55%	0%
4	$In(OTf)_3$	44%	56%	0%
5	HCl conc.	51%	49%	0%

4.4 Screen of Different Ru-catalysts

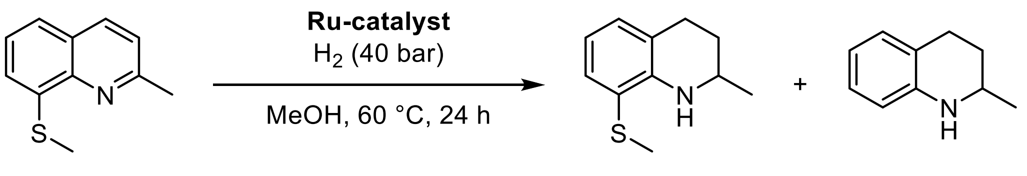

Table S7: Evaluation of different Ru-catalysts.

entry	Ru-catalyst	SM	yield 2a	yield 3a
1	Ru-Mo-S-0.33	49%	51%	0%
2	Ru-Mo-S-0.50	7%	90%	3%
3	Ru-W-S-0.33	1%	96%	2%
4	Ru-S	60%	32%	0%
5	Ru-S-SO_4	23%	76%	0%

Discussion: The best reaction conditions for the hydrogenation of 2-methyl-8-(methylthio)quinoline were found comprising Ru-W-S-0.33 (2.2 mg/0.1 mmol substrate) as catalyst, 40 bar of H_2 pressure and MeOH (0.15 M) as solvent at 60 °C for 24 h. Due to greater steric bulk of other substituents than methyl the reaction temperature for the substrate scope was increased to 80 °C.

Table S6 also quite clearly shows the importance of the secondary metal salt (ammonium molybdate or sodium tungstate) during the hydrothermal synthesis of the ruthenium catalyst, although no MoS_2 or WS_2 is implemented in the final catalyst (see sections 2.1-2.4). While Ru-Mo-S-0.50 and Ru-W-S-0.33 show high activity under the optimized conditions, Ru-S only shows 32% conversion. This can be attributed to the oxidized sulfur species present in the aforementioned catalysts that might bind to the catalyst's surface, stabilizing the nanoparticles, decreasing their size and therefore increasing the activity of the catalyst. This hypothesis is also strengthened by the catalytic result of Ru-S-SO_4 (Table S7, entry 5).

5. Catalyst Recycling Experiments

General Procedure for the Hydrogenation Recycling Experiments:

An 8 mL glass vial (screw-cap) equipped with a stir bar was charged with Ru-W-S-0.33 (13 mg) and 2-methyl-8-(methylthio)quinoline (114 mg, 0.60 mmol, 1.0 equiv.). MeOH was added (4 mL) and the glass vial was placed in a 150 mL stainless steel autoclave under air. The autoclave was pressurized and depressurized four times with hydrogen gas before the final hydrogen pressure of 40 bar was set. The reaction mixture was stirred at 80 °C for 24 h. After the autoclave was carefully depressurized, mesitylene (83.5 µL, 0.600 mmol, 1.0 equiv.) was added as internal standard and the conversion and product yield were determined using GC-FID. The reaction vial was centrifuged, supernatant was decanted with a syringe and the remaining catalyst was washed with Et_2O (twice, after each washing step, the catalyst was centrifuged off again). Then, 2-methyl-8-(methylthio)quinoline (114 mg, 0.60 mmol, 1.0 equiv.) and MeOH (4 mL) were added to the reaction vial and the hydrogenation was set up again. This sequence was repeated seven times and for the seventh time the reaction time was prolonged to 48 h.

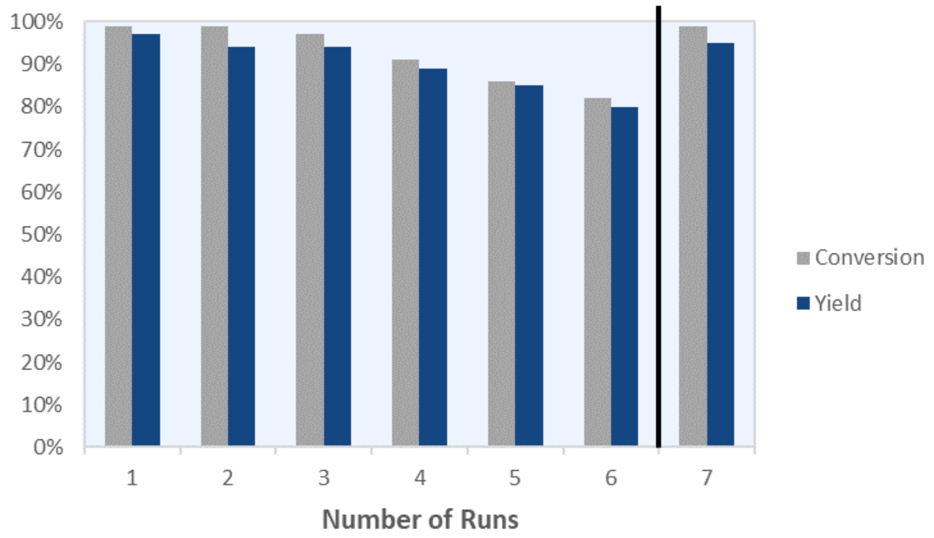

Figure S14: Catalyst recycling experiments for the hydrogenation of quinoline **1a** to THQ **2a** with conversions and yields for seven runs. The reaction time was prolonged to 48 h for the seventh run.

After the seventh run the catalyst was exposed to air for one month and it was then tested if the catalytic activity declined during that time. To our delight, the catalyst still showed good conversion of **1a** (88%) and yielded the product **2a** in 84% yield.

6. Reaction-Condition-Based Sensitivity Assessment

Following a procedure recently published by our group,[14] a reaction-condition-based sensitivity screen was conducted. Key reaction parameters were varied in a systematic manner and the reaction results were compared to the standard reaction conditions. Table S8 gives an overview of the experimental results. A graphic overview is given in Figure S15.

Standard reaction conditions: n = 0.10 mmol, c = 0.15 M, V = 0.66 mL, T = 80 °C, $p(H_2)$ = 40 bar.

Standard reaction procedure: A 4 mL glass vial (screw-cap) equipped with a stir bar was charged with Ru-W-S-0.33 (2.2 mg) and 2-methyl-8-(methylthio)quinoline (19 mg, 0.10 mmol, 1.0 equiv.). MeOH was added (0.66 mL) and the glass vial was placed in a 150 mL stainless steel autoclave under air. The autoclave was pressurized and depressurized four times with hydrogen gas before the final hydrogen pressure of 40 bar was set. The reaction mixture was stirred at 80 °C for 24 h. After the autoclave was carefully depressurized, mesitylene (13.9 µL, 0.100 mmol) was added as internal standard and the product yield was determined using GC-FID.

Big scale conditions: n = 2.50 mmol, c = 0.15 M, V = 16.5 mL, T = 80 °C, $p(H_2)$ = 40 bar.

Analysis: By systematic variation of the reaction parameters we determined the influence of minor changes on the reaction outcome. The reaction proved to be very robust and reproducible by minor changes in temperature, pressure, concentration, water and oxygen levels only having small to no influence on the reaction yield. Interestingly, a lower amount of oxygen resulted in a slightly decreased yield (-8%) due to an increased amount of hydrodesulfurisation. This implies a slightly increased activity of the catalyst under an inert atmosphere.

Table S8: Investigation of the reaction-condition-based sensitivity.

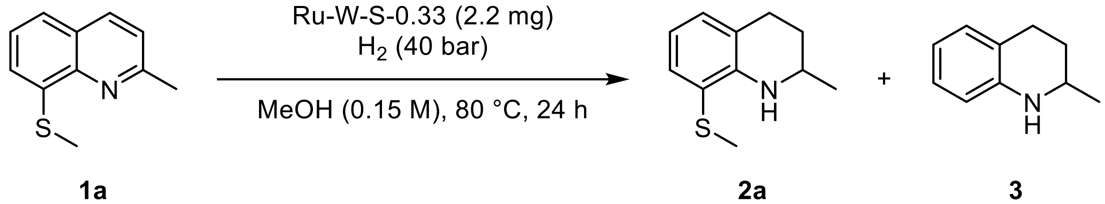

entry	experiment	deviation	yield 2a	yield deviation
1	standard	none	91%	-
2	high *c*	V(MeOH) = 0.5 mL	83%	-8%
3	low *c*	V(MeOH) = 0.9 mL	91%	0%
4	H_2O	+ H_2O (10 µL)	95%	4%
5	high O_2	+ purged with air (6.6 mL)	96%	5%
6	low O_2	+ dry MeOH, + argon atmosphere	83%	-8%
7	high *T*	T = 90 °C	94%	3%
8	low *T*	T = 70 °C	97%	6%
9	high *p*	p = 50 bar	81%	-10%
10	low *p*	p = 30 bar	86%	-5%
11	big scale	n = 2.5 mmol	95%	4%

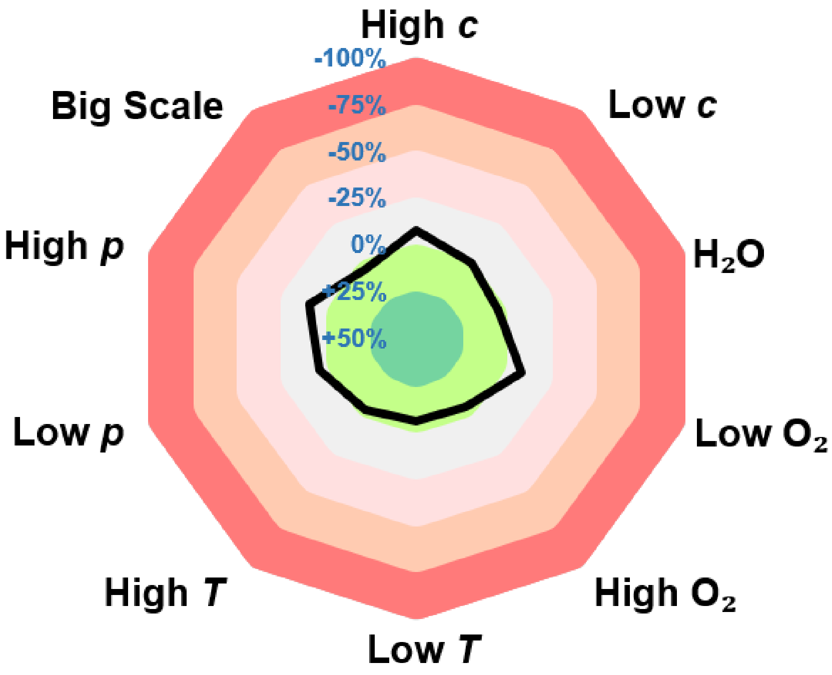

Figure S15: Radar Diagram for the reaction-condition-based sensitivity assessment.

7. Catalytic Hydrogenation Reactions

General Procedure for the Hydrogenation of Sulfur substituted Quinolines:

A 4 mL glass vial (screw-cap) equipped with a stir bar was charged with Ru-W-S-0.33 (6.5 mg) and sulfur substituted quinoline (0.30 mmol, 1.0 equiv.). MeOH was added (2 mL) and the glass vial was placed in a 150 mL stainless steel autoclave under air. The autoclave was pressurized and depressurized four times with hydrogen gas before the final hydrogen pressure of 40 bar was set. If not otherwise stated, the reaction mixture was stirred at 80 °C for 24 h. After the autoclave was carefully depressurized, the reaction mixture was filtered over Celite® using CH_2Cl_2. The solvent was removed *in vacuo* and the crude was purified by column chromatography. The diastereoselectivities were determined by NMR analysis.

2-Methyl-8-(methylthio)-1,2,3,4-tetrahydroquinoline (2a)

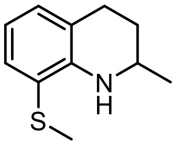

The title compound was synthesized from 2-methyl-8-(methylthio)quinoline (57 mg, 0.30 mmol, 1.0 equiv.) according to the General Procedure. The product was obtained after purification by column chromatography (*n*-pentane/Et_2O = 49:1, *v:v*) as yellow oil (49 mg, 0.25 mmol, 84%).

^{1}H-NMR (400 MHz, $CDCl_3$) δ 7.21 – 7.16 (m, 1H), 6.90 (dq, *J* = 7.4, 1.1 Hz, 1H), 6.55 (t, *J* = 7.5 Hz, 1H), 4.80 (s, 1H), 3.53 – 3.42 (m, 1H), 2.91 – 2.69 (m, 2H), 2.33 (s, 3H), 1.99 – 1.90 (m, 1H), 1.64 – 1.52 (m, 1H), 1.28 (d, *J* = 6.3 Hz, 3H).

^{13}C{^{1}H}-NMR (101 MHz, $CDCl_3$) δ 144.9, 131.3, 129.1, 121.1, 118.3, 116.4, 47.4, 30.0, 27.0, 22.8, 17.9.

HRMS (ESI) m/z calc. for $C_{11}H_{16}NS$ $[M+H]^+$ 194.0994, found 194.0998.

8-(Methylthio)-2-propyl-1,2,3,4-tetrahydroquinoline (2b)

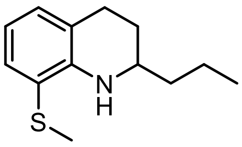

The title compound was synthesized from 8-(methylthio)-2-propylquinoline (65 mg, 0.30 mmol, 1.0 equiv.) according to the General Procedure. The product was obtained after column chromatography (*n*-pentane/Et_2O = 99:1, *v:v*) as yellow oil (60 mg, 0.27 mmol, 90%).

^{1}H-NMR (400 MHz, $CDCl_3$) δ 7.21 – 7.17 (m, 1H), 6.90 (dq, *J* = 7.4, 1.1 Hz, 1H), 6.54 (t, *J* = 7.5 Hz, 1H), 4.92 (s, 1H), 3.38 – 3.29 (m, 1H), 2.87 – 2.70 (m, 2H), 2.33 (s, 3H), 2.01 – 1.92 (m, 1H), 1.67 – 1.39 (m, 5H), 1.00 (t, *J* = 7.3 Hz, 3H).

^{13}C{^{1}H}-NMR (101 MHz, $CDCl_3$) δ 144.8, 131.3, 129.1, 121.3, 118.3, 116.2, 51.5, 39.0, 27.9, 26.8, 19.1, 17.8, 14.4.

HRMS (ESI) m/z calc. for $C_{13}H_{20}NS$ $[M+H]^+$ 222.1311, found 222.1311.

2-Isopropyl-8-(methylthio)-1,2,3,4-tetrahydroquinoline (2c)

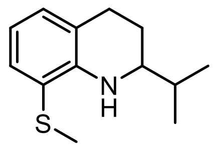

The title compound was synthesized from 2-isopropyl-8-(methylthio)quinoline (65 mg, 0.30 mmol, 1.0 equiv.) according to the General Procedure. The product was obtained after column chromatography (*n*-pentane/Et_2O = 99:1, *v*:*v*) as yellow oil (59 mg, 0.27 mmol, 89%).

^{1}H-NMR (400 MHz, $CDCl_3$) δ 7.22 – 7.17 (m, 1H), 6.90 (dd, *J* = 7.3, 1.4 Hz, 1H), 6.53 (t, *J* = 7.5 Hz, 1H), 4.98 (s, 1H), 3.14 – 3.08 (m, 1H), 2.86 – 2.70 (m, 2H), 2.33 (s, 3H), 1.97 – 1.88 (m, 1H), 1.83 – 1.73 (m, 1H), 1.71 – 1.59 (m, 1H), 1.04 (d, *J* = 6.7 Hz, 3H), 1.01 (d, *J* = 6.8 Hz, 3H).

^{13}C{^{1}H}-NMR (101 MHz, $CDCl_3$) δ 145.2, 131.6, 129.0, 121.4, 118.2, 116.1, 57.5, 32.7, 27.0, 24.4, 18.8, 18.5, 17.9.

HRMS (ESI) m/z calc. for $C_{13}H_{20}NS$ $[M+H]^+$ 222.1311, found 222.1309.

2-Methyl-8-(phenylthio)-1,2,3,4-tetrahydroquinoline (2d)

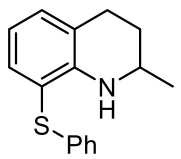

The title compound was synthesized from 2-methyl-8-(phenylthio)quinoline (75 mg, 0.30 mmol, 1.0 equiv.) according to the General Procedure. The product was obtained after column chromatography (*n*-pentane/EtOAc = 98:2, *v*:*v*) as a colorless oil (64 mg, 0.25 mmol, 84%).

^{1}H-NMR (400 MHz, $CDCl_3$) δ 7.28 (dt, *J* = 7.7, 0.6 Hz, 1H), 7.25 – 7.18 (m, 2H), 7.14 – 7.07 (m, 3H), 7.03 (d, *J* = 7.4 Hz, 1H), 6.57 (t, *J* = 7.5 Hz, 1H), 4.89 (br, 1H), 3.42 (dqd, *J* = 9.5, 6.3, 3.3 Hz, 1H), 2.91 – 2.73 (m, 2H), 1.97 – 1.88 (m, 1H), 1.61 – 1.49 (m, 1H), 1.13 (d, *J* = 6.3 Hz, 3H).

^{13}C{^{1}H}-NMR (101 MHz, $CDCl_3$) δ 146.3, 137.4, 135.3, 130.9, 129.0, 126.6, 125.3, 121.6, 116.2, 112.3, 47.3, 29.7, 26.9, 22.6.

HRMS (ESI) m/z calc. for $C_{16}H_{18}NS$ $[M+H]^+$ 256.1155, found 256.1154.

3-Methyl-8-(methylthio)-1,2,3,4-tetrahydroquinoline (2e)

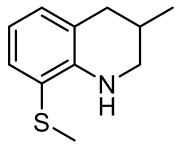

The title compound was synthesized from 3-methyl-8-(methylthio)quinoline (57 mg, 0.30 mmol, 1.0 equiv.) according to the General Procedure. The product was obtained after column chromatography (*n*-pentane/EtOAc = 98:2, *v*:*v*) as a brown oil (45 mg, 0.23 mmol, 77%).

^{1}H-NMR (400 MHz, $CDCl_3$) δ 7.19 (d, *J* = 7.6 Hz, 1H), 6.89 (d, *J* = 7.3 Hz, 1H), 6.55 (t, *J* = 7.5 Hz, 1H), 4.95 (br, 1H), 3.39 (ddd, *J* = 11.2, 3.9, 2.1 Hz, 1H), 2.96 (dd, *J* = 11.2, 9.5 Hz, 1H), 2.79 (ddd, *J* = 16.0, 4.9, 2.2 Hz, 1H), 2.45 (dd, *J* = 15.9, 10.3 Hz, 1H), 2.33 (s, 3H), 2.13 – 1.99 (m, 1H), 1.06 (d, *J* = 6.6 Hz, 3H).

^{13}C{^{1}H}-NMR (101 MHz, $CDCl_3$) δ 144.5, 131.3, 129.3, 121.0, 118.4, 116.4, 49.0, 35.9, 27.0, 19.0, 17.9.

GC-MS (EI) m/z calc. for $C_{11}H_{15}NS$ $[M]^+$ 193.09, found: 193.10 (100), 178.10 (57), 145.10 (30), 144.10 (27), 130.10 (25), 117.10 (14).

2-Methyl-6-(methylthio)-1,2,3,4-tetrahydroquinoline (2f)

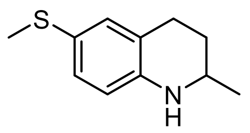

The title compound was synthesized from 2-methyl-6-(methylthio)quinoline (57 mg, 0.30 mmol, 1.0 equiv.) according to the General Procedure. The product was obtained after column chromatography (*n*-pentane/EtOAc = 95:5, *v*:*v*) as a light brown oil in (45 mg, 0.23 mmol, 77%).

^{1}H-NMR (400 MHz, $CDCl_3$) δ 7.05 – 6.98 (m, 2H), 6.42 (d, *J* = 8.1 Hz, 1H), 3.39 (br, 1H), 2.89 – 2.63 (m, 2H), 2.40 (s, 3H), 1.99 – 1.85 (m, 1H), 1.63 – 1.51 (m, 1H), 1.21 (d, *J* = 6.3 Hz, 3H).

^{13}C{^{1}H}-NMR (101 MHz, $CDCl_3$) δ 143.9, 131.7, 129.3, 123.7, 121.9, 114.7, 47.3, 30.0, 26.6, 22.7, 19.5.

HRMS (ESI) m/z calc. for $C_{11}H_{15}NS$ $[M]^+$ 193.0920, found 193.0919.

2-Ethyl-3-methyl-8-(methylthio)-1,2,3,4-tetrahydroquinoline (2g)

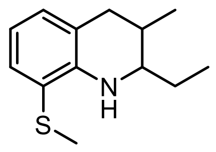

The title compound was synthesized from 2-Ethyl-3-methyl-8-(methylthio)quinoline (65 mg, 0.30 mmol, 1.0 equiv.) at 100 °C according to the General Procedure. The two diastereomeric products were obtained after column chromatography (*n*-pentane/Et_2O = 49:1, *v*:*v*) as yellow liquid (53 mg, 0.24 mmol, 80%; *d.r.* = 1.7:1).

^{1}H-NMR (mixture of diastereomers, 400 MHz, $CDCl_3$) δ 7.22 – 7.16 (m, 1H), 6.92 – 6.87 (m, 1H), 6.57 – 6.50 (m, 1H), 5.05 – 4.89 (m, 1H), 3.24 (ddd, *J* = 8.6, 5.9, 3.1 Hz, 0.64H), 3.00 – 2.87 (m, 1H), 2.76 (ddt, *J* = 16.0, 4.9, 0.7 Hz, 0.39H), 2.54 – 2.42 (m, 1H), 2.35 – 2.30 (m, 3H), 2.18 – 2.08 (m, 0.68H), 1.86 – 1.75 (m, 0.39H), 1.75 – 1.65 (m, 0.39H), 1.59 – 1.40 (m, 2H), 1.02 (td, *J* = 7.6, 2.3 Hz, 4H), 0.91 (d, *J* = 6.9 Hz, 2H).

^{13}C{^{1}H}-NMR (mixture of diastereomers, 101 MHz, $CDCl_3$) δ 144.6, 144.1, 131.5, 131.3, 129.7, 129.1, 120.8, 120.3, 118.1, 117.8, 116.3, 116.0, 58.6, 56.6, 34.9, 34.7, 30.2, 28.9, 27.1, 24.8, 18.5, 17.9, 17.9, 14.1, 10.7, 9.4.

HRMS (ESI) m/z calc. for $C_{13}H_{20}NS$ $[M+H]^+$ 222.1311, found 222.1310.

(3*S*)-5-(Methylthio)-3-(prop-1-en-2-yl)-1,2,3,4,4a,9,9a,10-octahydroacridine (2h)

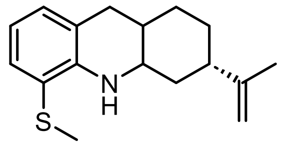

The title compound was synthesized from (*S*)-5-(methylthio)-3-(prop-1-en-2-yl)-1,2,3,4-tetrahydroacridine (81 mg, 0.30 mmol, 1.0 equiv.) at 100 °C according to the General Procedure. The two diastereomeric products were obtained after column chromatography (*n*-pentane/Et_2O = 49:1, *v*:*v*) as yellow oils (*cis*: 39 mg, 0.14 mmol, 48%; *trans*: 20 mg, 0.07 mmol, 24%; overall: 59 mg, 0.22 mmol, 72%).

^{1}H-NMR (*cis*-diastereomer, 400 MHz, $CDCl_3$) δ 7.21 (dt, *J* = 7.7, 1.3 Hz, 1H), 6.90 (dtd, *J* = 7.4, 1.4, 0.6 Hz, 1H), 6.52 (t, *J* = 7.5 Hz, 1H), 4.77 – 4.71 (m, 2H), 4.65 (s, 1H), 3.73 (q, *J* = 3.1 Hz, 1H), 3.05 (ddt, *J* = 16.1, 5.6, 1.2 Hz, 1H), 2.47 (dd, *J* = 16.4, 2.0 Hz, 1H), 2.41 – 2.33 (m, 1H), 2.32 (s, 3H), 1.96 – 1.88 (m, 2H), 1.83 – 1.77 (m, 1H), 1.76 (t, *J* = 1.1 Hz, 3H), 1.63 (ddd, *J* = 13.8, 12.4, 3.0 Hz, 1H), 1.49 – 1.24 (m, 3H).

^{13}C{^{1}H}-NMR (*cis*-diastereomer, 101 MHz, $CDCl_3$) δ 150.3, 144.2, 131.6, 129.8, 119.0, 117.6, 116.1, 108.7, 50.2, 38.2, 37.3, 34.0, 33.1, 31.4, 26.8, 21.2, 18.1.

HRMS (ESI) m/z calc. for $C_{17}H_{24}NS$ $[M+H]^+$ 274.1624, found 274.1625.

3-Methyl-8-(methylthio)-2-phenyl-1,2,3,4-tetrahydroquinoline (2i)

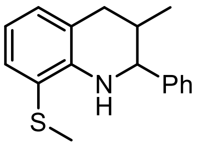

The title compound was synthesized from 3-methyl-8-(methylthio)-2-phenylquinoline (80 mg, 0.30 mmol, 1.0 equiv.) at 100 °C according to the General procedure. The two diastereomeric products were obtained after column chromatography (*n*-pentane/Et_2O = 49:1, *v*:*v*) as yellow oil (66 mg, 0.25 mmol, 82%; *d.r.* > 95:5).

^{1}H-NMR (400 MHz, $CDCl_3$) δ 7.38 – 7.26 (m, 6H), 6.96 (ddt, *J* = 7.6, 1.6, 0.8 Hz, 1H), 6.61 (t, *J* = 7.5 Hz, 1H), 5.33 (s, 1H), 4.62 (dd, *J* = 3.9, 1.9 Hz, 1H), 2.98 (ddd, *J* = 16.1, 4.8, 1.0 Hz, 1H), 2.53 (dd, *J* = 16.1, 6.8 Hz, 1H), 2.35 (s, 3H), 2.38 – 2.28 (m, 1H), 0.82 (d, *J* = 6.9 Hz, 3H).

^{13}C{^{1}H}-NMR (101 MHz, $CDCl_3$) δ 144.5, 142.8, 131.8, 129.7, 128.3, 127.3, 127.2, 120.1, 118.3, 116.6, 59.5, 33.8, 31.8, 18.1, 15.2.

HRMS (ESI) m/z calc. for $C_{17}H_{20}NS$ $[M+H]^+$ 270.1311, found 270.1312.

8-(Methylthio)-2-phenyl-1,2,3,4-tetrahydroquinoline (2j)

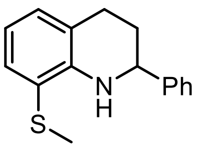

The title compound was synthesized from 8-(methylthio)-2-phenylquinoline (75 mg, 0.30 mmol, 1.0 equiv.) according to the General Procedure. The product was obtained after column chromatography (*n*-pentane/Et_2O = 49:1, *v*:*v*) as yellow solid (43 mg, 0.17 mmol, 56%).

^{1}H-NMR (400 MHz, $CDCl_3$) δ 7.43 – 7.35 (m, 4H), 7.33 – 7.26 (m, 2H), 6.96 (ddt, *J* = 7.4, 1.7, 1.0 Hz, 1H), 6.61 (t, *J* = 7.5 Hz, 1H), 5.24 (s, 1H), 4.55 (dd, *J* = 9.0, 3.5 Hz, 1H), 2.97 – 2.86 (m, 1H), 2.80 – 2.69 (m, 1H), 2.35 (s, 3H), 2.19 – 2.11 (m, 1H), 2.05 – 1.93 (m, 1H).

^{13}C{^{1}H}-NMR (101 MHz, $CDCl_3$) δ 145.0, 144.9, 131.8, 129.2, 128.8, 127.5, 126.5, 121.0, 118.6, 116.6, 56.4, 31.0, 26.6, 18.0.

HRMS (ESI) m/z calc. for $C_{10}H_{18}NS$ $[M+H]^+$ 256.1155, found 256.1154.

2-(4-Fluorophenyl)-8-(methylthio)-1,2,3,4-tetrahydroquinoline (2k)

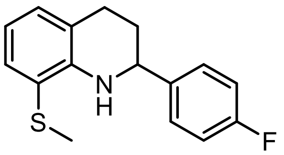

The title compound was synthesized from 2-(4-fluorophenyl)-8-(methylthio)quinoline (81 mg, 0.30 mmol, 1.0 equiv.) according to the General Procedure. The product was obtained after column chromatography (*n*-pentane/Et_2O = 49:1, *v*:*v*) as white solid (72 mg, 0.26 mmol, 88%).

^{1}H-NMR (400 MHz, $CDCl_3$) δ 7.38 – 7.32 (m, 2H), 7.26 (ddt, *J* = 7.7, 1.5, 0.7 Hz, 1H), 7.09 – 7.02 (m, 2H), 6.96 (dq, *J* = 7.4, 1.2 Hz, 1H), 6.62 (t, *J* = 7.6 Hz, 1H), 5.17 (s, 1H), 4.52 (ddd, *J* = 9.0, 3.7, 1.4 Hz, 1H), 2.96 – 2.86 (m, 1H), 2.73 (dt, *J* = 16.3, 5.0 Hz, 1H), 2.34 (s, 3H), 2.18 – 2.07 (m, 1H), 2.01 – 1.89 (m, 1H).

^{13}C{^{1}H}-NMR (101 MHz, $CDCl_3$) δ 162.3 (d, *J* = 245.3 Hz), 144.7, 140.6 (d, *J* = 3.2 Hz), 131.7, 129.2, 128.1 (d, *J* = 8.0 Hz), 120.9, 118.7, 116.9, 115.6 (d, *J* = 21.4 Hz), 55.7, 31.0, 26.5, 18.0.

^{19}F{^{1}H}-NMR (376 MHz, $CDCl_3$) δ -115.4.

HRMS (ESI) m/z calc. for $C_{16}H_{17}NSF$ $[M+H]^+$ 274.1060, found 274.1060.

2-(4-Chlorophenyl)-8-(methylthio)-1,2,3,4-tetrahydroquinoline (2l)

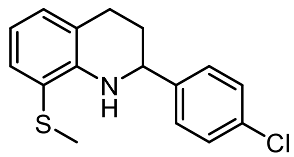

The title compound was synthesized from 2-(4-chlorophenyl)-8-(methylthio)quinoline (86 mg, 0.30 mmol, 1.0 equiv.) according to the General Procedure. The product was obtained after column chromatography (*n*-pentane/Et_2O = 49:1, *v*:*v*) as off-white solid (76 mg, 0.26 mmol, 87%).

^{1}H-NMR (400 MHz, $CDCl_3$) δ 7.36 – 7.30 (m, 4H), 7.28 – 7.24 (m, 1H), 6.95 (dq, *J* = 7.4, 0.8 Hz, 1H), 6.62 (t, *J* = 7.6 Hz, 1H), 5.19 (s, 1H), 4.52 (ddd, *J* = 8.8, 3.6, 1.8 Hz, 1H), 2.95 – 2.85 (m, 1H), 2.76 – 2.67 (m, 1H), 2.34 (s, 3H), 2.18 – 2.07 (m, 1H), 1.99 – 1.89 (m, 1H).

^{13}C{^{1}H}-NMR (101 MHz, $CDCl_3$) δ 144.6, 143.4, 133.1, 131.7, 129.2, 128.9, 127.9, 120.9, 118.8, 116.9, 55.7, 30.8, 26.3, 18.0.

HRMS (ESI) m/z calc. for $C_{16}H_{17}NSCl$ $[M+H]^+$ 290.0765, found 290.0764.

2-(4-Bromophenyl)-8-(methylthio)-1,2,3,4-tetrahydroquinoline (2m)

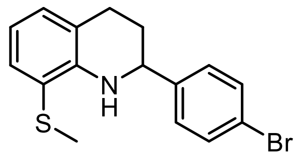

The title compound was synthesized from 2-(4-bromophenyl)-8-(methylthio)quinoline (99 mg, 0.30 mmol, 1.0 equiv.) at 100 °C according to the General Procedure. The product was obtained after column chromatography (*n*-pentane/Et_2O = 49:1, *v*:*v*) as yellow oil (80 mg, 0.25 mmol, 82%).

^{1}H-NMR (400 MHz, $CDCl_3$) δ 7.52 – 7.44 (m, 2H), 7.29 – 7.24 (m, 3H), 6.95 (dd, *J* = 7.4, 1.5 Hz, 1H), 6.61 (t, *J* = 7.5 Hz, 1H), 5.18 (s, 1H), 4.50 (ddd, *J* = 8.7, 3.7, 1.7 Hz, 1H), 2.95 – 2.83 (m, 1H), 2.76 – 2.66 (m, 1H), 2.34 (s, 3H), 2.18 – 2.06 (m, 1H), 2.00 – 1.88 (m, 1H).

^{13}C{^{1}H}-NMR (101 MHz, $CDCl_3$) δ 144.6, 144.0, 131.8, 131.7, 129.2, 128.3, 121.2, 120.9, 118.8, 116.9, 55.7, 30.8, 26.3, 18.0.

GC-MS (EI): m/z calc. for $C_{16}H_{16}BrNS$ $[M]^+$ 335.0166, found: 336.00 (19), 335.00 (100), 334.00 (42), 333.00 (98), 332.00 (25), 320.00 (14), 318.00 (16), 179.10 (11), 178.10 (81), 177.00 (10), 176.00 (13), 164.00 (11), 162.00 (18), 150.00 (18), 131.10 (17), 130.00 (24), 117.00 (15), 104.00 (13), 103.00 (11), 102.10 (12), 77.00 (12).

8-(Methylthio)-2-(thiophen-2-yl)-1,2,3,4-tetrahydroquinoline (2n)

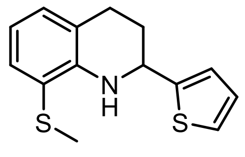

The title compound was synthesized from 8-(methylthio)-2-(thiophen-2-yl)quinoline (77 mg, 0.30 mmol, 1.0 equiv.) at 120 °C according to the General Procedure. The product was obtained after column chromatography (*n*-pentane/Et_2O = 49:1, *v*:*v*) as yellow solid (56 mg, 0.21 mmol, 71%).

^{1}H-NMR (400 MHz, $CDCl_3$) δ 7.29 – 7.21 (m, 2H), 7.03 – 6.93 (m, 3H), 6.61 (t, *J* = 7.5 Hz, 1H), 5.38 (s, 1H), 4.88 – 4.81 (m, 1H), 2.97 – 2.87 (m, 1H), 2.83 – 2.73 (m, 1H), 2.34 (s, 3H), 2.26 – 2.16 (m, 1H), 2.14 – 2.03 (m, 1H).

^{13}C{^{1}H}-NMR (101 MHz, $CDCl_3$) δ 149.3, 144.3, 132.0, 129.3, 126.9, 124.2, 123.6, 121.0, 118.9, 117.1, 52.1, 31.7, 26.3, 18.0.

HRMS (ESI) m/z calc. for $C_{14}H_{16}NS_2$ $[M+H]^+$ 262.0719, found 262.0717.

2-(Furan-2-yl)-8-(methylthio)-1,2,3,4-tetrahydroquinoline (2o)

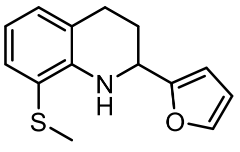

The title compound was synthesized from 2-(furan-2-yl)-8-(methylthio)quinoline (48 mg, 0.20 mmol, 1.0 equiv.) at 100 °C according to the General Procedure. The product was obtained after column chromatography (*n*-pentane/Et_2O = 49:1, *v*:*v*) as yellow oil (38 mg, 0.15 mmol, 77%).

^{1}H-NMR (400 MHz, $CDCl_3$) δ 7.39 (dd, *J* = 1.7, 0.9 Hz, 1H), 7.24 (dd, *J* = 7.7, 1.5 Hz, 1H), 6.93 (dd, *J* = 7.3, 1.5 Hz, 1H), 6.60 (t, *J* = 7.5 Hz, 1H), 6.34 (dd, *J* = 3.2, 1.8 Hz, 1H), 6.20 (d, *J* = 3.2 Hz, 1H), 5.30 (s, 1H), 4.68 – 4.61 (m, 1H), 2.89 – 2.69 (m, 2H), 2.34 (s, 3H), 2.25 – 2.08 (m, 2H).

^{13}C{^{1}H}-NMR (101 MHz, $CDCl_3$) δ 157.0, 144.0, 141.8, 131.7, 129.1, 121.0, 118.9, 117.0, 110.3, 105.5, 49.9, 26.7, 25.7, 18.1.

HRMS (ESI) m/z calc. for $C_{14}H_{15}NOSNa$ $[M+Na]^+$ 268.0767, found 268.0764.

8-Fluoro-6-(methylthio)-1,2,3,4-tetrahydroquinoline (2p)

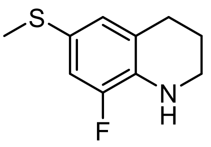

The title compound was synthesized from 8-fluoro-6-(methylthio)quinoline (58 mg, 0.30 mmol, 1.0 equiv.) according to the General Procedure. The product was obtained after column chromatography (*n*-pentane/EtOAc = 95:5, *v*:*v*) as a colorless oil (49 mg, 0.25 mmol, 83%).

^{1}H-NMR (400 MHz, $CDCl_3$) δ 6.85 (dd, *J* = 11.3, 2.0 Hz, 1H), 6.81 – 6.77 (m, 1H), 3.96 (br, 1H), 3.33 (t, *J* = 5.5 Hz, 2H), 2.74 (t, *J* = 6.4 Hz, 2H), 2.40 (s, 3H), 2.04 – 1.86 (m, 2H).

^{13}C{^{1}H}-NMR (101 MHz, $CDCl_3$) δ 150.8 (d, *J* = 240.3 Hz), 132.1 (d, *J* = 12.4 Hz), 126.4 (d, *J* = 2.6 Hz), 124.1 (d, *J* = 4.3 Hz), 123.0 (d, *J* = 7.4 Hz), 114.2 (d, *J* = 19.5 Hz), 41.3, 26.6 (d, *J* = 3.1 Hz), 21.8, 19.0.

^{19}F-NMR (376 MHz, $CDCl_3$) δ -137.96.

HRMS (ESI) m/z calc. for $C_{10}H_{12}FNS$ $[M+H]^+$ 197.0669, found 197.0670.

1-(8-(Isopropylthio)-1,2,3,4-tetrahydroquinolin-2-yl)ethan-1-ol (2q)

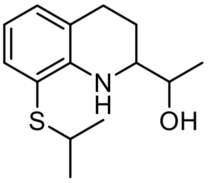

The title compound was synthesized from 1-(8-(isopropylthio)quinolin-2- yl)ethan-1-one (74 mg, 0.30 mmol, 1.0 equiv.) according to the General Procedure. The product was obtained after column chromatography (*n*-pentane/EtOAc = 8:2, *v*:*v*) as a white solid (62 mg, 0.25 mmol, 82%).

^{1}H-NMR (major diastereomer, 400 MHz, $CDCl_3$) δ 6.94 (t, *J* = 7.8 Hz, 1H), 6.76 – 6.69 (m, 1H), 6.42 (d, *J* = 8.0 Hz, 1H), 3.87 (qd, *J* = 6.4, 3.8 Hz, 1H), 3.39 – 3.33 (m, 1H), 3.27 (dt, *J* = 10.4, 3.4 Hz, 1H), 3.09 – 3.01 (m, 1H), 2.73 – 2.59 (m, 1H), 1.99 – 1.92 (m, 1H), 1.79 – 1.57 (m, 1H), 1.34 – 1.22 (m, 9H).

^{13}C{^{1}H}-NMR (major diastereomer, 101 MHz, $CDCl_3$) δ 145.3, 135.8, 126.8, 122.3, 120.0, 113.2, 69.9, 56.4, 37.2, 24.6, 23.4, 23.3, 22.5, 17.7.

HRMS (ESI) m/z calc. for $C_{14}H_{21}NOSNa$ $[M+Na]^+$ 274.1236, found 274.1232.

7-(Isopropylthio)-2,3-dihydroquinolin-4(1*H*)-one (2r)

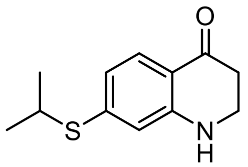

The title compound was synthesized from 7-(isopropylthio)-4-methoxyquinoline (70 mg, 0.30 mmol, 1.0 equiv.) according to the General Procedure. The product was obtained after column chromatography (*n*-pentane/EtOAc = 1:1, *v*:*v*) as a yellow solid (35 mg, 0.16 mmol, 53%).

^{1}H-NMR (400 MHz, $CDCl_3$) δ 7.74 (d, J = 8.4 Hz, 1H), 6.65 (dd, J = 8.4, 1.7 Hz, 1H), 6.55 (d, J = 1.7 Hz, 1H), 4.35 (br, 1H), 3.62 – 3.44 (m, 3H), 2.67 (t, J = 6.8 Hz, 2H), 1.35 (d, J = 6.7 Hz, 6H).

^{13}C{^{1}H}-NMR (101 MHz, $CDCl_3$) δ 192.9, 152.1, 146.2, 128.1, 118.2, 117.2, 114.2, 42.4, 38.1, 36.4, 23.2.

HRMS (ESI) m/z calc. for $C_{12}H_{15}NOSNa$ $[M+Na]^+$ 244.0767, found 244.0766.

3-(1,3-Dithian-2-yl)-1,2,3,4-tetrahydroquinoline (2s)

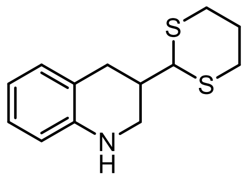

The title compound was synthesized from 3-(1,3-dithian-2-yl)quinoline (74 mg, 0.30 mmol, 1.0 equiv.) at 100 °C according to the General Procedure. The product was obtained after column chromatography (*n*-pentane/EtOAc = 4:1, *v*:*v*) as white solid (52 mg, 0.21 mmol, 69%).

^{1}H-NMR (400 MHz, $CDCl_3$) δ 7.01 – 6.94 (m, 2H), 6.63 (td, J = 7.4, 1.0 Hz, 1H), 6.52 – 6.47 (m, 1H), 4.11 (d, J = 8.1 Hz, 1H), 3.85 (br, 1H), 3.52 (ddd, J = 11.4, 3.3, 1.7 Hz, 1H), 3.30 (dd, J = 11.4, 8.6 Hz, 1H), 3.04 – 2.85 (m, 6H), 2.41 – 2.31 (m, 1H), 2.17 – 2.07 (m, 1H), 1.97 – 1.85 (m, 1H).

^{13}C{^{1}H}-NMR (101 MHz, $CDCl_3$) δ 144.2, 130.0, 127.1, 120.0, 117.5, 114.2, 50.5, 44.7, 37.1, 31.2, 30.4, 30.3, 26.2.

HRMS (ESI) m/z calc. for $C_{13}H_{18}NS_2$ $[M+H]^+$ 252.0875, found 252.0875.

2-Methyl-6-((trifluoromethyl)thio)-1,2,3,4-tetrahydroquinoline (2t)

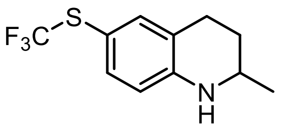

The title compound was synthesized from 2-methyl-6-((trifluoromethyl)thio)quinoline (73 mg, 0.30 mmol, 1.0 equiv.) according to the General Procedure. The product was obtained after column chromatography (*n*-pentane/EtOAc = 9:1, *v*:*v*) as yellow oil (64 mg, 0.26 mmol, 86%).

^{1}H-NMR (400 MHz, $CDCl_3$) δ 7.23 – 7.17 (m, 2H), 6.44 – 6.39 (m, 1H), 4.01 (s, 1H), 3.51 – 3.40 (m, 1H), 2.88 – 2.68 (m, 2H), 1.99 – 1.90 (m, 1H), 1.62 – 1.49 (m, 1H), 1.23 (d, J = 6.4 Hz, 3H).

^{13}C{^{1}H}-NMR (101 MHz, $CDCl_3$) δ 147.19, 137.99, 131.49, 128.42, 121.62, 114.14, 108.69 (q, J = 2.0 Hz), 47.21, 29.42, 26.42, 22.61.

^{19}F{^{1}H}-NMR (376 MHz, $CDCl_3$) δ -44.7.

HRMS (ESI) m/z calc. for $C_{11}H_{13}NSF_3$ $[M+H]^+$ 248.0715, found 248.0721.

2-Methyl-8-(methylsulfinyl)-1,2,3,4-tetrahydroquinoline (2u)

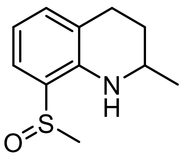

The title compound was synthesized from 2-methyl-8-(methylsulfinyl)quinoline (62 mg, 0.30 mmol, 1.0 equiv.) at 60 °C according to the General Procedure. The product was obtained after column chromatography (*n*-pentane/EtOAc = 4:1, *v*:*v*) as yellow oil (25 mg, 0.12 mmol, 40%).

^{1}H-NMR (400 MHz, $CDCl_3$) δ 7.06 – 6.95 (m, 2H), 6.53 (t, *J* = 7.5 Hz, 1H), 3.52 – 3.40 (m, 1H), 2.93 (s, 3H), 2.93 – 2.81 (m, 1H), 2.77 – 2.70 (m, 1H), 1.99 – 1.90 (m, 1H), 1.69 – 1.54 (m, 1H), 1.25 (d, *J* = 6.2 Hz, 3H).

^{13}C{^{1}H}-NMR (101 MHz, $CDCl_3$) δ 146.2, 132.5, 124.7, 123.2, 120.7, 115.3, 46.8, 37.4, 29.4, 27.1, 22.8.

HRMS (ESI) m/z calc. for $C_{11}H_{15}NOSNa$ $[M+Na]^+$ 232.0767, found 232.0765.

2-Methyl-8-(methylsulfonyl)-1,2,3,4-tetrahydroquinoline (2v)

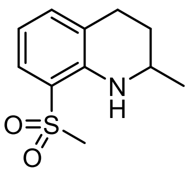

The title compound was synthesized from 2-methyl-8-(methylsulfonyl)quinoline (66 mg, 0.30 mmol, 1.0 equiv.) according to the General Procedure. The product was obtained after column chromatography on silica gel (*n*-pentane/EtOAc = 9:1, *v*:*v*) as a colorless oil (64 mg, 0.28 mmol, 95%).

^{1}H-NMR (400 MHz, $CDCl_3$) δ 7.55 (d, *J* = 8.0 Hz, 1H), 7.14 (d, *J* = 7.4, Hz, 1H), 6.62 (t, *J* = 7.6 Hz, 1H), 6.06 (br, 1H), 3.55 – 3.44 (m, 1H), 3.02 (s, 3H), 2.92 – 2.73 (m, 2H), 2.01 – 1.92 (m, 1H), 1.61 – 1.50 (m, 1H), 1.27 (d, *J* = 6.4 Hz, 3H).

^{13}C{^{1}H}-NMR (101 MHz, $CDCl_3$) δ 144.1, 134.9, 127.7, 123.5, 120.2, 115.5, 47.2, 42.2, 28.7, 27.0, 22.7.

HRMS (ESI) m/z calc. for $C_{11}H_{15}NO_2SNa$ $[M+Na]^+$ 248.0716, found 248.0715.

1,2,3,4-Tetrahydroquinoline-8-sulfonyl fluoride (2w)

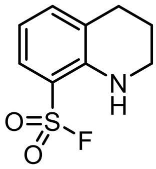

The title compound was synthesized from quinoline-8-sulfonyl fluoride (63 mg, 0.30 mmol, 1.0 equiv.) according to the General Procedure. The product was obtained after column chromatography (*n*-pentane/EtOAc = 4:1, *v*:*v*) as yellow oil (56 mg, 0.26 mmol, 87%).

^{1}H-NMR (400 MHz, $CDCl_3$) δ 7.57 (ddd, *J* = 8.3, 1.6, 0.7 Hz, 1H), 7.19 (ddt, *J* = 7.3, 1.7, 0.8 Hz, 1H), 6.58 (ddd, *J* = 8.2, 7.1, 0.9 Hz, 1H), 6.07 (s, 1H), 3.48 – 3.41 (m, 2H), 2.81 (t, *J* = 6.3 Hz, 2H), 1.99 – 1.90 (m, 2H).

^{13}C{^{1}H}-NMR (101 MHz, $CDCl_3$) δ 145.19, 136.58, 128.93, 123.84, 114.90, 111.26 (d, *J* = 21.1 Hz), 41.78, 27.79, 20.32.

^{19}F{^{1}H}-NMR (376 MHz, $CDCl_3$) δ 63.0.

HRMS (ESI) m/z calc. for $C_9H_{10}NO_2SFNa$ $[M+Na]^+$ 238.0309, found 238.0308.

2,2,2-Trifluoro-*N*-(methyl(oxo)(1,2,3,4-tetrahydroquinolin-7-yl)-λ⁶-sulfaneylidene)acetamide (2x)

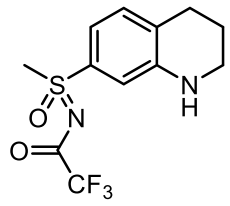

The title compound was synthesized from 2,2,2-trifluoro-*N*-(methyl(oxo)(quinolin-7-yl)-λ^6-sulfaneylidene)acetamide (91 mg, 0.30 mmol, 1.0 equiv.) according to the General Procedure. The product was obtained after column chromatography (*n*-pentane/EtOAc = 9:1, *v*:*v*) as white solid (73 mg, 0.24 mmol, 79%).

^{1}H-NMR (400 MHz, $CDCl_3$) δ 7.11 (dt, *J* = 7.9, 1.0 Hz, 1H), 7.05 (dd, *J* = 7.9, 2.0 Hz, 1H), 6.97 (d, *J* = 2.0 Hz, 1H), 4.33 (s, 1H), 3.38 (s, 3H), 3.36 – 3.32 (m, 2H), 2.80 (t, *J* = 6.3 Hz, 2H), 1.98 – 1.88 (m, 2H).

^{13}C{^{1}H}-NMR (101 MHz, $CDCl_3$) δ 164.30 (q, *J* = 37.8 Hz), 146.05, 134.51, 130.82, 128.23, 116.15 (q, *J* = 288.2 Hz), 113.86, 111.03, 44.54, 41.64, 27.40, 20.94.

^{19}F{^{1}H}-NMR (376 MHz, $CDCl_3$) δ -75.9.

HRMS (ESI) m/z calc. for $C_{12}H_{12}N_2O_2SF_3$ $[M-H]^-$ 305.0566, found 305.0575.

_N_-Propyl-1,2,3,4-tetrahydroquinoline-8-sulfonamide (2y)

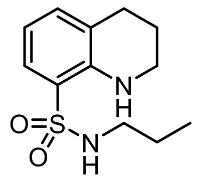

The title compound was synthesized from *N*-propylquinoline-8-sulfonamide (75 mg, 0.30 mmol, 1.0 equiv.) according to the General Procedure. The product was obtained after column chromatography (*n*-pentane/EtOAc = 4:1, *v*:*v*) as colourless oil (64 mg, 0.25 mmol, 84%).

^{1}H-NMR (400 MHz, $CDCl_3$) δ 7.53 (dd, *J* = 8.0, 1.6 Hz, 1H), 7.09 (dd, *J* = 7.4, 1.5 Hz, 1H), 6.61 (t, *J* = 7.6 Hz, 1H), 5.85 (s, 1H), 4.68 (t, *J* = 6.3 Hz, 1H), 3.41 – 3.33 (m, 2H), 2.86 – 2.76 (m, 4H), 1.95 – 1.86 (m, 2H), 1.43 (h, *J* = 7.3 Hz, 2H), 0.84 (t, *J* = 7.4 Hz, 3H).

^{13}C{^{1}H}-NMR (101 MHz, $CDCl_3$) δ 143.0, 134.2, 128.1, 123.7, 120.0, 115.3, 45.1, 41.9, 27.8, 22.8, 21.0, 11.2.

HRMS (ESI) m/z calc. for $C_{12}H_{18}N_2O_2SNa$ $[M+Na]^+$ 277.0981, found 277.0977.

_N_-(1,2,3,4-Tetrahydroquinolin-3-yl)-4-(trifluoromethoxy)benzenesulfonamide (2z)

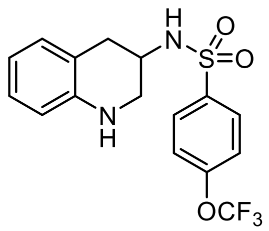

The title compound was synthesized from *N*-(quinolin-3-yl)-4-(trifluoromethoxy)benzenesulfonamide (111 mg, 0.300 mmol, 1.0 equiv.) according to the General Procedure. The product was obtained after column chromatography on silica gel (*n*-pentane/EtOAc = 4:1, *v*:*v*) as a white solid (92 mg, 0.25 mmol, 82%).

^{1}H-NMR (400 MHz, $CDCl_3$) δ 7.95 – 7.86 (m, 2H), 7.32 (d, *J* = 8.0, Hz, 2H), 7.01 (t, *J* = 7.8 Hz, 1H), 6.77 (d, *J* = 7.5 Hz, 1H), 6.64 (td, *J* = 7.4, 1.2 Hz, 1H), 6.50 (dd, *J* = 8.0 Hz, 1H), 5.10 (d, *J* = 8.8 Hz, 1H), 3.97 – 3.88 (m, 1H), 3.79 (br, 1H), 3.31 (d, *J* = 11.5 Hz, 1H), 3.12 (ddd, *J* = 11.4, 4.5, 2.3 Hz, 1H), 2.93 (dd, *J* = 16.5, 4.6 Hz, 1H), 2.56 (dt, *J* = 16.4, 3.1 Hz, 1H).

^{13}C{^{1}H}-NMR (101 MHz, $CDCl_3$) δ 152.2 (q, *J* = 1.9 Hz), 143.3, 139.9, 130.5, 129.2, 127.7, 121.1, 120.4 (q, *J* = 259.3 Hz), 118.6, 117.2, 114.4, 46.4, 46.3, 33.5.

^{19}F-NMR (376 MHz, $CDCl_3$) δ -57.70.

HRMS (ESI+): *m/z* calculated $[C_{16}H_{15}N_2O_3SF_3Na]^+$: 395.0648, found: 395.0647.

N-(1,2,3,4-Tetrahydroquinolin-3-yl)ethanesulfonamide (2aa)

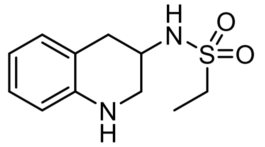

The title compound was synthesized from *N*-(quinolin-3-yl)ethanesulfonamide (71 mg, 0.30 mmol, 1.0 equiv.) according to the General Procedure. The product was obtained after column chromatography on silica gel (*n*-pentane/EtOAc = 1:1, *v*:*v*) as a white solid (61 mg, 0.25 mmol, 84%).

^{1}H-NMR (400 MHz, $CDCl_3$) δ 7.02 (t, *J* = 7.7 Hz, 1H), 6.96 (d, *J* = 7.4 Hz, 1H), 6.68 (td, *J* = 7.4, 1.0 Hz, 1H), 6.53 (d, *J* = 8.0 Hz, 1H), 4.72 (br, 1H), 4.04 – 3.96 (m, 1H), 3.43 (dd, *J* = 11.4, 1.1 Hz, 1H), 3.30 (ddd, *J* = 11.5, 4.6, 2.2 Hz, 1H), 3.16 – 3.02 (m, 3H), 2.77 (dt, *J* = 16.4, 2.8 Hz, 1H), 1.34 (t, *J* = 7.4 Hz, 3H).

^{13}C{^{1}H}-NMR (101 MHz, $CDCl_3$) δ 143.4, 130.5, 127.7, 118.4, 117.5, 114.5, 48.6, 47.1, 46.4, 34.4, 8.6.

HRMS (ESI) m/z calc. for $C_{11}H_{16}N_2O_2SNa$ $[M+Na]^+$ 263.0825, found 263.0822.

5-(Methylthio)-1,2,3,4-tetrahydroquinazoline (2ab)

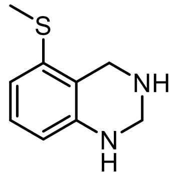

The title compound was synthesized from 5-(methylthio)quinazoline (53 mg, 0.30 mmol, 1.0 equiv.) according to the General Procedure. The product was obtained after column chromatography (*n*-pentane/EtOAc = 9:1, *v*:*v*) as colourless oil (29 mg, 0.16 mmol, 54%).

^{1}H-NMR (400 MHz, $CDCl_3$) δ 7.00 (t, *J* = 7.9 Hz, 1H), 6.59 (dd, *J* = 7.8, 1.1 Hz, 1H), 6.35 (dd, *J* = 8.0, 1.1 Hz, 1H), 4.20 (s, 2H), 3.98 (s, 2H), 2.43 (s, 3H).

^{13}C{^{1}H}-NMR (101 MHz, $CDCl_3$) δ 143.6, 136.3, 127.3, 119.6, 114.8, 112.5, 57.8, 44.6, 15.3.

HRMS (ESI) m/z calc. for $C_9H_{13}N_2S$ $[M+H]^+$ 181.0794, found 181.0794.

8-(Methylthio)-1,2,3,4-tetrahydroisoquinoline (2ac)

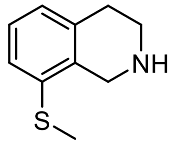

The title compound was synthesized from 8-(methylthio)isoquinoline (53 mg, 0.30 mmol, 1.0 equiv.) according to the General Procedure. The product was obtained after column chromatography on silica gel (CH_2Cl_2/MeOH = 9:1, *v*:*v*) as a colorless oil (35 mg, 0.20 mmol, 65%).

^{1}H-NMR (400 MHz, DMSO-d_6) δ 7.22 (t, *J* = 7.7 Hz, 1H), 7.13 (d, *J* = 8.0 Hz, 1H), 6.97 (d, *J* = 7.5, Hz, 1H), 3.93 (s, 2H), 3.15 (t, *J* = 5.9 Hz, 2H), 2.87 (t, *J* = 6.0 Hz, 2H), 2.47 (s, 3H).

^{13}C{^{1}H}-NMR (101 MHz, DMSO-d_6) δ 135.6, 133.9, 129.1, 127.1, 125.5, 122.7, 43.3, 41.1, 26.7, 14.5.

HRMS (ESI) m/z calc. for $C_{10}H_{14}NS$ $[M+H]^+$ 180.0842, found 180.0844.

8. Unsuccessful Substrates

Starting Materials	Obtained Product Motifs (GC-MS analysis)
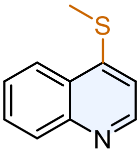	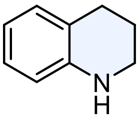 HDS and subsequent reduction of the N-heterocycle.
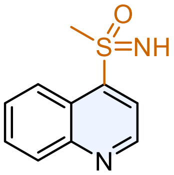	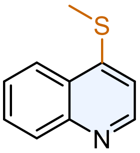 Reduction of the sulfoximine to a thioether.
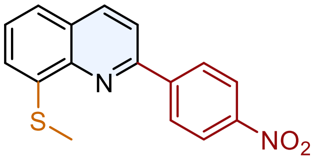	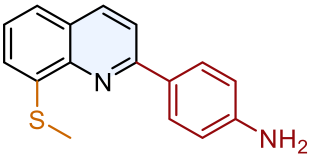 Reduction of the nitro group to an amine.
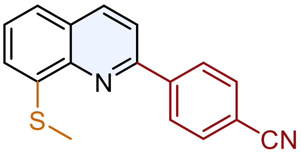	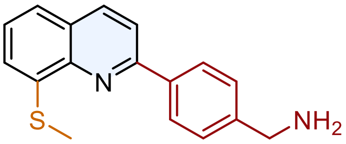 Reduction of the nitrile group to an amine.
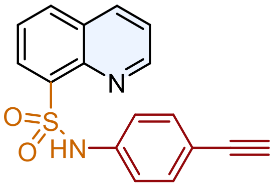	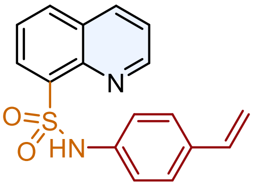 Reduction of the alkyne moiety to an alkene and also SM remaining.
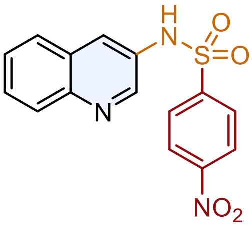	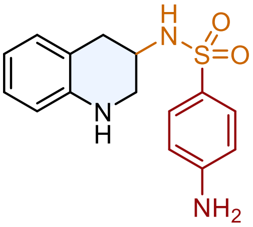 Reduction of the nitro group to an amine.
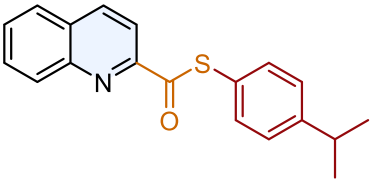	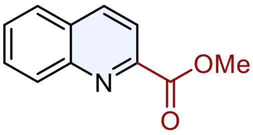 Transesterification with MeOH under reaction conditions.
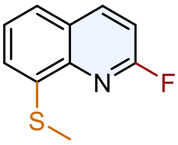	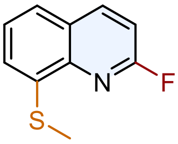 No reactivity at all observed (in THF; S_NAr observed in MeOH), only SM after reaction.
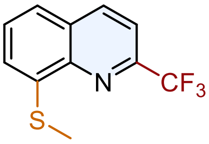	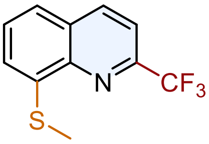 No reactivity at all observed, only SM after reaction.
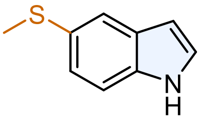	No hydrogenation activity observed, severe HDS could be detected. 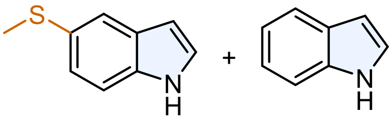

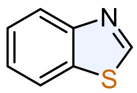	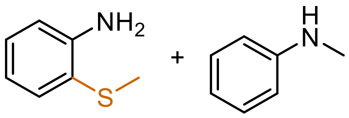	Ring opening and HDS observed.
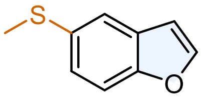	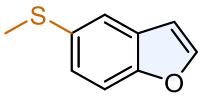	No reactivity at all observed, only SM after reaction.

9. Diversifications of Products 2s and 2w

9.1 Corey-Seebach Type Umpolung of 2s

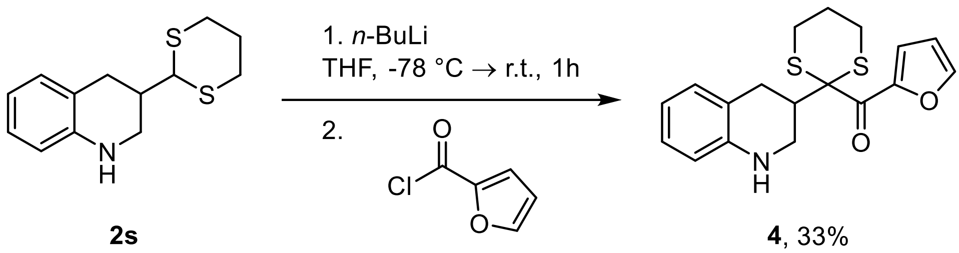

2s **4**, 33%

Under argon in an oven-dried Schlenk tube **2s** (75 mg, 0.30 mmol, 1.0 equiv.) was dissolved in dry THF (3 mL) and cooled to -78 °C. *n*-BuLi (1.6 M in *n*-hexane, 0.39 mL, 0.63 mmol, 2.1 equiv.) was added dropwise and the reaction was slowly warmed to r.t. and stirred for 1 h. The reaction mixture was cooled to -78 °C and 2-furoyl chloride (30 µL, 0.30 mmol, 1.0 equiv.) was added. The reaction was stirred at r.t. for 16 h and quenched with aq. sat. NH_4Cl solution. The phases were separated, aqueous phase extracted with CH_2Cl_2 (3x), combined organic phases dried over $MgSO_4$ and concentrated *in vacuo*. The crude product was purified by column chromatography (*n*-pentane/EtOAc = 4:1) to afford the product as yellow sticky gum (34 mg, 0.10 mmol, 33%).

^{1}H-NMR (400 MHz, $CDCl_3$) δ 7.58 – 7.51 (m, 2H), 6.99 – 6.90 (m, 2H), 6.61 (td, *J* = 7.4, 1.2 Hz, 1H), 6.53 – 6.46 (m, 2H), 3.86 (s, 1H), 3.53 (dt, *J* = 11.3, 2.4 Hz, 1H), 3.47 – 3.40 (m, 1H), 3.26 – 3.10 (m, 3H), 3.06 – 2.96 (m, 1H), 2.77 – 2.67 (m, 3H), 2.11 – 2.00 (m, 1H), 1.97 – 1.81 (m, 1H).

^{13}C{^{1}H}-NMR (101 MHz, $CDCl_3$) δ 182.9, 150.3, 146.3, 144.3, 129.9, 127.1, 120.9, 120.6, 117.7, 114.4, 112.0, 64.9, 43.7, 40.5, 29.0, 28.0, 27.8, 24.8.

HRMS (ESI) m/z calc. for $C_{18}H_{20}NO_2S_2$ $[M+H]^+$ 346.0930, found 346.0926.

9.2 SuFEx Click Reaction of 2w

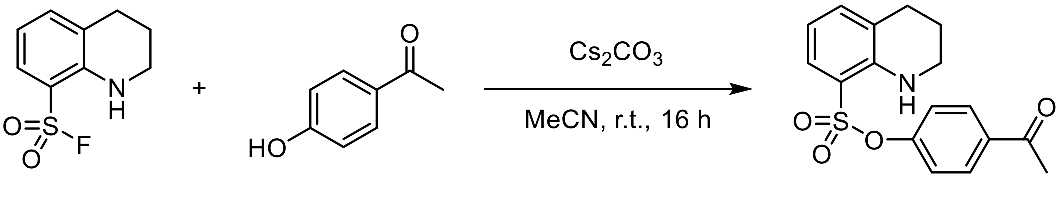

2w **5**, 92%

Prepared according to a literature procedure by Glorius and co-workers.[15] A Schlenk tube was charged with 1,2,3,4-tetrahydroquinoline-8-sulfonyl fluoride (38 mg, 0.18 mmol, 1.0 equiv.), 1-(4-hydroxyphenyl)ethan-1-one (74 mg, 0.54 mmol, 3.0 equiv.), Cs_2CO_3 (0.29 g, 0.90 mmol, 5.0 equiv.) and MeCN (1.8 mL). The mixture was subsequently stirred at r.t. for 18 h, after which it was filtered over Celite® using EtOAc. The solvents were removed *in vacuo* and the residue was purified by column chromatography (*n*-pentane/EtOAc = 4:1) to afford the product as white solid (54 mg, 0.16 mmol, 92%).

^{1}H-NMR (400 MHz, $CDCl_3$) δ 7.92 – 7.84 (m, 2H), 7.29 – 7.25 (m, 1H), 7.15 – 7.07 (m, 3H), 6.44 (dd, *J* = 8.2, 7.2 Hz, 1H), 6.07 (br, 1H), 3.41 (t, *J* = 5.6 Hz, 2H), 2.80 (t, *J* = 6.3 Hz, 2H), 2.56 (s, 3H), 1.97 – 1.88 (m, 2H).

^{13}C{^{1}H}-NMR (101 MHz, $CDCl_3$) δ 153.2, 144.6, 135.8, 130.1, 129.3, 123.4, 122.7, 114.6, 113.5, 41.8, 27.8, 26.8, 20.7.

HRMS (ESI) m/z calc. for $C_{17}H_{18}NO4S$ $[M+H]^+$ 332.0951, found 332.0947.

10. Crystallographic Data

X-Ray diffraction: Data sets for compounds **2l**, **2n** and **2s** were collected with a Bruker D8 Venture Photon III Diffractometer. Programs used: data collection: *APEX4* Version 2021.4-0; cell refinement: *SAINT* Version 8.40B; data reduction: *SAINT* Version 8.40B; absorption correction, *SADABS* Version 2016/2 (Bruker AXS (**2021**) *APEX4 Version 2021.4-0, SAINT Version 8.40B* and *SADABS Bruker AXS area detector scaling and absorption correction Version 2016/2,* Bruker AXS Inc., Madison, Wisconsin, USA); structure solution *SHELXT*-Version 2018-3;[16] structure refinement *SHELXL*-Version 2018-3[17] and graphics, *XP* (Bruker AXS (**1998**) *XP – Interactive molecular graphics, Version 5.1,* Bruker AXS Inc., Madison, Wisconsin, USA). *R*-values are given for observed reflections, and *w*R^2 values are given for all reflections.

X-ray crystal structure analysis of 2l (glo10411): A colorless, prism-like specimen of $C_{16}H_{16}ClNS$, approximate dimensions 0.092 mm x 0.122 mm x 0.185 mm, was used for the X-ray crystallographic analysis. The X-ray intensity data were measured on a single crystal diffractometer Bruker D8 Venture Photon III system equipped with a micro focus tube Cu ImS (CuKα, λ = 1.54178 Å) and a MX mirror monochromator. A total of 1959 frames were collected. The total exposure time was 22.66 hours. The frames were integrated with the Bruker SAINT software package using a wide-frame algorithm. The integration of the data using a triclinic unit cell yielded a total of 10790 reflections to a maximum θ angle of 66.59 (0.84 Å resolution), of which 2412 were independent (average redundancy 4.473, completeness = 97.5%, R_{int} = 3.22%, R_{sig} = 2.53%) and 2299 (95.32%) were greater than 2σ(F^2). The final cell constants of a = 7.4753(2) Å, b = 9.0431(2) Å, c = 10.6525(2) Å, α = 91.2950(10), β = 101.9730(10), γ = 96.0700(10), volume = 699.76(3) Å^3, are based upon the refinement of the XYZ-centroids of 8731 reflections above 20 σ(I) with 12.18° < 2θ < 136.4. Data were corrected for absorption effects using the Multi-Scan method (SADABS). The ratio of minimum to maximum apparent transmission was 0.864. The calculated minimum and maximum transmission coefficients (based on crystal size) are 0.5500 and 0.7290. The structure was solved and refined using the Bruker SHELXTL Software Package, using the space group *P*-1, with Z = 2 for the formula unit, $C_{16}H_{16}ClNS$. The final anisotropic full-matrix least-squares refinement on F^2 with 177 variables converged at R1 = 2.60%, for the observed data and wR2 = 6.97% for all data. The goodness-of-fit was 1.068. The largest peak in the final difference electron density synthesis was 0.269 e^-/Å^3 and the largest hole was -0.188 e^-/Å^3 with an RMS deviation of 0.040 e^-/Å^3. On the basis of the final model, the calculated density was 1.375 g/cm^3 and F(000), 304 e^-. The hydrogen at N1 atom was refined freely, but with N-H distance restraints (DFIX). CCDC Nr.: 2295005.

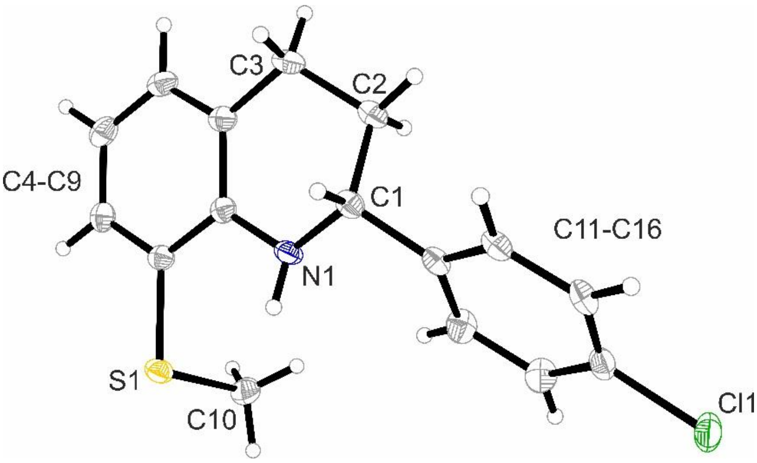

Figure S16: Crystal structure of compound **2l**. Thermal ellipsoids are shown at 50% probability.

X-ray crystal structure analysis of 2n (glo10417): A colorless, plate-like specimen of $C_{14}H_{15}NS_2$, approximate dimensions 0.072 mm x 0.163 mm x 0.185 mm, was used for the X-ray crystallographic analysis. The X-ray intensity data were measured on a single crystal diffractometer Bruker D8 Venture Photon III system equipped with a micro focus tube Mo ImS (MoKα, λ = 0.71073 Å) and a MX mirror monochromator. A total of 675 frames were collected. The total exposure time was 3.75 hours. The frames were integrated with the Bruker SAINT software package using a narrow-frame algorithm. The integration of the data using a triclinic unit cell yielded a total of 16979 reflections to a maximum θ angle of 27.50° (0.77 Å resolution), of which 2861 were independent (average redundancy 5.935, completeness = 98.1%, R_{int} = 3.79%, R_{sig} = 2.39%) and 2655 (92.80%) were greater than $2\sigma(F^2)$. The final cell constants of a = 7.4657(2) Å, b = 8.9227(2) Å, c = 9.7329(2) Å, α = 87.7760(10)°, β = 81.5140(10)°, γ = 81.6040(10)°, volume = 634.28(3) $Å^3$, are based upon the refinement of the XYZ-centroids of 9911 reflections above 20 σ(I) with 6.208° < 2θ < 54.98°. Data were corrected for absorption effects using the Multi-Scan method (SADABS). The ratio of minimum to maximum apparent transmission was 0.951. The calculated minimum and maximum transmission coefficients (based on crystal size) are 0.9300 and 0.9720. The structure was solved and refined using the Bruker SHELXTL Software Package, using the space group *P*-1, with Z = 2 for the formula unit, $C_{14}H_{15}NS_2$. The final anisotropic full-matrix least-squares refinement on F^2 with 159 variables converged at R1 = 3.13%, for the observed data and wR2 = 8.76% for all data. The goodness-of-fit was 1.078. The largest peak in the final difference electron density synthesis was 0.370 $e^-/Å^3$ and the largest hole was -0.335 $e^-/Å^3$ with an RMS deviation of 0.051 $e^-/Å^3$. On the basis of the final model, the calculated density was 1.369 g/cm^3 and F(000), 276 e^-. The hydrogen at N1 atom was refined freely. CCDC Nr.: 2295006.

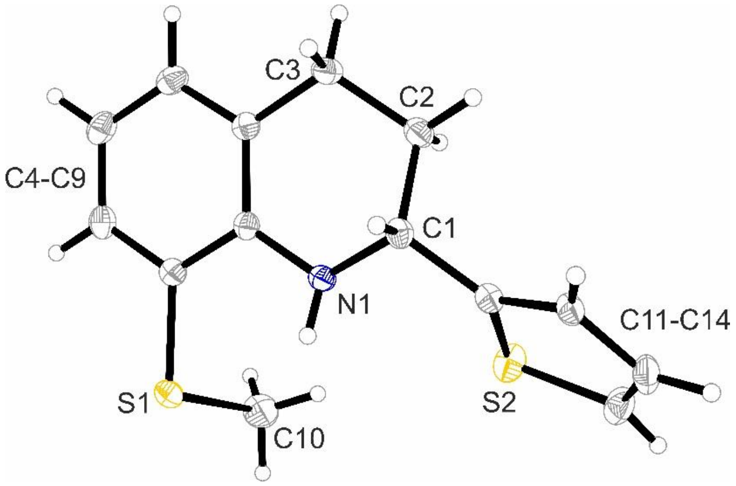

Figure S17: Crystal structure of compound **2n**. Thermal ellipsoids are shown at 50% probability.

X-ray crystal structure analysis of 2s (glo10459): A colorless, plate-like specimen of $C_{13}H_{17}NS_2$, approximate dimensions 0.040 mm x 0.281 mm x 0.286 mm, was used for the X-ray crystallographic analysis. The X-ray intensity data were measured on a single crystal diffractometer Bruker D8 Venture Photon III system equipped with a micro focus tube Cu ImS (CuKα, λ = 1.54178 Å) and a MX mirror monochromator. A total of 1208 frames were collected. The total exposure time was 15.74 hours. The frames were integrated with the Bruker SAINT software package using a wide-frame algorithm. The integration of the data using a monoclinic unit cell yielded a total of 18458 reflections to a maximum θ angle of 66.61° (0.84 Å resolution), of which 2167 were independent (average redundancy 8.518, completeness = 99.9%, R_{int} = 8.48%, R_{sig} = 4.23%) and 1771 (81.73%) were greater than $2\sigma(F^2)$. The final cell constants of a = 12.8678(4) Å, b = 11.3664(3) Å, c = 8.7741(3) Å, β = 107.201(2)°, volume = 1225.91(7) Å^3, are based upon the refinement of the XYZ-centroids of 6966 reflections above 20 σ(I) with 7.191° < 2θ < 133.2°. Data were corrected for absorption effects using the Multi-Scan method (SADABS). The ratio of minimum to maximum apparent transmission was 0.746. The calculated minimum and maximum transmission coefficients (based on crystal size) are 0.4190 and 0.8670. The structure was solved and refined using the Bruker SHELXTL Software Package, using the space group $P2_1/c$, with Z = 4 for the formula unit, $C_{13}H_{17}NS_2$. The final anisotropic full-matrix least-squares refinement on F^2 with 149 variables converged at R1 = 4.68%, for the observed data and wR2 = 12.13% for all data. The goodness-of-fit was 1.063. The largest peak in the final difference electron density synthesis was 0.513 e$^-$/Å^3 and the largest hole was -0.331 e$^-$/Å^3 with an RMS deviation of 0.072 e$^-$/Å^3. On the basis of the final model, the calculated density was 1.362 g/cm^3 and F(000), 536 e$^-$. The hydrogen at N1 atom was refined freely. CCDC Nr.: 2295007.

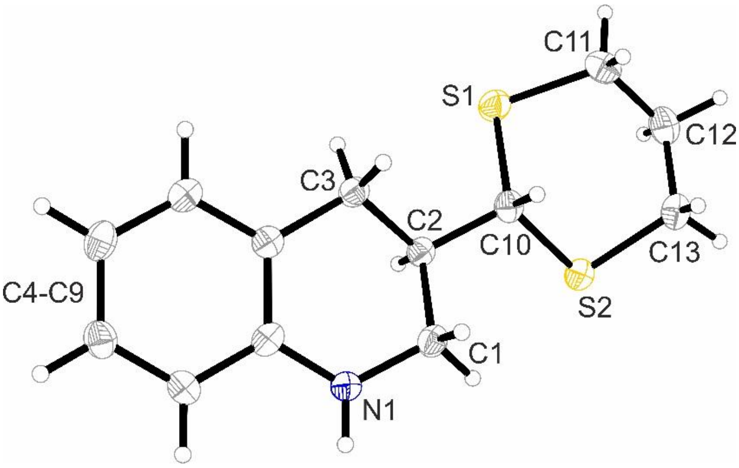

Figure S18: Crystal structure of compound **2s**. Thermal ellipsoids are shown at 50% probability.

11. References

(1) Sorribes, I.; Liu, L.; Corma, A. Nanolayered Co–Mo–S Catalysts for the Chemoselective Hydrogenation of Nitroarenes. *ACS Catal.* **2017**, *7*, 2698–2708.

(2) Powell, C. *X-ray Photoelectron Spectroscopy Database XPS, Version 4.1, NIST Standard Reference Database 20.*

(3) Fantauzzi, M.; Elsener, B.; Atzei, D.; Rigoldi, A.; Rossi, A. Exploiting XPS for the identification of sulfides and polysulfides. *RSC Adv.* **2015**, *5*, 75953–75963.

(4) Gobbo, P.; Biesinger, M. C.; Workentin, M. S. Facile synthesis of gold nanoparticle (AuNP)-carbon nanotube (CNT) hybrids through an interfacial Michael addition reaction. *Chem. Commun.* **2013**, *49*, 2831–2833.

(5) Denmark, S. E.; Venkatraman, S. On the mechanism of the Skraup-Doebner-Von Miller quinoline synthesis. *J. Org. Chem.* **2006**, *71*, 1668–1676.

(6) Joseph, P. A.; Priyadarshini, S.; Kantam, M. L.; Sreedhar, B. Investigation of the scope and mechanism of copper catalyzed regioselective methylthiolation of aryl halides. *Tetrahedron* **2013**, *69*, 8276–8283.

(7) Srinivasulu, A.; Shantharjun, B.; Vani, D.; Ashalu, K. C.; Mohd, A.; Wencel-Delord, J.; Colobert, F.; Reddy, K. R. Iron-Catalyzed Minisci Type Acetylation of N -Heteroarenes Mediated by $CH(OEt)_3$/TBHP. *Eur. J. Org. Chem.* **2019**, 1815–1819.

(8) Oechsner, R. M.; Wagner, J. P.; Fleischer, I. Acetate Facilitated Nickel Catalyzed Coupling of Aryl Chlorides and Alkyl Thiols. *ACS Catal.* **2022**, *12*, 2233–2243.

(9) Khodaei, M.; Bahrami, K.; Karimi, A. H_2O_2/Tf_2O System: An Efficient Oxidizing Reagent for Selective Oxidation of Sulfanes. *Synthesis* **2008**, 1682–1684.

(10) Li, C.; Kähny, M.; Breit, B. Rhodium-catalyzed chemo-, regio-, and enantioselective addition of 2-pyridones to terminal allenes. *Angew. Chem. Int. Ed.* **2014**, *53*, 13780–13784.

(11) Mukherjee, H.; Debreczeni, J.; Breed, J.; Tentarelli, S.; Aquila, B.; Dowling, J. E.; Whitty, A.; Grimster, N. P. A study of the reactivity of $S^{(VI)}$-F containing warheads with nucleophilic amino-acid side chains under physiological conditions. *Org. Biomol. Chem.* **2017**, *15*, 9685–9695.

(12) Lohier, J.-F.; Glachet, T.; Marzag, H.; Gaumont, A.-C.; Reboul, V. Mechanistic investigation of the NH-sulfoximination of sulfide. Evidence for λ^6-sulfanenitrile intermediates. *Chem. Commun.* **2017**, *53*, 2064–2067.

(13) Wang, X.; Rissanen, K.; Bolm, C. A One-Pot Domino Reaction Providing Fluorinated 5,6-Dihydro-1,2-thiazine 1-Oxides from Sulfoximines and 1-Trifluoromethylstyrenes. *Org. Lett.* **2023**, *25*, 1569–1572.

(14) Pitzer, L.; Schäfers, F.; Glorius, F. Rapid Assessment of the Reaction-Condition-Based Sensitivity of Chemical Transformations. *Angew. Chem. Int. Ed.* **2019**, *58*, 8572–8576.

(15) Erchinger, J. E.; Hoogesteger, R.; Laskar, R.; Dutta, S.; Hümpel, C.; Rana, D.; Daniliuc, C. G.; Glorius, F. EnT-Mediated N-S Bond Homolysis of a Bifunctional Reagent Leading to Aliphatic Sulfonyl Fluorides. *J. Am. Chem. Soc.* **2023**, *145*, 2364–2374.

(16) Sheldrick, G. M. SHELXT - integrated space-group and crystal-structure determination. *Acta Cryst.* **2015**, *A71*, 3–8.

(17) Sheldrick, G. M. Crystal structure refinement with SHELXL. *Acta Cryst.* **2015**, *C71*, 3–8.

12. NMR Spectra

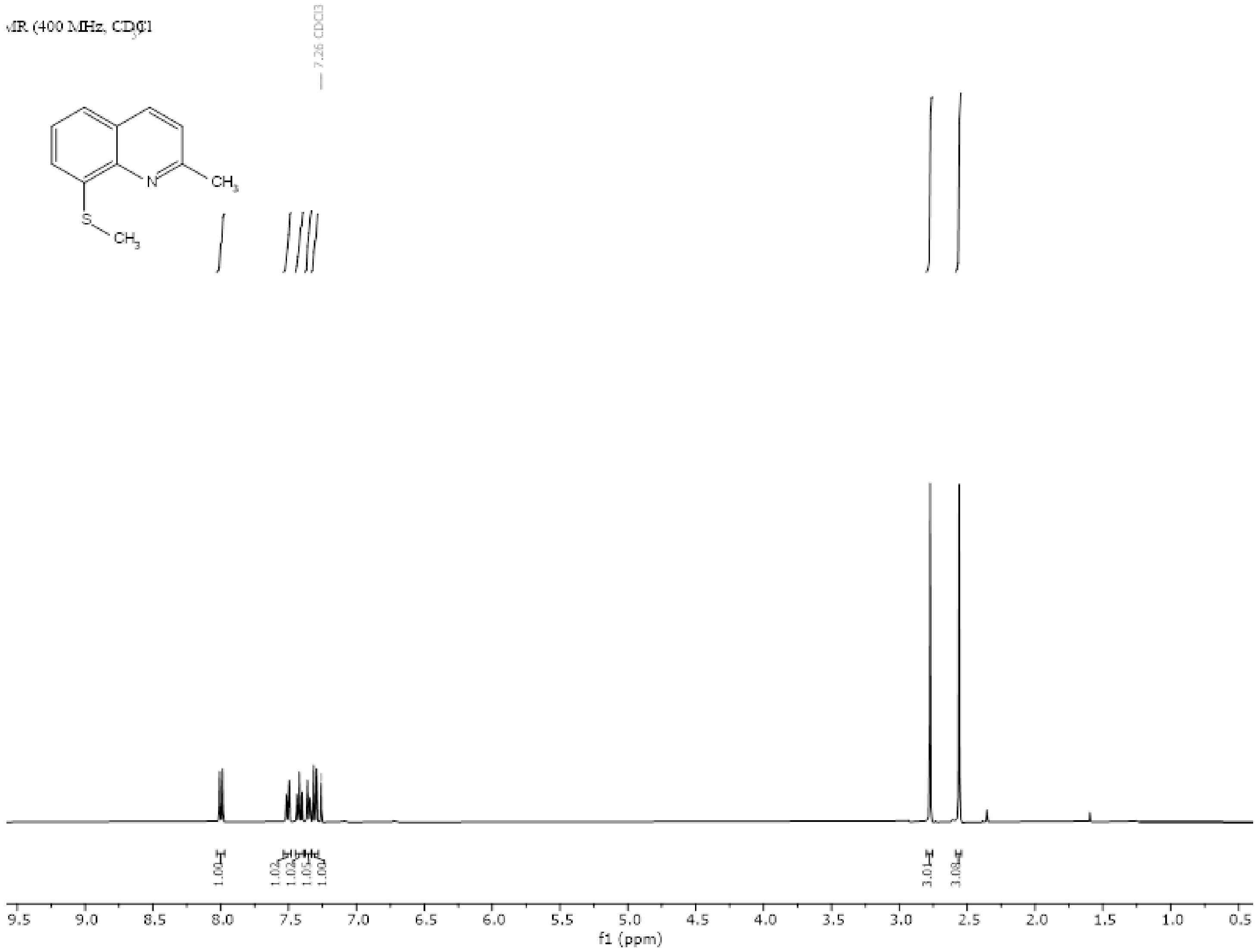

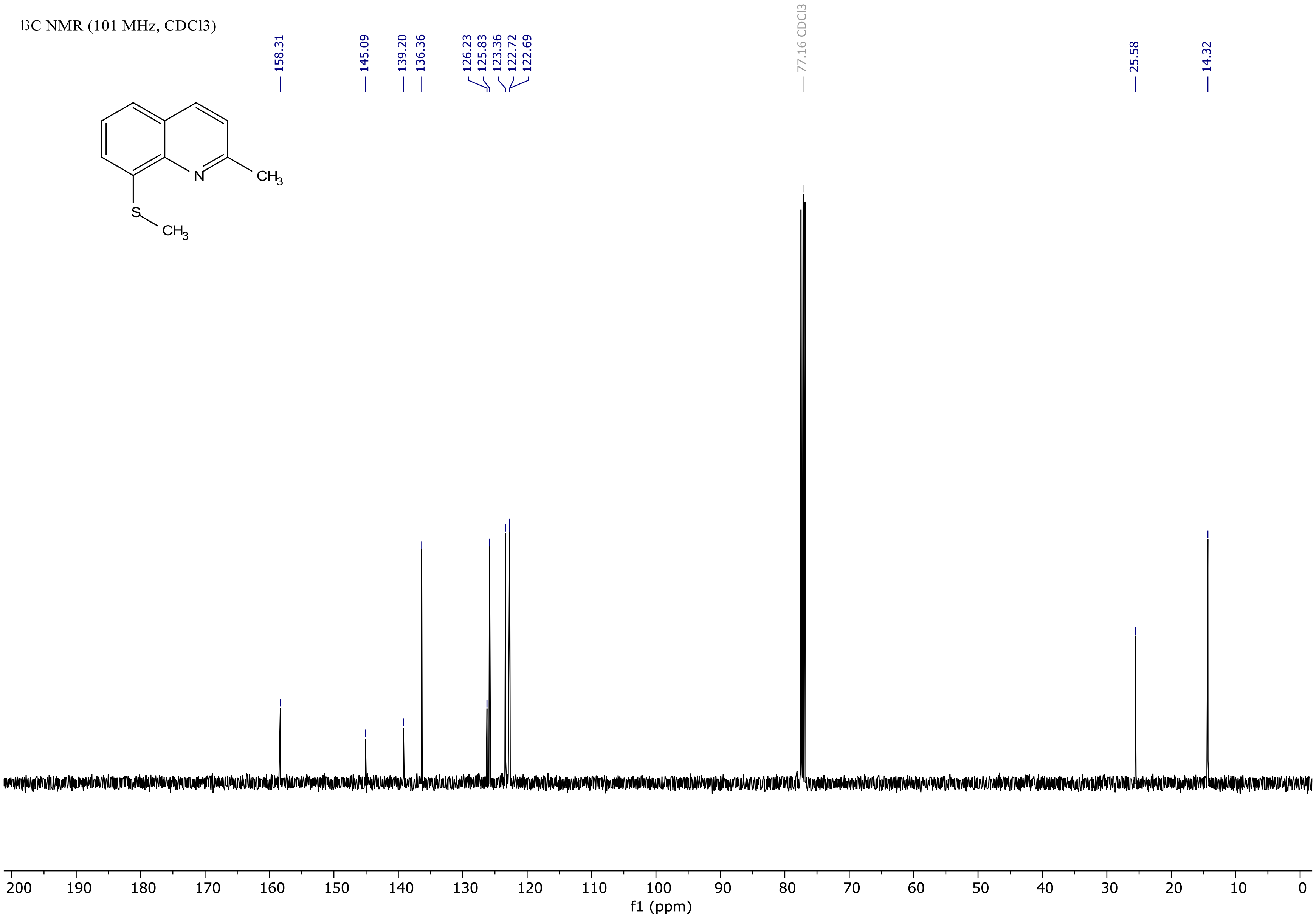
l3C NMR (101 MHz, CDCl3)
158.31
145.09
139.20
136.36
126.23
125.83
123.36
122.72
122.69
77.16 CDCl3
25.58
14.32
N
CH3
S
CH3
200 190 180 170 160 150 140 130 120 110 100 90 80 70 60 50 40 30 20 10 0
f1 (ppm)

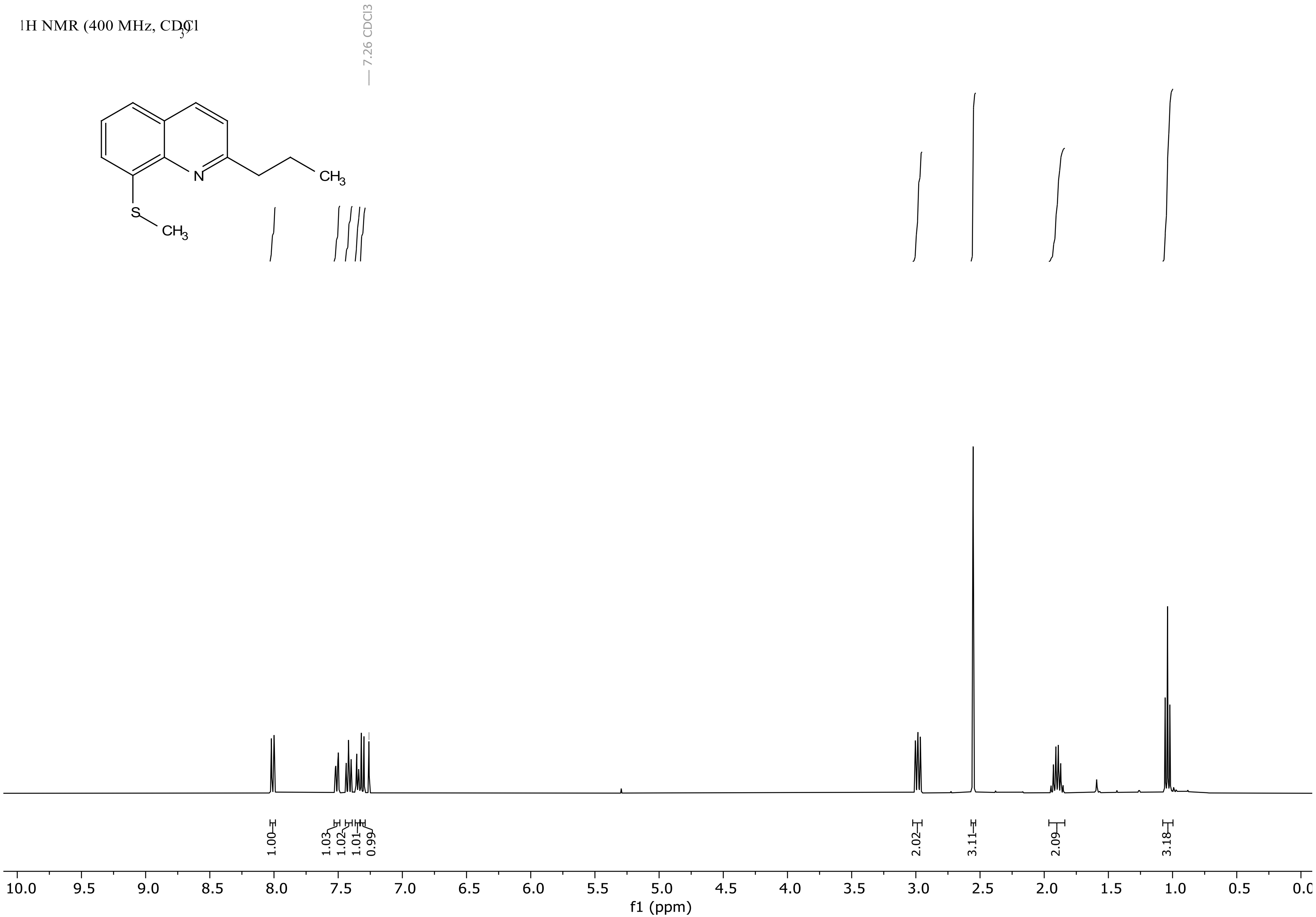
1H NMR (400 MHz, CDCl3)
7.26 CDCl3
N
S
CH3
CH3
1.00
1.03
1.02
1.01
0.99
2.02
3.11
2.09
3.18
10.0
9.5
9.0
8.5
8.0
7.5
7.0
6.5
6.0
5.5
5.0
4.5
4.0
3.5
3.0
2.5
2.0
1.5
1.0
0.5
f1 (ppm)

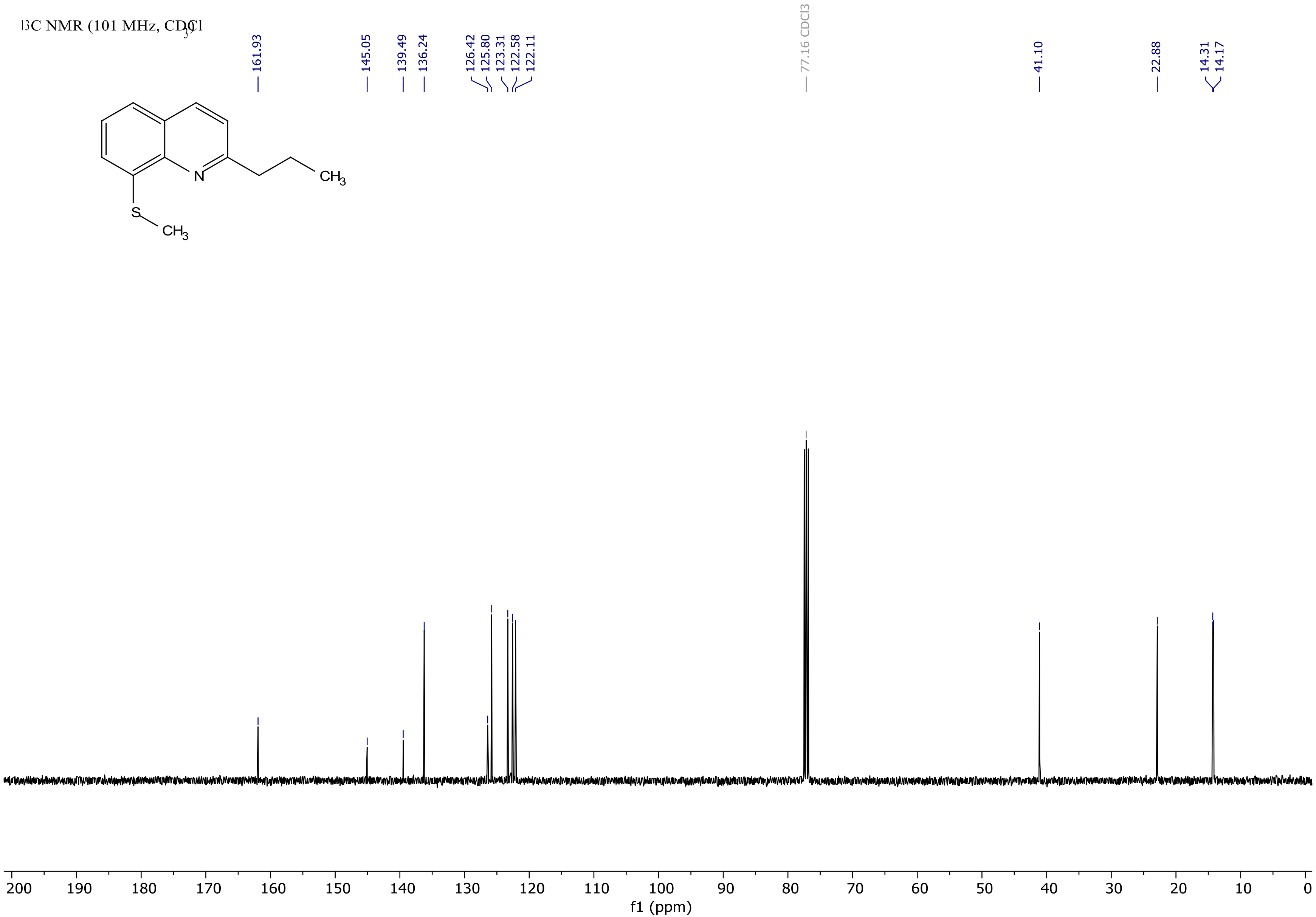
13C NMR (101 MHz, CDCl3)
161.93
145.05
139.49
136.24
126.42
125.80
123.31
122.58
122.11
77.16 CDCl3
41.10
22.88
14.31
14.17
N
S
CH3
CH3
200
190
180
170
160
150
140
130
120
110
100
90
80
70
60
50
40
30
20
10
0
f1 (ppm)

1H NMR (400 MHz, $CDCl_3$)

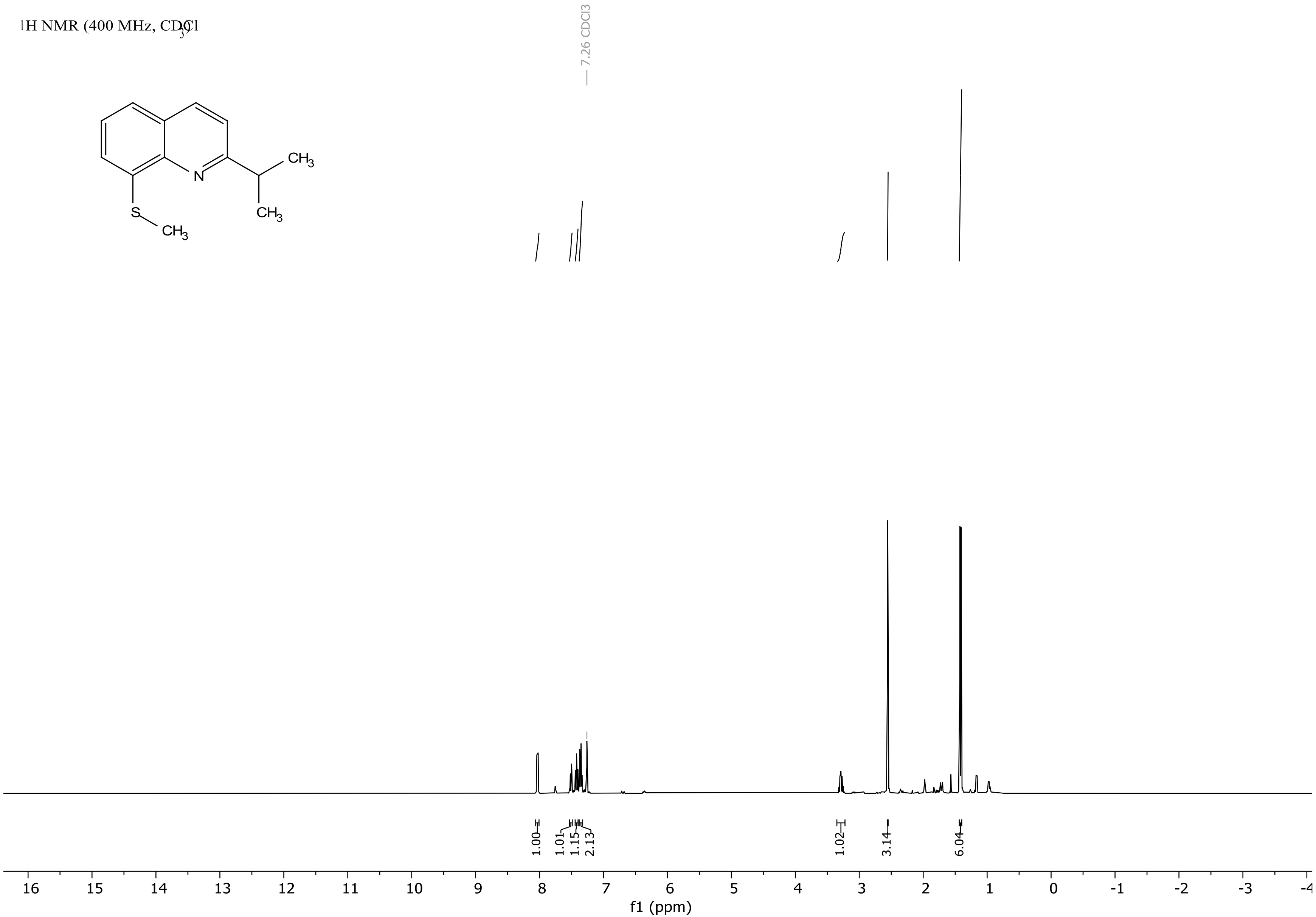

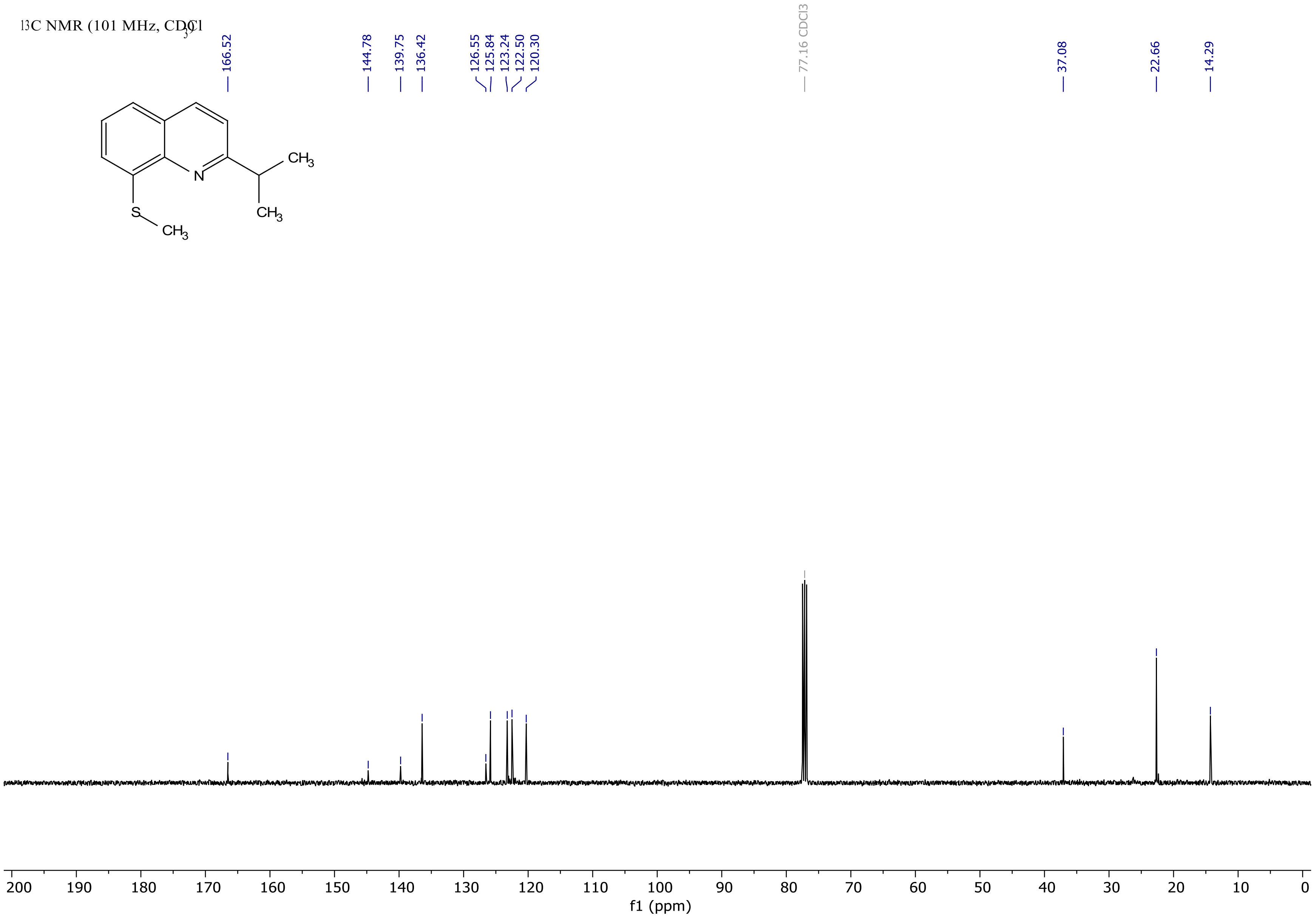
13C NMR (101 MHz, CDCl3)
166.52
144.78
139.75
136.42
126.55
125.84
123.24
122.50
120.30
77.16 CDCl3
37.08
22.66
14.29
CH3
N
S
CH3
CH3
200
190
180
170
160
150
140
130
120
110
100
90
80
70
60
50
40
30
20
10
0
f1 (ppm)

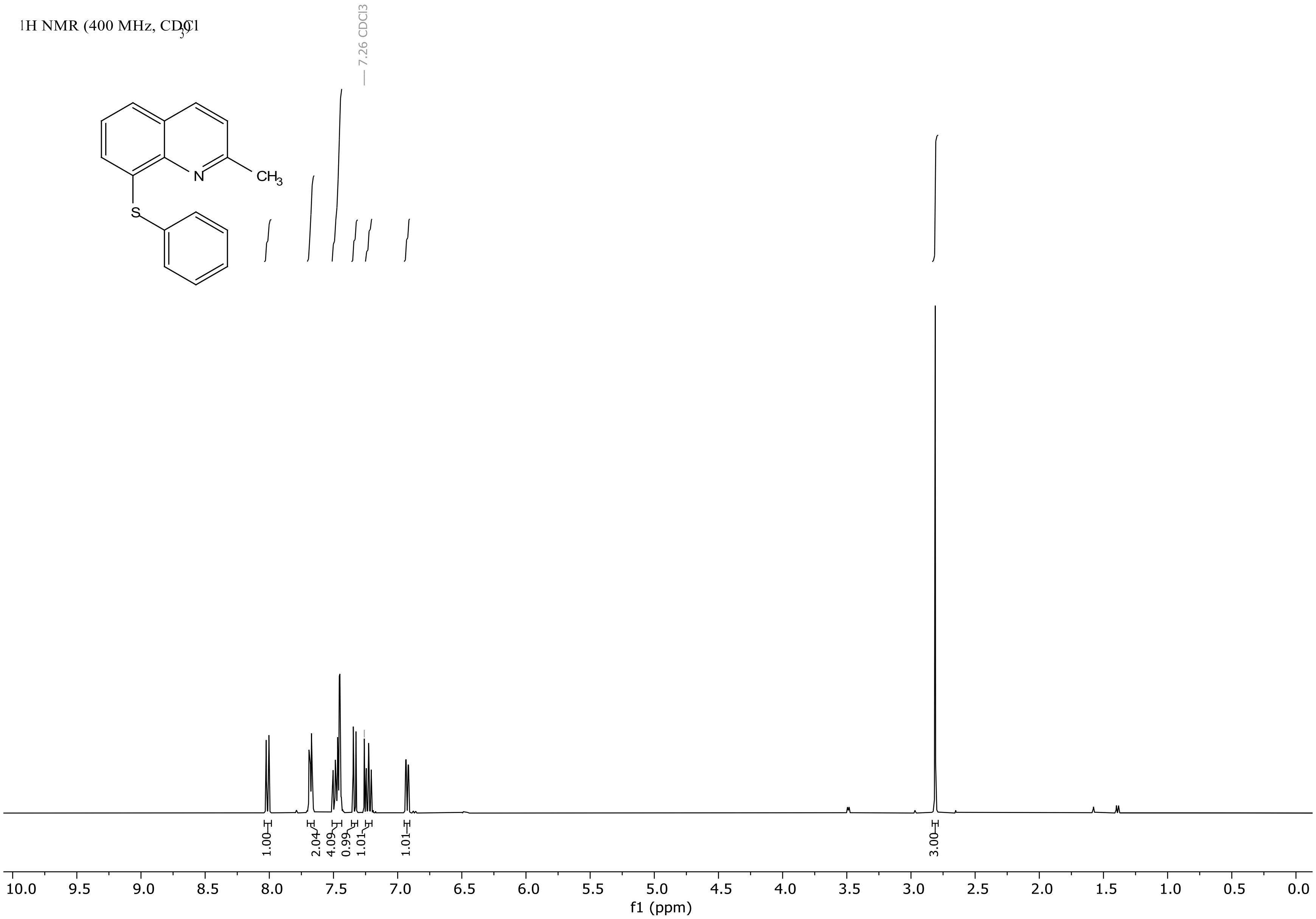
1H NMR (400 MHz, CDCl3)
7.26 CDCl3
N
CH3
S
1.00
2.04
4.09
0.99
1.01
1.01
3.00
10.0 9.5 9.0 8.5 8.0 7.5 7.0 6.5 6.0 5.5 5.0 4.5 4.0 3.5 3.0 2.5 2.0 1.5 1.0 0.5 0.0
f1 (ppm)

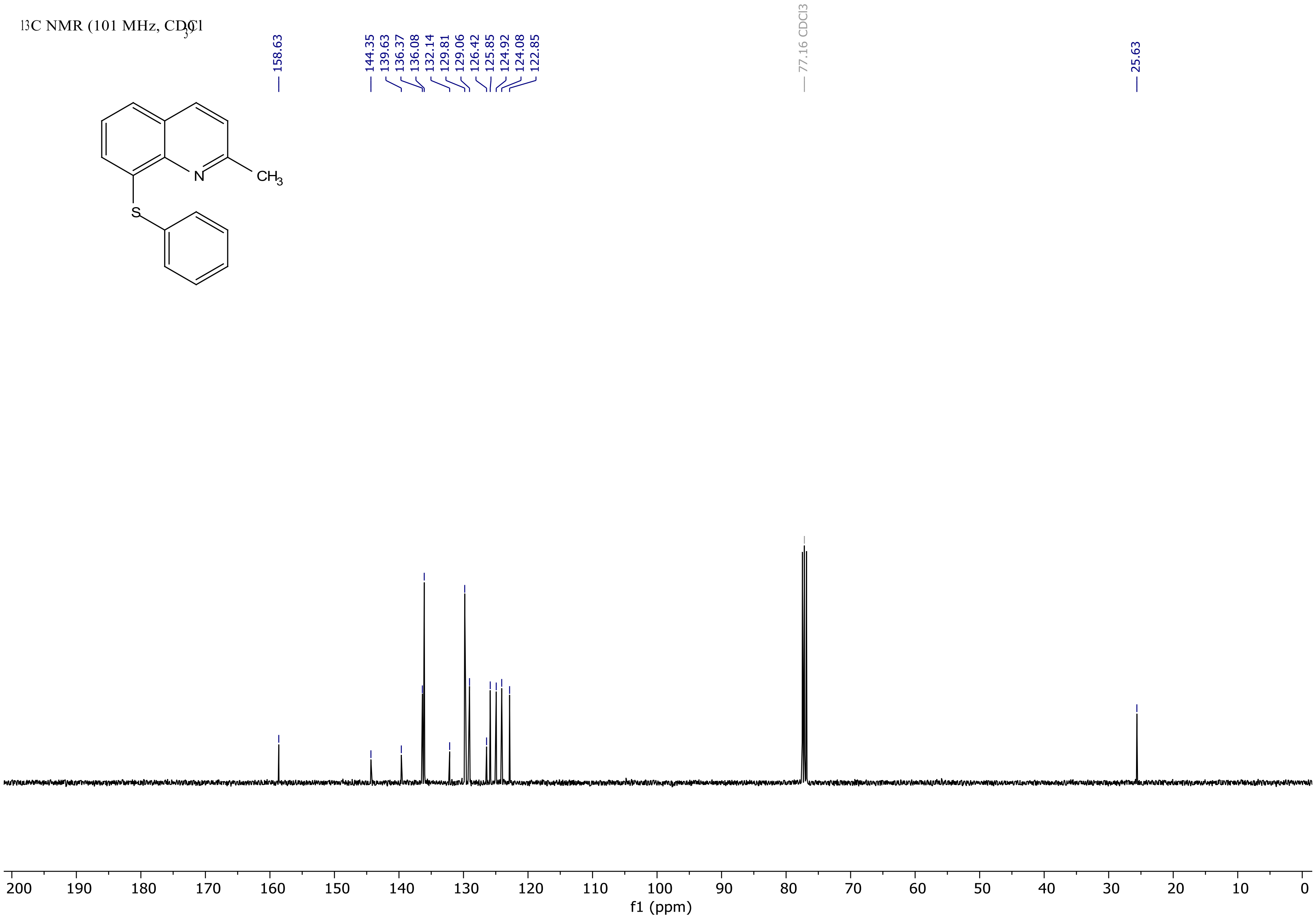
13C NMR (101 MHz, CDCl3)
158.63
144.35
139.63
136.37
136.08
132.14
129.81
129.06
126.42
125.85
124.92
124.08
122.85
77.16 CDCl3
25.63
N
CH3
S
200
190
180
170
160
150
140
130
120
110
100
90
80
70
60
50
40
30
20
10
0
f1 (ppm)

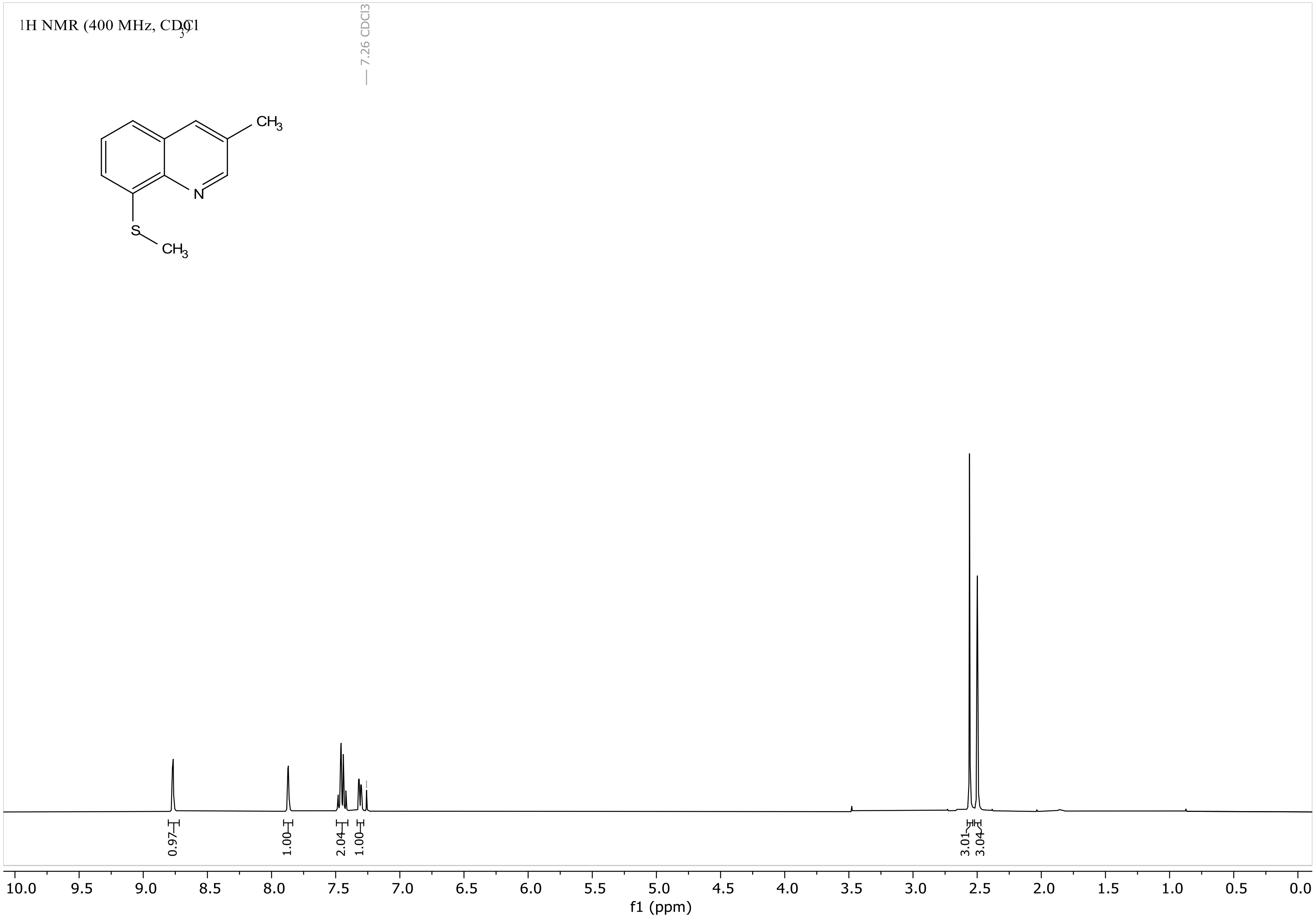

1H NMR (400 MHz, CDCl3)
7.26 CDCl3
CH3
N
S
CH3
0.97
1.00
2.04
1.00
3.01
3.04
10.0 9.5 9.0 8.5 8.0 7.5 7.0 6.5 6.0 5.5 5.0 4.5 4.0 3.5 3.0 2.5 2.0 1.5 1.0 0.5 0.0
f1 (ppm)

^{13}C NMR (101 MHz, $CDCl_3$)

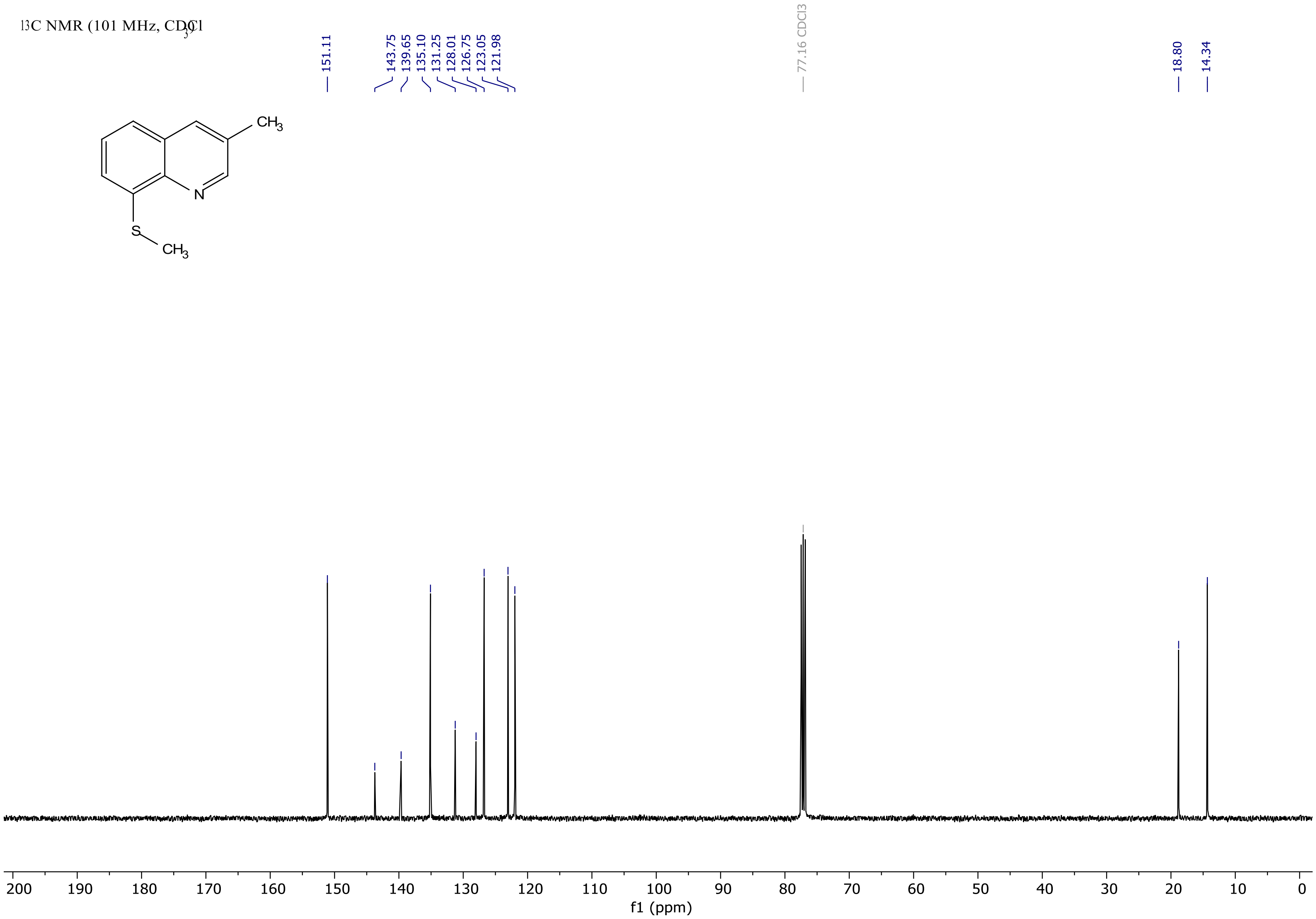

1H NMR (400 MHz, CDCl3)

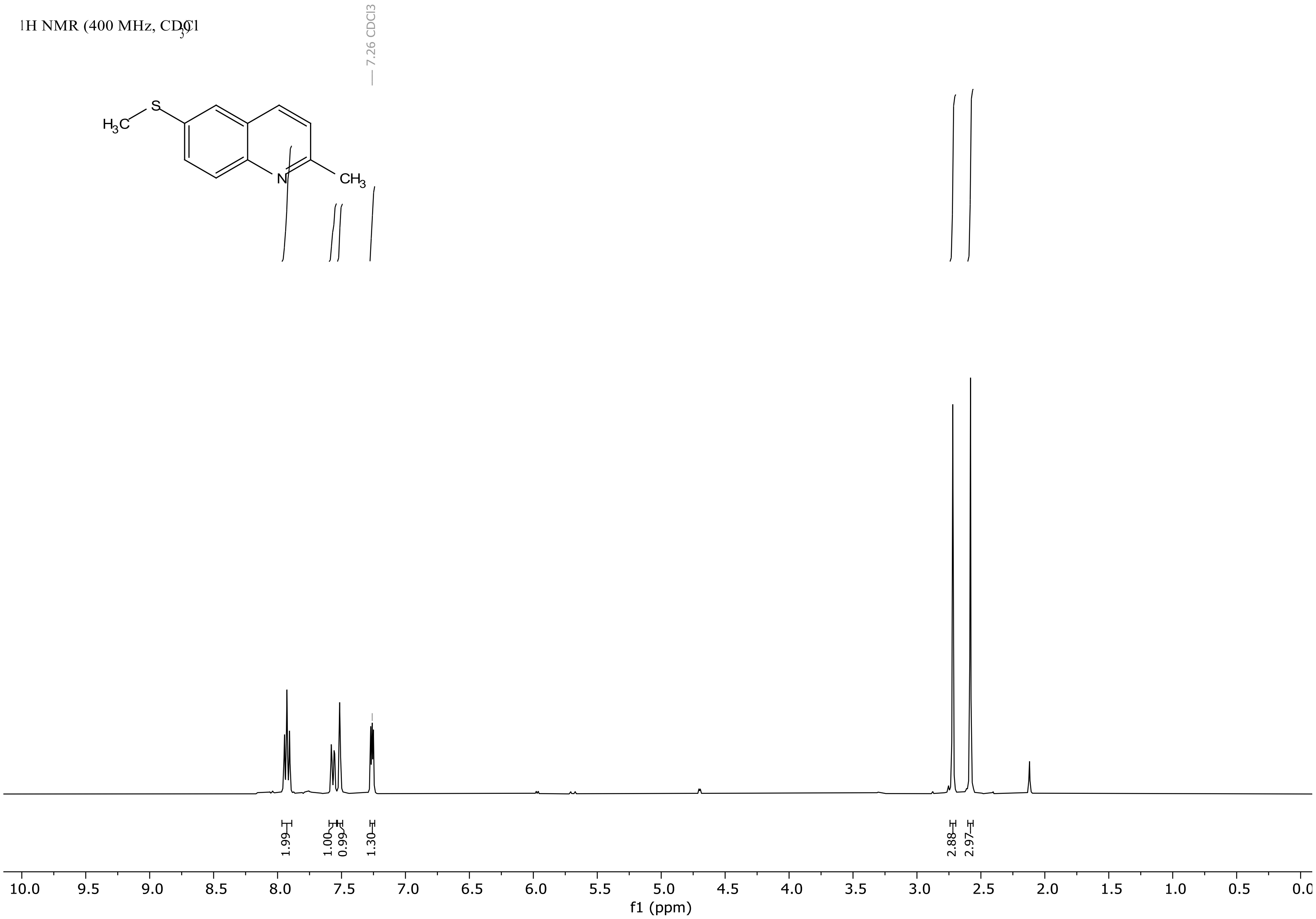

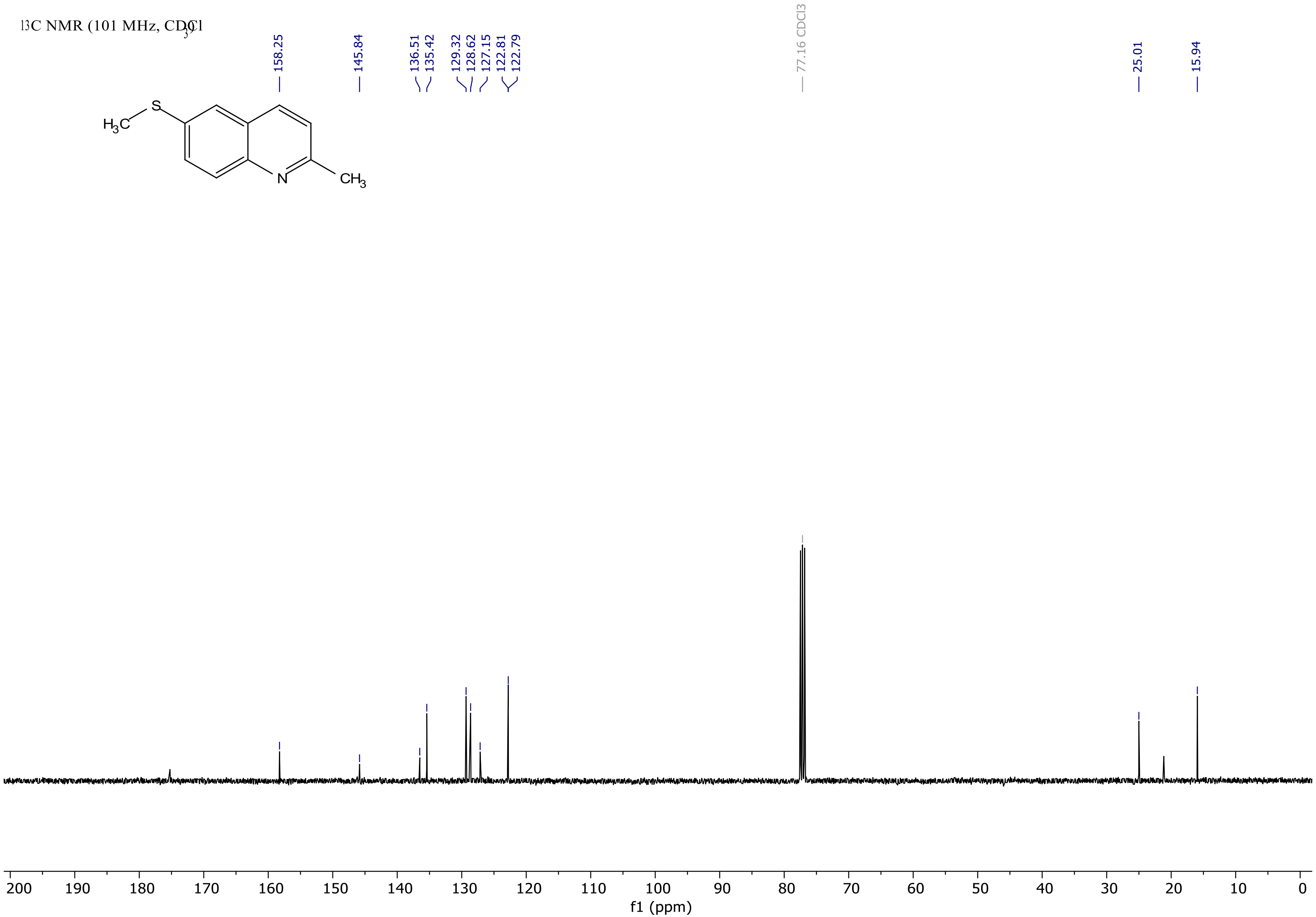
13C NMR (101 MHz, CDCl3)
158.25
145.84
136.51
135.42
129.32
128.62
127.15
122.81
122.79
77.16 CDCl3
25.01
15.94
S
H3C
N
CH3
200 190 180 170 160 150 140 130 120 110 100 90 80 70 60 50 40 30 20 10 0
f1 (ppm)

1H NMR (400 MHz, $CDCl_3$)

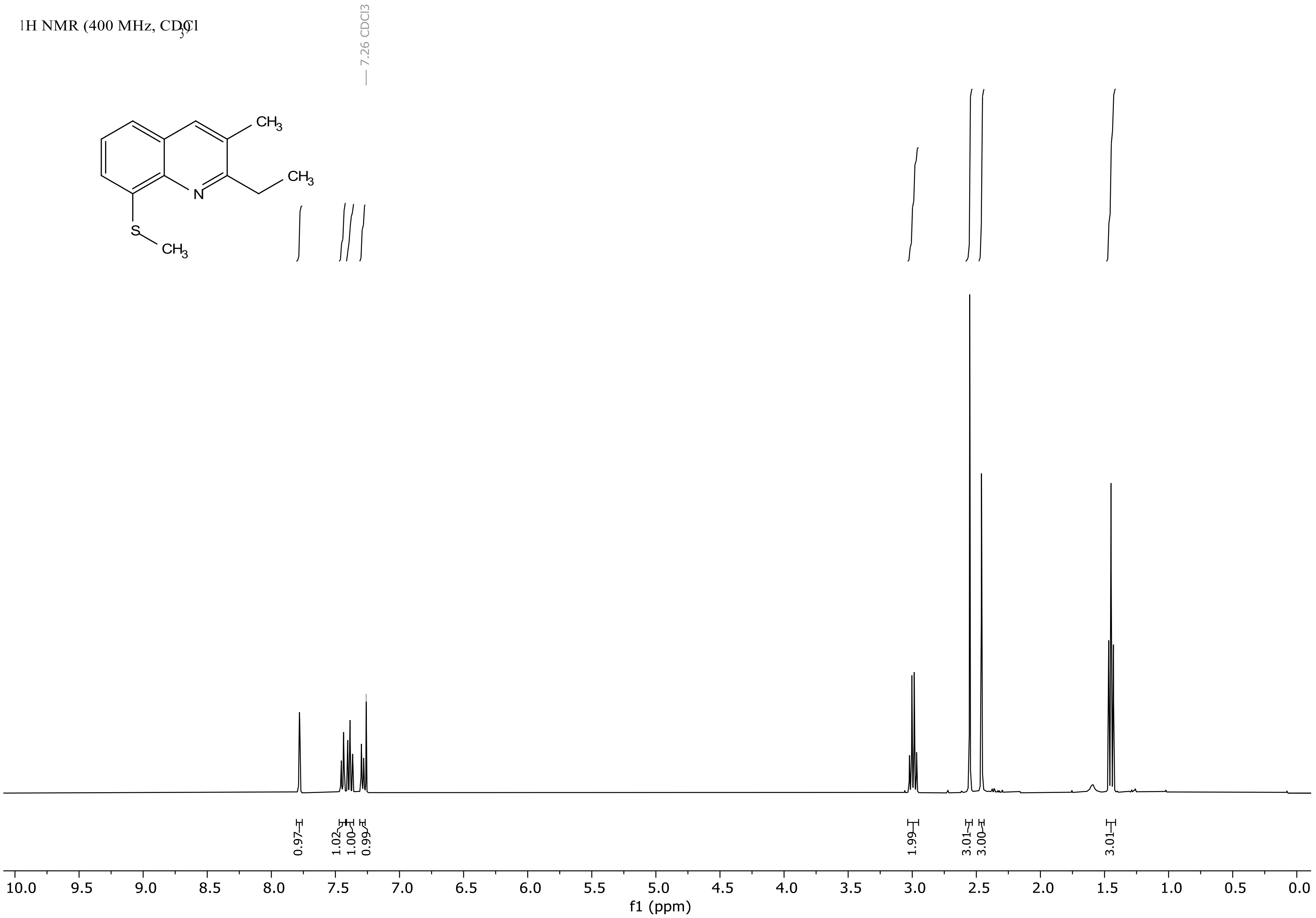

13C NMR (101 MHz, CDCl3)

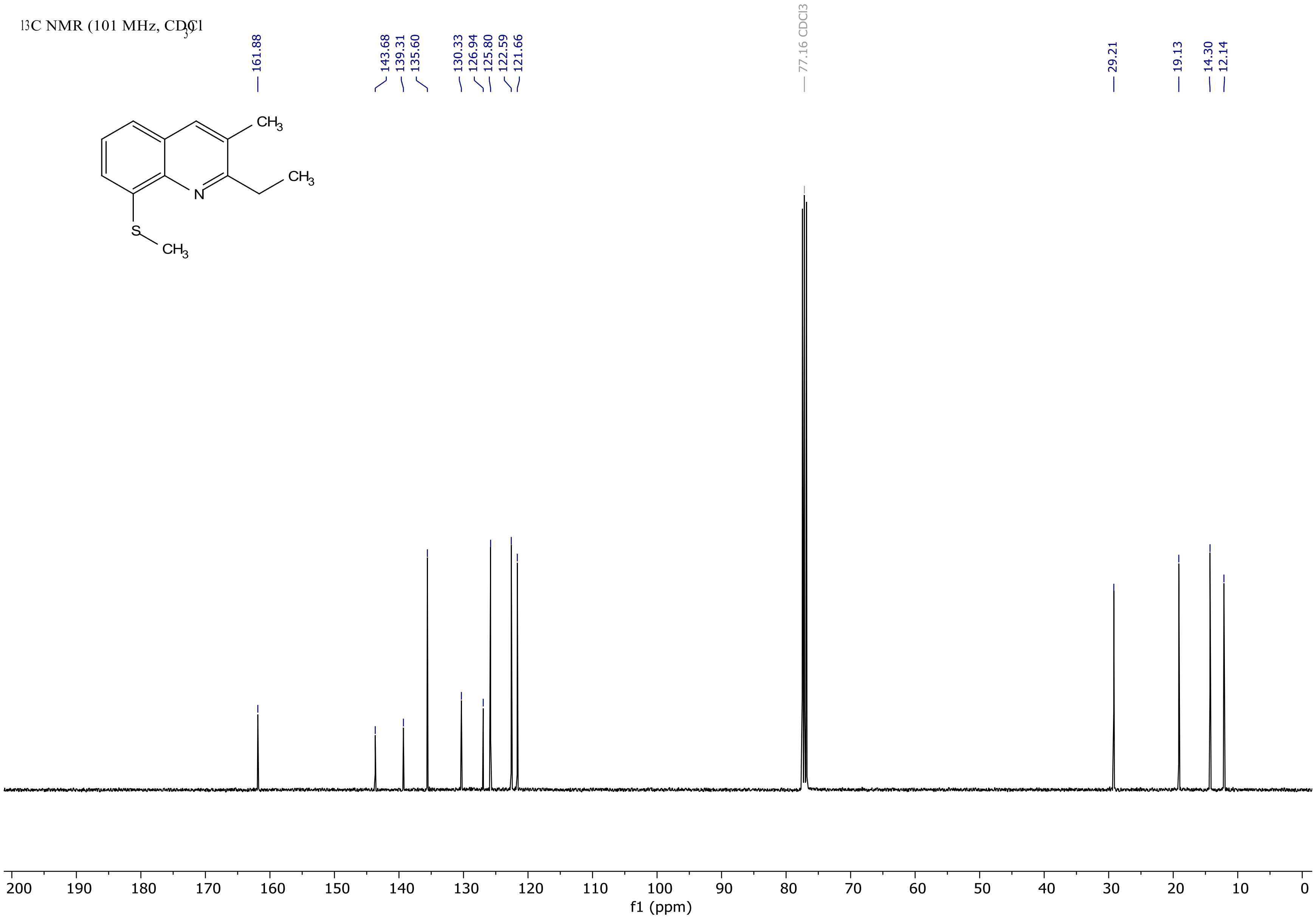

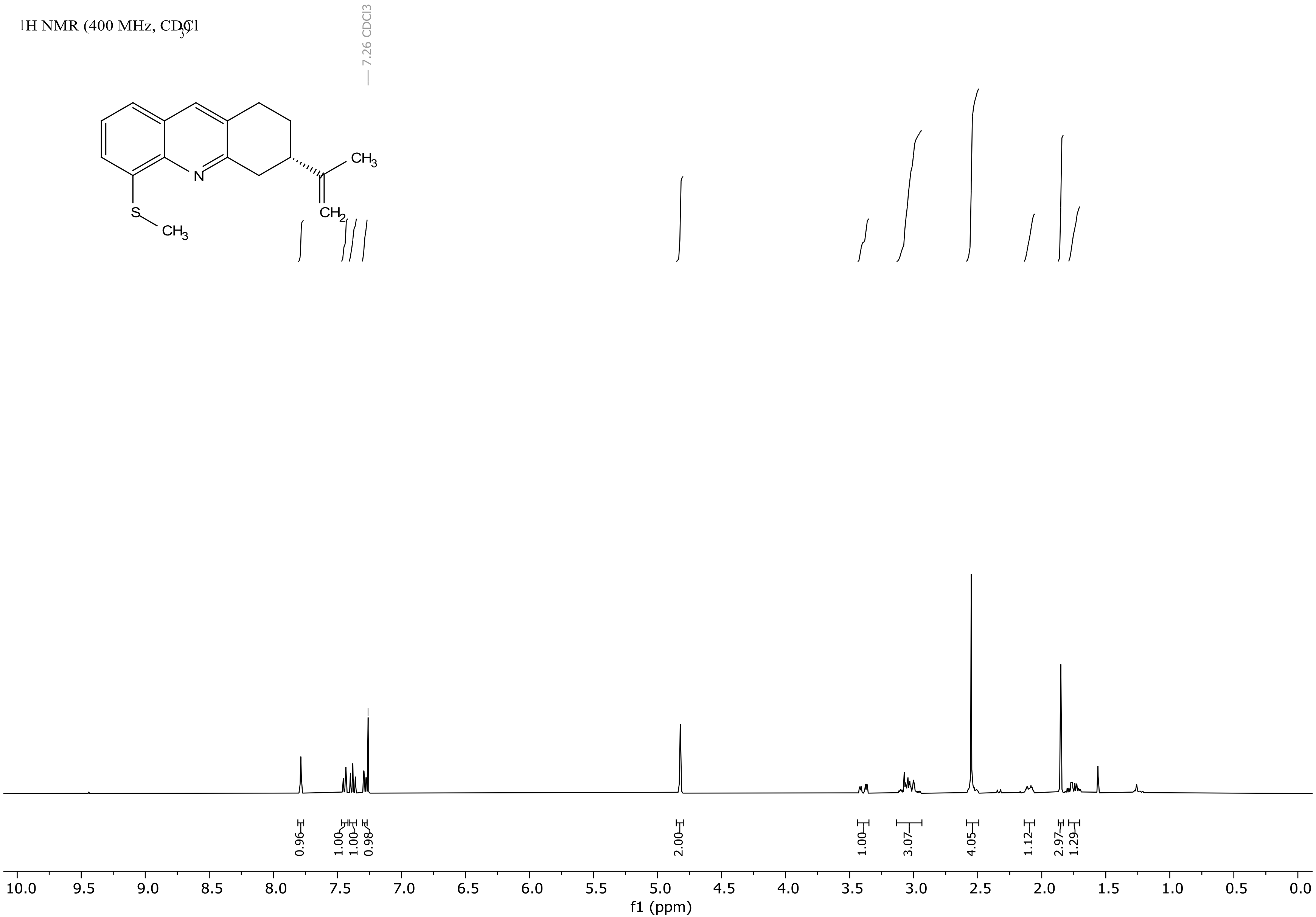
1H NMR (400 MHz, CDCl3)
7.26 CDCl3
CH3
N
S
CH3
CH2
0.96
1.00
1.00
0.98
2.00
1.00
3.07
4.05
1.12
2.97
1.29
10.0 9.5 9.0 8.5 8.0 7.5 7.0 6.5 6.0 5.5 5.0 4.5 4.0 3.5 3.0 2.5 2.0 1.5 1.0 0.5 0.0
f1 (ppm)

13C NMR (101 MHz, CDCl3)

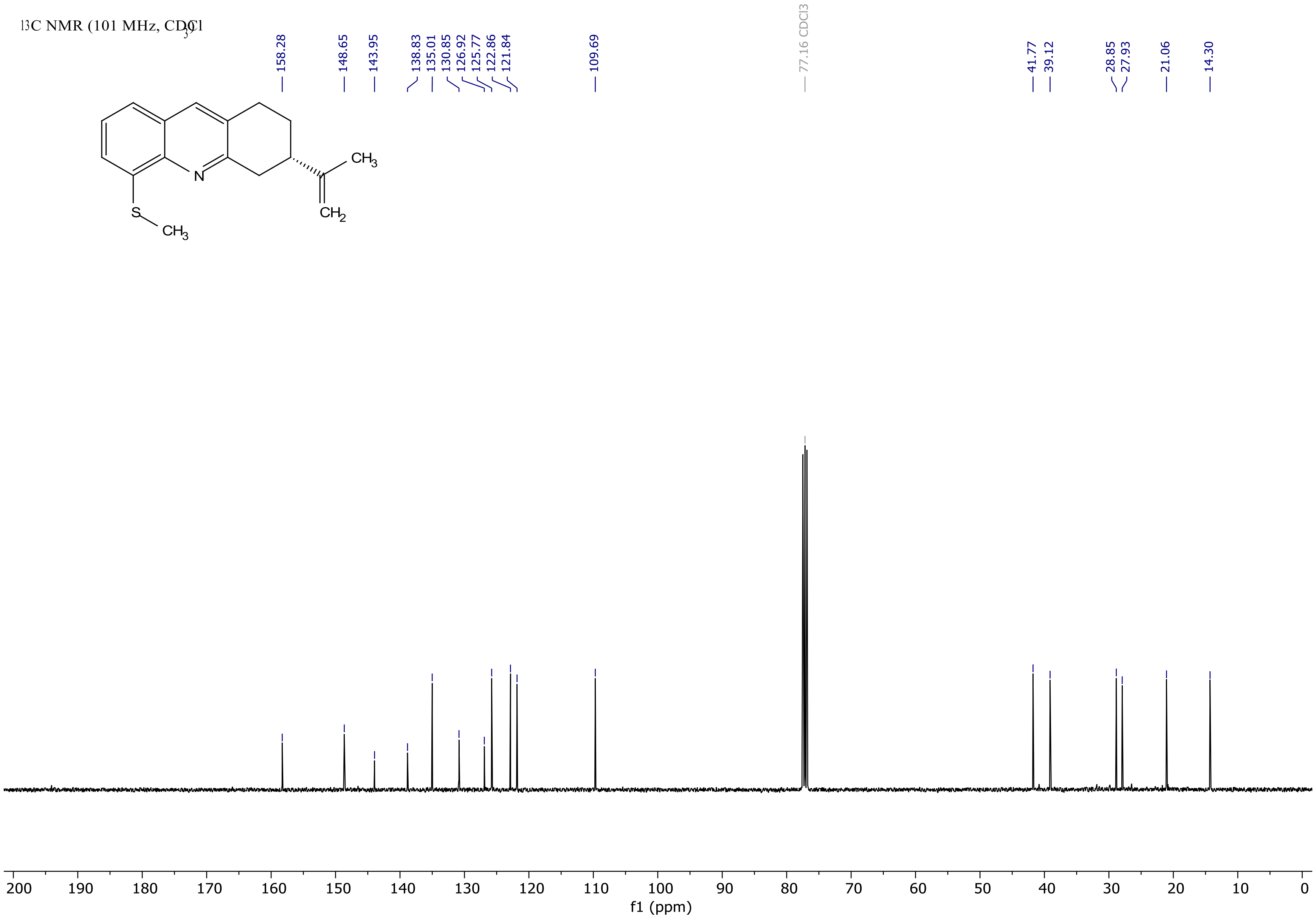

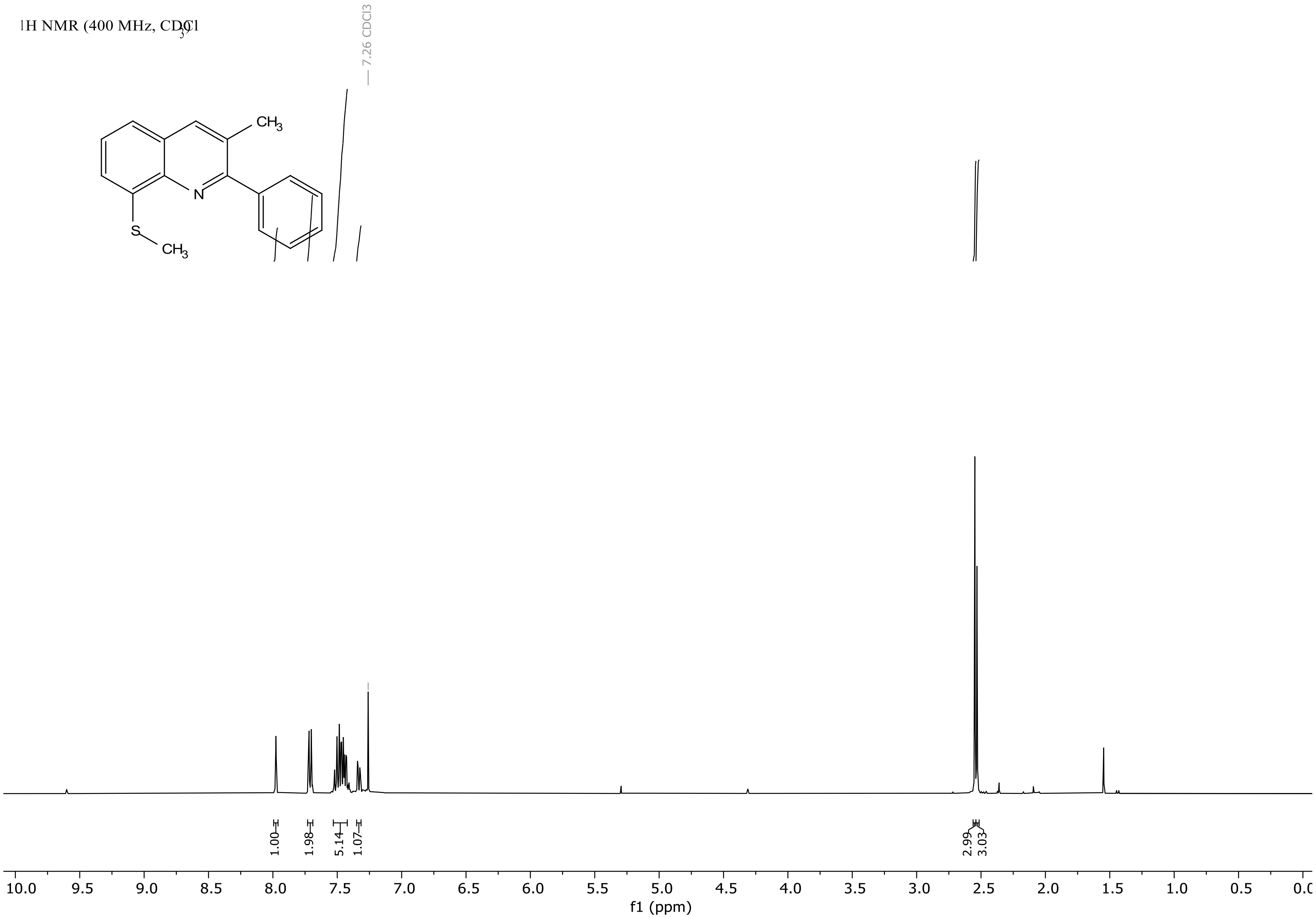
1H NMR (400 MHz, CDCl3)
7.26 CDCl3
CH3
N
S
CH3
1.00
1.98
5.14
1.07
2.99
3.03
10.0 9.5 9.0 8.5 8.0 7.5 7.0 6.5 6.0 5.5 5.0 4.5 4.0 3.5 3.0 2.5 2.0 1.5 1.0 0.5
f1 (ppm)

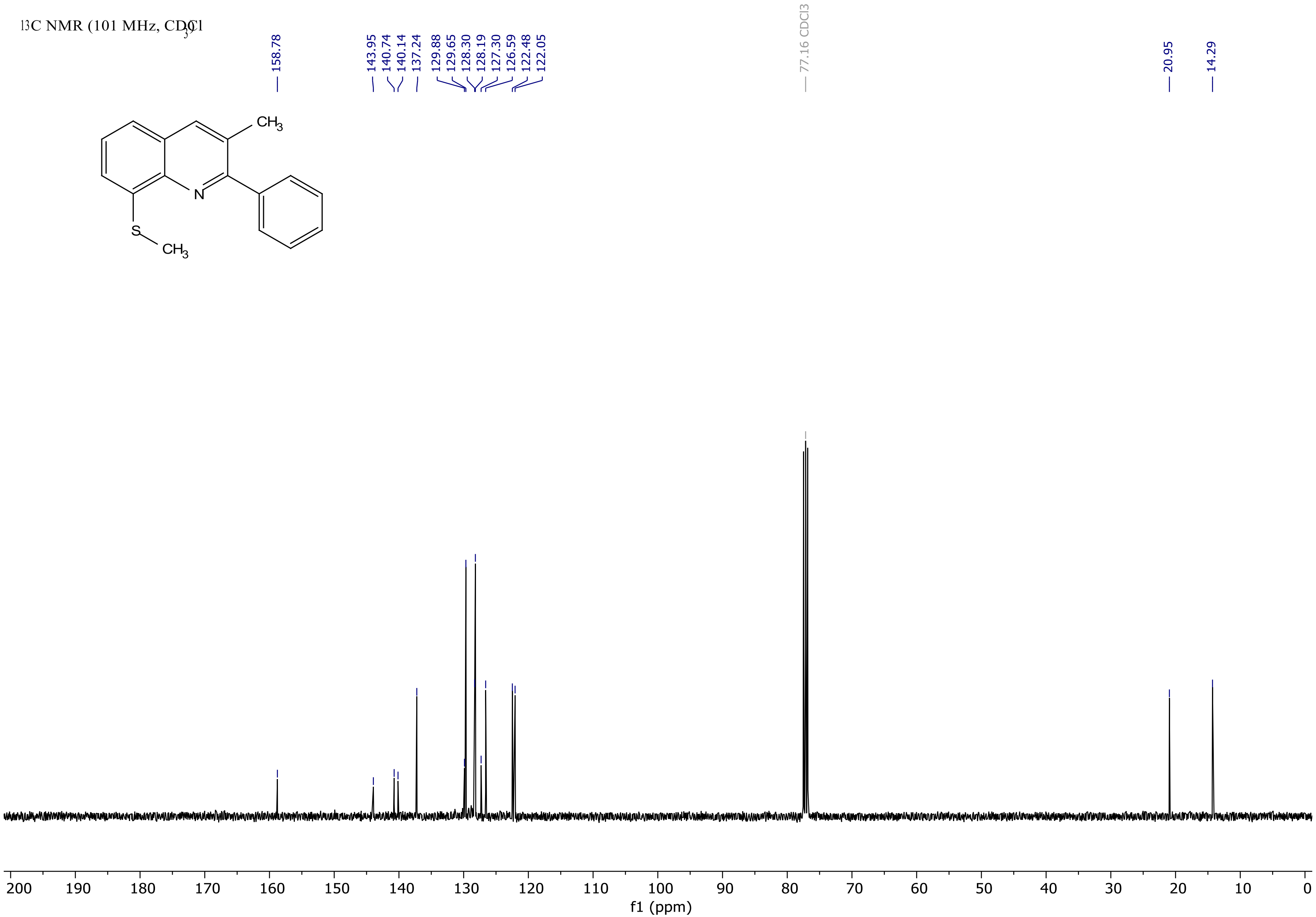
13C NMR (101 MHz, CDCl3)
CH3
N
S
CH3
158.78
143.95
140.74
140.14
137.24
129.88
129.65
128.30
128.19
127.30
126.59
122.48
122.05
77.16 CDCl3
20.95
14.29
200 190 180 170 160 150 140 130 120 110 100 90 80 70 60 50 40 30 20 10 0
f1 (ppm)

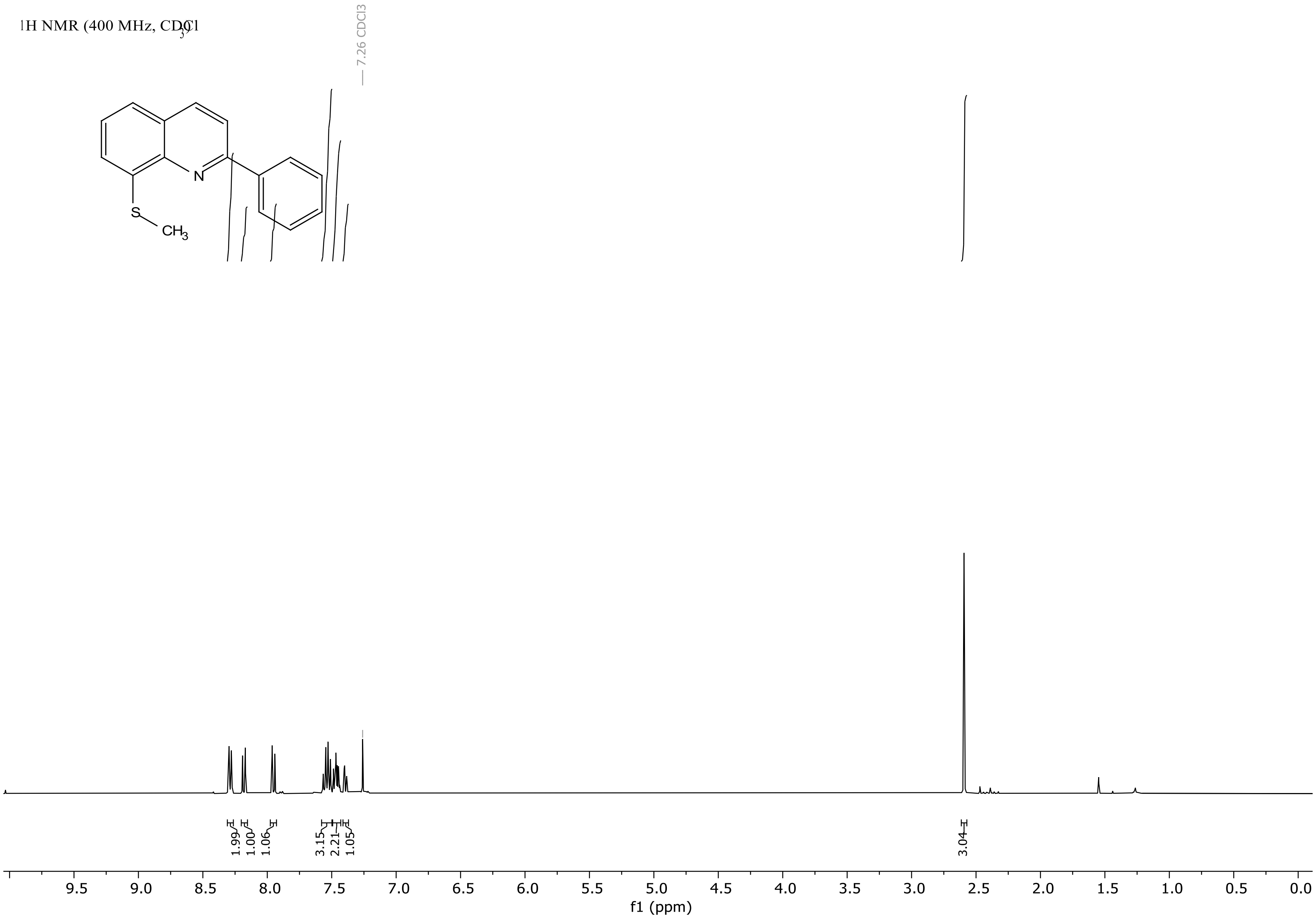
1H NMR (400 MHz, CDCl3)
7.26 CDCl3
N
S
CH3
1.99
1.00
1.06
3.15
2.21
1.05
3.04
9.5 9.0 8.5 8.0 7.5 7.0 6.5 6.0 5.5 5.0 4.5 4.0 3.5 3.0 2.5 2.0 1.5 1.0 0.5 0.0
f1 (ppm)

13C NMR (101 MHz, CDCl3)

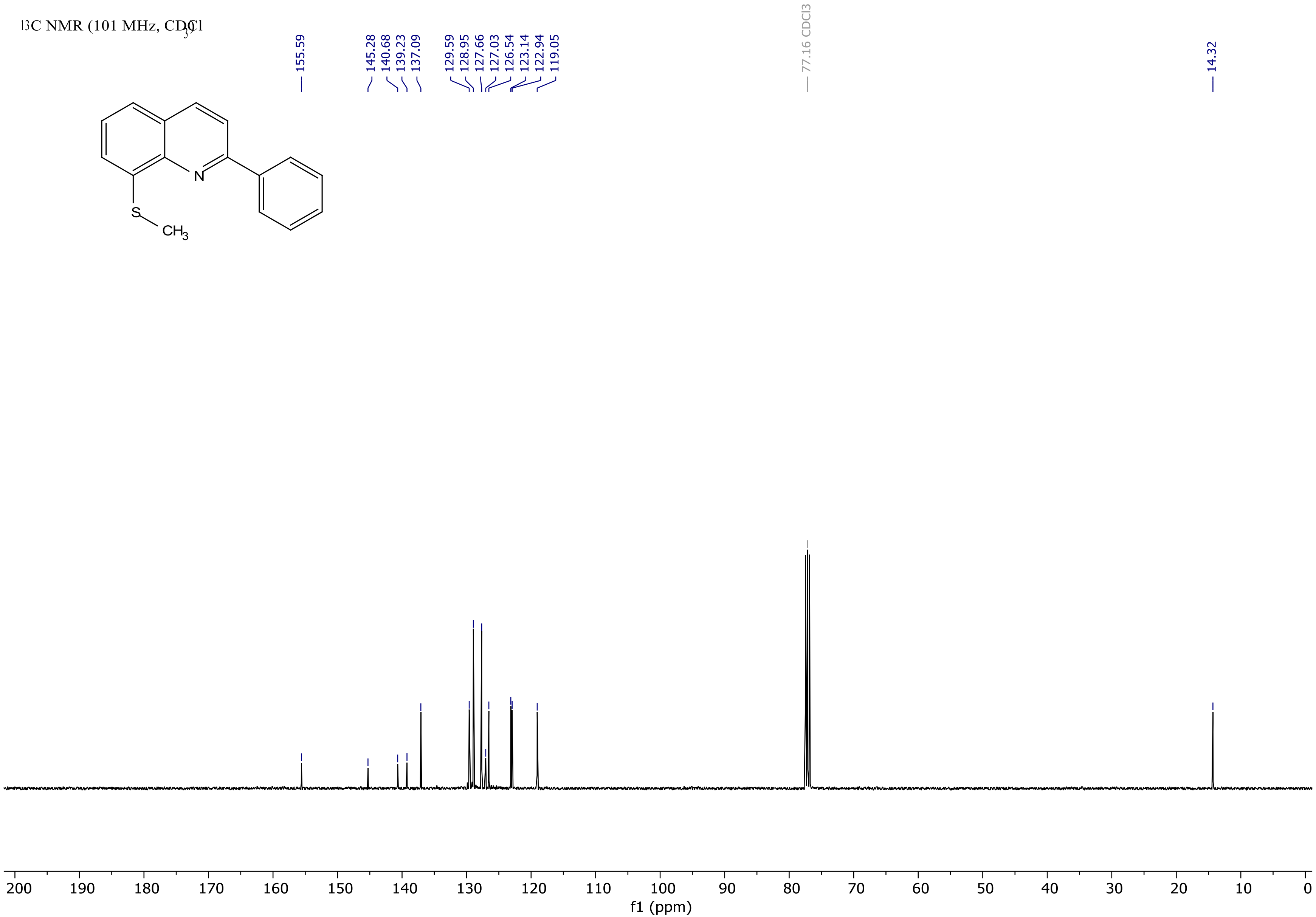

1H NMR (400 MHz, CDCl3)

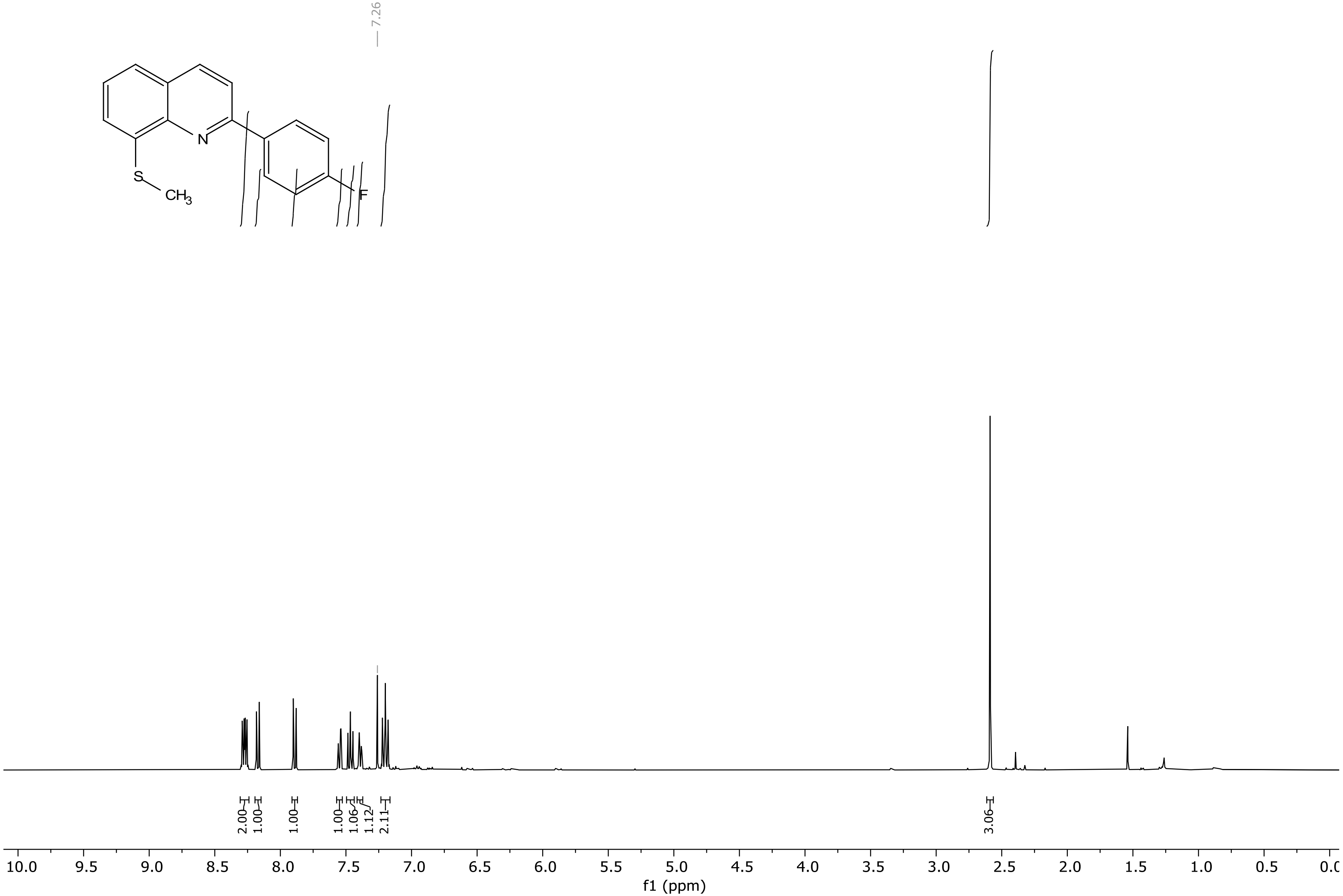

13C NMR (101 MHz, CDCl3)

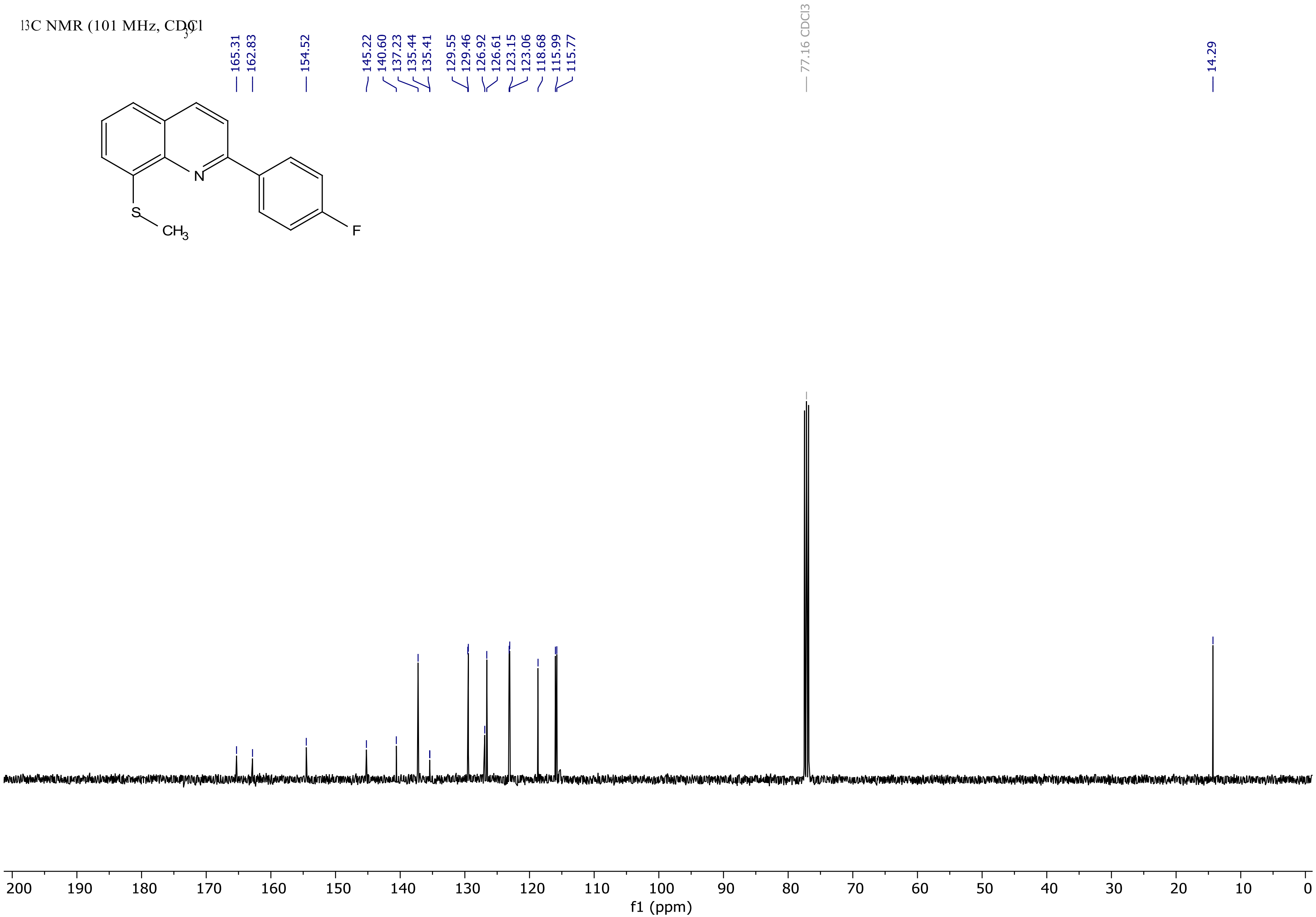

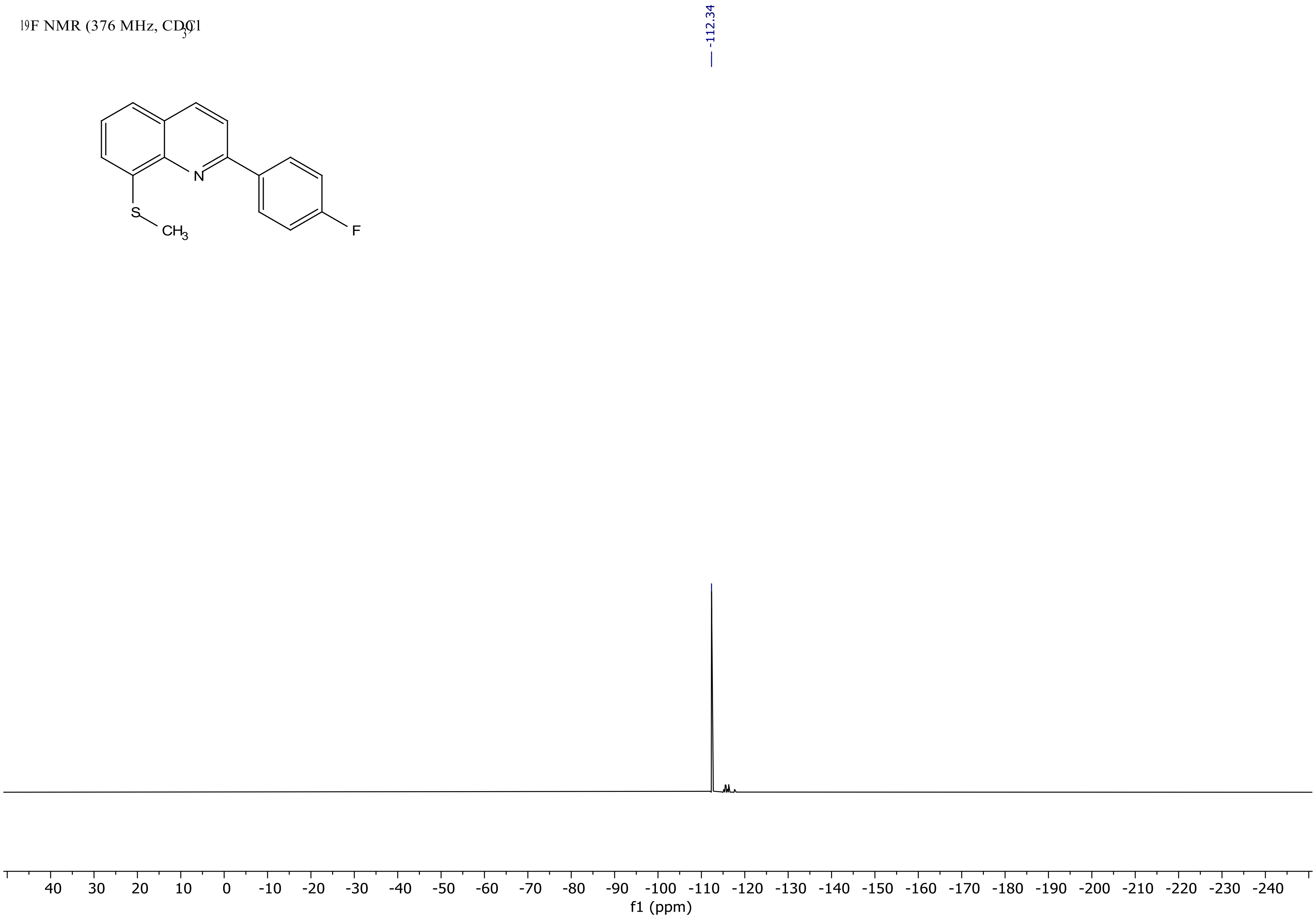
19F NMR (376 MHz, CDCl3)
-112.34
S
CH3
N
F
40 30 20 10 0 -10 -20 -30 -40 -50 -60 -70 -80 -90 -100 -110 -120 -130 -140 -150 -160 -170 -180 -190 -200 -210 -220 -230 -240
f1 (ppm)

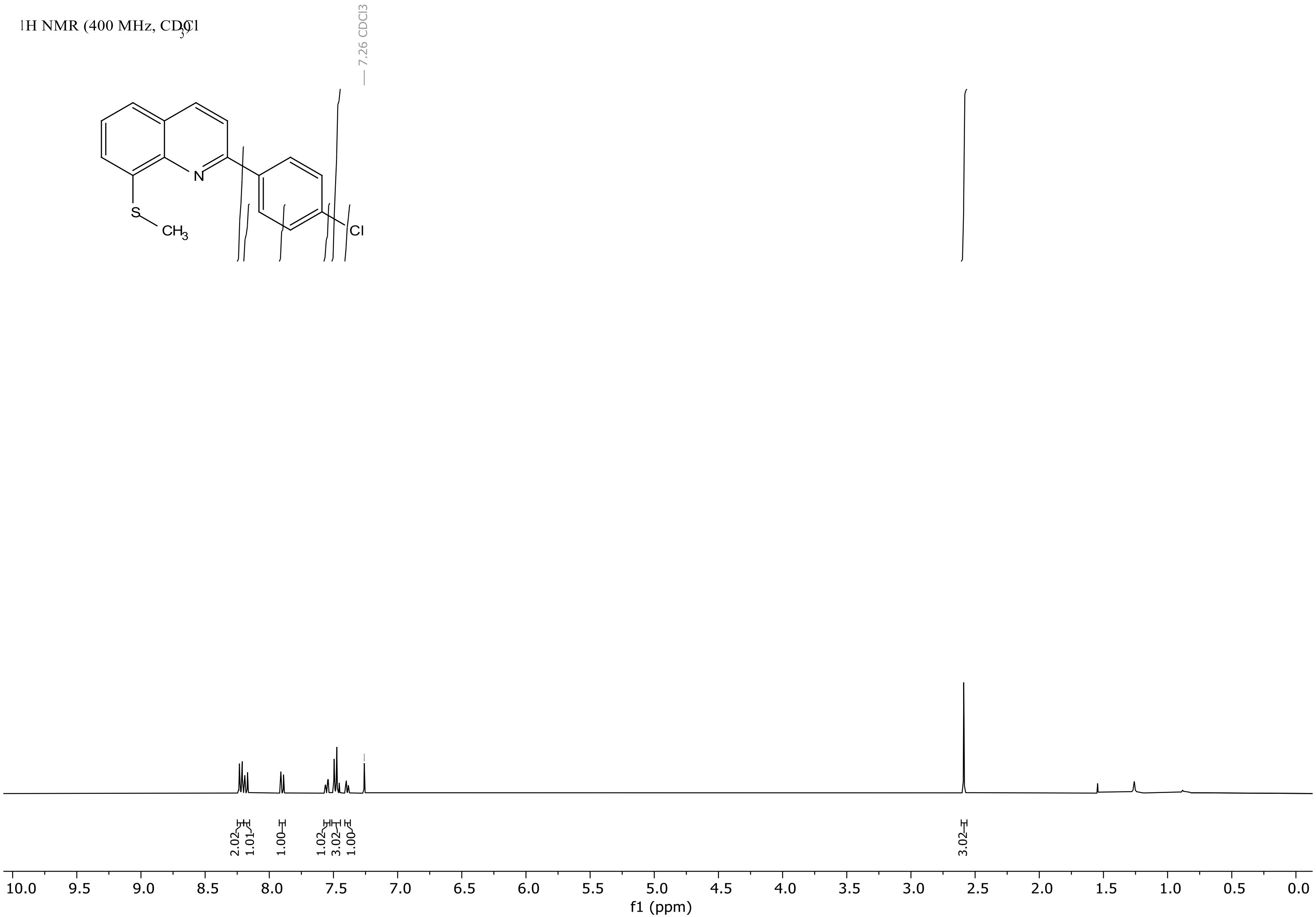
1H NMR (400 MHz, CDCl3)
7.26 CDCl3
N
S
CH3
Cl
2.02
1.01
1.00
1.02
3.02
1.00
3.02
10.0
9.5
9.0
8.5
8.0
7.5
7.0
6.5
6.0
5.5
5.0
4.5
4.0
3.5
3.0
2.5
2.0
1.5
1.0
0.5
0.0
f1 (ppm)

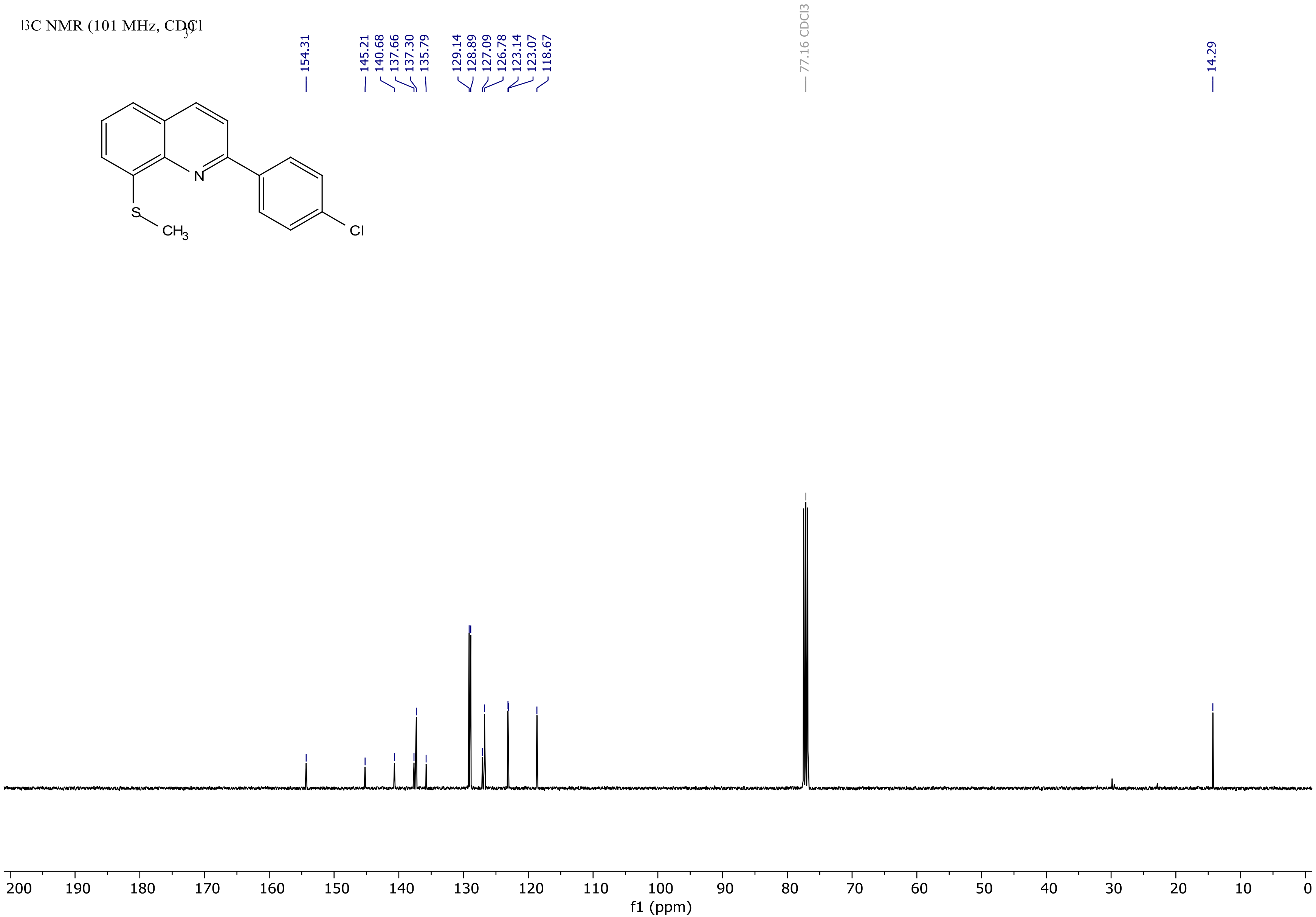
13C NMR (101 MHz, CDCl3)
154.31
145.21
140.68
137.66
137.30
135.79
129.14
128.89
127.09
126.78
123.14
123.07
118.67
77.16 CDCl3
14.29
N
S
CH3
Cl
200
190
180
170
160
150
140
130
120
110
100
90
80
70
60
50
40
30
20
10
0
f1 (ppm)

1H NMR (400 MHz, CDCl3)

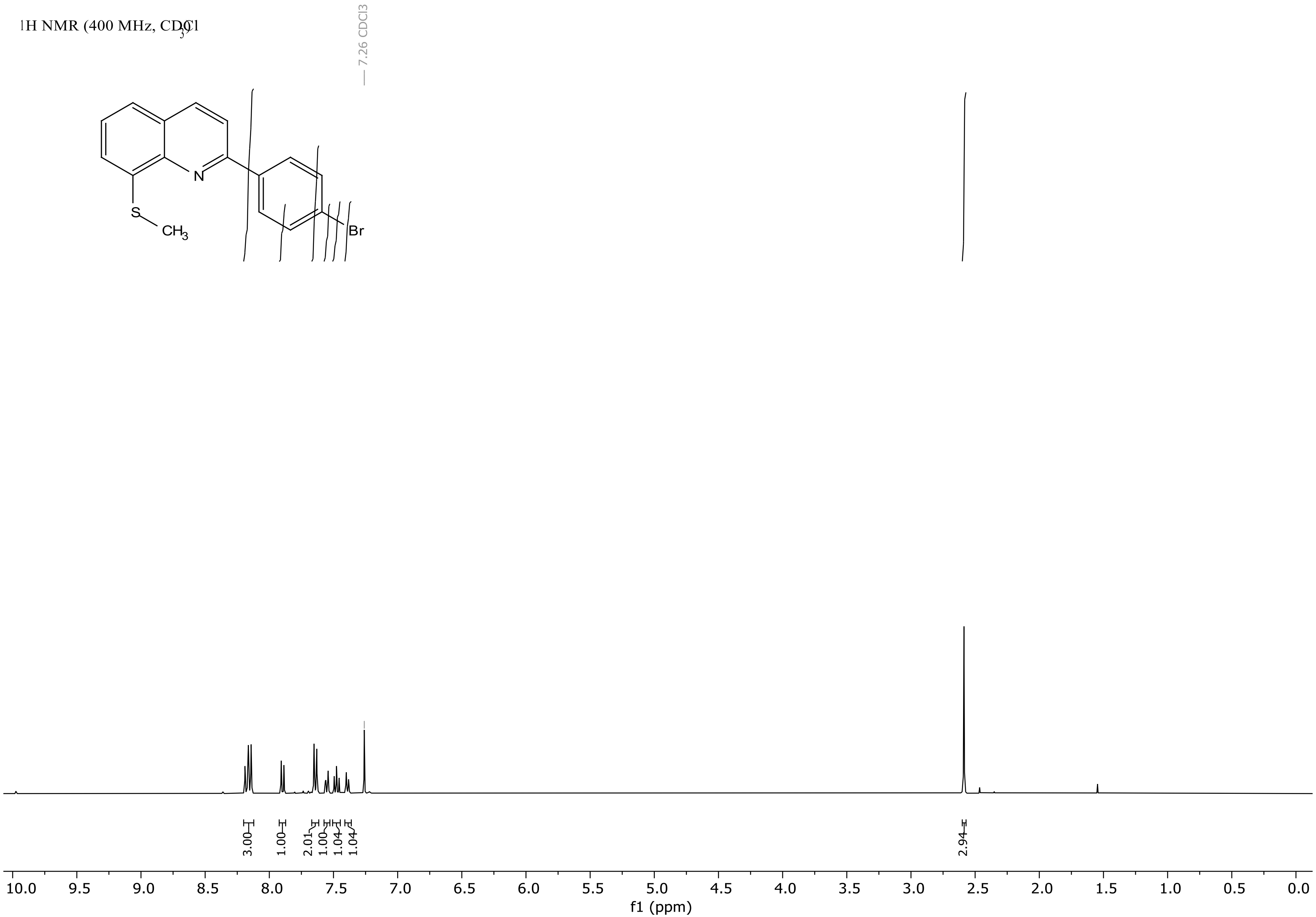

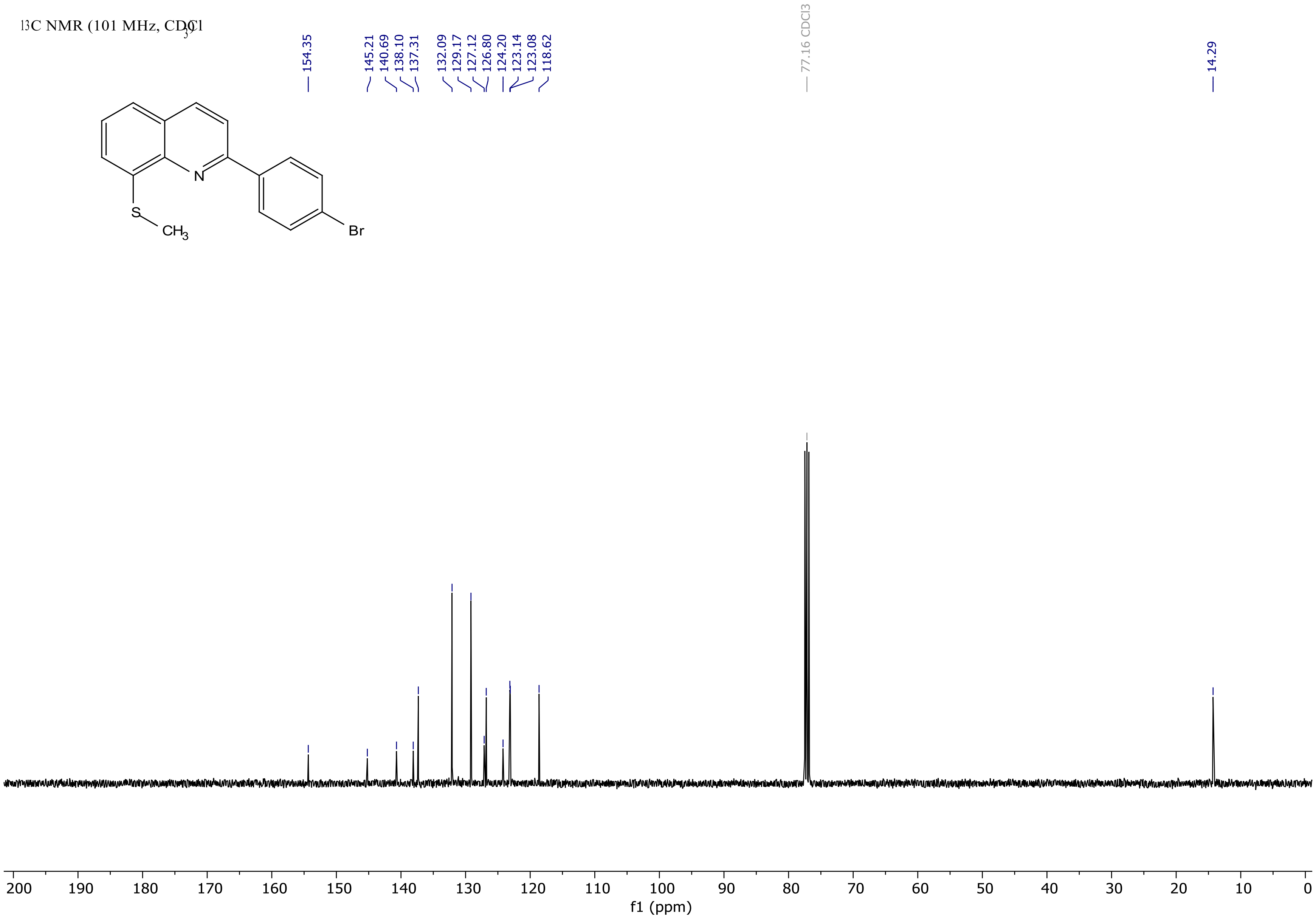
13C NMR (101 MHz, CDCl3)
154.35
145.21
140.69
138.10
137.31
132.09
129.17
127.12
126.80
124.20
123.14
123.08
118.62
77.16 CDCl3
14.29
N
S
CH3
Br
200 190 180 170 160 150 140 130 120 110 100 90 80 70 60 50 40 30 20 10 0
f1 (ppm)

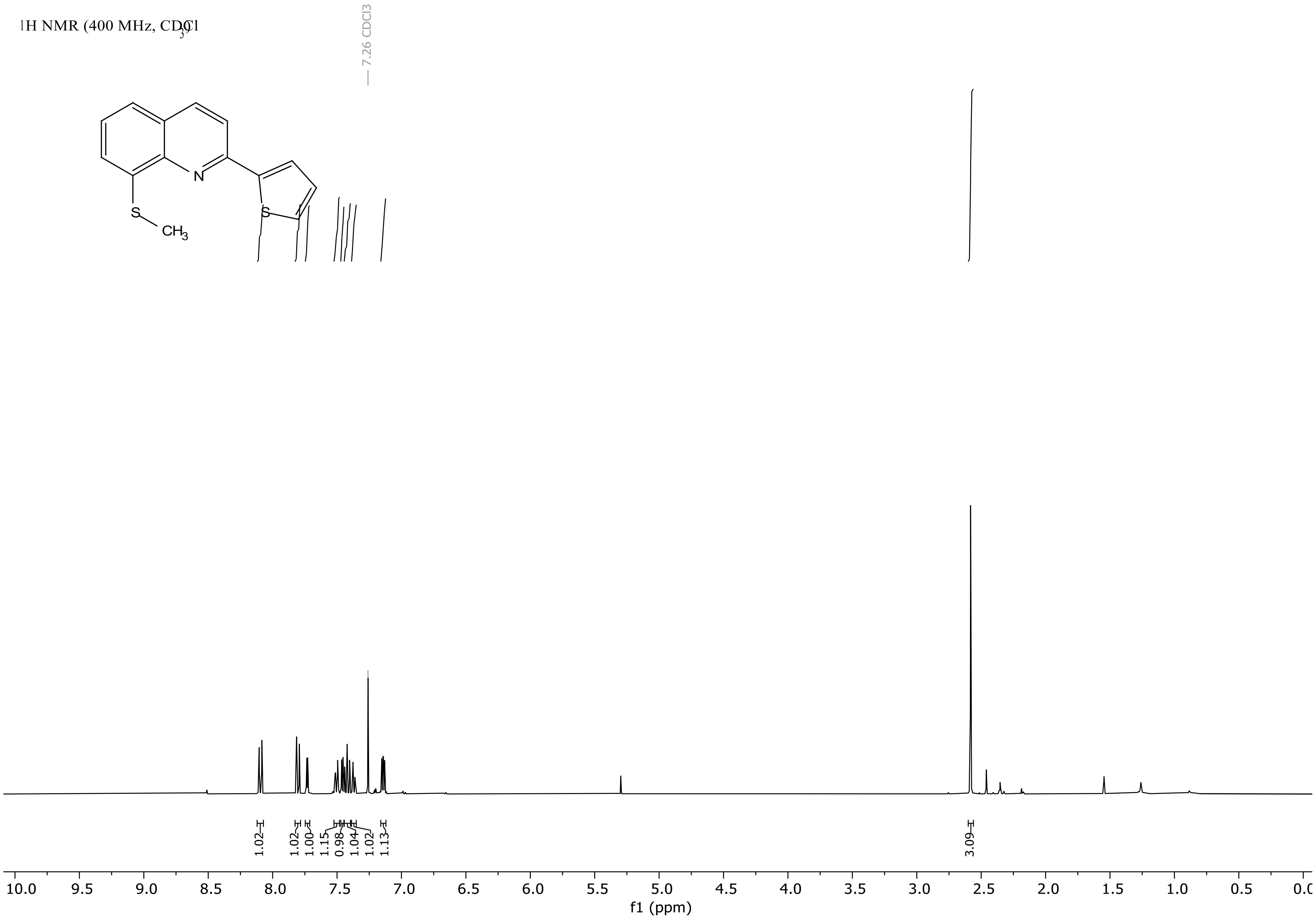
1H NMR (400 MHz, CDCl3)
7.26 CDCl3
N
S
S
CH3
1.02
1.02
1.00
1.15
0.98
1.04
1.02
1.13
3.09
10.0
9.5
9.0
8.5
8.0
7.5
7.0
6.5
6.0
5.5
5.0
4.5
4.0
3.5
3.0
2.5
2.0
1.5
1.0
0.5
f1 (ppm)

13C NMR (101 MHz, $CDCl_3$)

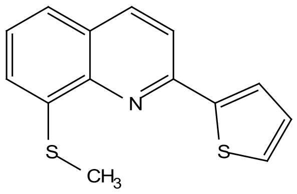

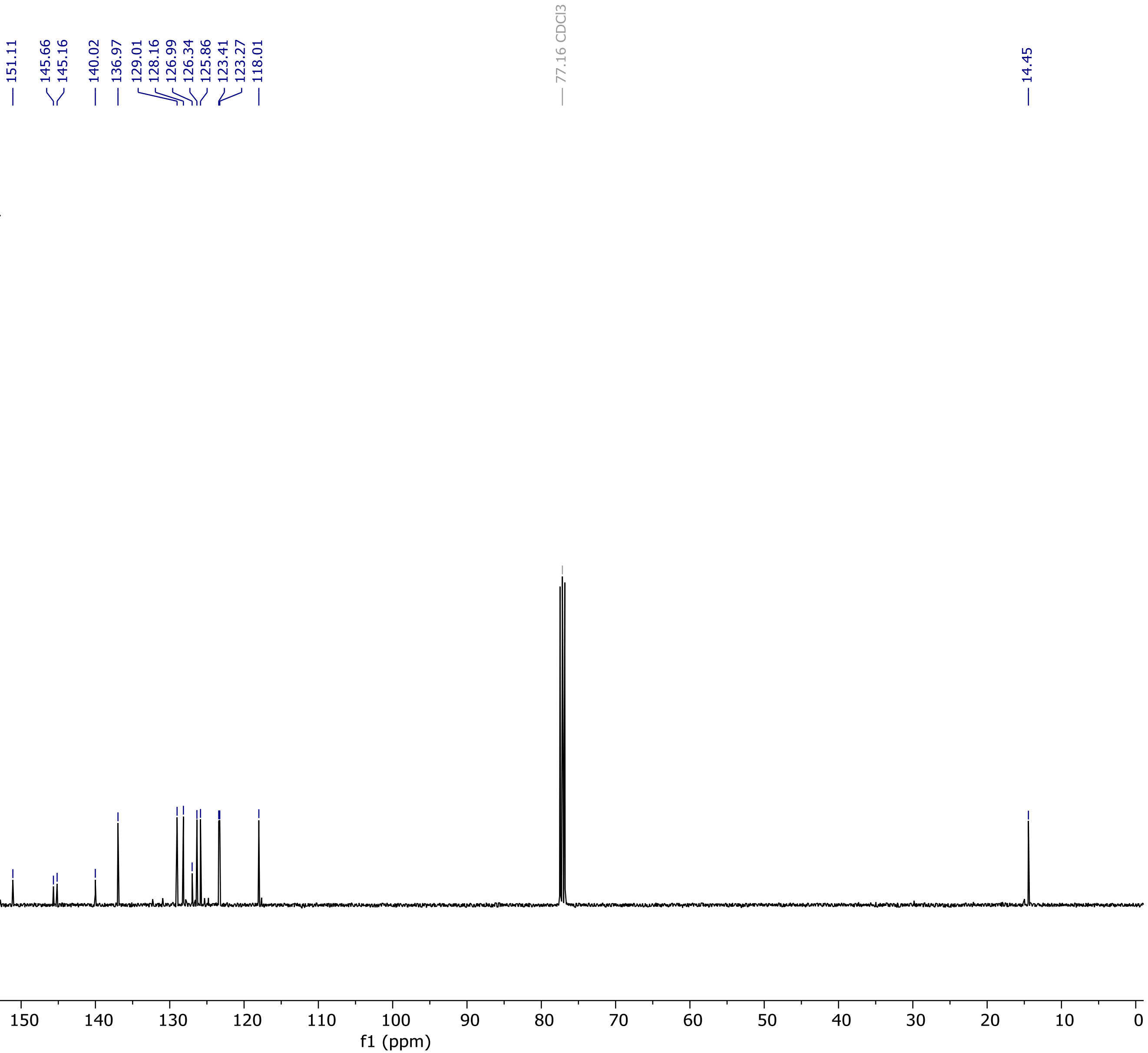

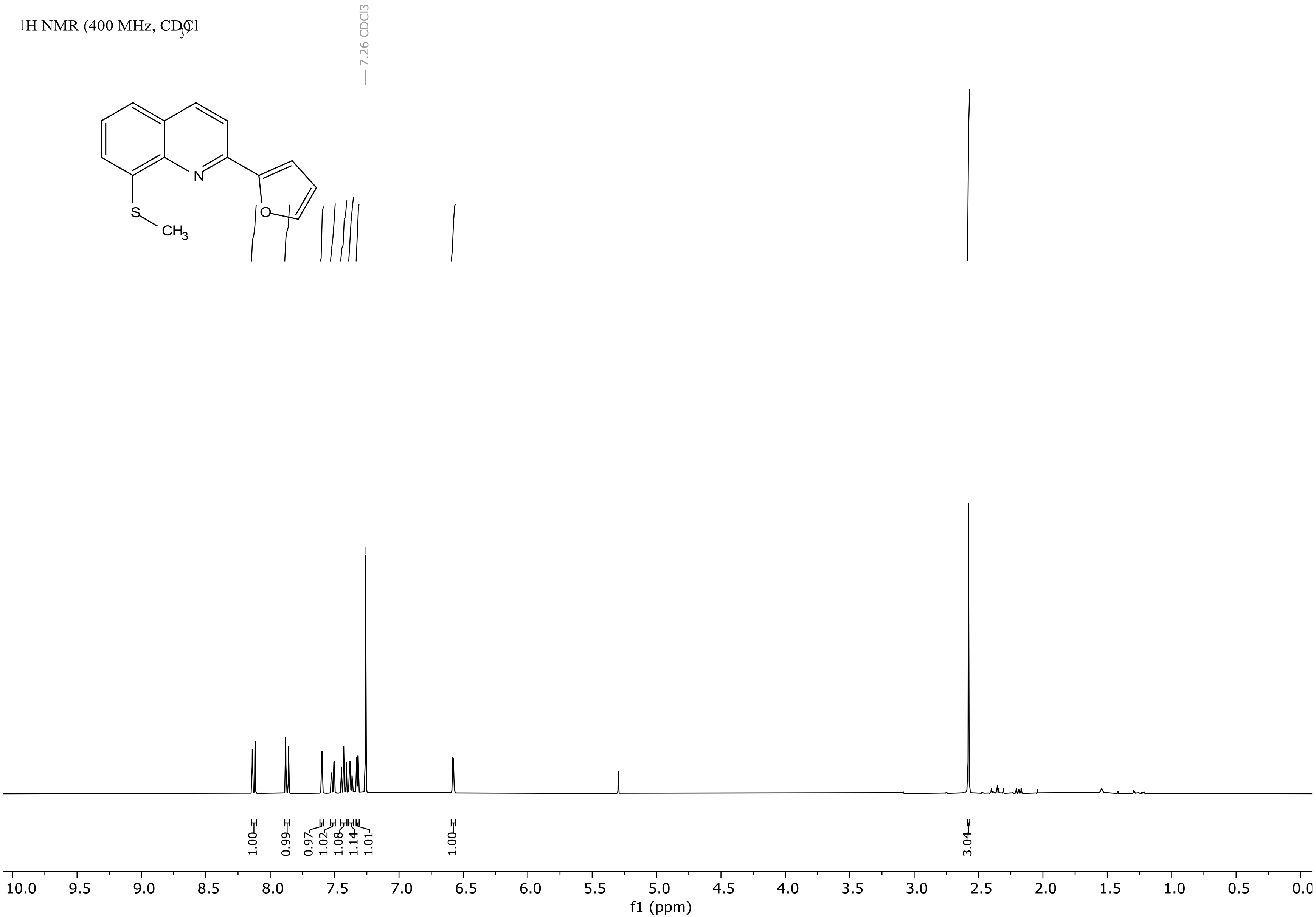
1H NMR (400 MHz, CDCl3)
7.26 CDCl3
N
O
S
CH3
1.00
0.99
0.97
1.02
1.08
1.14
1.01
1.00
3.04
10.0
9.5
9.0
8.5
8.0
7.5
7.0
6.5
6.0
5.5
5.0
4.5
4.0
3.5
3.0
2.5
2.0
1.5
1.0
0.5
0.0
f1 (ppm)

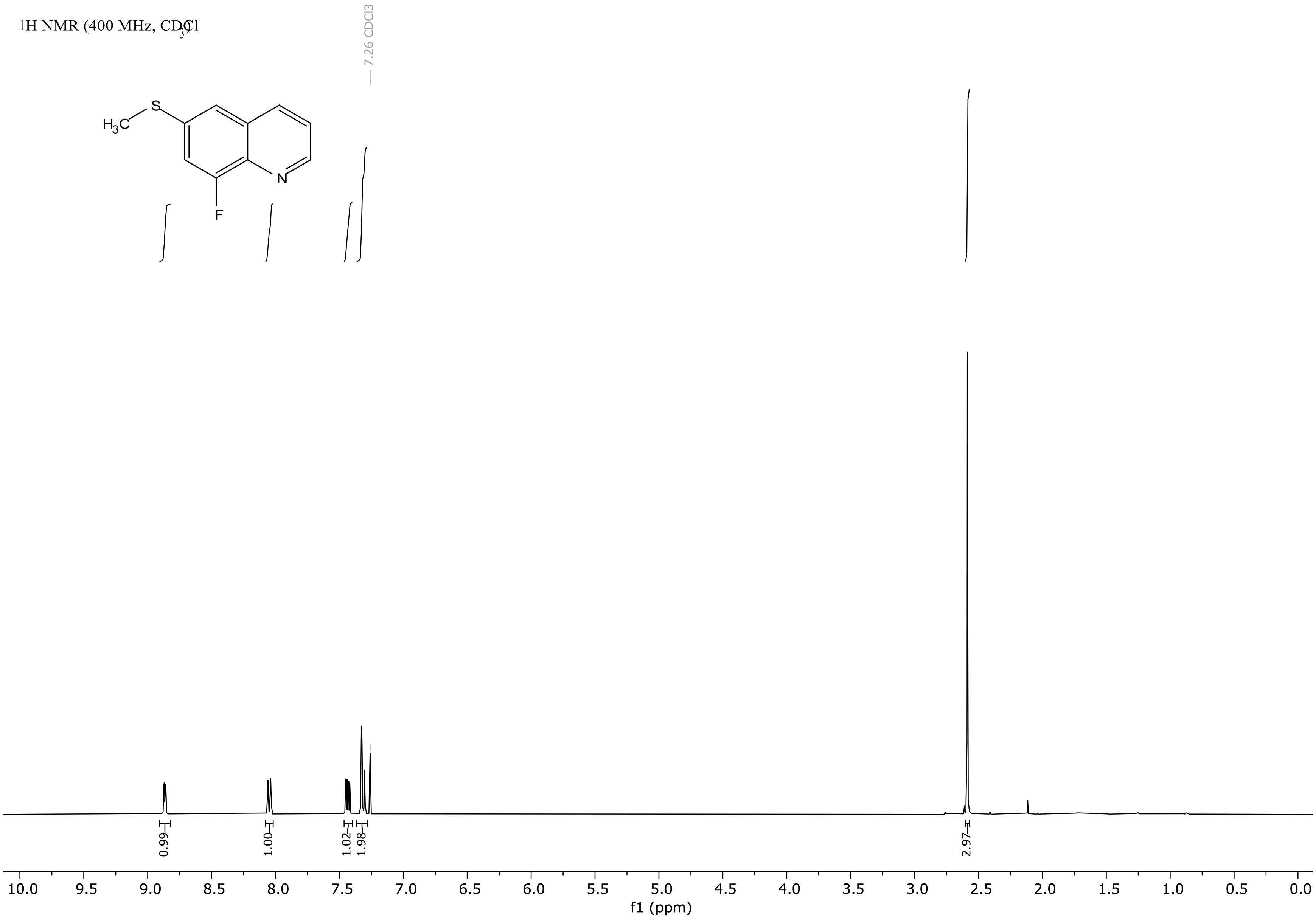
1H NMR (400 MHz, CDCl3)
7.26 CDCl3
H3C
S
N
F
0.99
1.00
1.02
1.98
2.97
10.0 9.5 9.0 8.5 8.0 7.5 7.0 6.5 6.0 5.5 5.0 4.5 4.0 3.5 3.0 2.5 2.0 1.5 1.0 0.5 0.0
f1 (ppm)

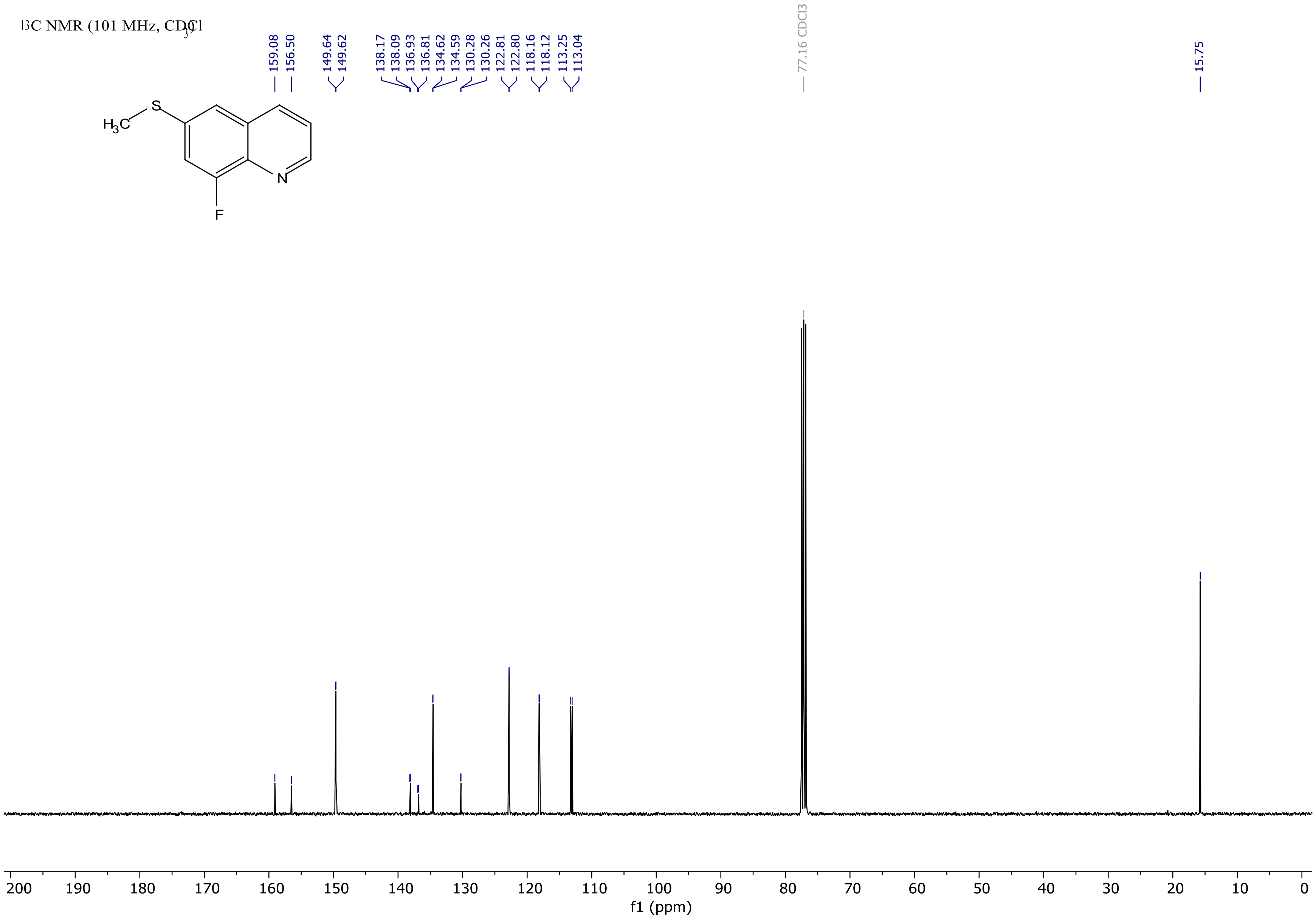
13C NMR (101 MHz, CDCl3)
159.08
156.50
149.64
149.62
138.17
138.09
136.93
136.81
134.62
134.59
130.28
130.26
122.81
122.80
118.16
118.12
113.25
113.04
77.16 CDCl3
15.75
S
H3C
N
F
200 190 180 170 160 150 140 130 120 110 100 90 80 70 60 50 40 30 20 10 0
f1 (ppm)

19F NMR (376 MHz, CDCl3)

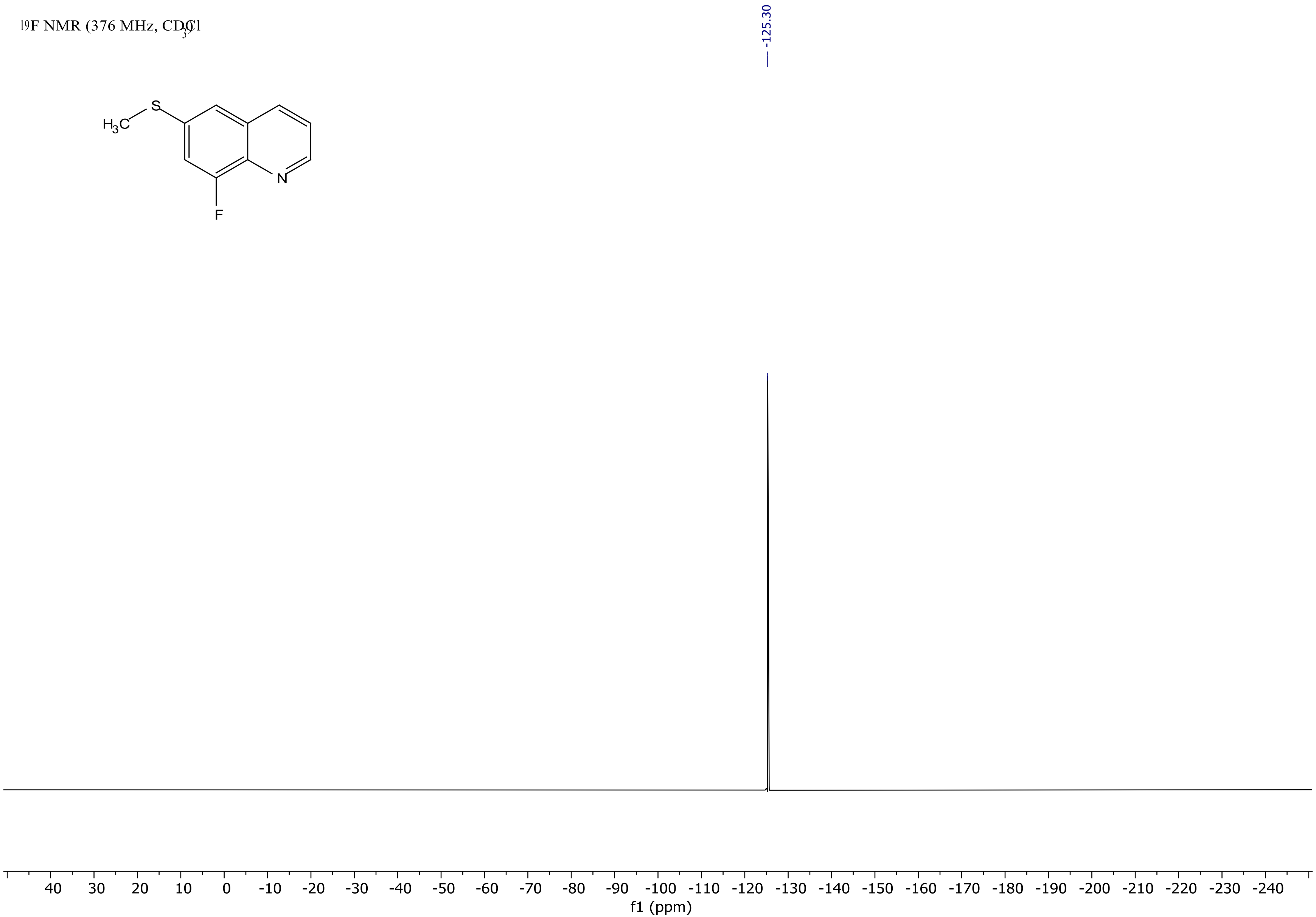

1H NMR (400 MHz, CDCl3)

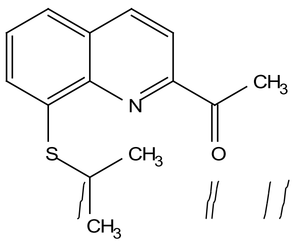

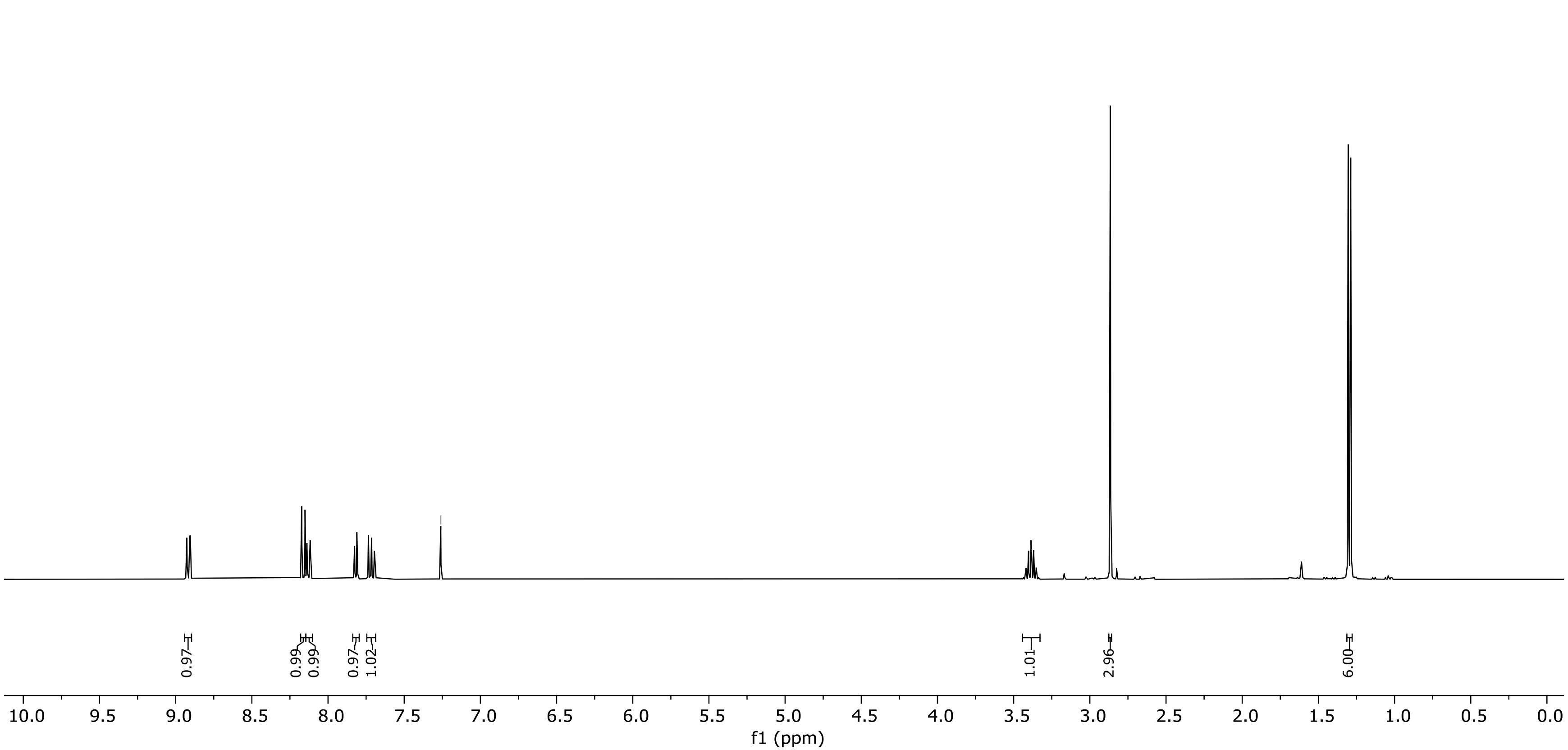

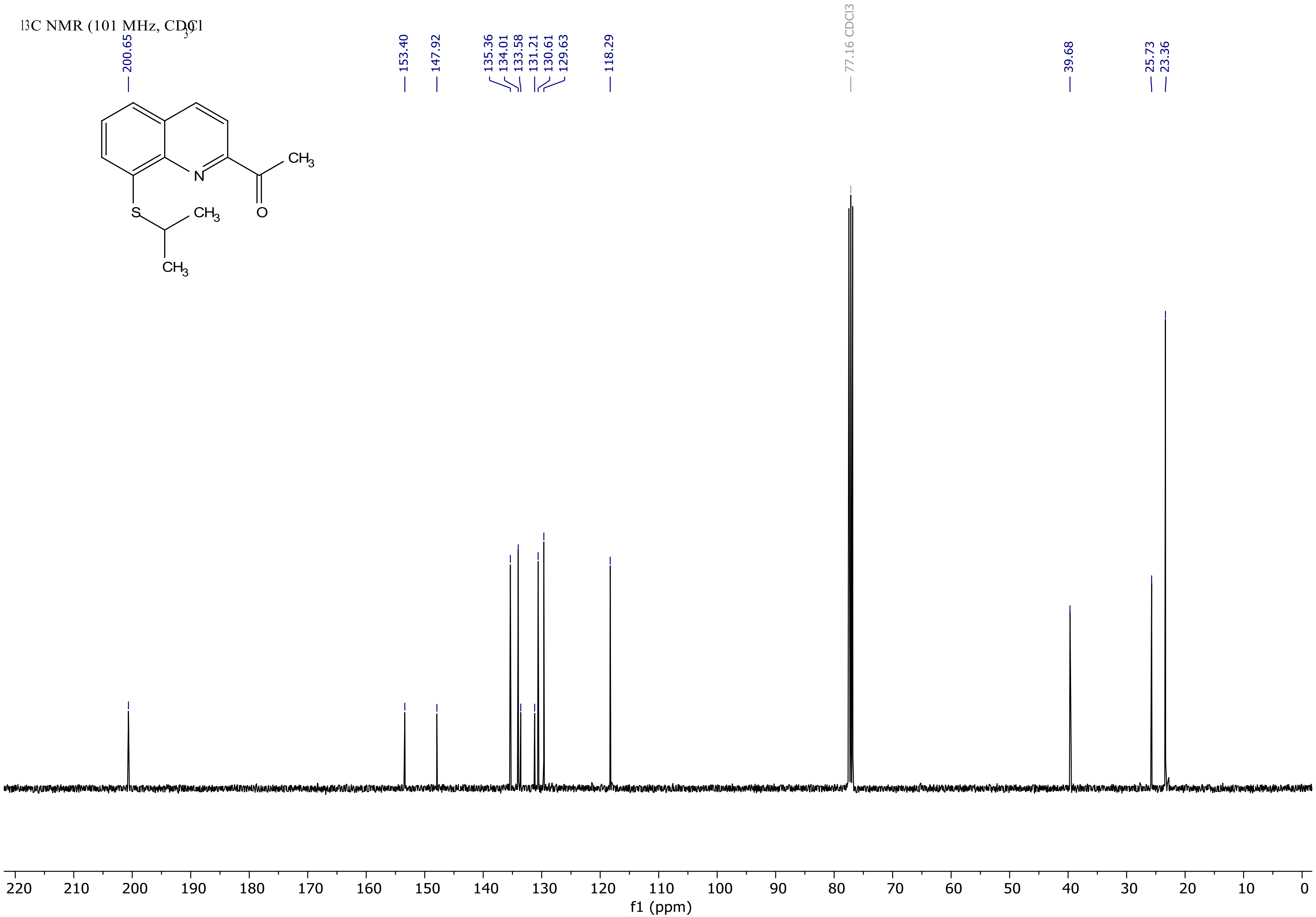
13C NMR (101 MHz, CDCl3)
200.65
153.40
147.92
135.36
134.01
133.58
131.21
130.61
129.63
118.29
77.16 CDCl3
39.68
25.73
23.36
CH3
N
S
CH3
O
CH3
220 210 200 190 180 170 160 150 140 130 120 110 100 90 80 70 60 50 40 30 20 10 0
f1 (ppm)

1H NMR (400 MHz, CDCl3)

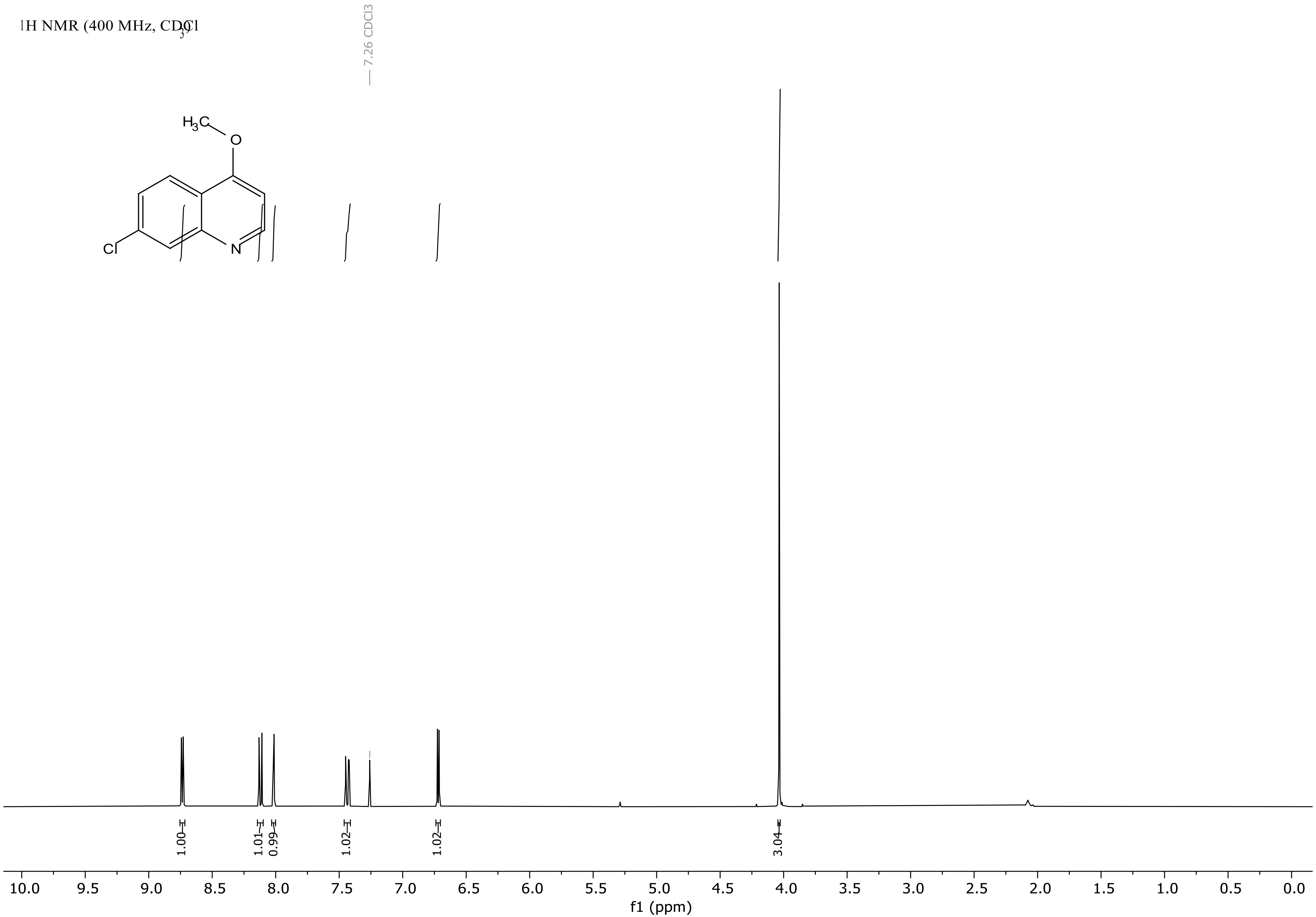

13C NMR (101 MHz, $CDCl_3$)

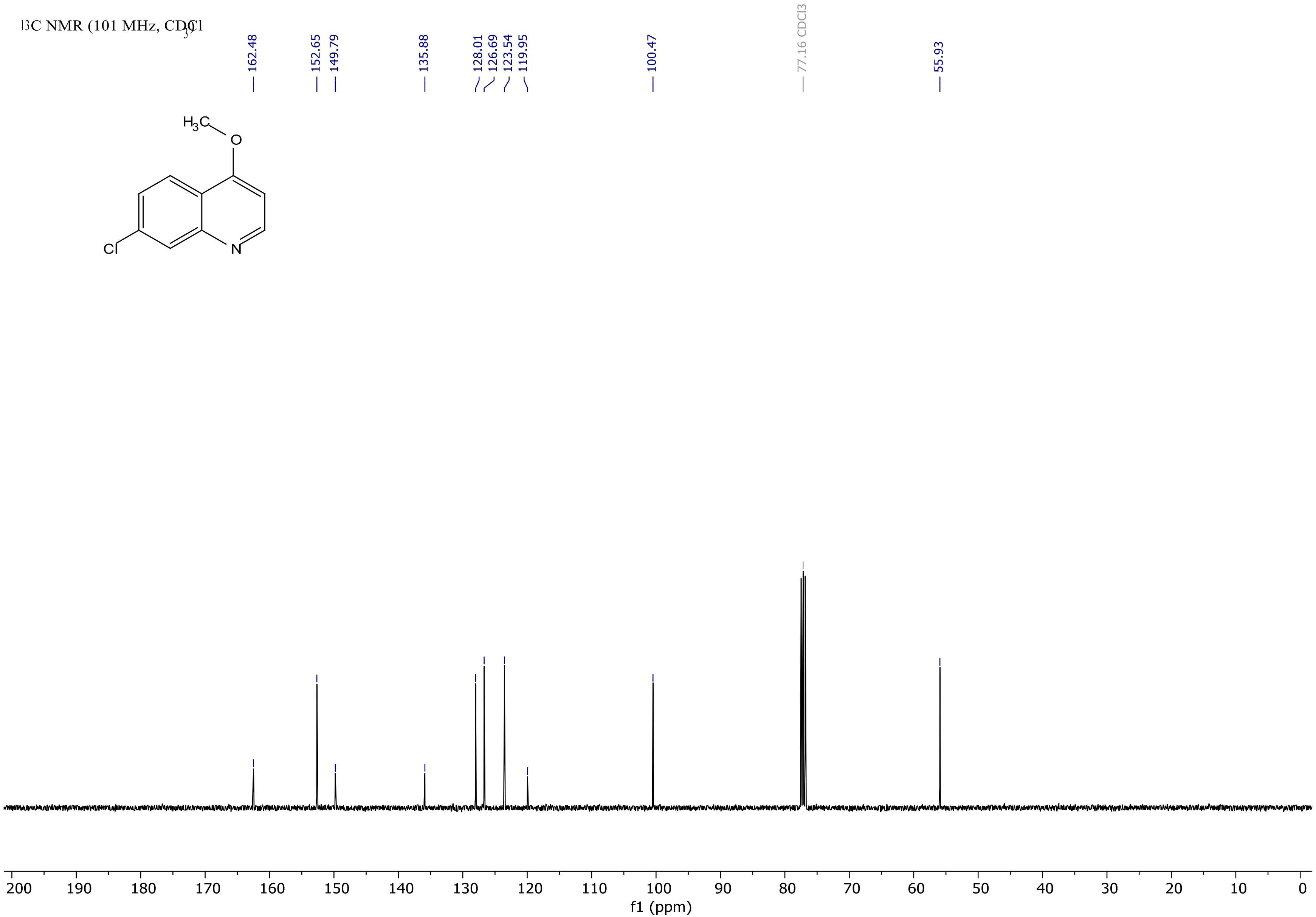

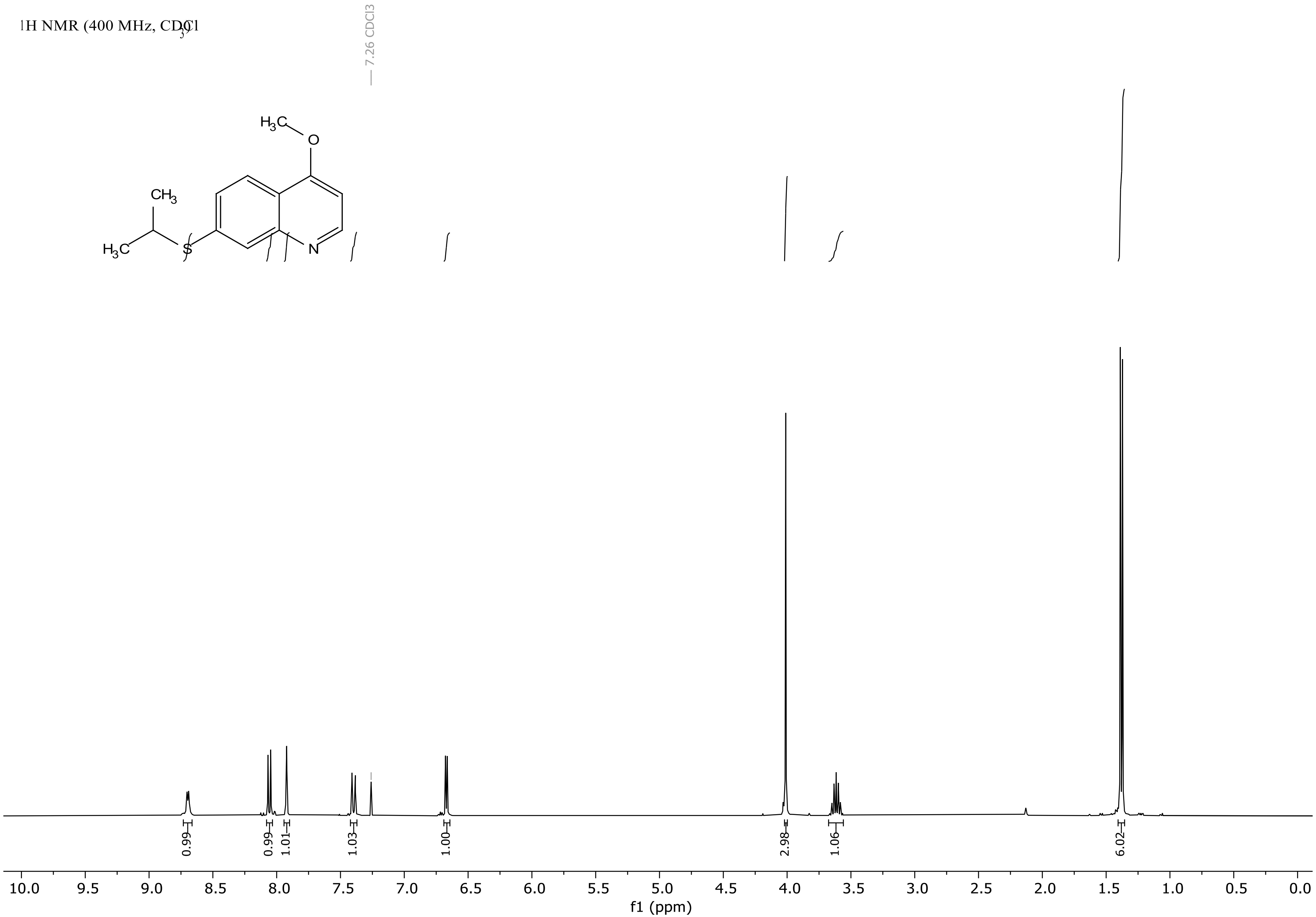
1H NMR (400 MHz, CDCl3)
7.26 CDCl3
H3C
O
CH3
H3C
S
N
0.99
0.99
1.01
1.03
1.00
2.98
1.06
6.02
10.0
9.5
9.0
8.5
8.0
7.5
7.0
6.5
6.0
5.5
5.0
4.5
4.0
3.5
3.0
2.5
2.0
1.5
1.0
0.5
0.0
f1 (ppm)

13C NMR (101 MHz, $CDCl_3$)

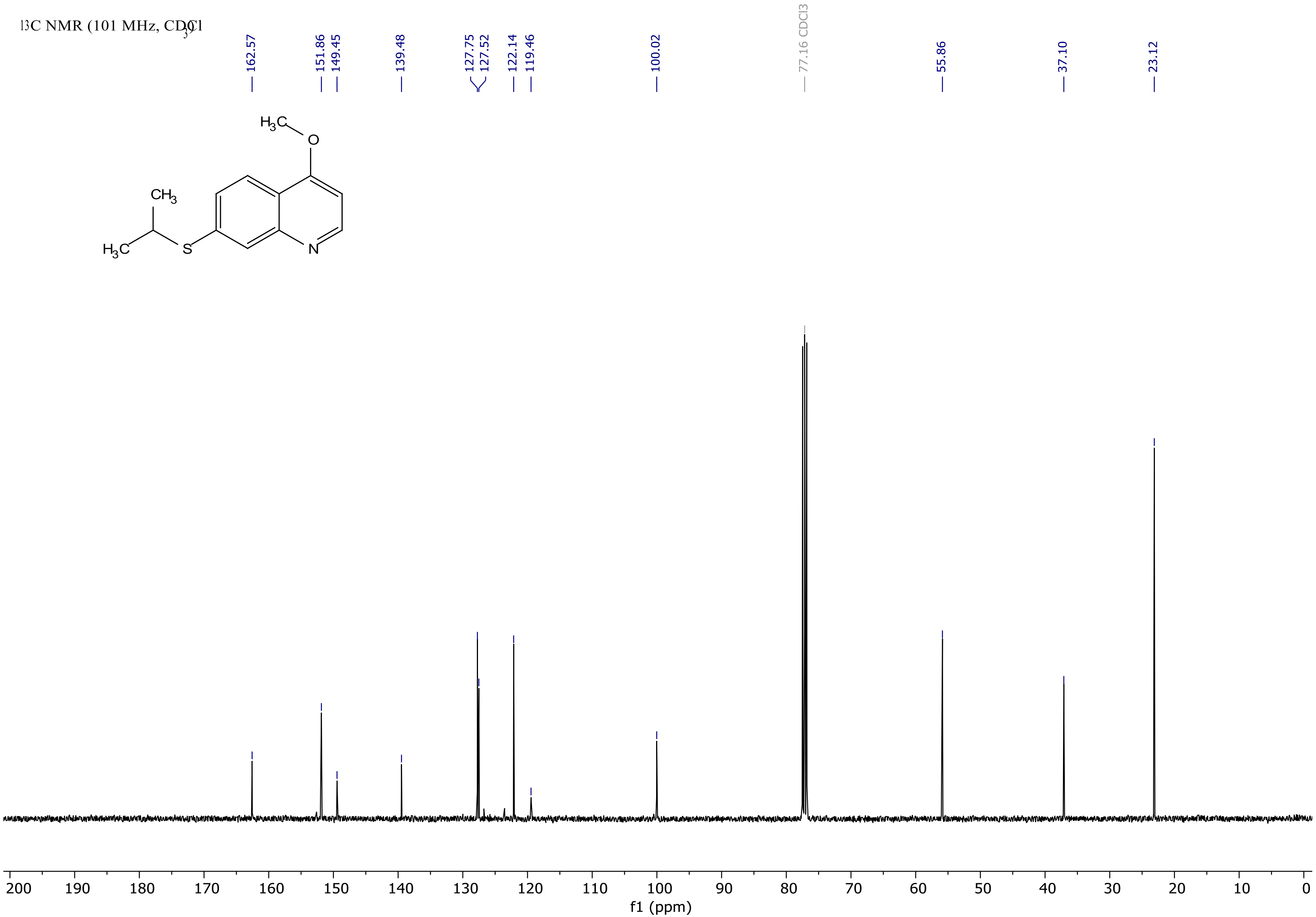

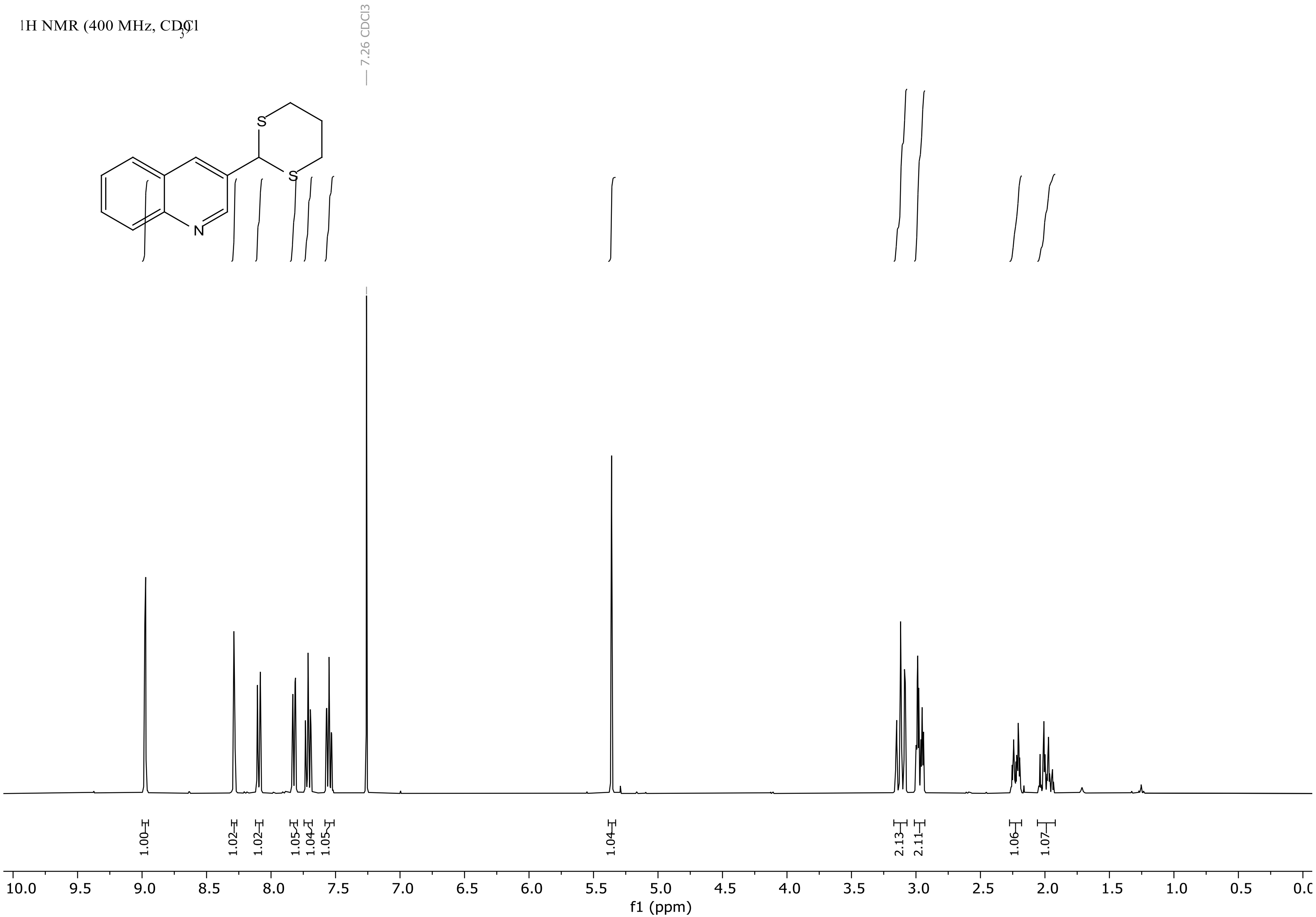

1H NMR (400 MHz, CDCl3)
7.26 CDCl3
S
S
N
1.00
1.02
1.02
1.05
1.04
1.05
1.04
2.13
2.11
1.06
1.07
10.0
9.5
9.0
8.5
8.0
7.5
7.0
6.5
6.0
5.5
5.0
4.5
4.0
3.5
3.0
2.5
2.0
1.5
1.0
0.5
f1 (ppm)

13C NMR (101 MHz, CDCl3)

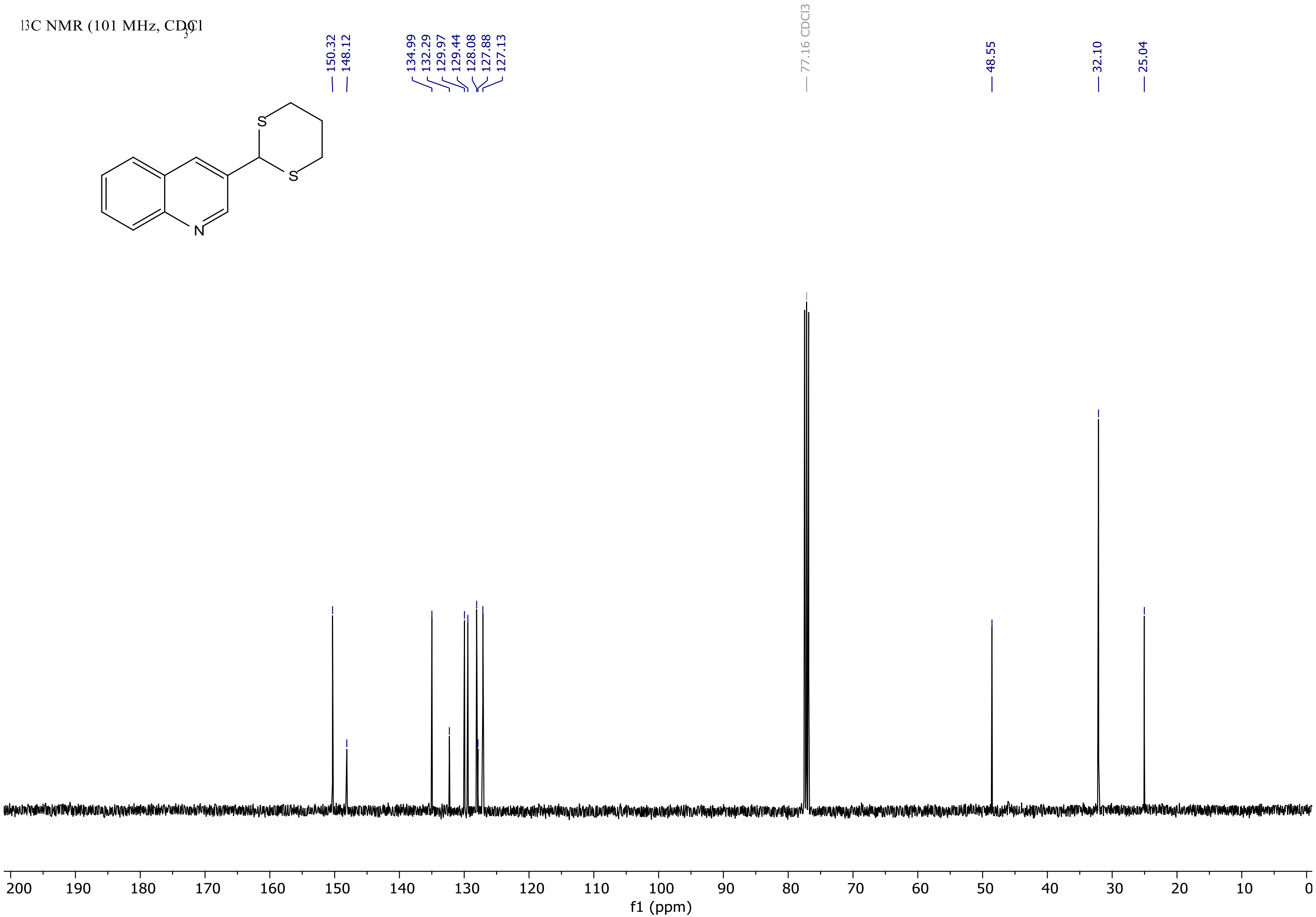

1H NMR (400 MHz, CDCl3)

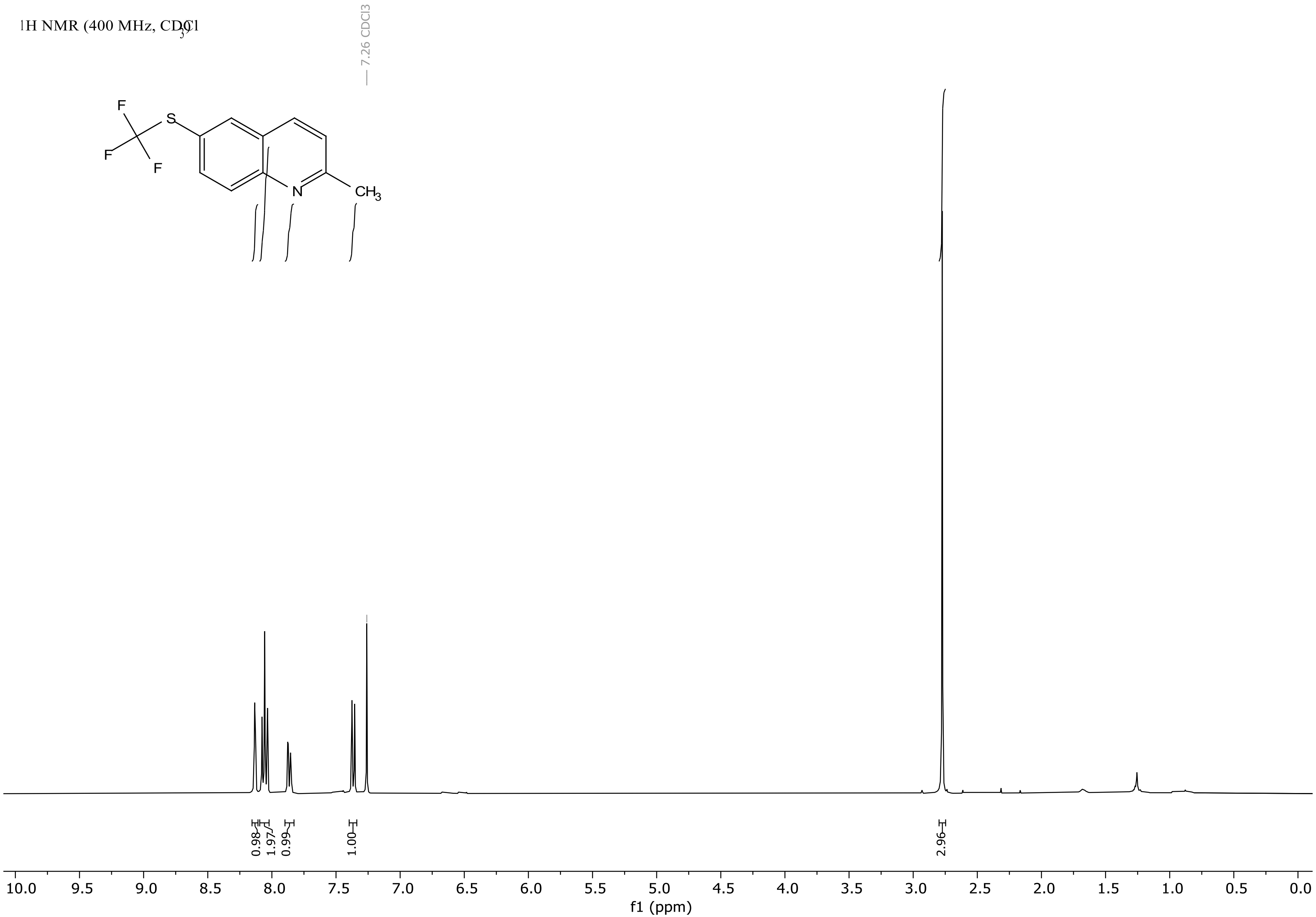

13C NMR (101 MHz, CDCl3)

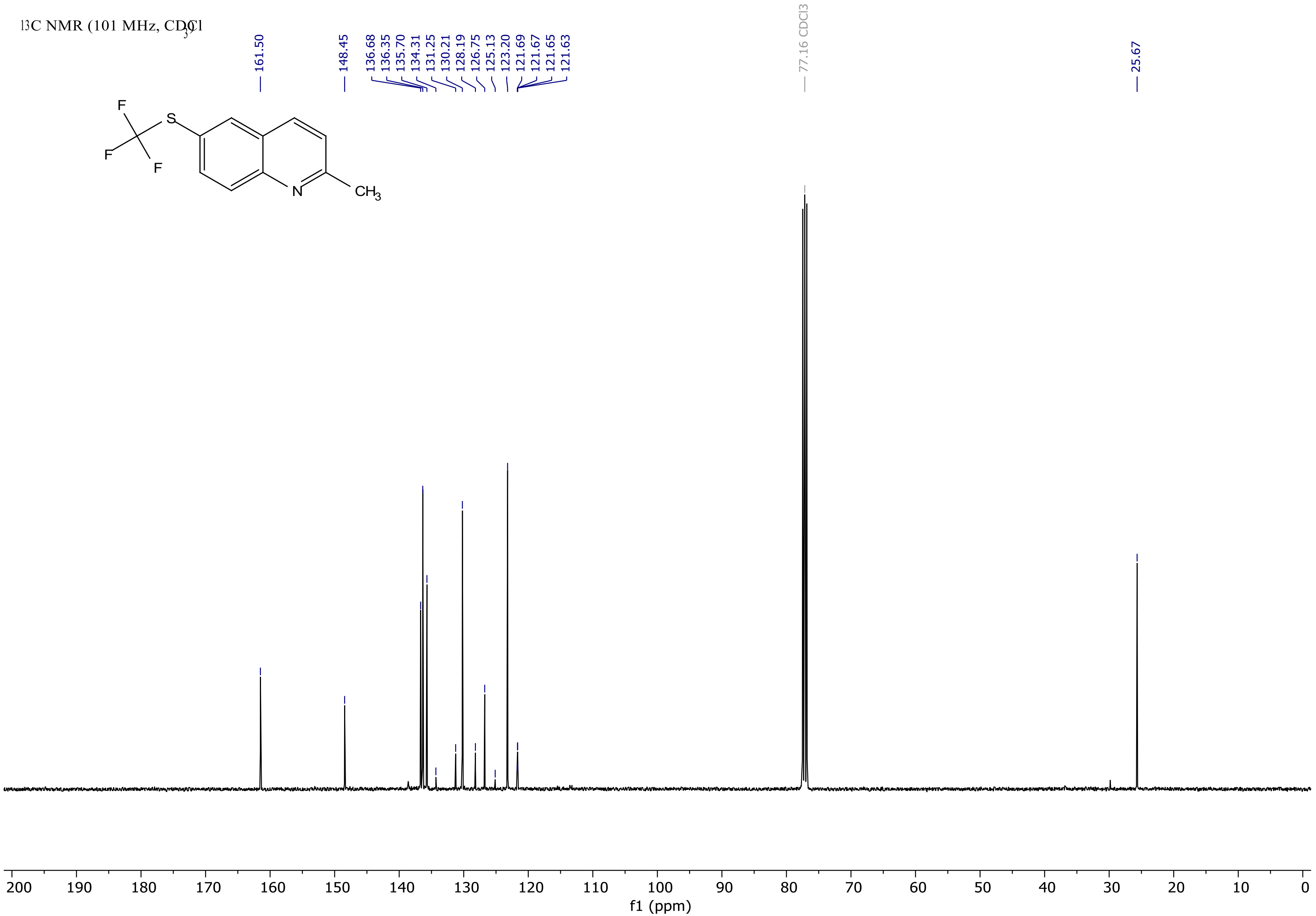

^{19}F NMR (376 MHz, $CDCl_3$)

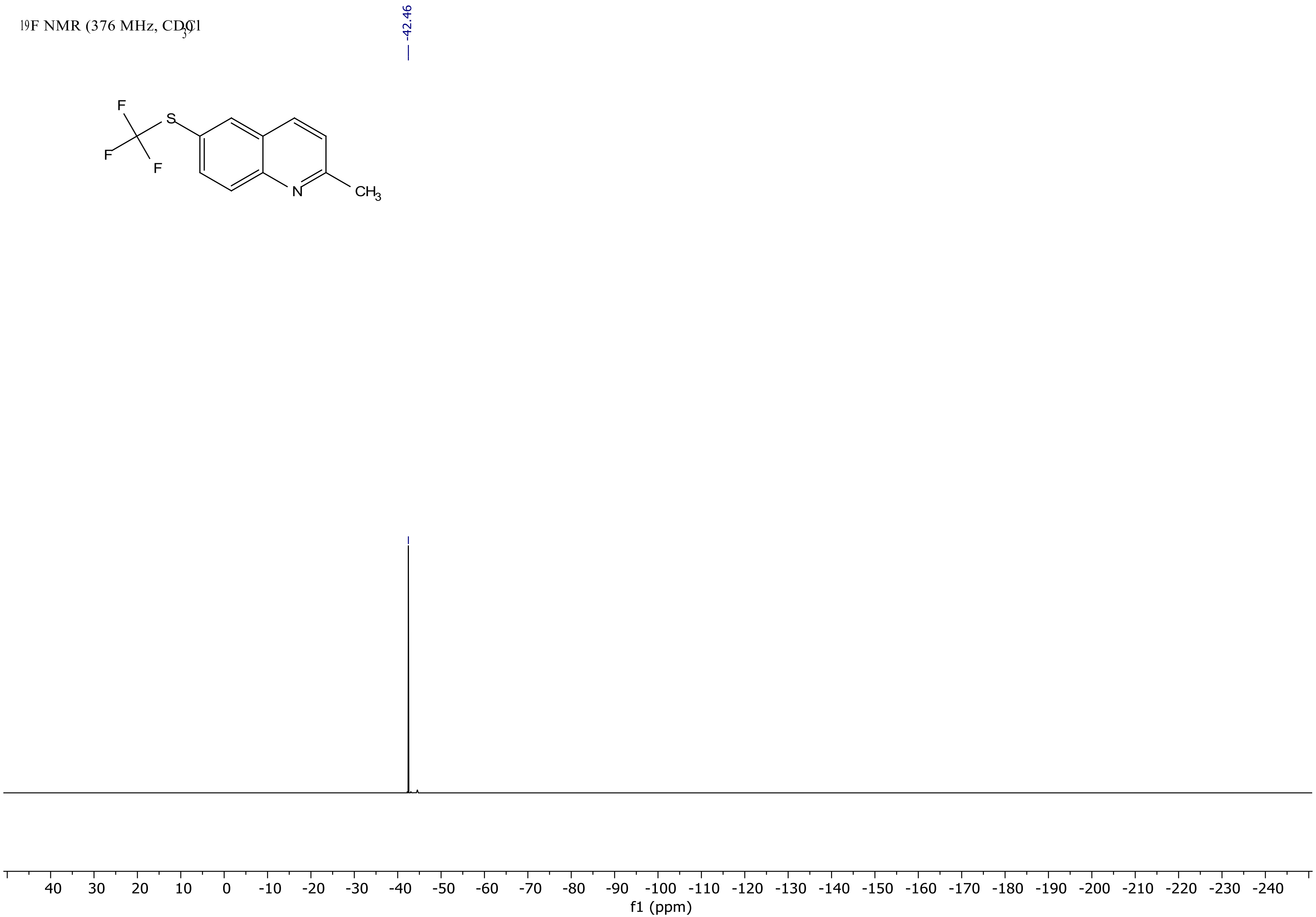

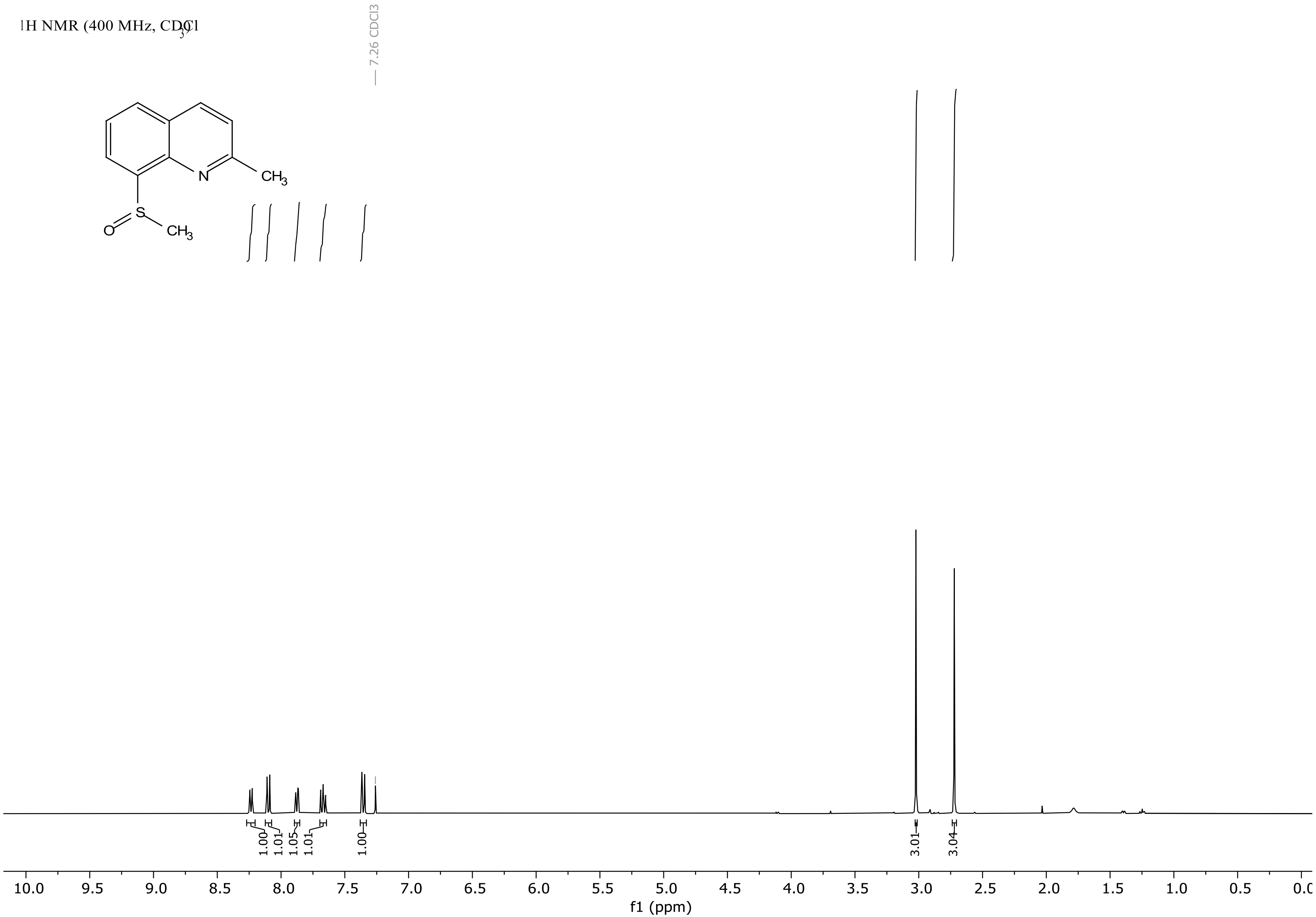
1H NMR (400 MHz, CDCl3)
7.26 CDCl3
N
CH3
S
O
CH3
1.00
1.01
1.05
1.01
1.00
3.01
3.04
10.0 9.5 9.0 8.5 8.0 7.5 7.0 6.5 6.0 5.5 5.0 4.5 4.0 3.5 3.0 2.5 2.0 1.5 1.0 0.5 0.0
f1 (ppm)

13C NMR (101 MHz, $CDCl_3$)

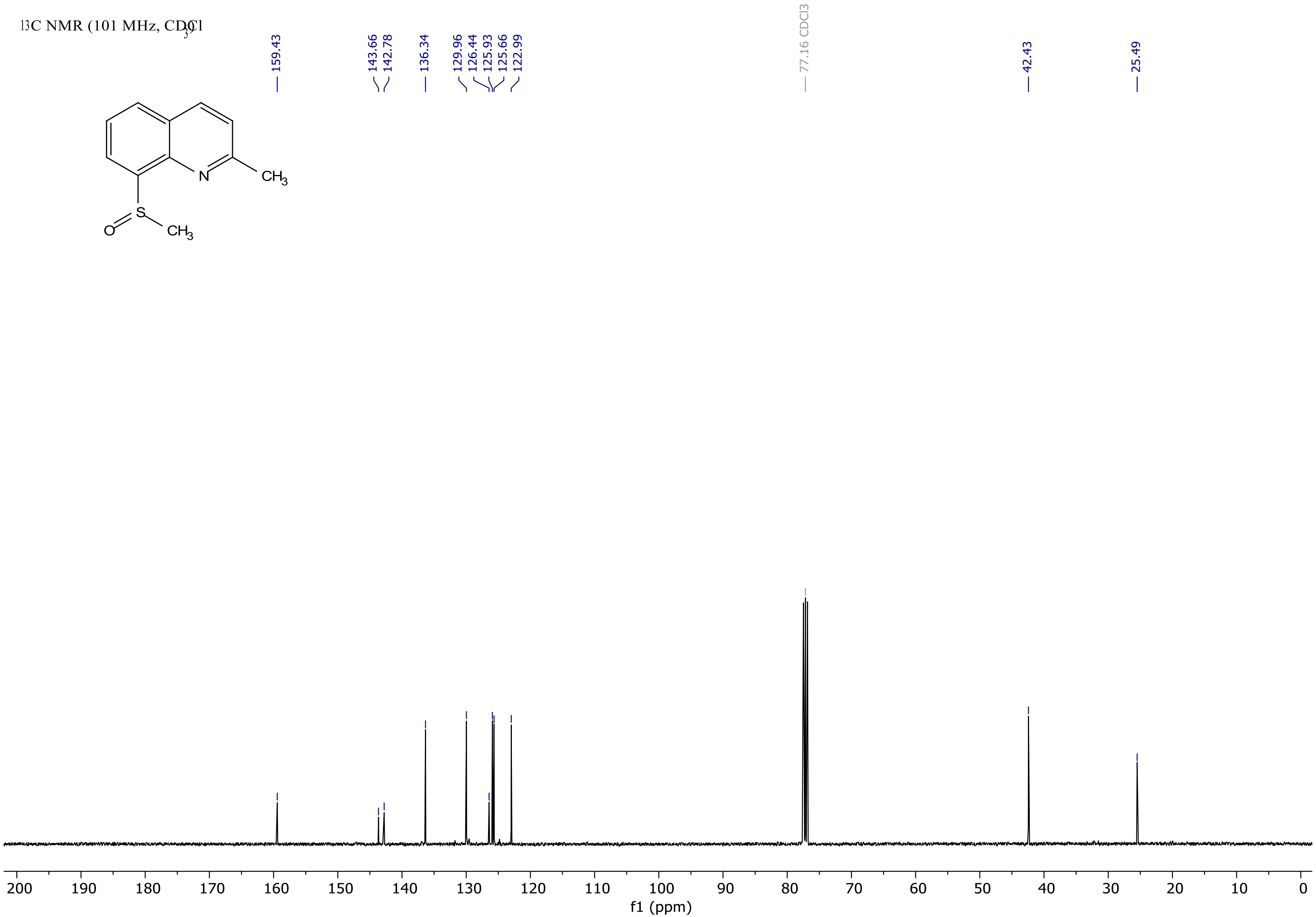

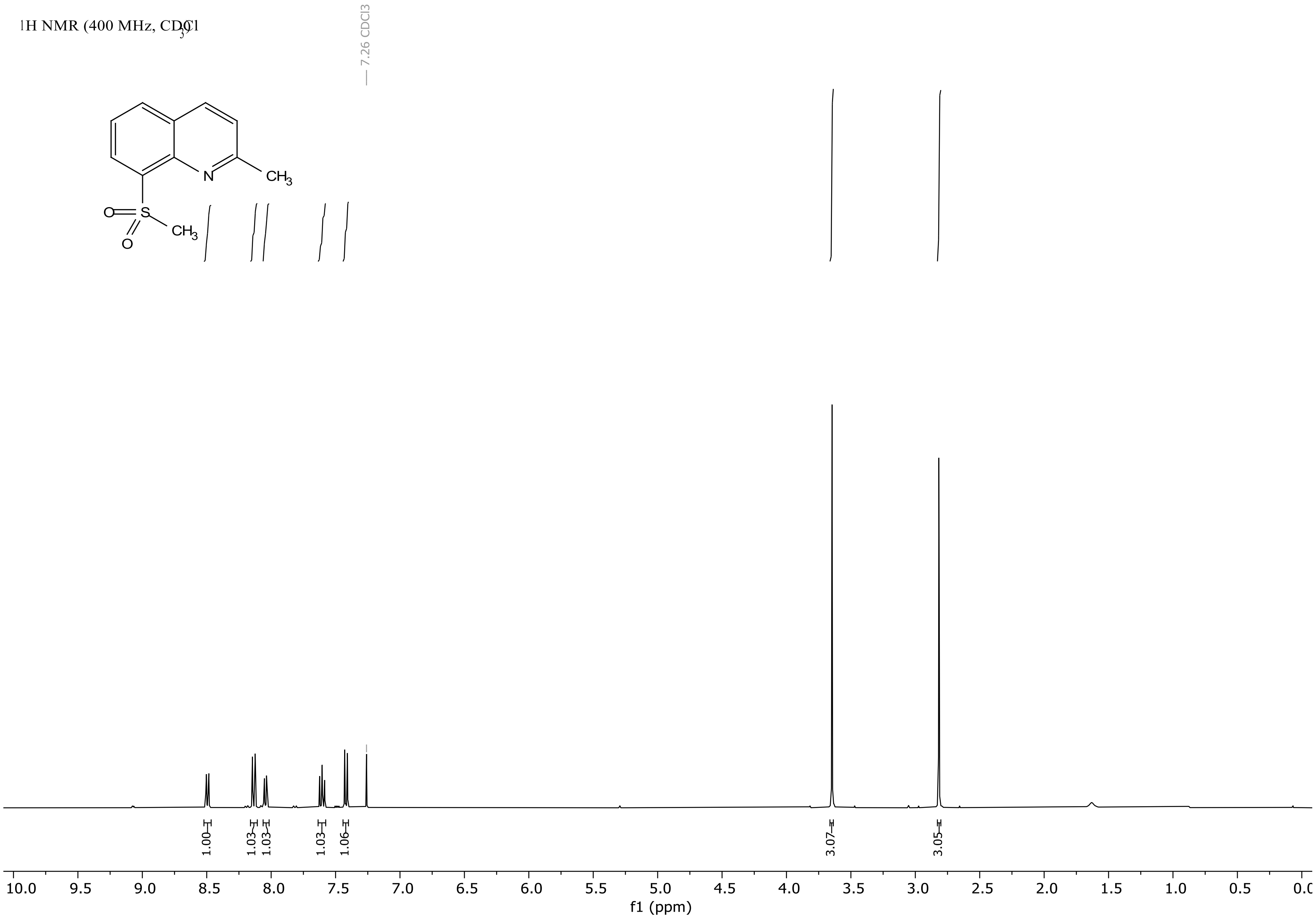
1H NMR (400 MHz, CDCl3)
7.26 CDCl3
N
CH3
S
O
O
CH3
1.00
1.03
1.03
1.03
1.06
3.07
3.05
10.0
9.5
9.0
8.5
8.0
7.5
7.0
6.5
6.0
5.5
5.0
4.5
4.0
3.5
3.0
2.5
2.0
1.5
1.0
0.5
f1 (ppm)

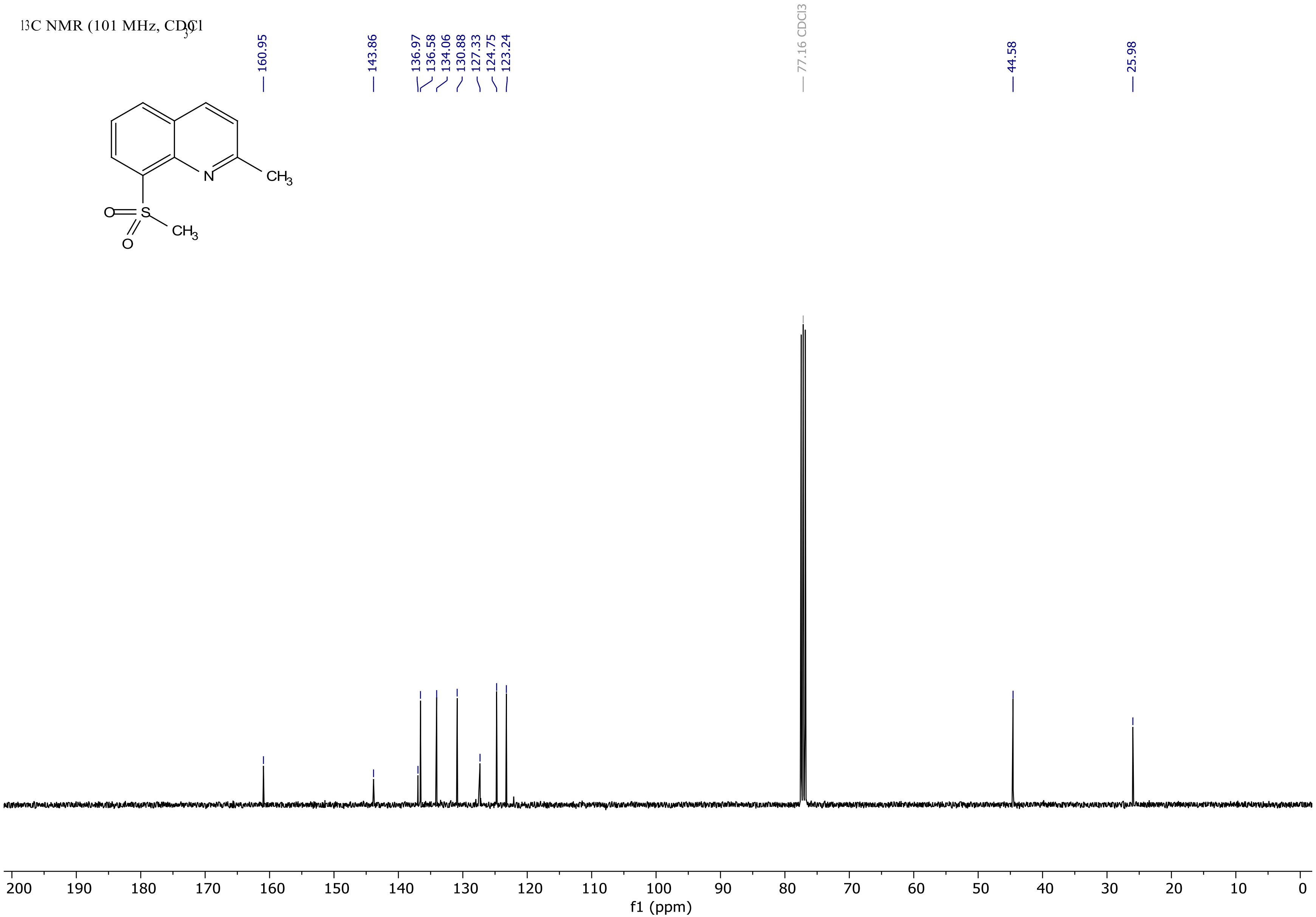
13C NMR (101 MHz, CDCl3)
160.95
143.86
136.97
136.58
134.06
130.88
127.33
124.75
123.24
77.16 CDCl3
44.58
25.98
N
CH3
O
S
O
CH3
200
190
180
170
160
150
140
130
120
110
100
90
80
70
60
50
40
30
20
10
0
f1 (ppm)

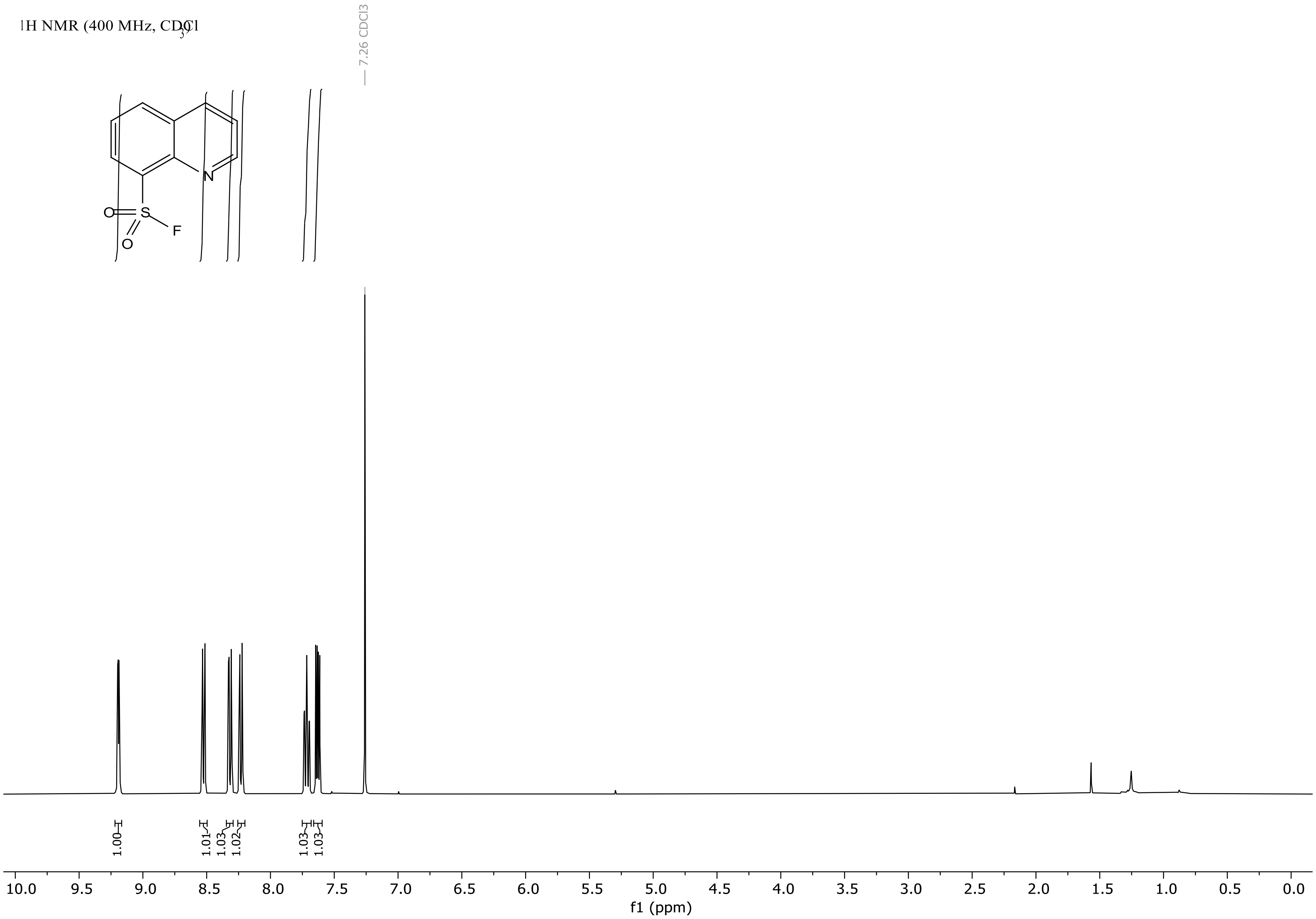
1H NMR (400 MHz, CDCl3)
7.26 CDCl3
N
O
S
F
O
1.00
1.01
1.03
1.02
1.03
1.03
10.0 9.5 9.0 8.5 8.0 7.5 7.0 6.5 6.0 5.5 5.0 4.5 4.0 3.5 3.0 2.5 2.0 1.5 1.0 0.5 0.0
f1 (ppm)

13C NMR (101 MHz, CDCl3)

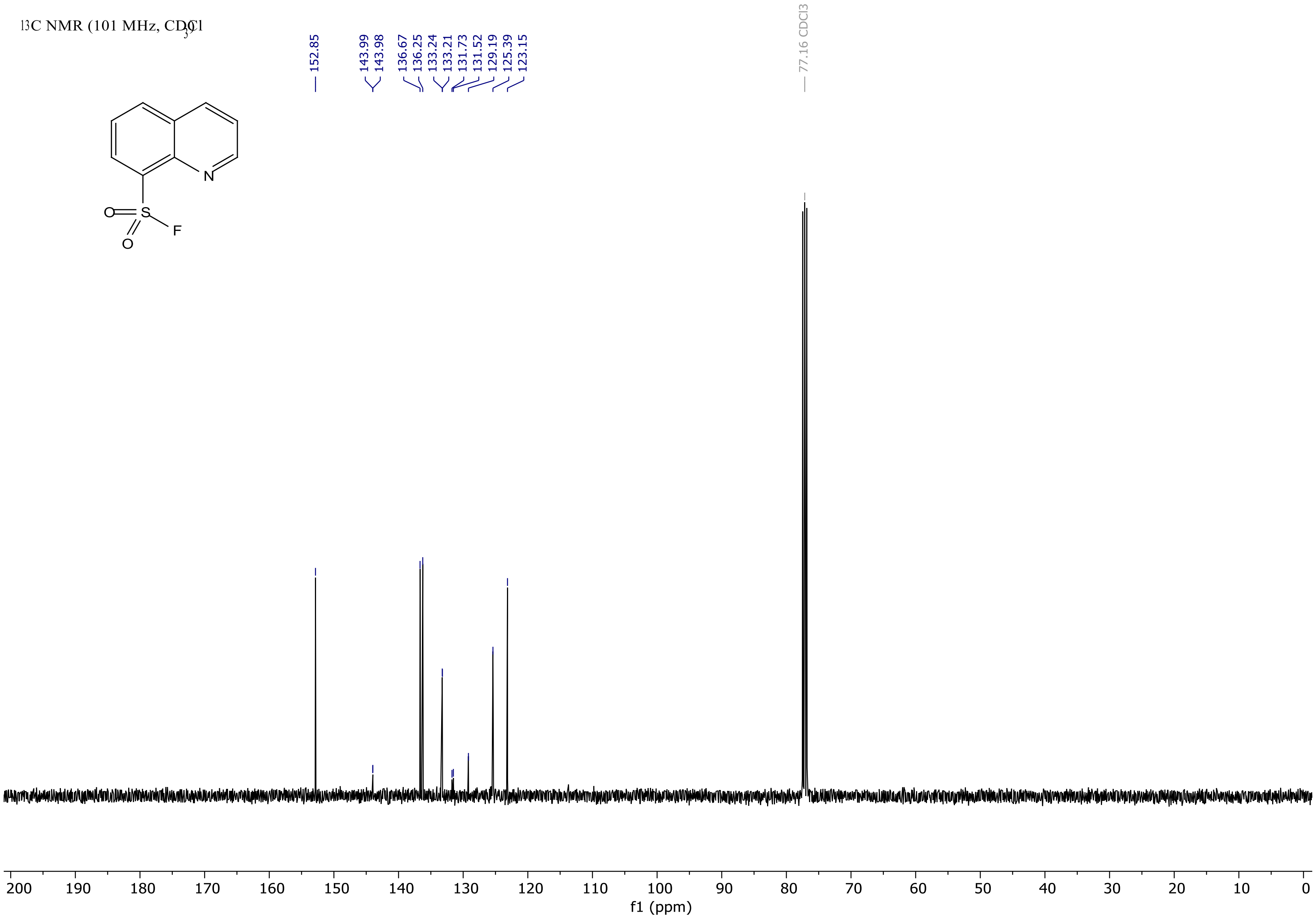

^{19}F NMR (376 MHz, $CDCl_3$)

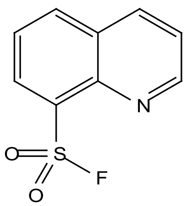

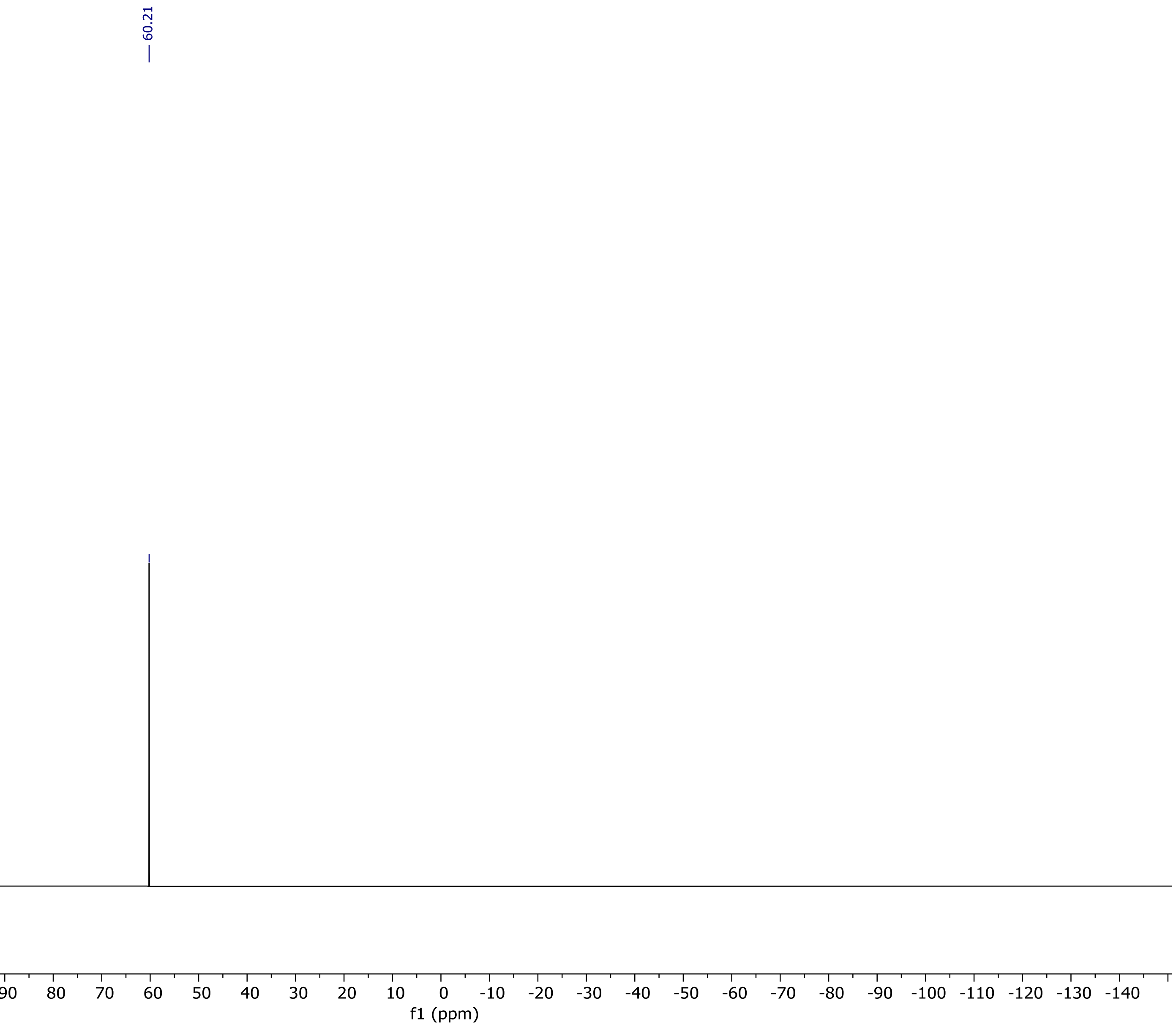

1H NMR (400 MHz, CDCl3)

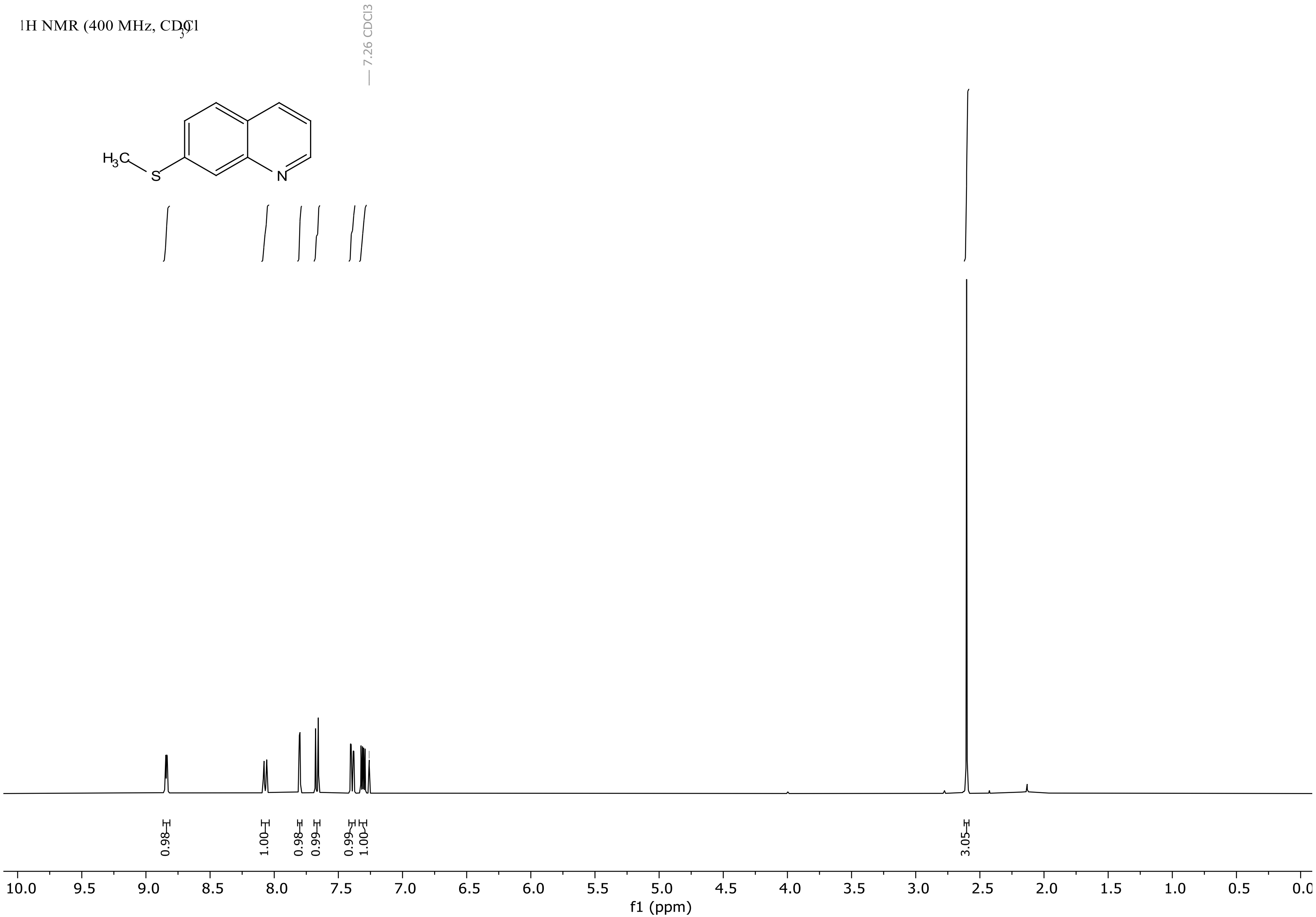

13C NMR (101 MHz, CDCl3)

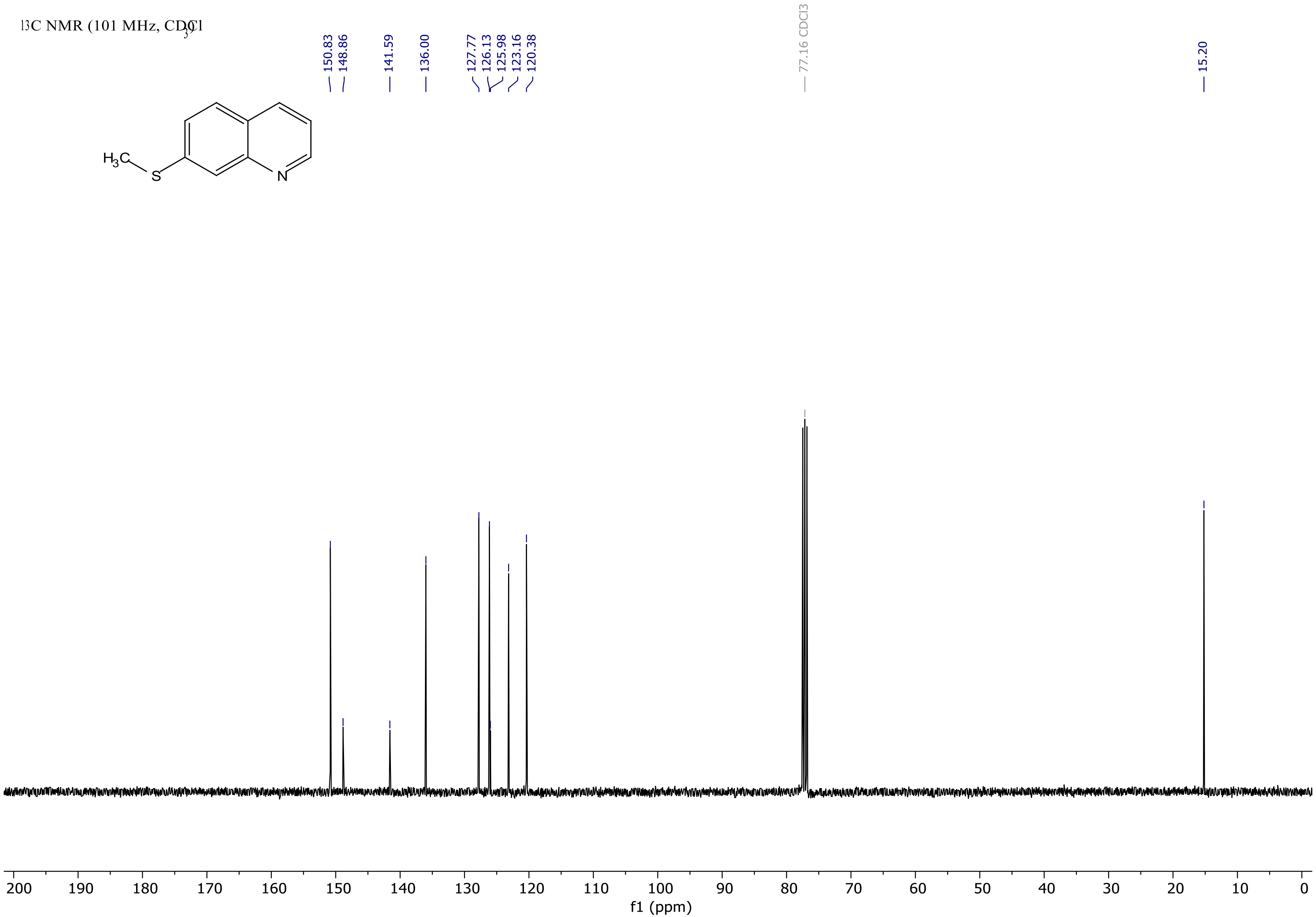

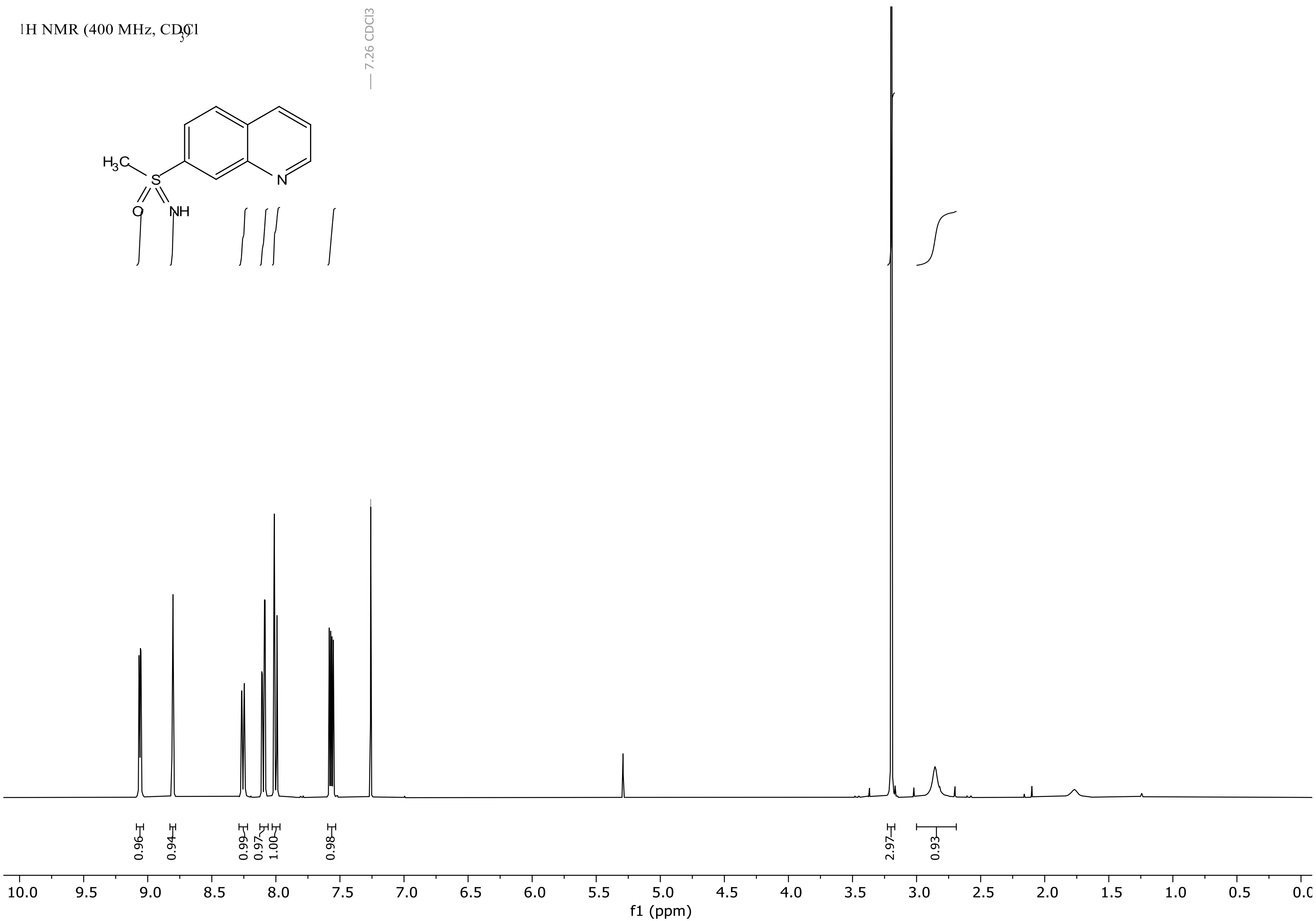

1H NMR (400 MHz, CDCl3)
7.26 CDCl3
H3C
S
O
NH
N
0.96
0.94
0.99
0.97
1.00
0.98
2.97
0.93
10.0 9.5 9.0 8.5 8.0 7.5 7.0 6.5 6.0 5.5 5.0 4.5 4.0 3.5 3.0 2.5 2.0 1.5 1.0 0.5 0.0
f1 (ppm)

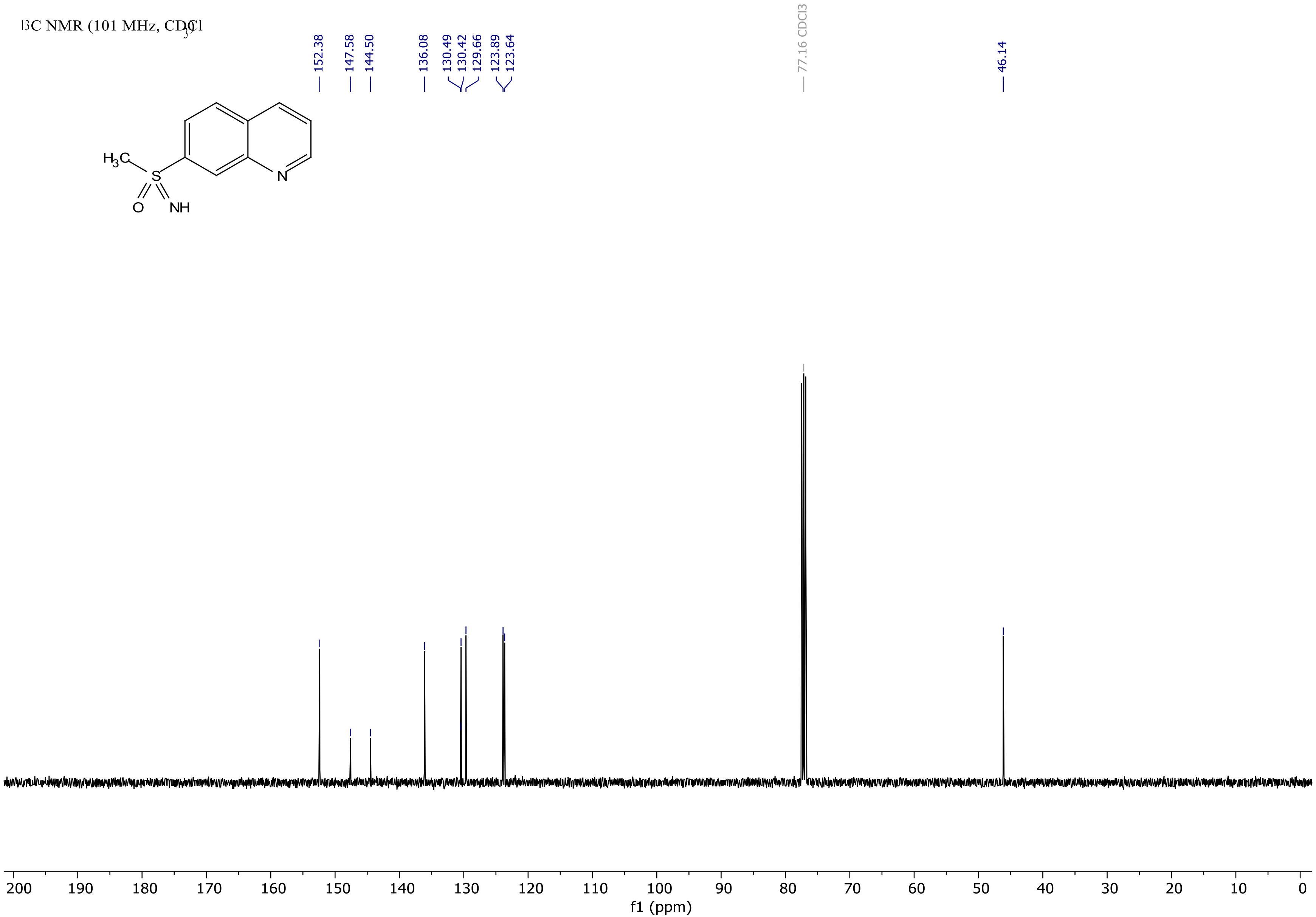
13C NMR (101 MHz, CDCl3)
152.38
147.58
144.50
136.08
130.49
130.42
129.66
123.89
123.64
77.16 CDCl3
46.14
H3C
S
O
NH
N
200
190
180
170
160
150
140
130
120
110
100
90
80
70
60
50
40
30
20
10
0
f1 (ppm)

1H NMR (400 MHz, CDCl3)

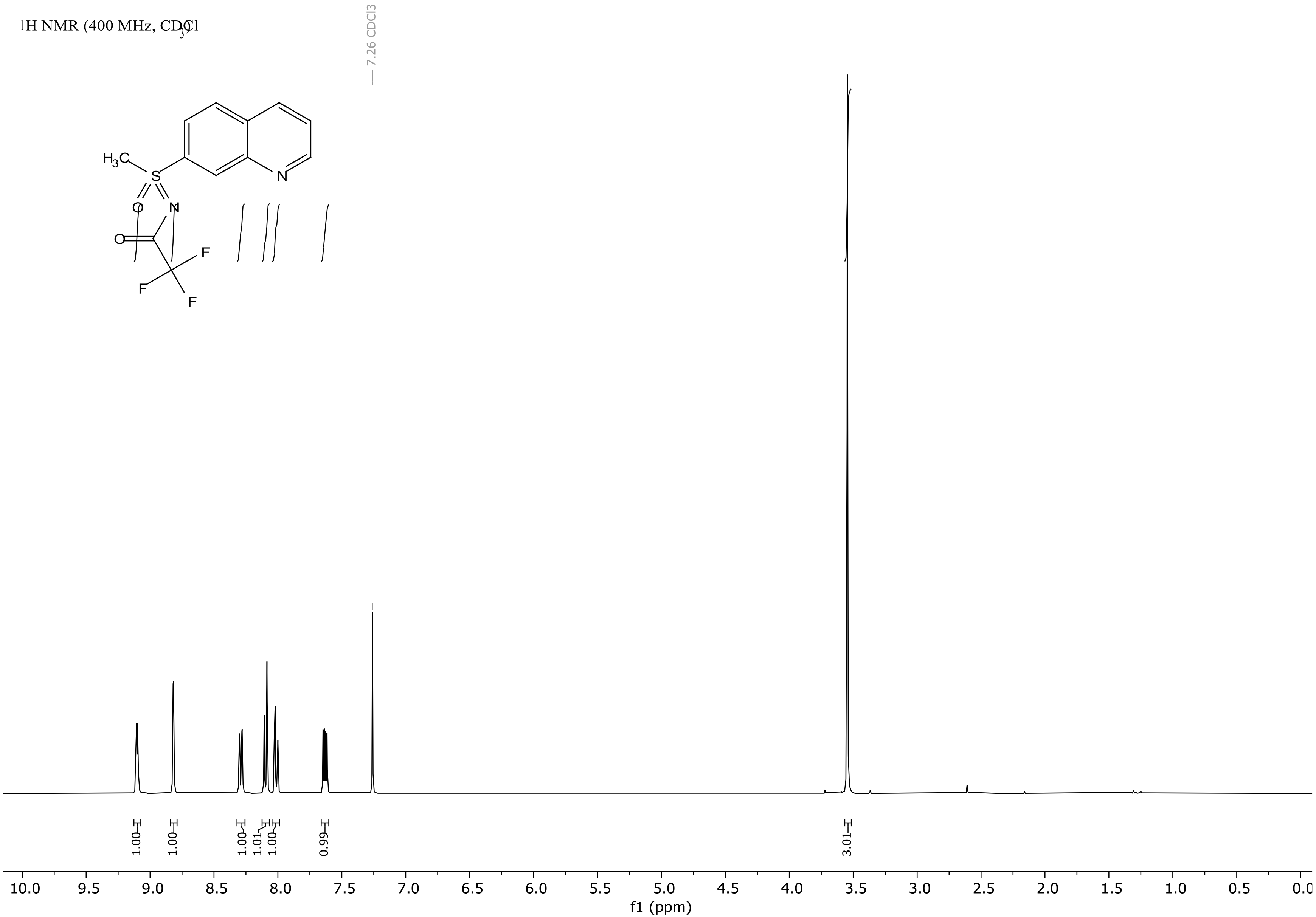

13C NMR (101 MHz, CDCl3)

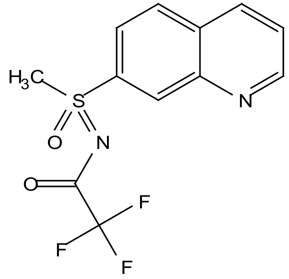

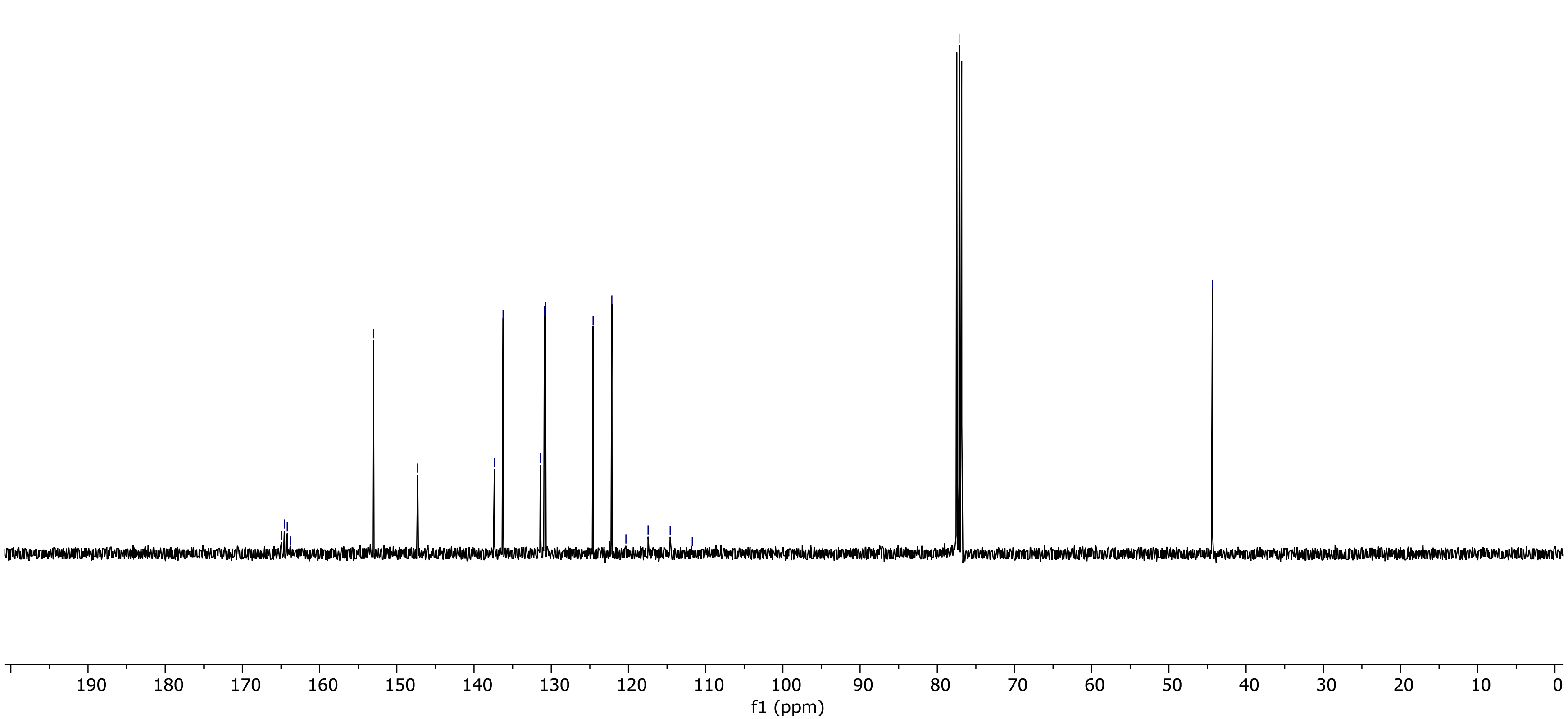

19F NMR (376 MHz, $CDCl_3$)

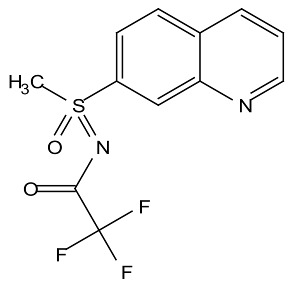

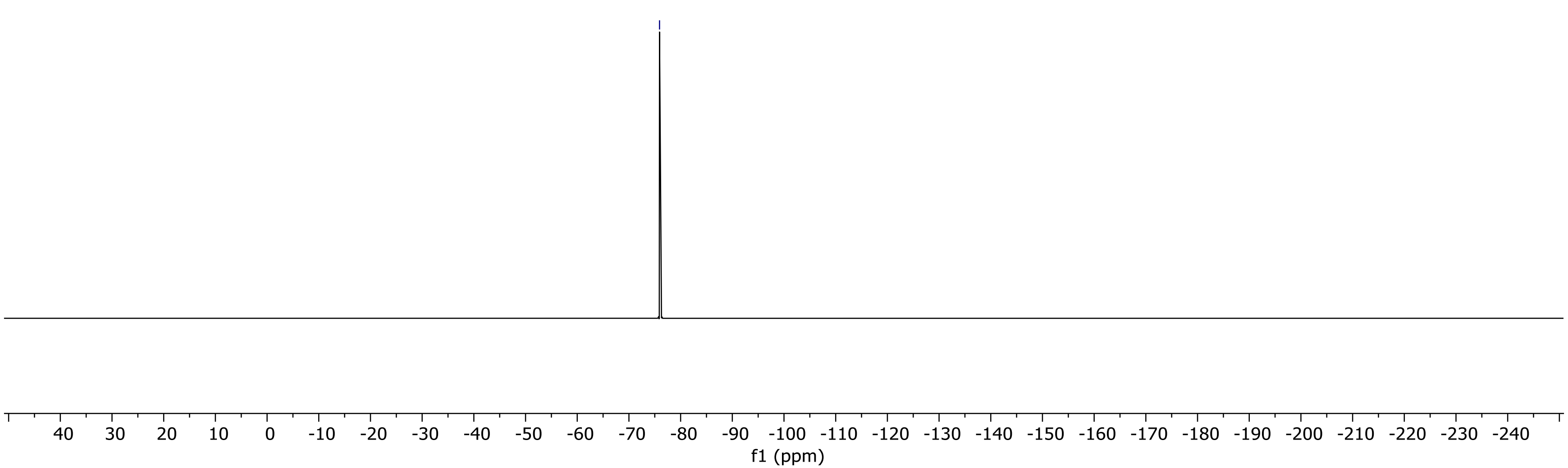

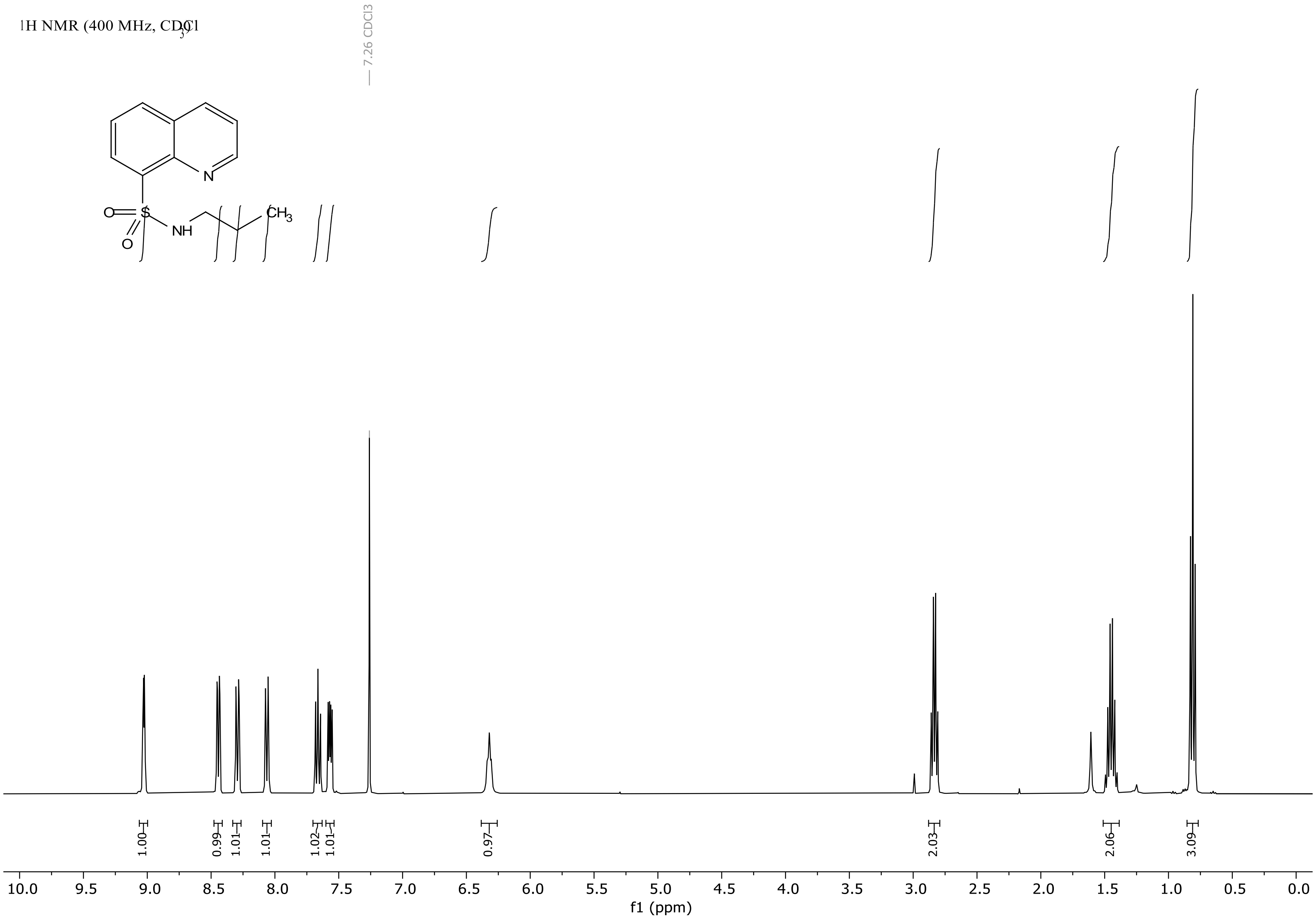
1H NMR (400 MHz, CDCl3)
7.26 CDCl3
N
O
S
O
NH
CH3
1.00
0.99
1.01
1.01
1.02
1.01
0.97
2.03
2.06
3.09
10.0 9.5 9.0 8.5 8.0 7.5 7.0 6.5 6.0 5.5 5.0 4.5 4.0 3.5 3.0 2.5 2.0 1.5 1.0 0.5 0.0
f1 (ppm)

13C NMR (101 MHz, CDCl3)

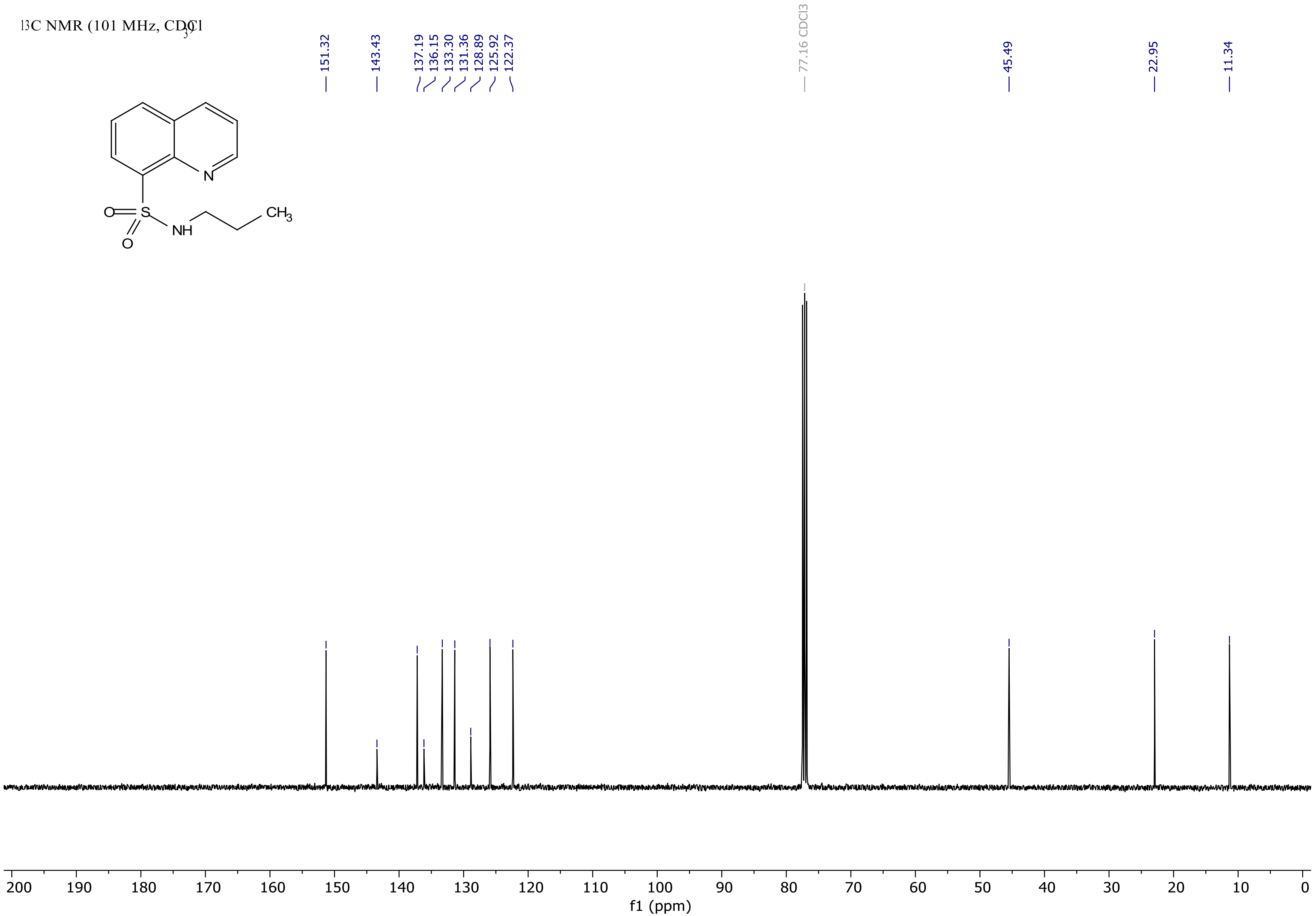

1H NMR (400 MHz, CDCl3)

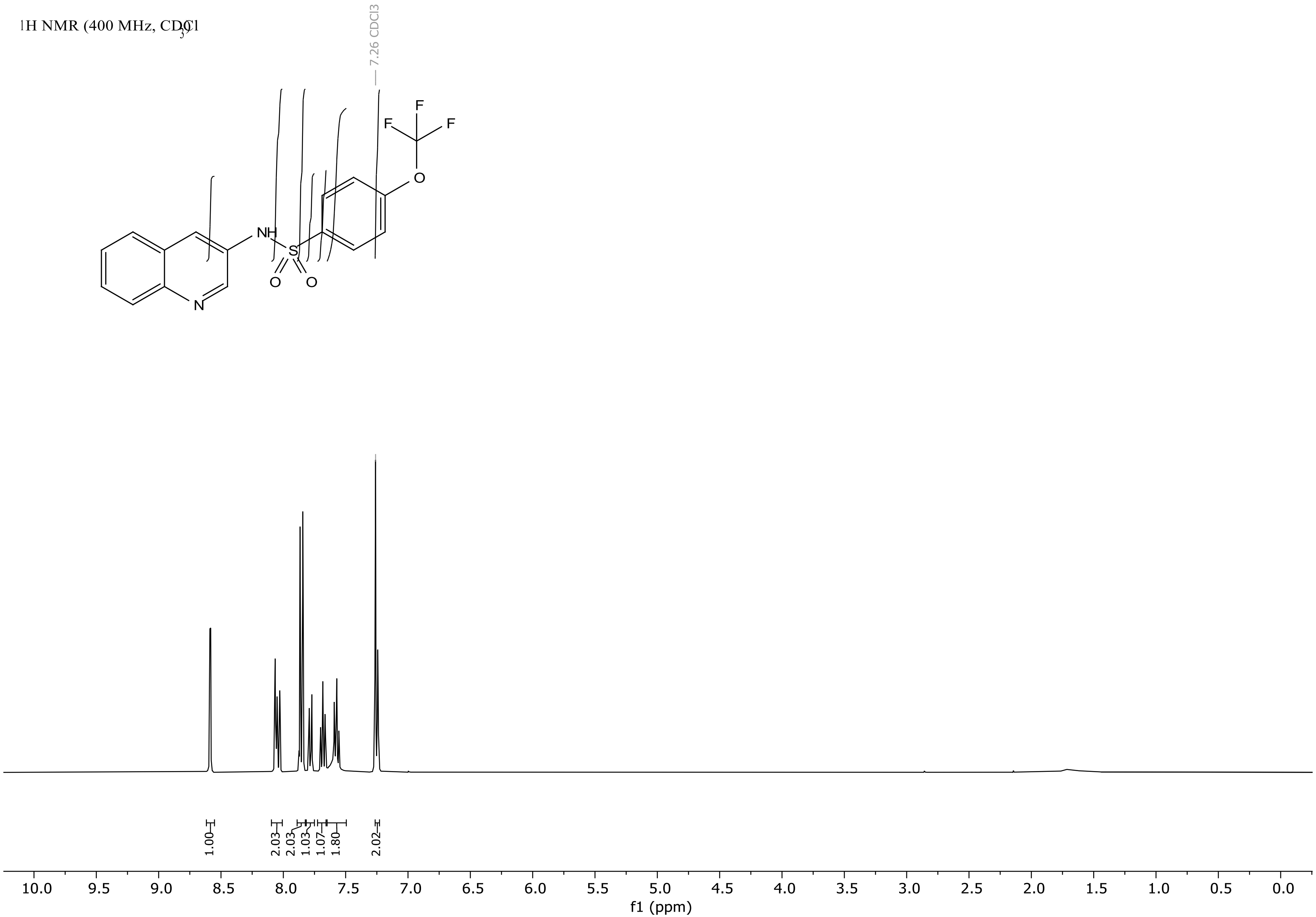

13C NMR (101 MHz, CDCl3)

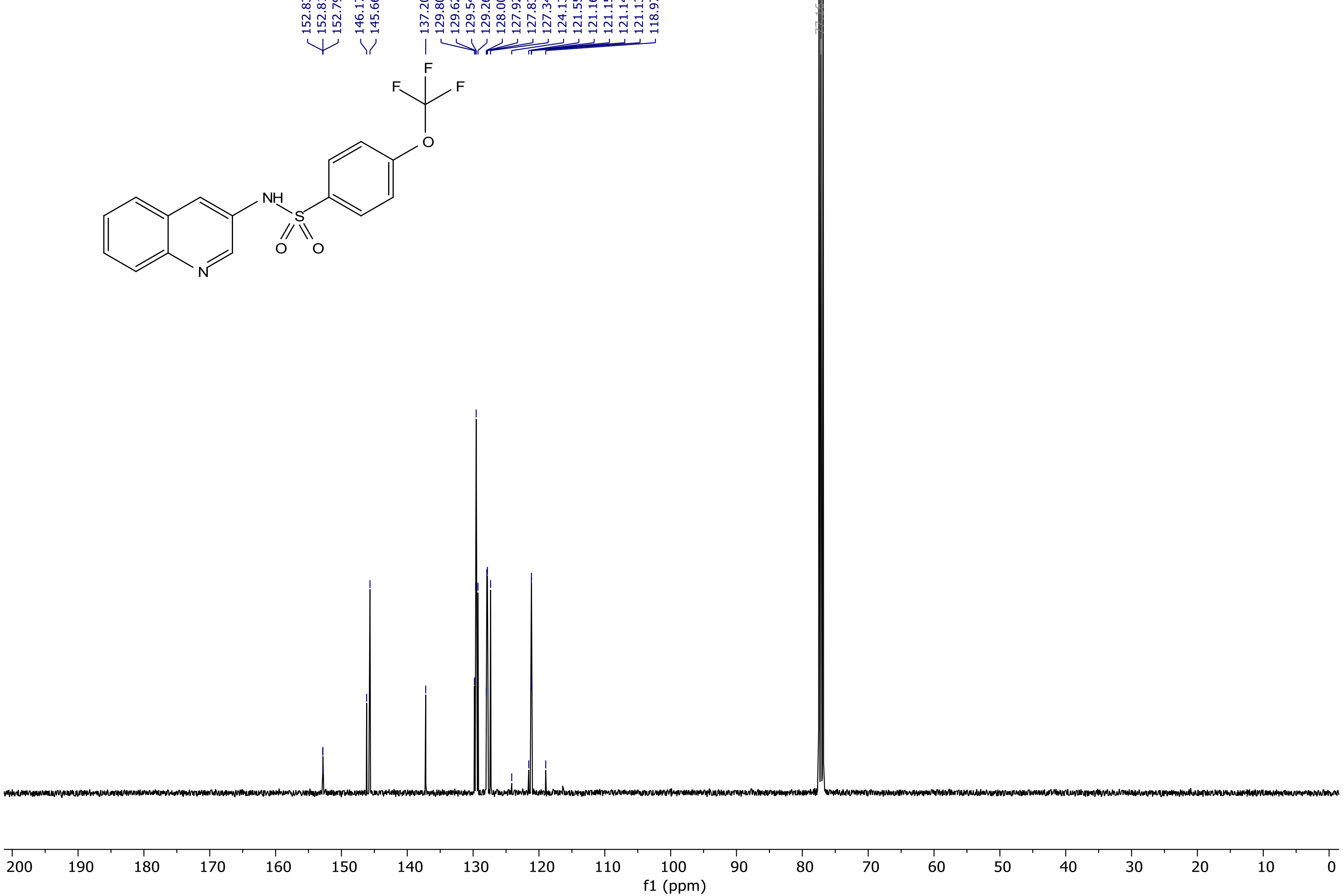

19F NMR (376 MHz, CDCl3)

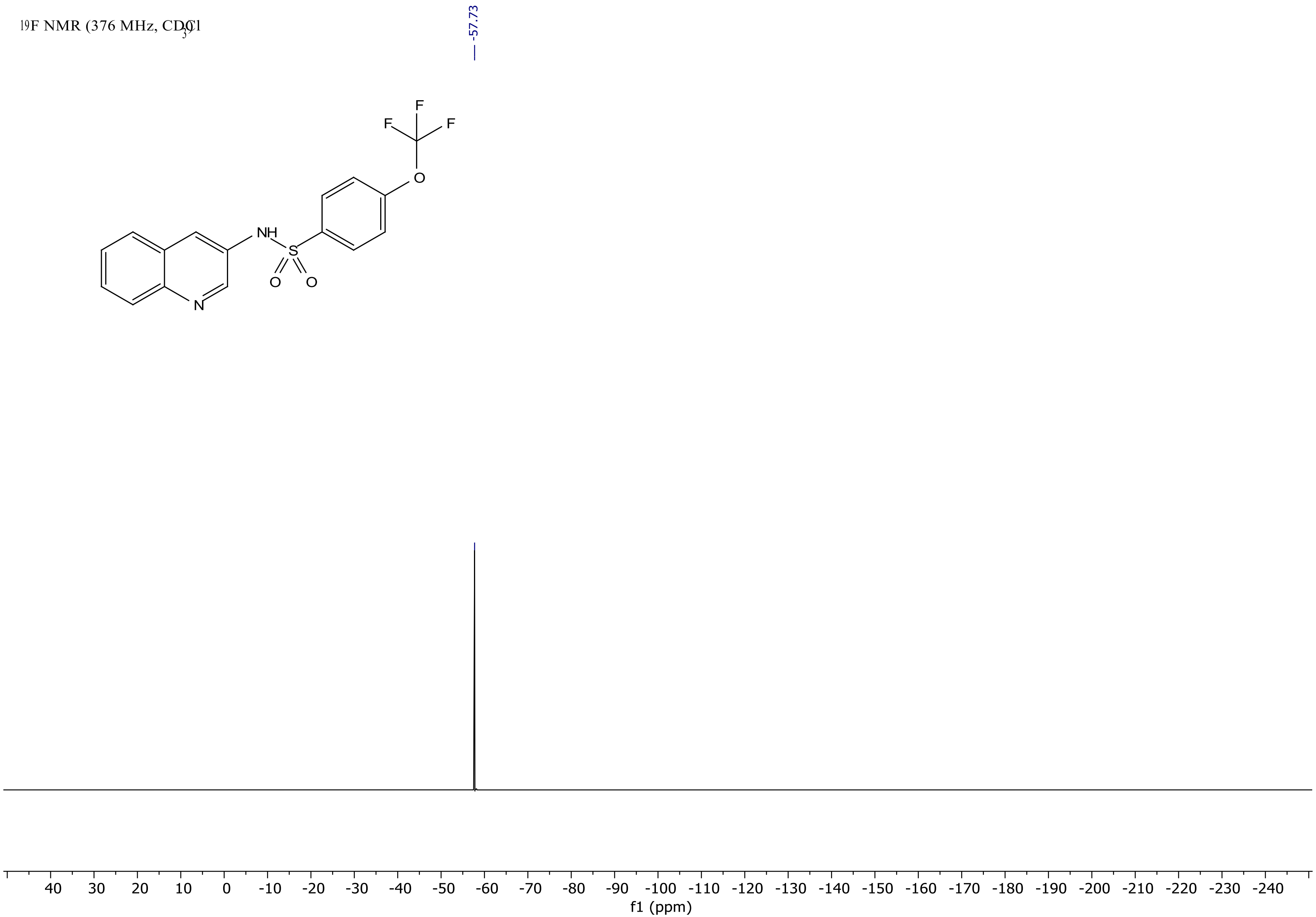

1H NMR (400 MHz, $CDCl_3$) δ 1.4.

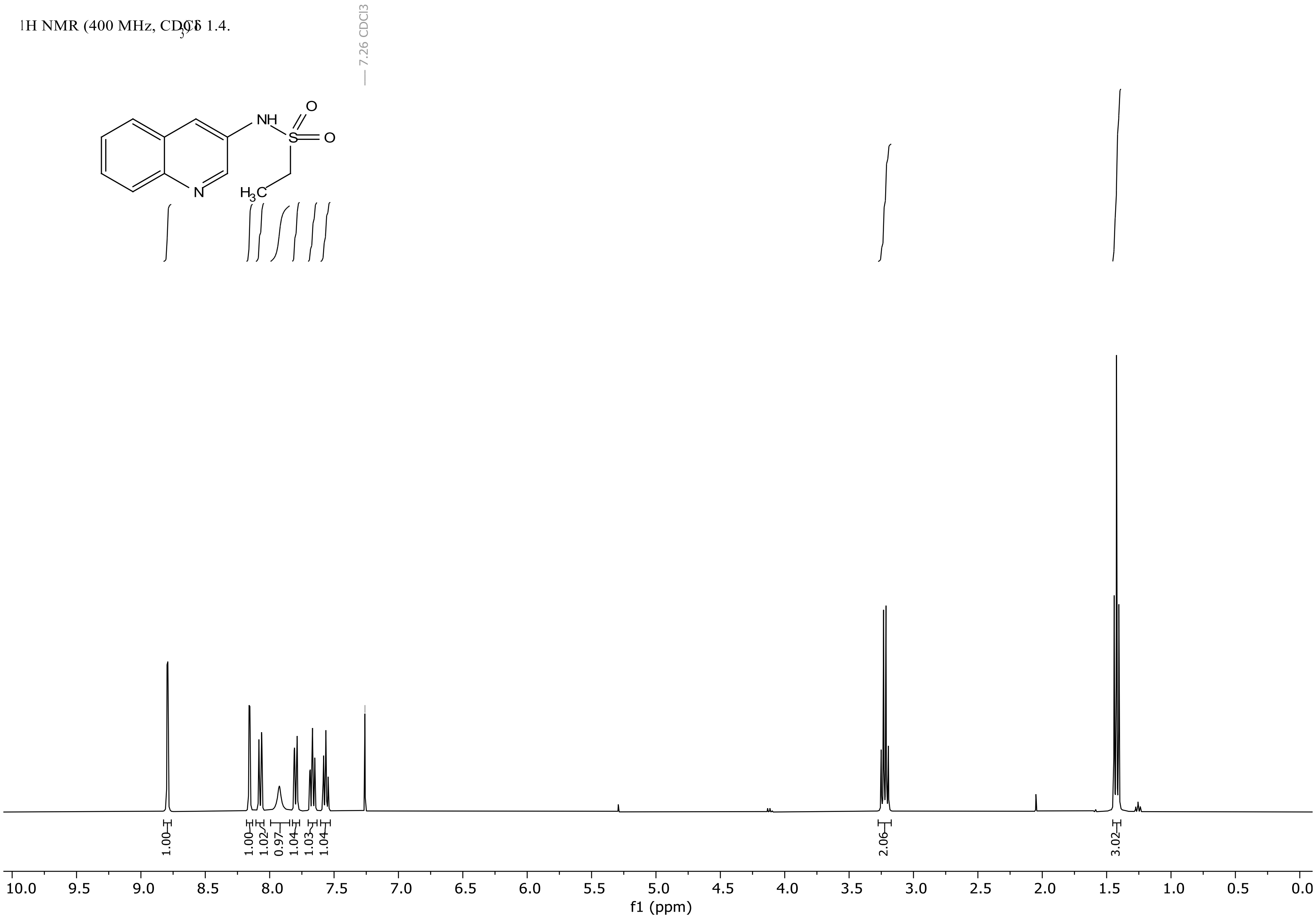

13C NMR (101 MHz, CDCl3)

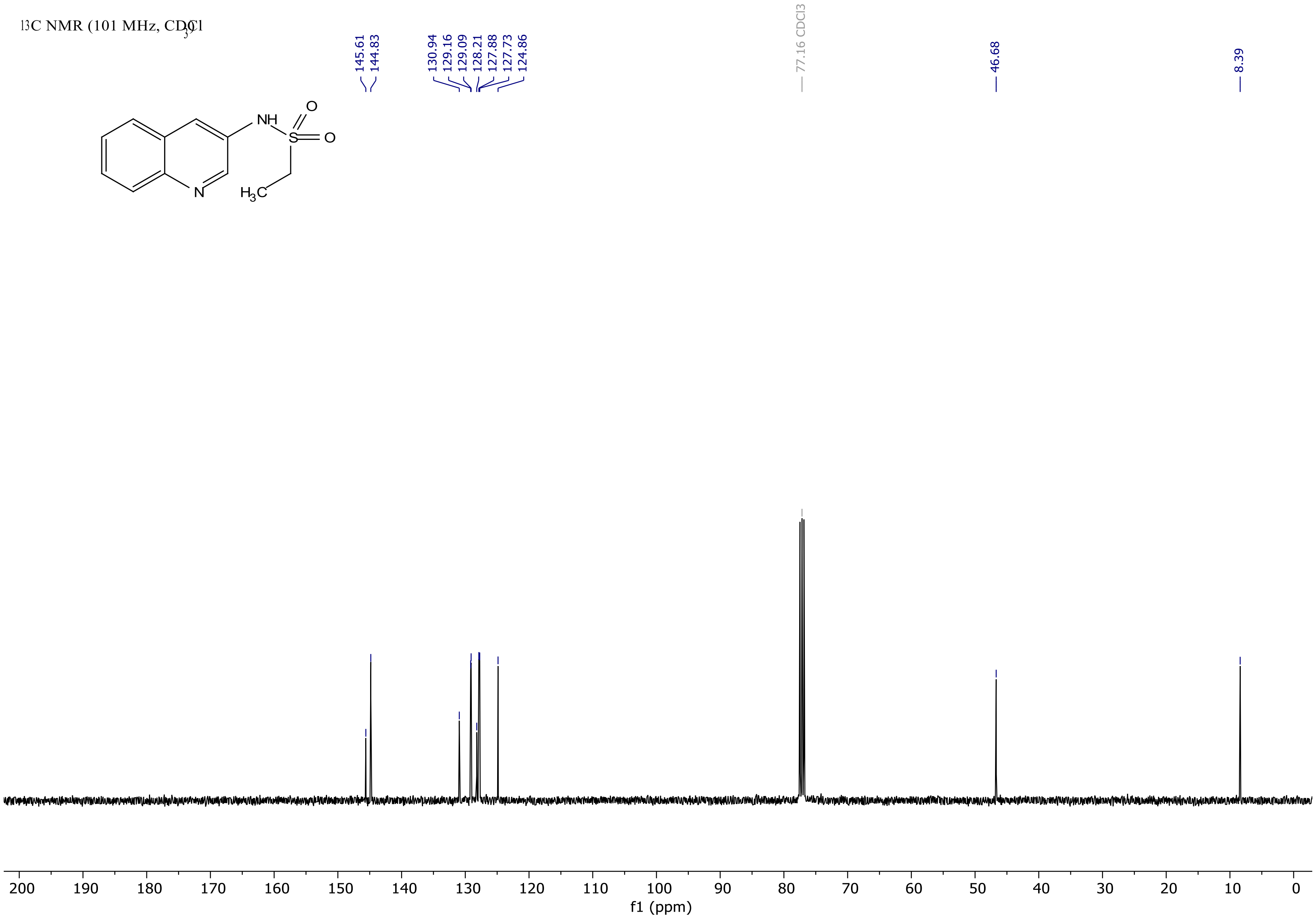

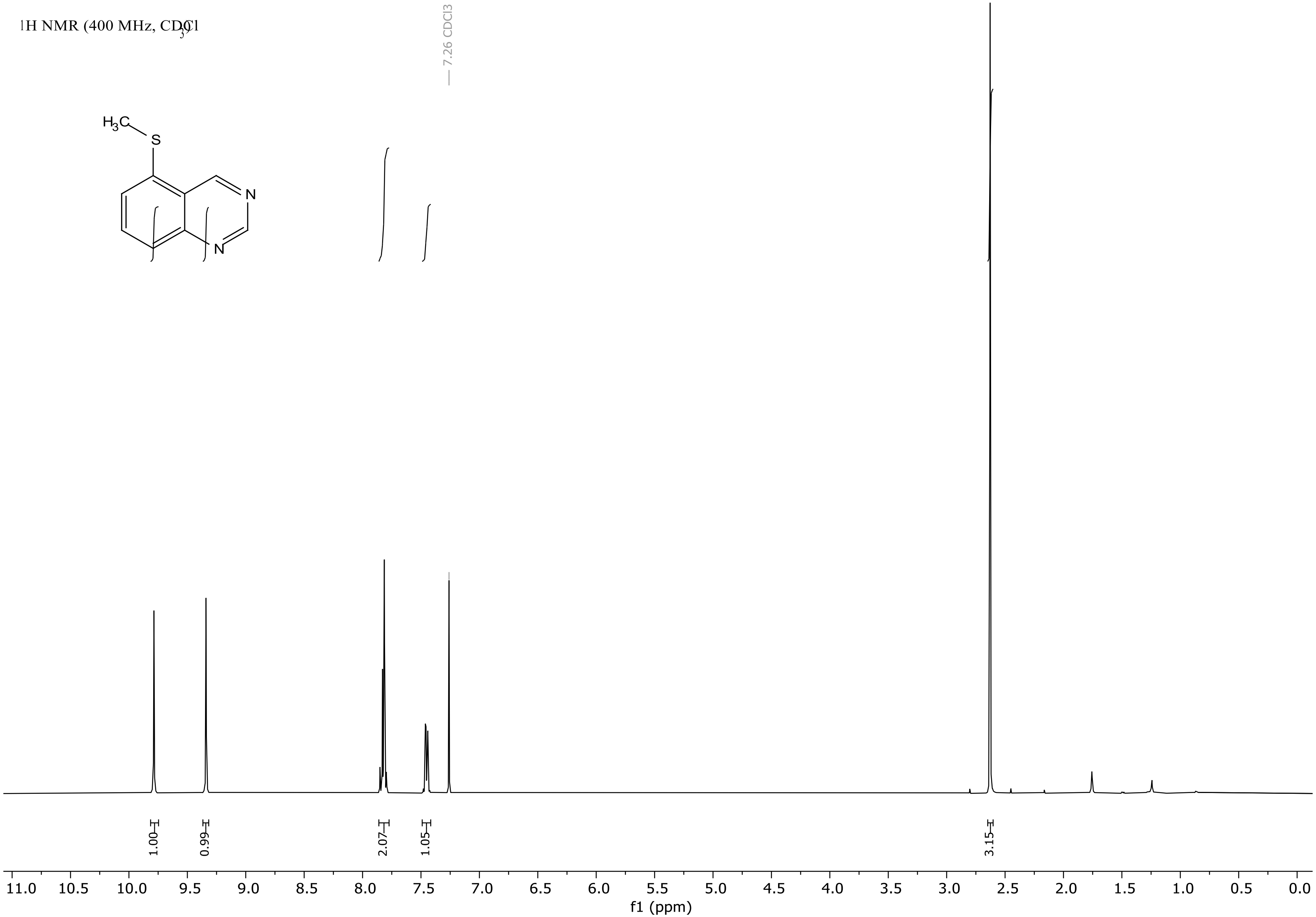
1H NMR (400 MHz, CDCl3)
7.26 CDCl3
H3C
S
N
N
1.00
0.99
2.07
1.05
3.15
11.0
10.5
10.0
9.5
9.0
8.5
8.0
7.5
7.0
6.5
6.0
5.5
5.0
4.5
4.0
3.5
3.0
2.5
2.0
1.5
1.0
0.5
0.0
f1 (ppm)

13C NMR (101 MHz, CDCl3)

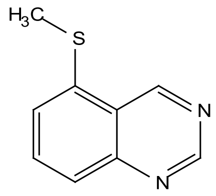

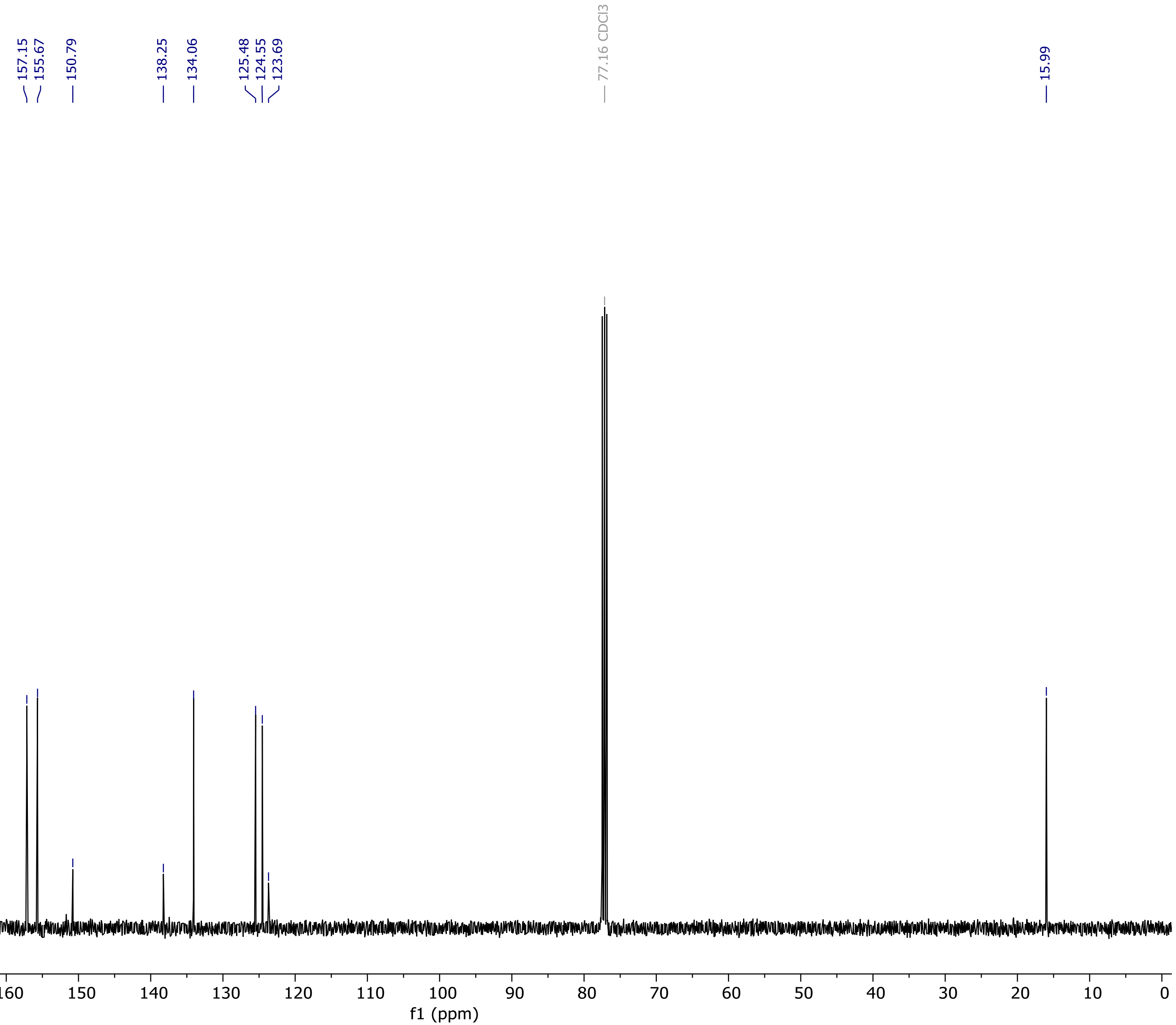

1H NMR (400 MHz, CDCl3)

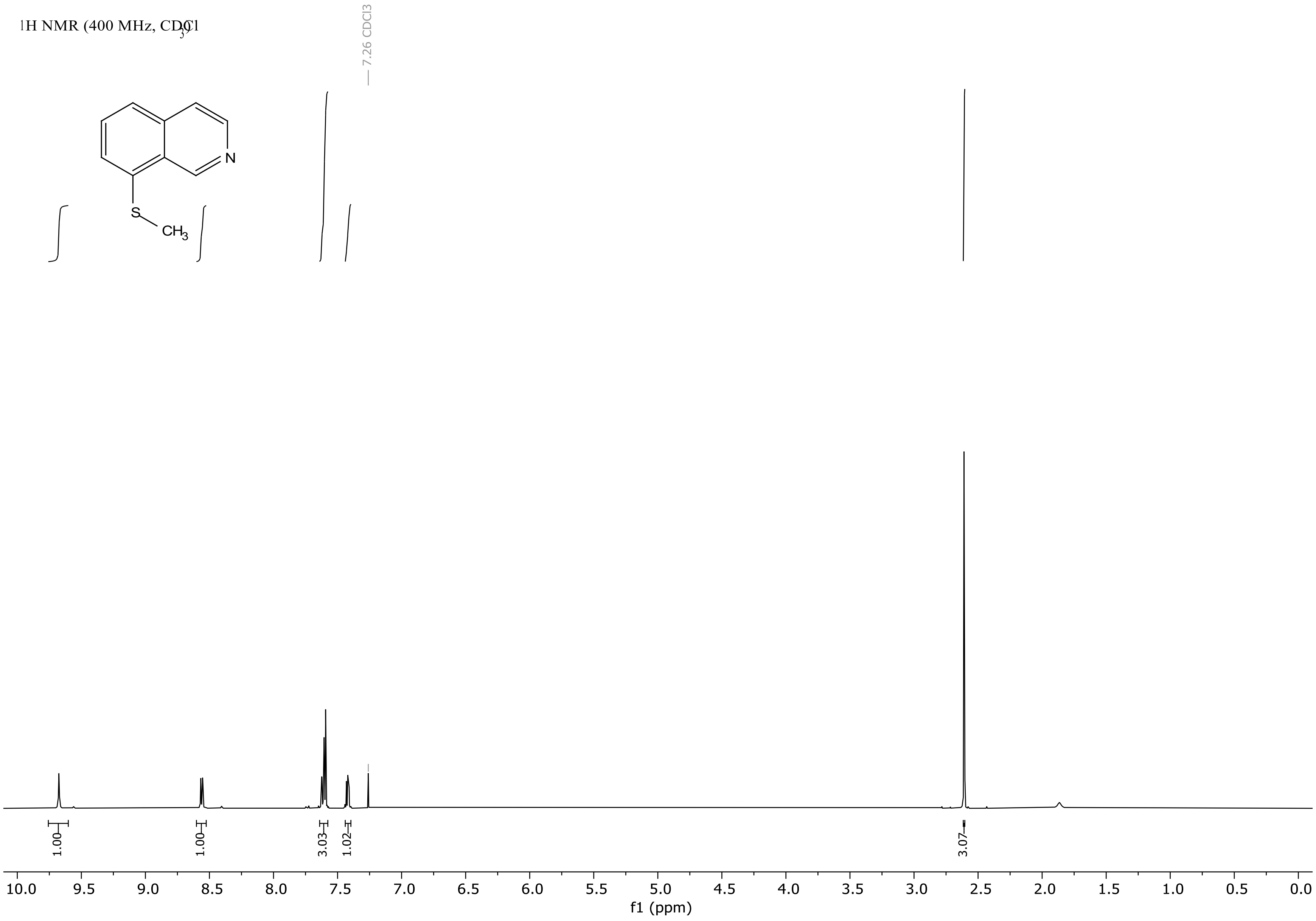

13C NMR (101 MHz, CDCl3)

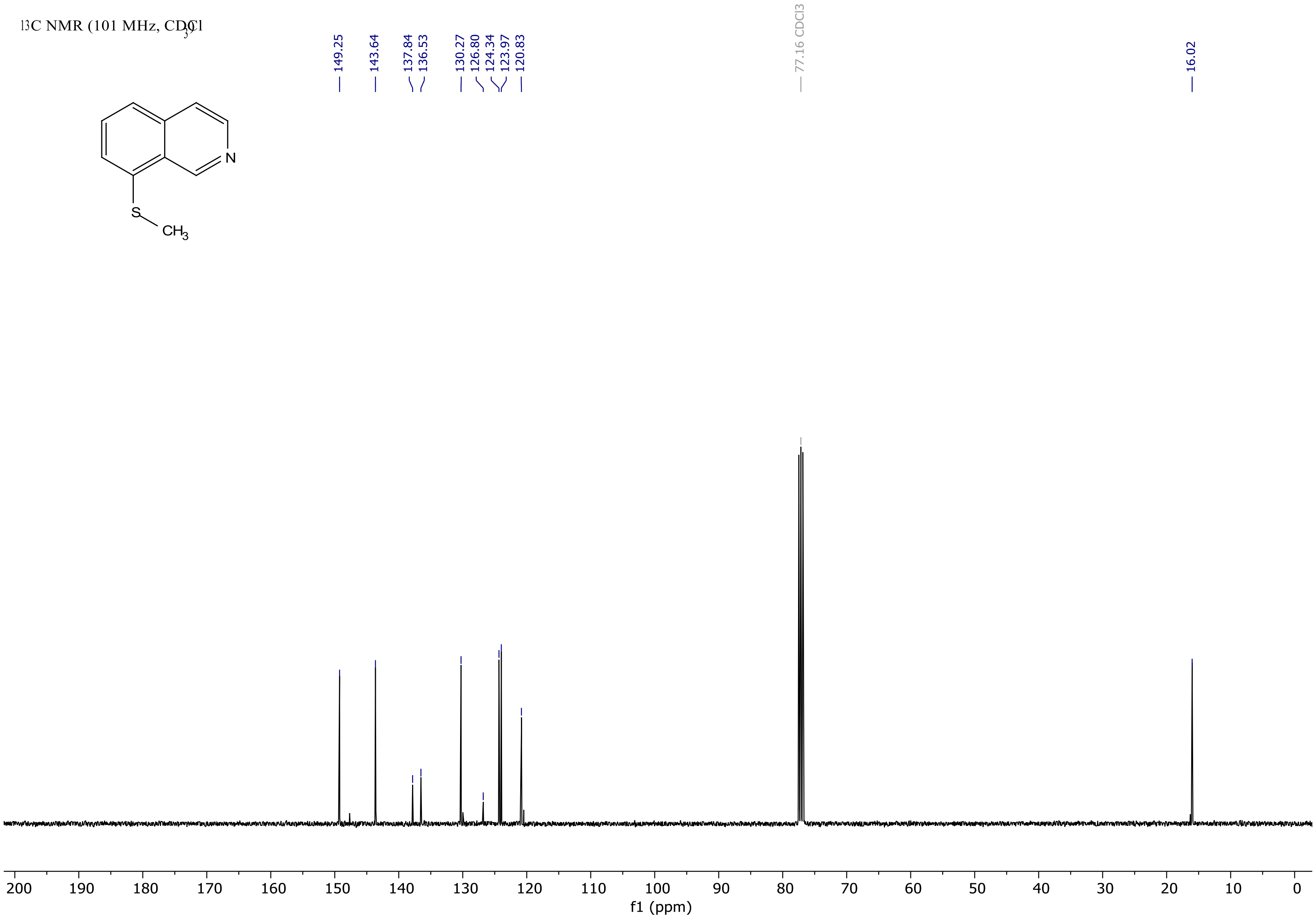

1H NMR (400 MHz, CDCl3)

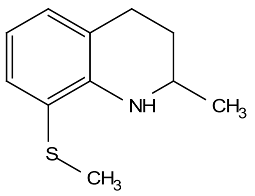

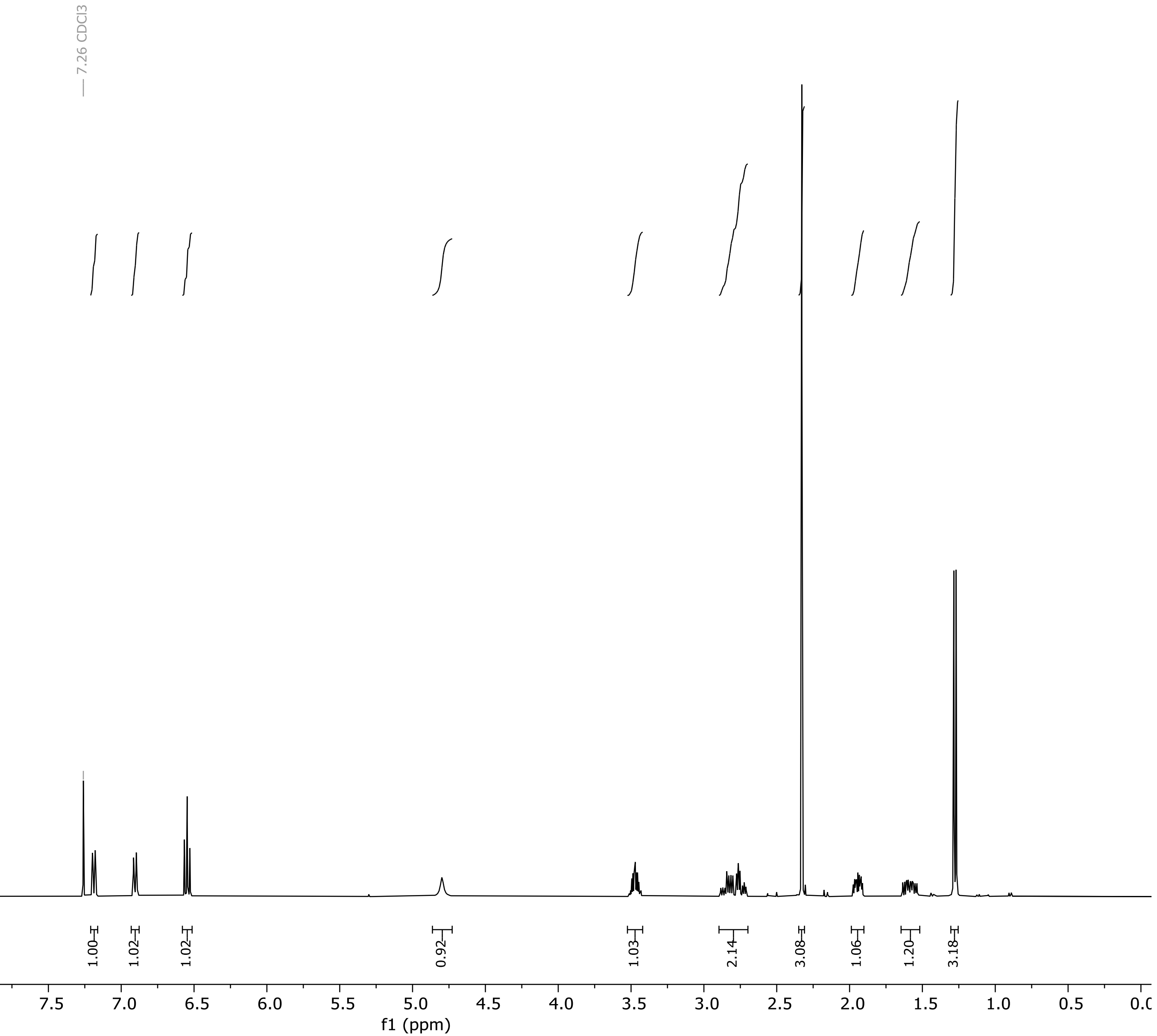

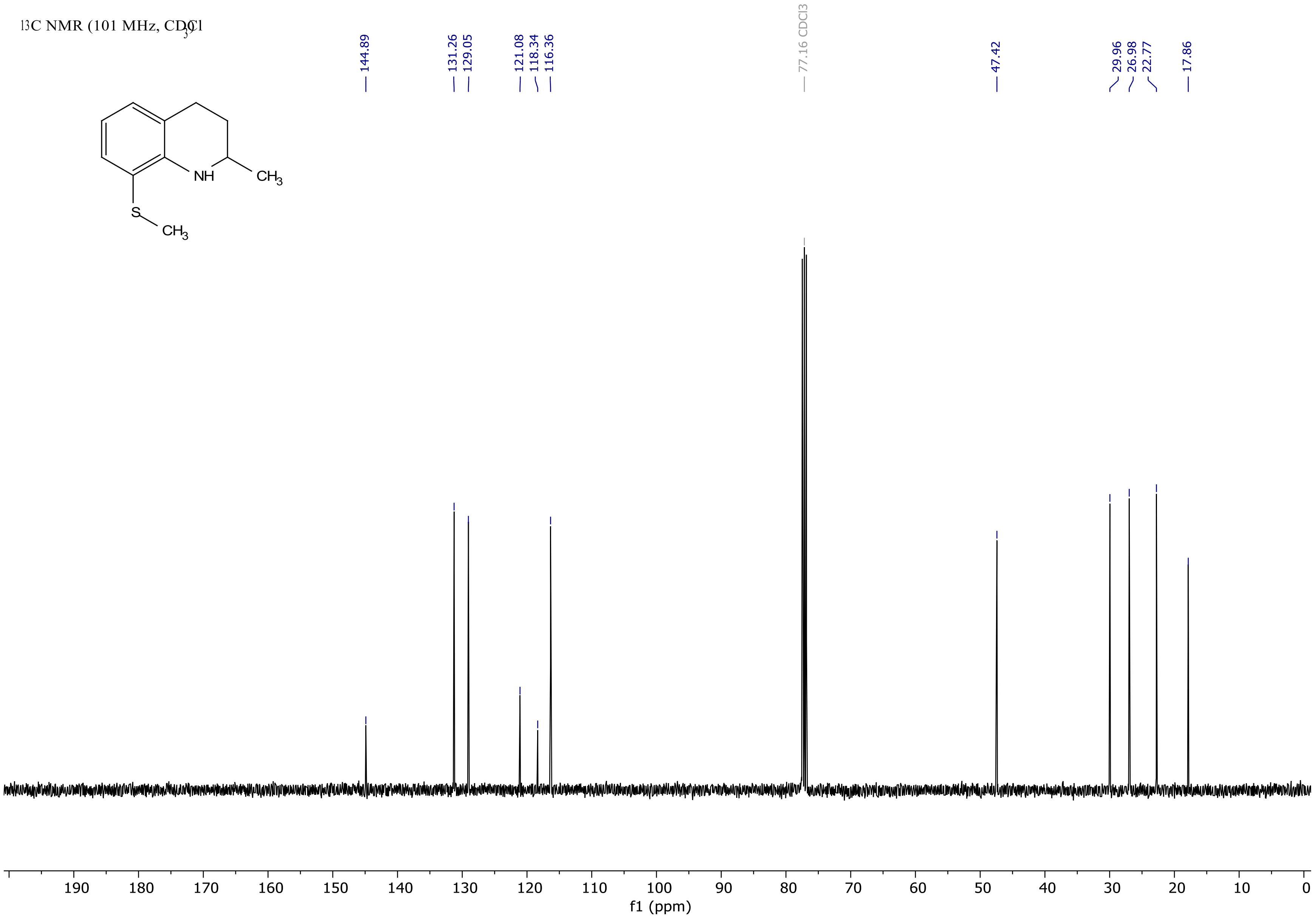
l3C NMR (101 MHz, CD3Cl
NH
CH3
S
CH3
144.89
131.26
129.05
121.08
118.34
116.36
77.16 CDCl3
47.42
29.96
26.98
22.77
17.86
190 180 170 160 150 140 130 120 110 100 90 80 70 60 50 40 30 20 10 0
f1 (ppm)

1H NMR (400 MHz, $CDCl_3$)

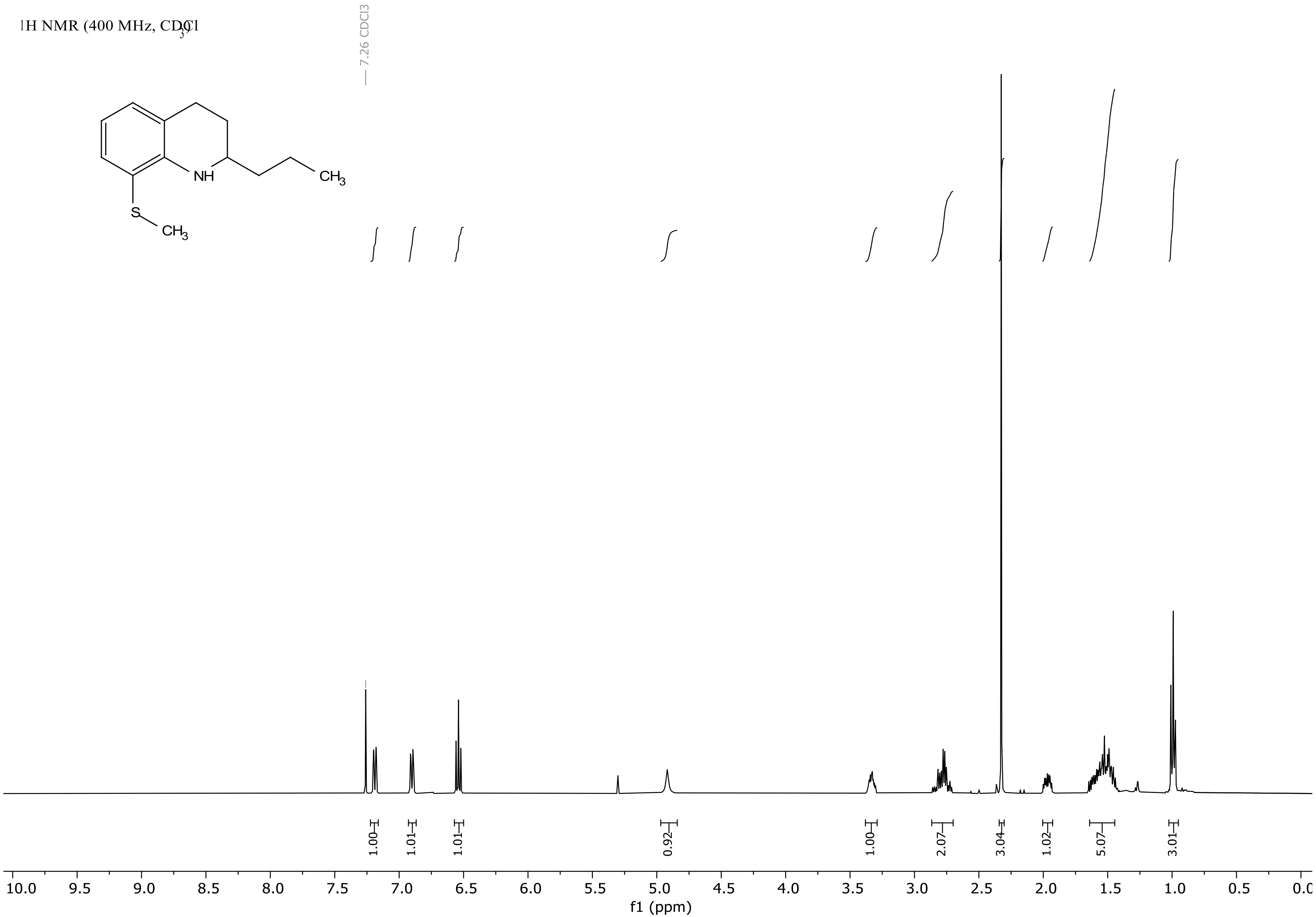

13C NMR (101 MHz, CDCl3)

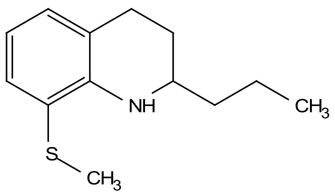

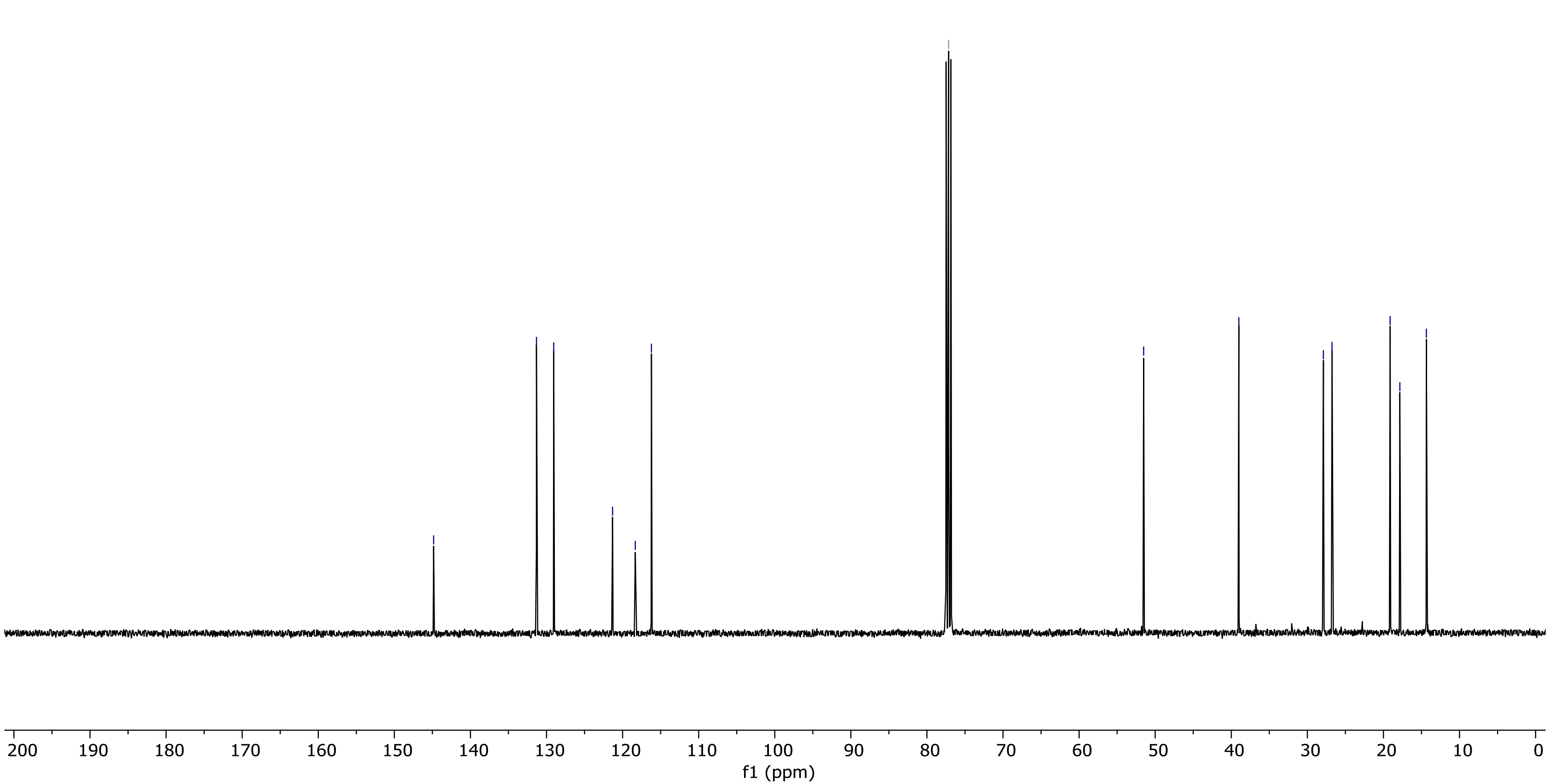

1H NMR (400 MHz, CDCl3)

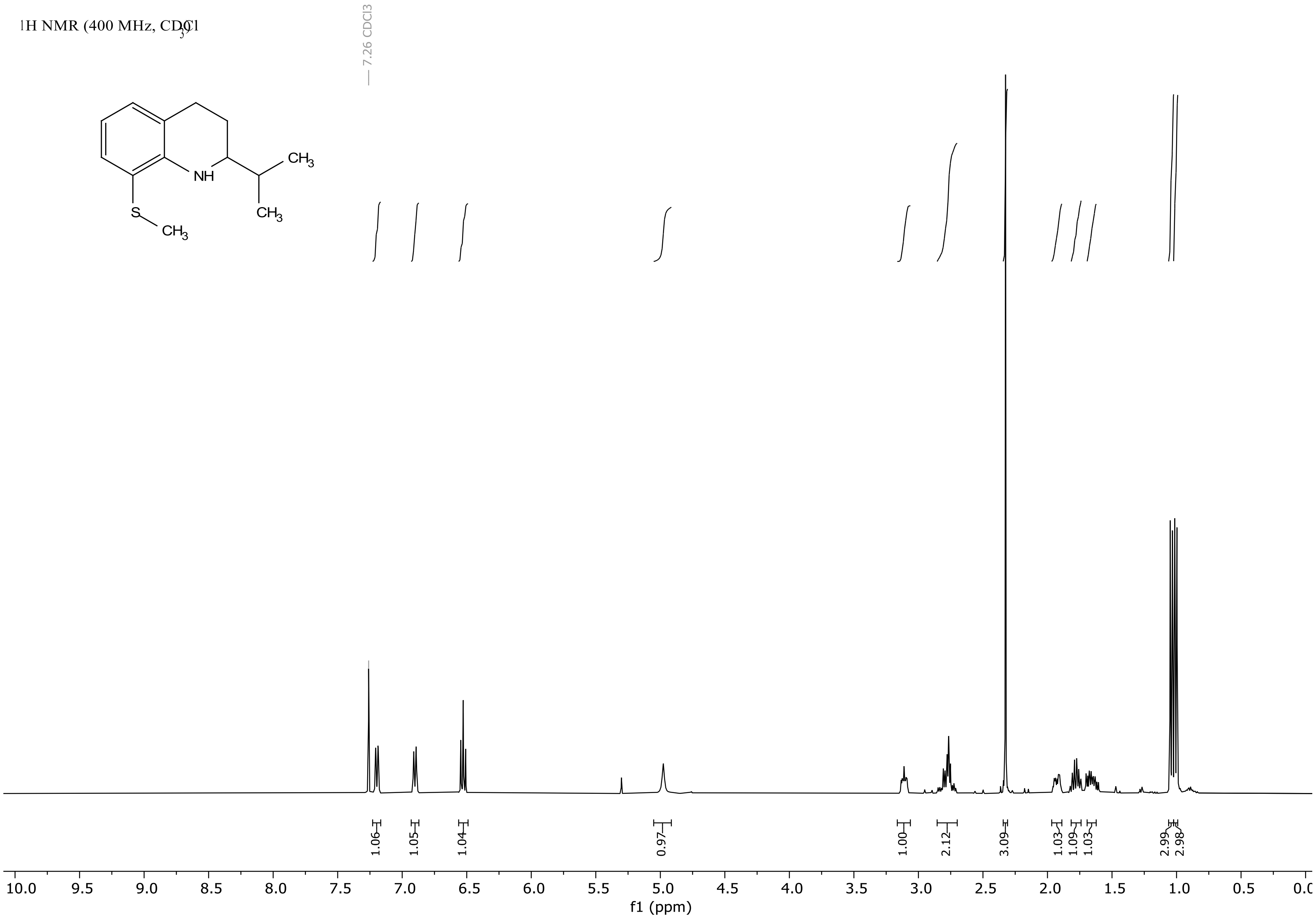

13C NMR (101 MHz, CDCl3)

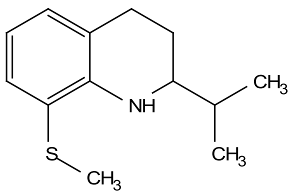

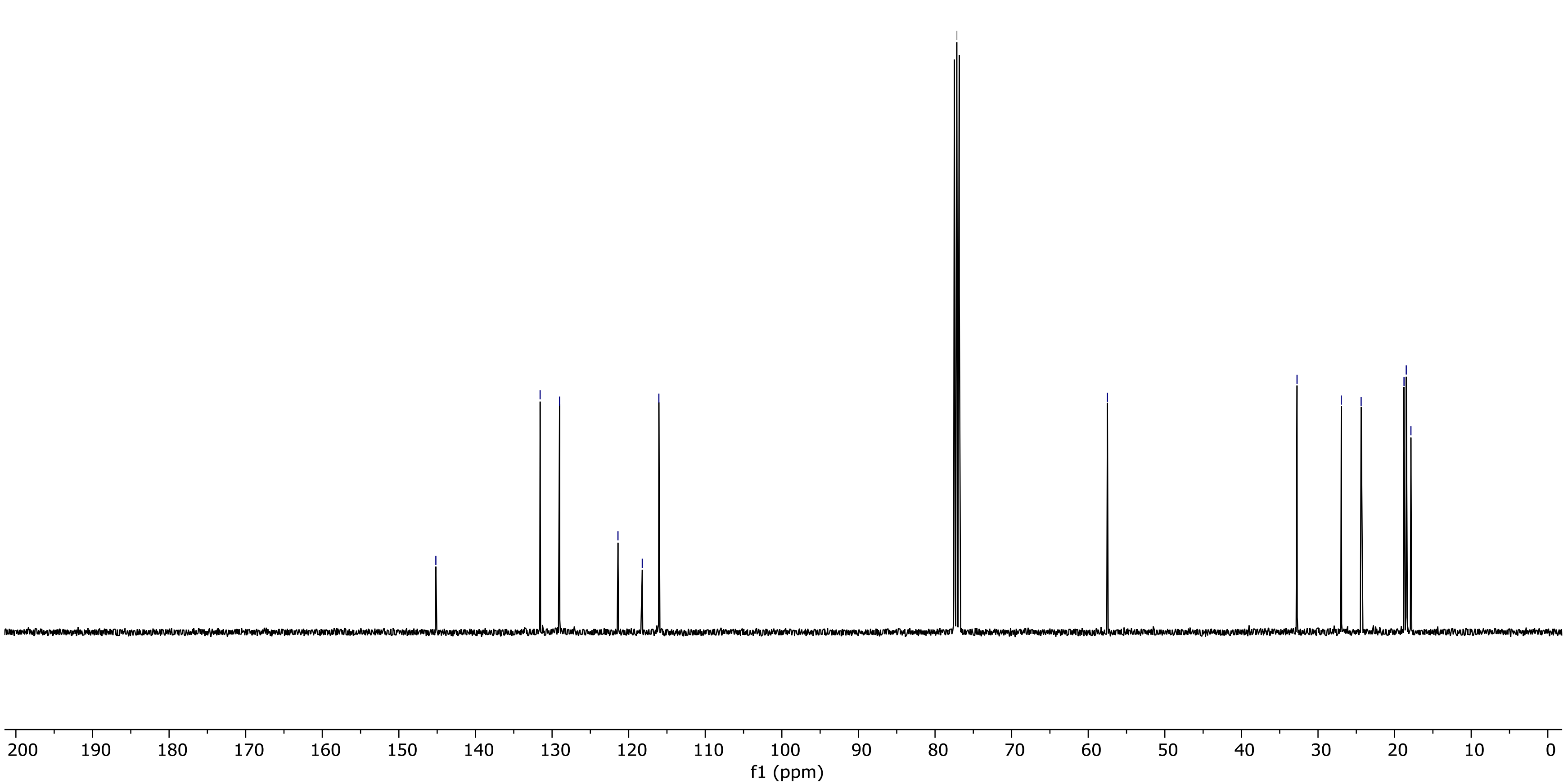

1H NMR (400 MHz, $CDCl_3$)

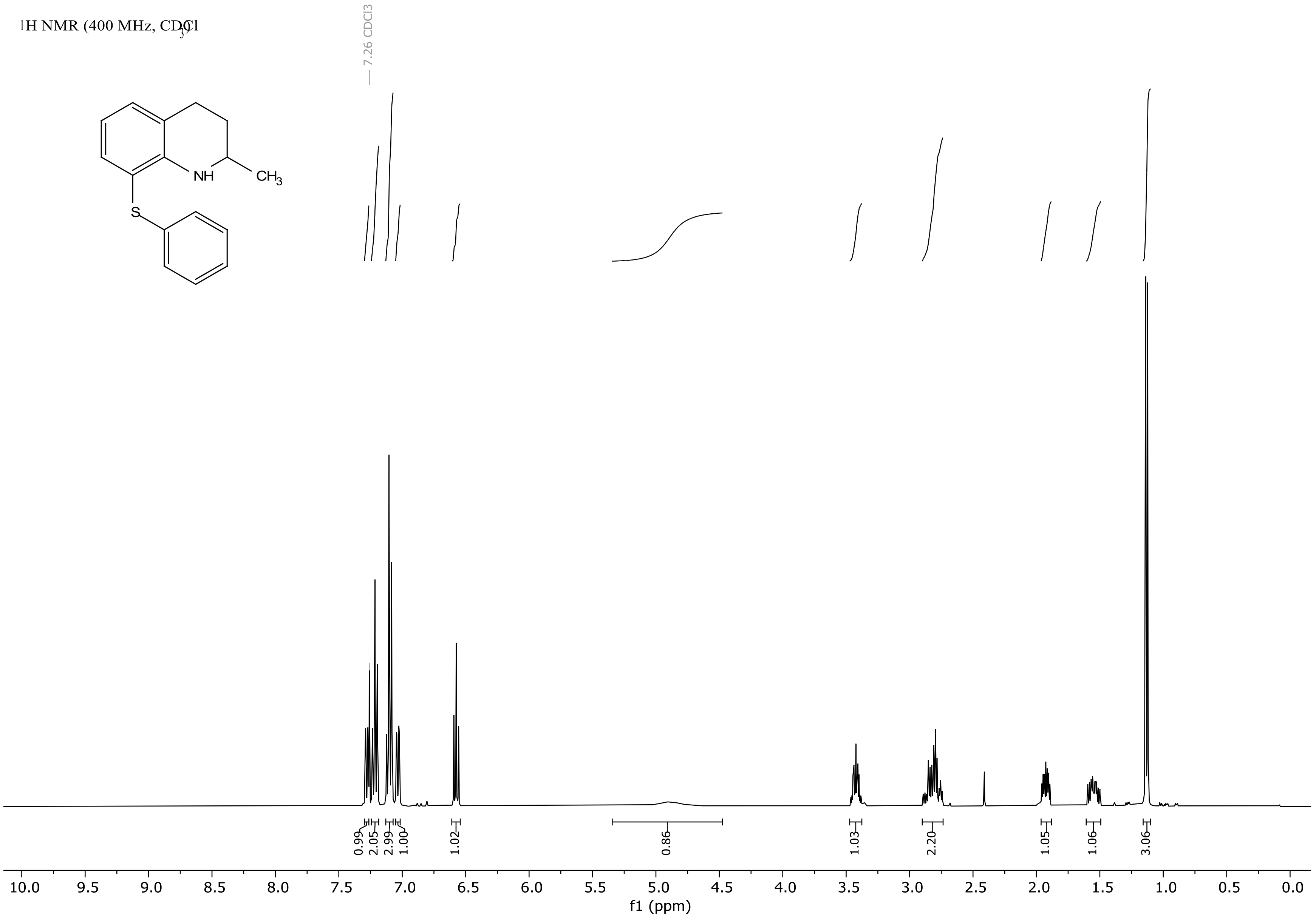

13C NMR (101 MHz, CDCl3)

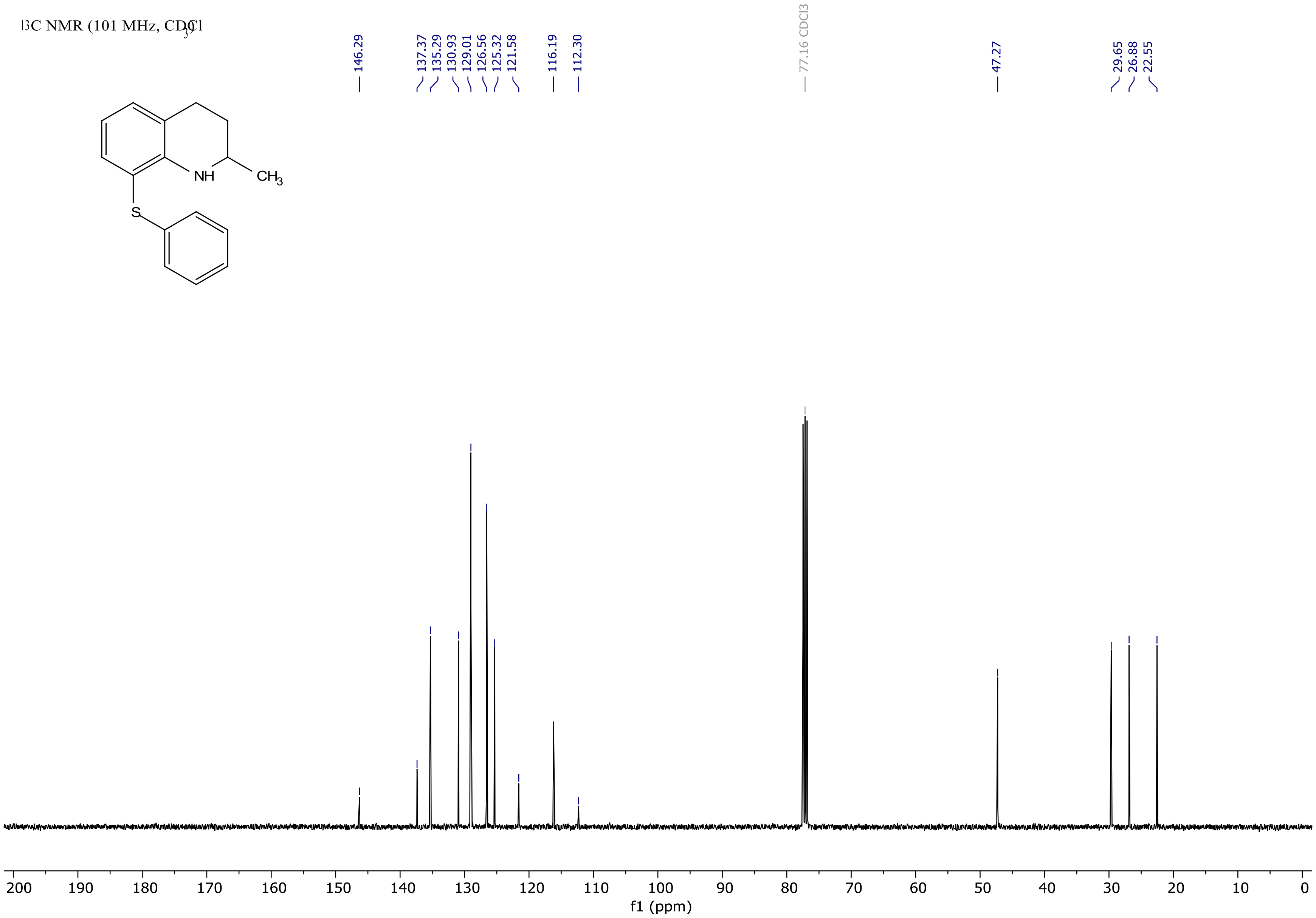

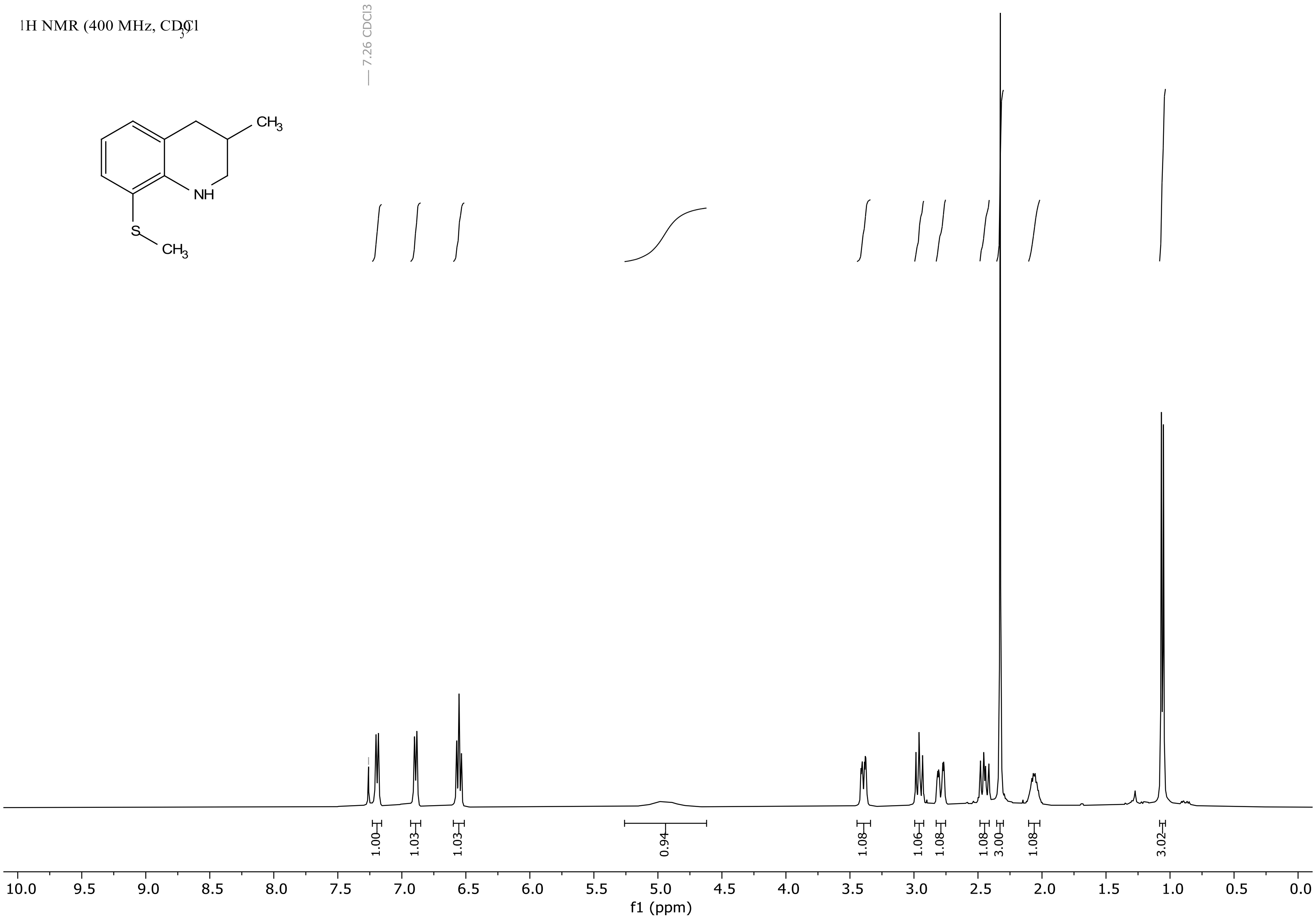
1H NMR (400 MHz, CDCl3)
7.26 CDCl3
CH3
NH
S
CH3
1.00
1.03
1.03
0.94
1.08
1.06
1.08
1.08
3.00
1.08
3.02
10.0 9.5 9.0 8.5 8.0 7.5 7.0 6.5 6.0 5.5 5.0 4.5 4.0 3.5 3.0 2.5 2.0 1.5 1.0 0.5 0.0
f1 (ppm)

13C NMR (101 MHz, CDCl3)

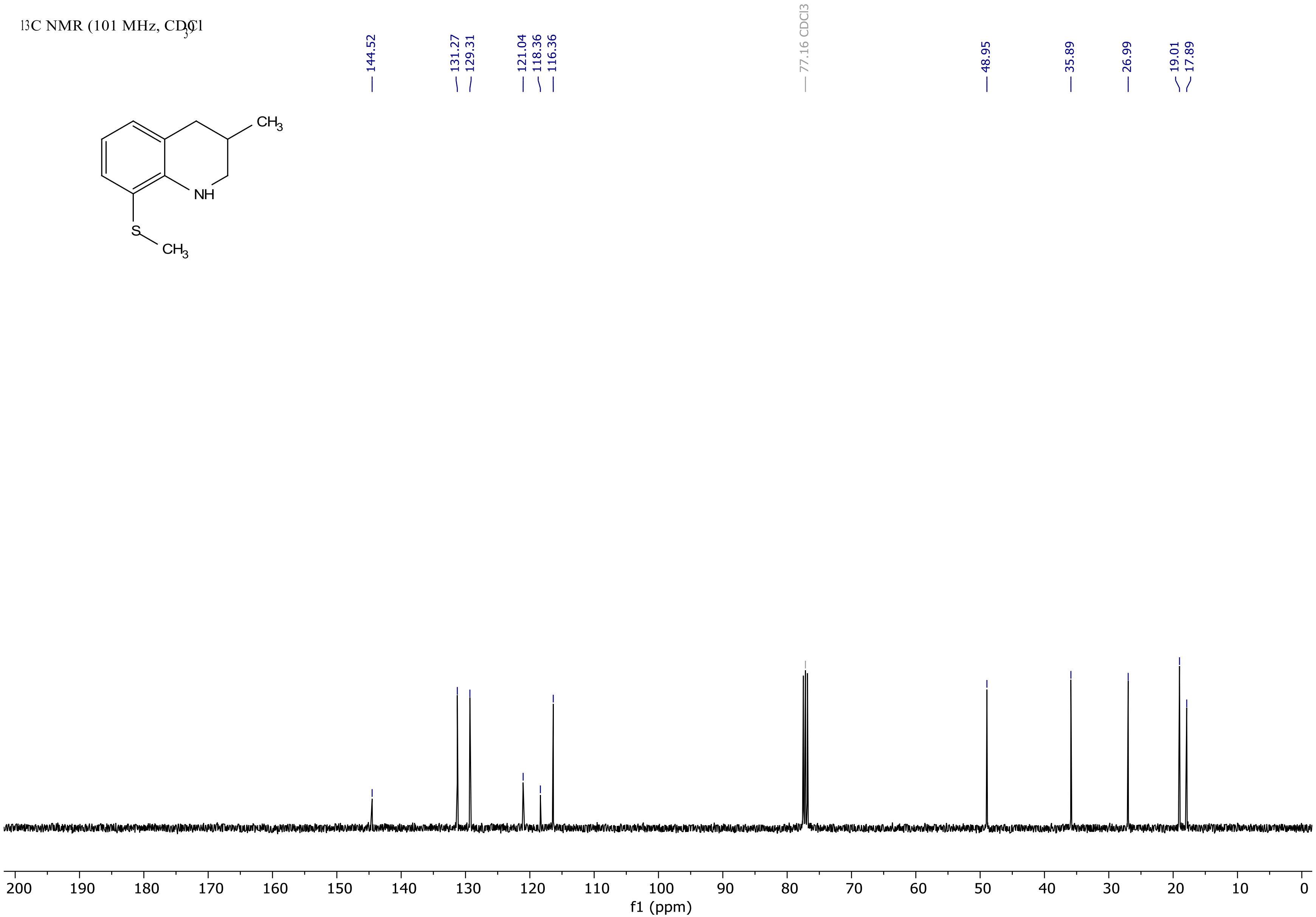

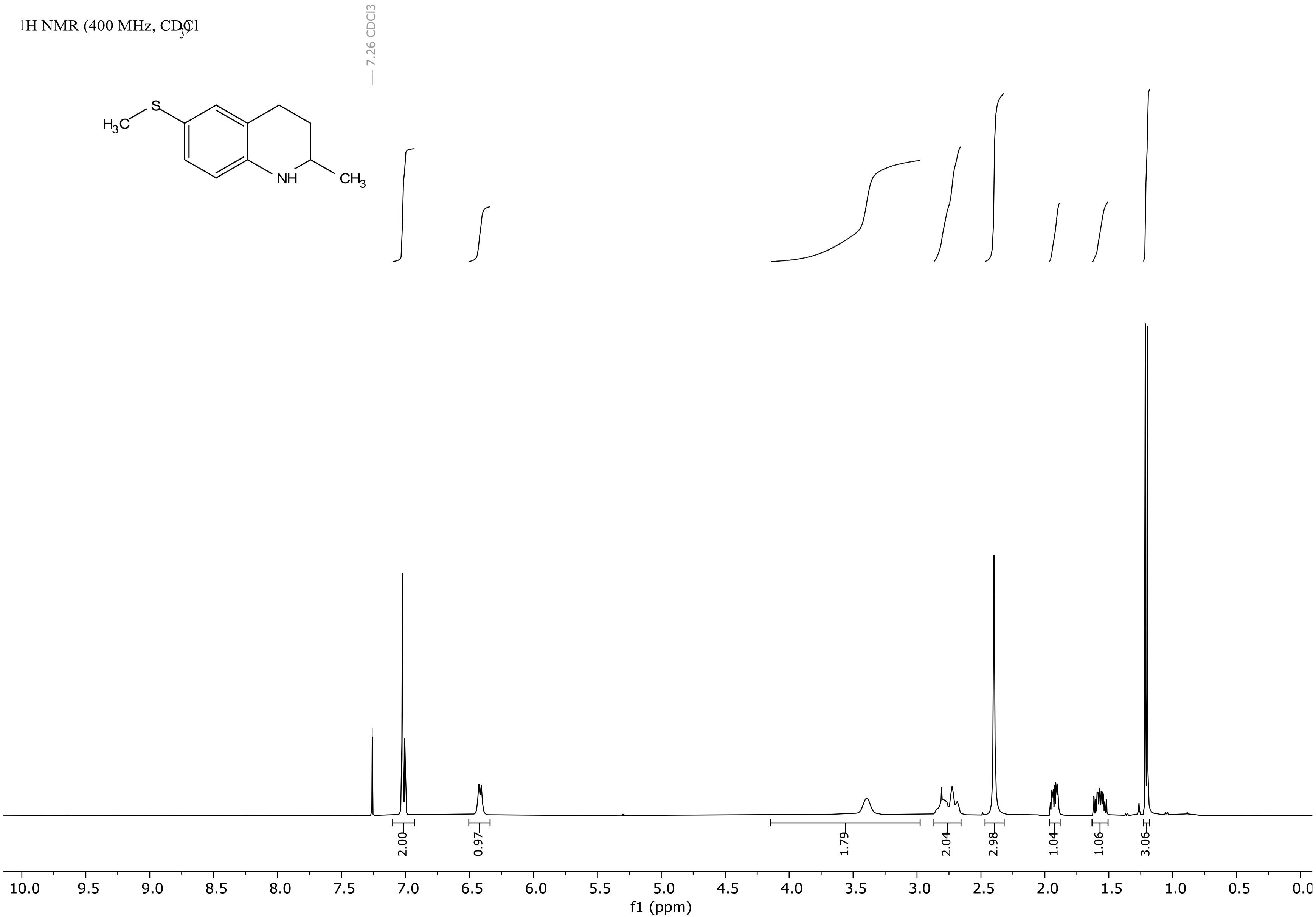
1H NMR (400 MHz, CDCl3)
7.26 CDCl3
S
H3C
NH
CH3
2.00
0.97
1.79
2.04
2.98
1.04
1.06
3.06
10.0 9.5 9.0 8.5 8.0 7.5 7.0 6.5 6.0 5.5 5.0 4.5 4.0 3.5 3.0 2.5 2.0 1.5 1.0 0.5 0.0
f1 (ppm)

13C NMR (101 MHz, $CDCl_3$)

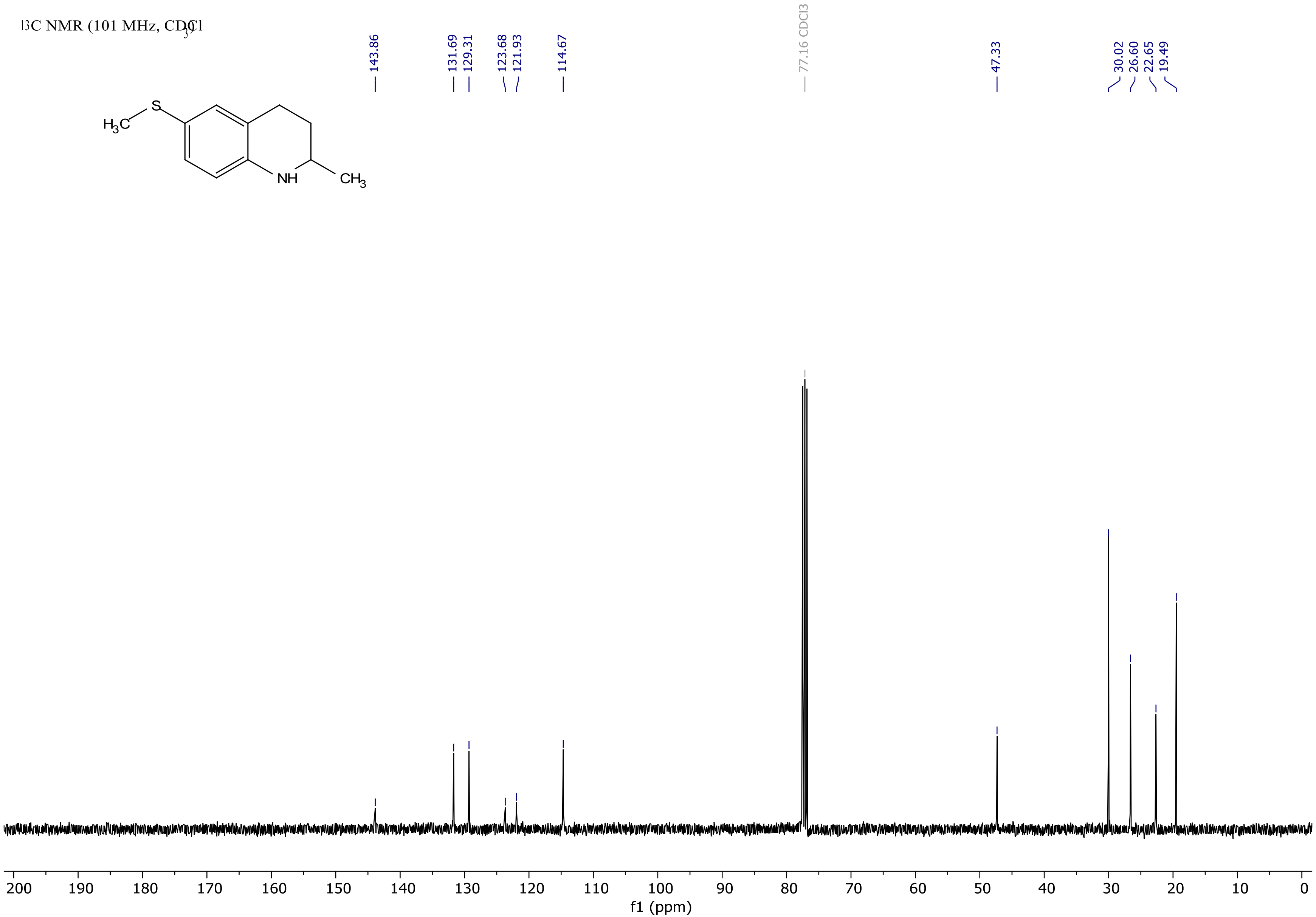

1H NMR (400 MHz, CDCl3)

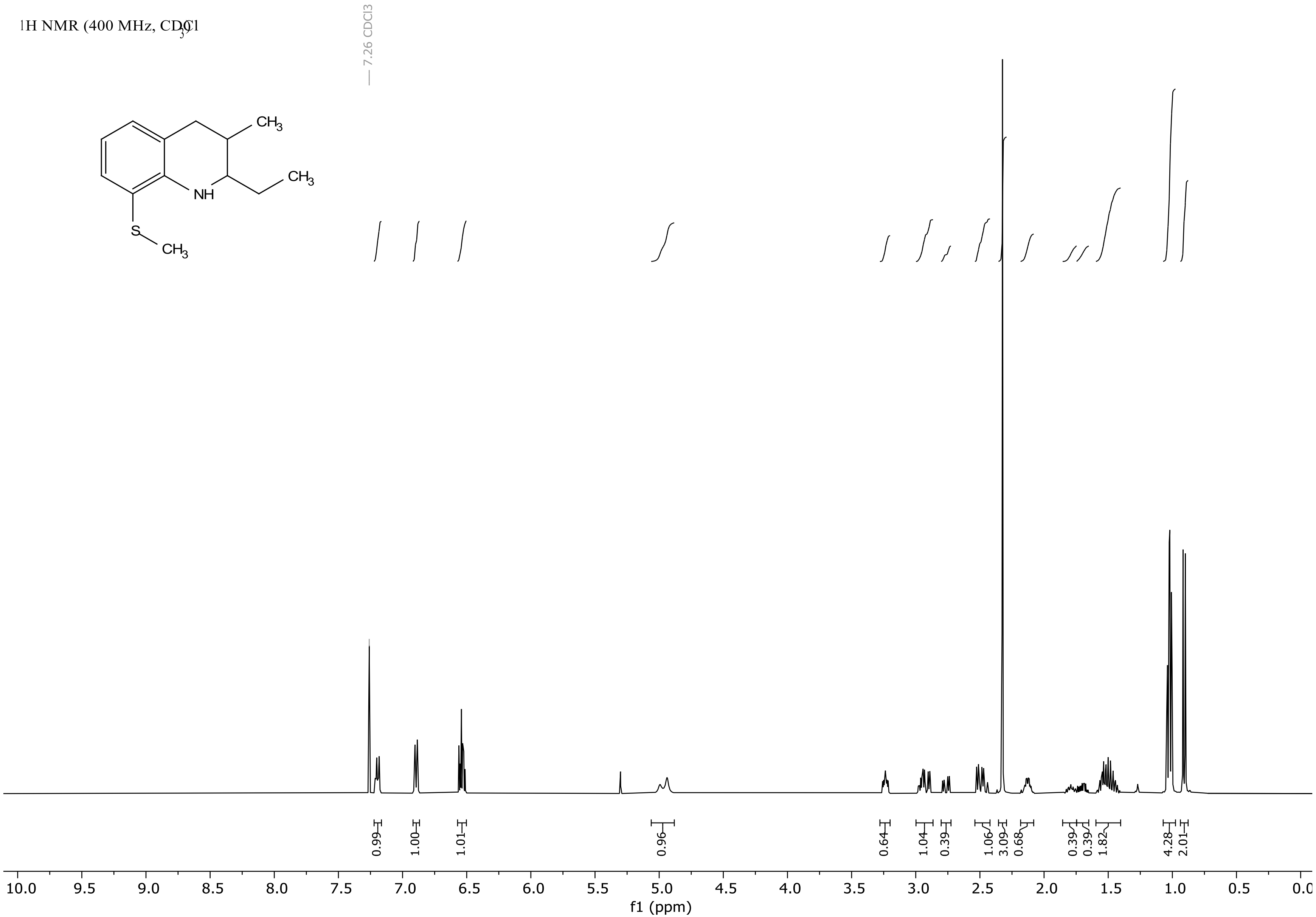

13C NMR (101 MHz, CDCl3)

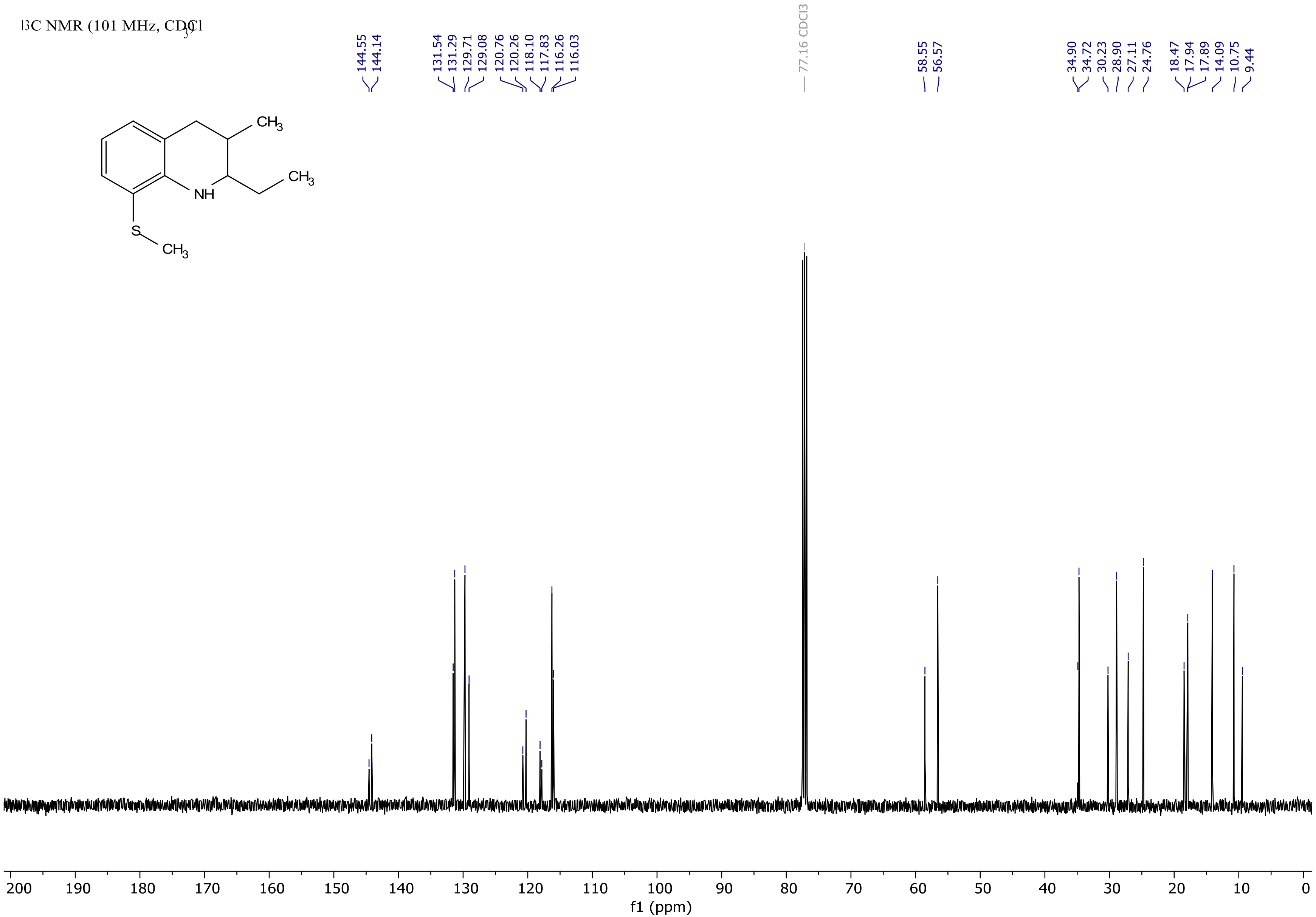

1H NMR (400 MHz, CDCl3)

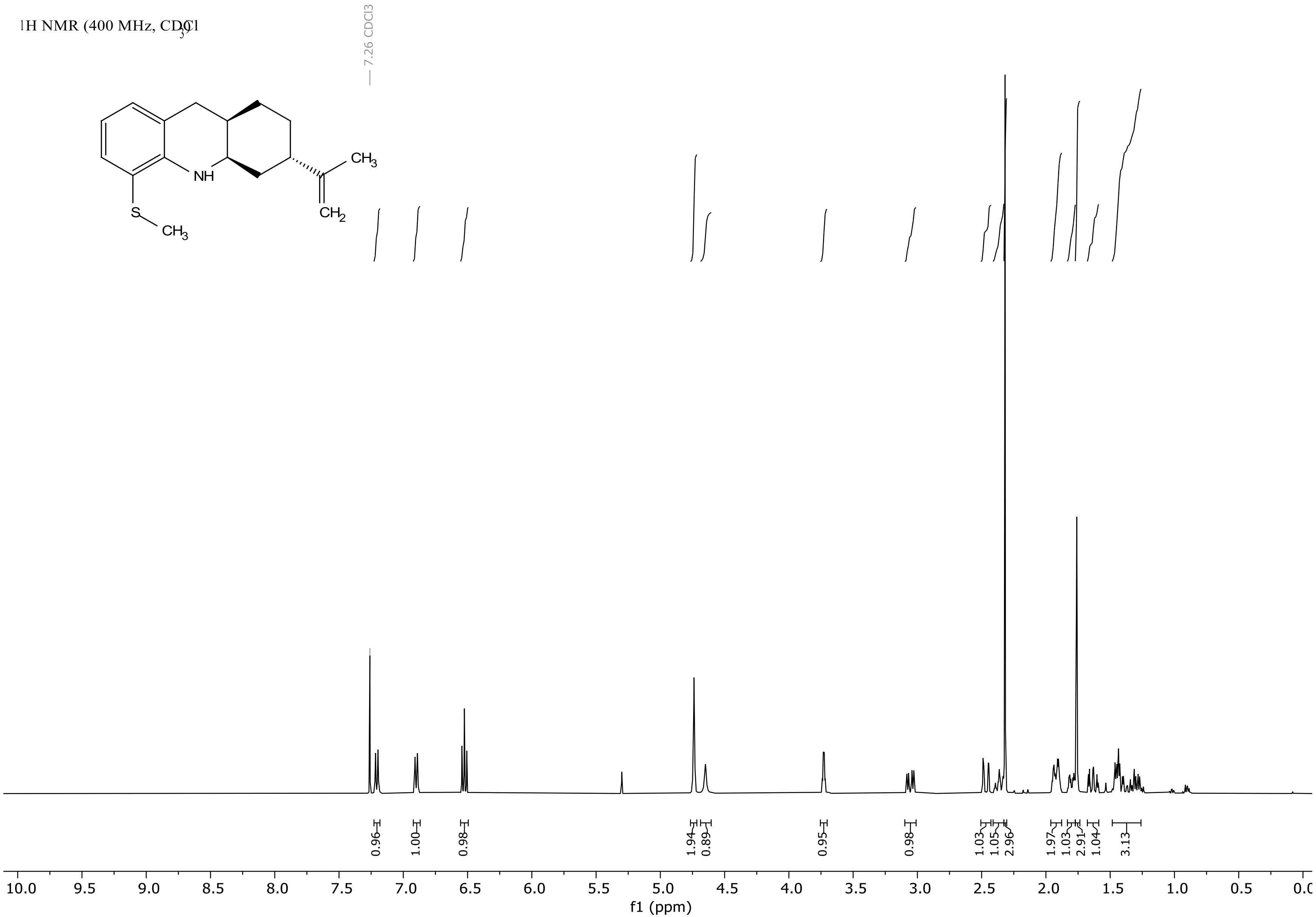

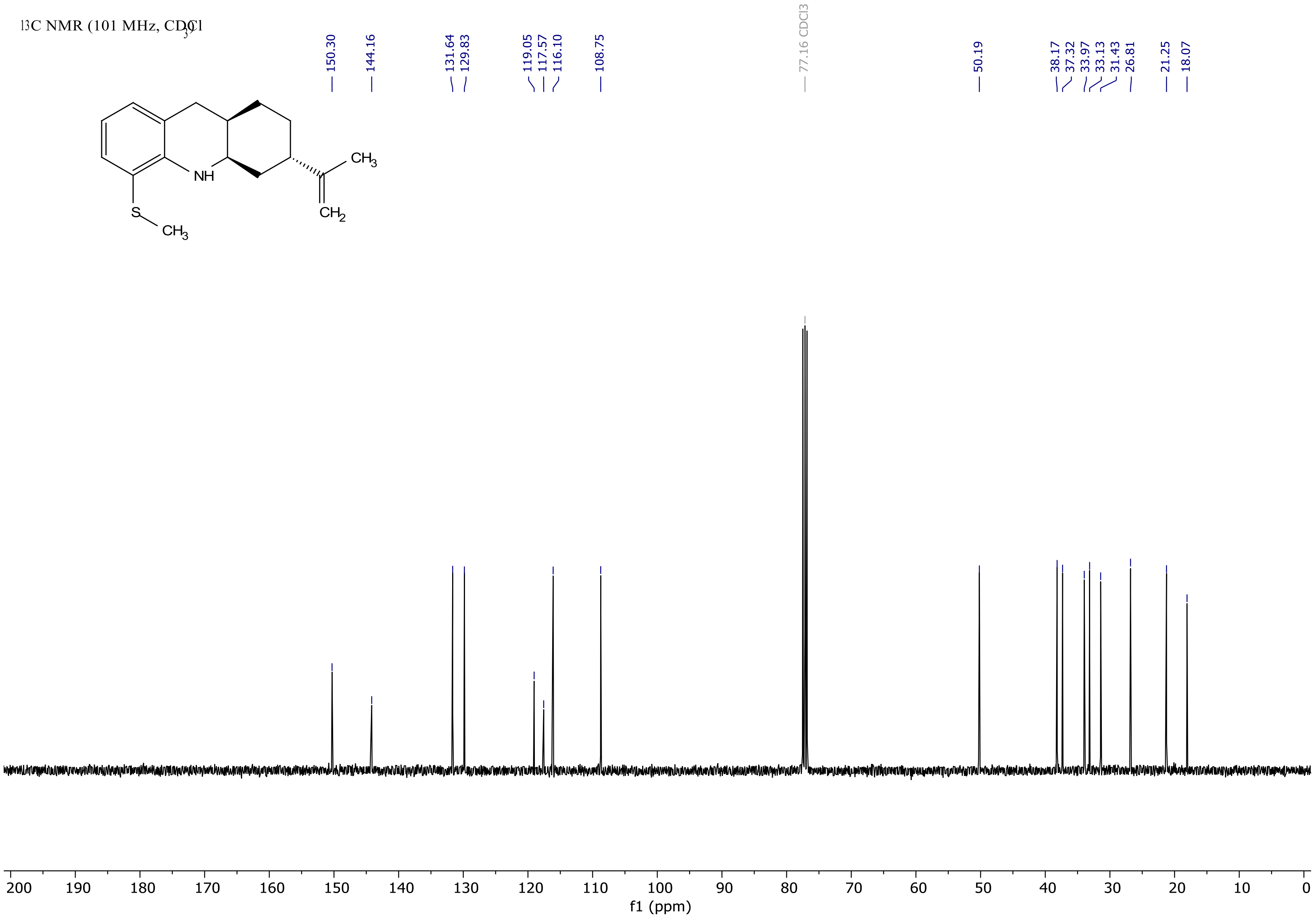
13C NMR (101 MHz, CDCl3)
150.30
144.16
131.64
129.83
119.05
117.57
116.10
108.75
77.16 CDCl3
50.19
38.17
37.32
33.97
33.13
31.43
26.81
21.25
18.07
NH
S
CH3
CH3
CH2
200 190 180 170 160 150 140 130 120 110 100 90 80 70 60 50 40 30 20 10 0
f1 (ppm)

1H NMR (400 MHz, CDCl3)

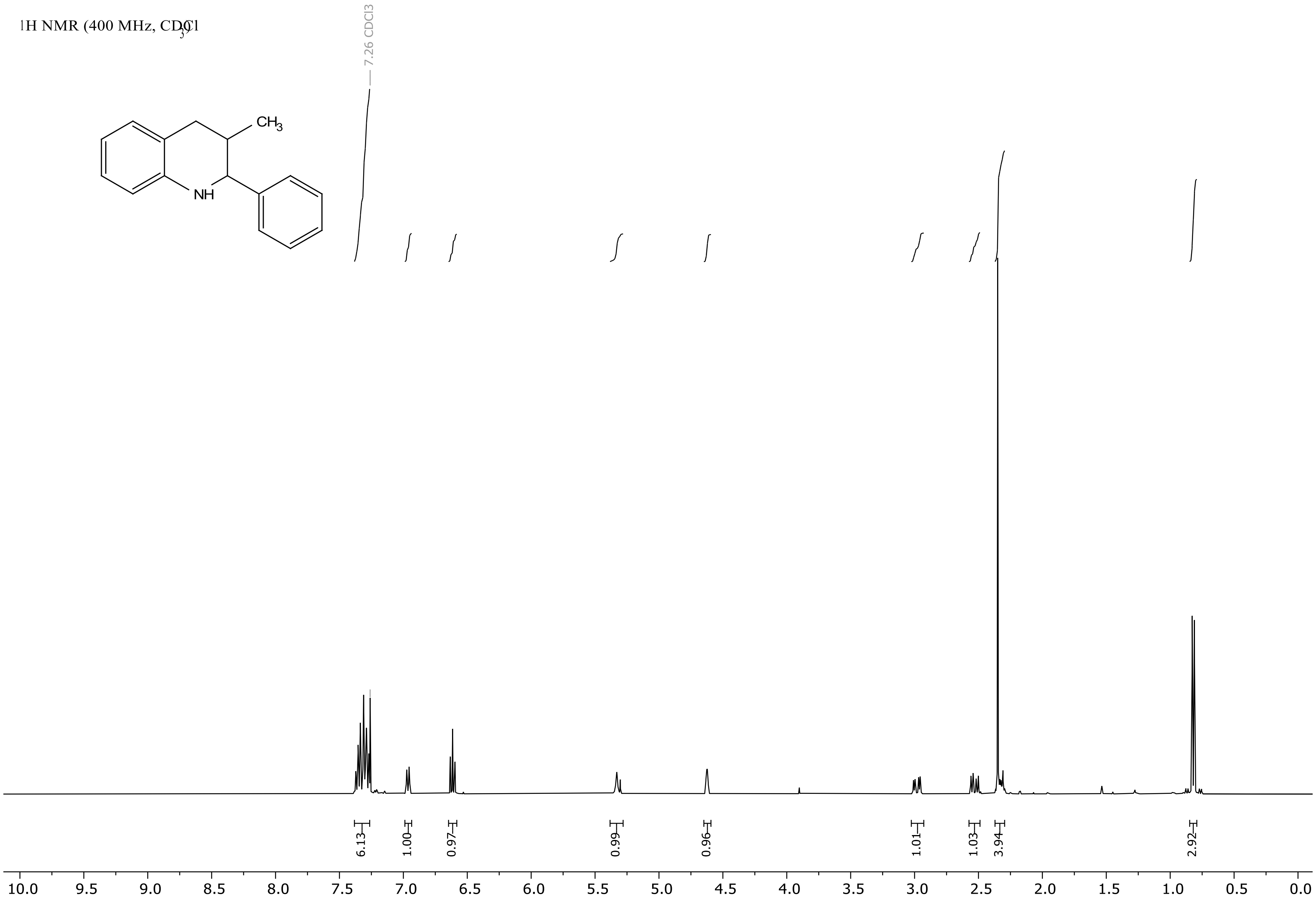

13C NMR (101 MHz, CDCl3)

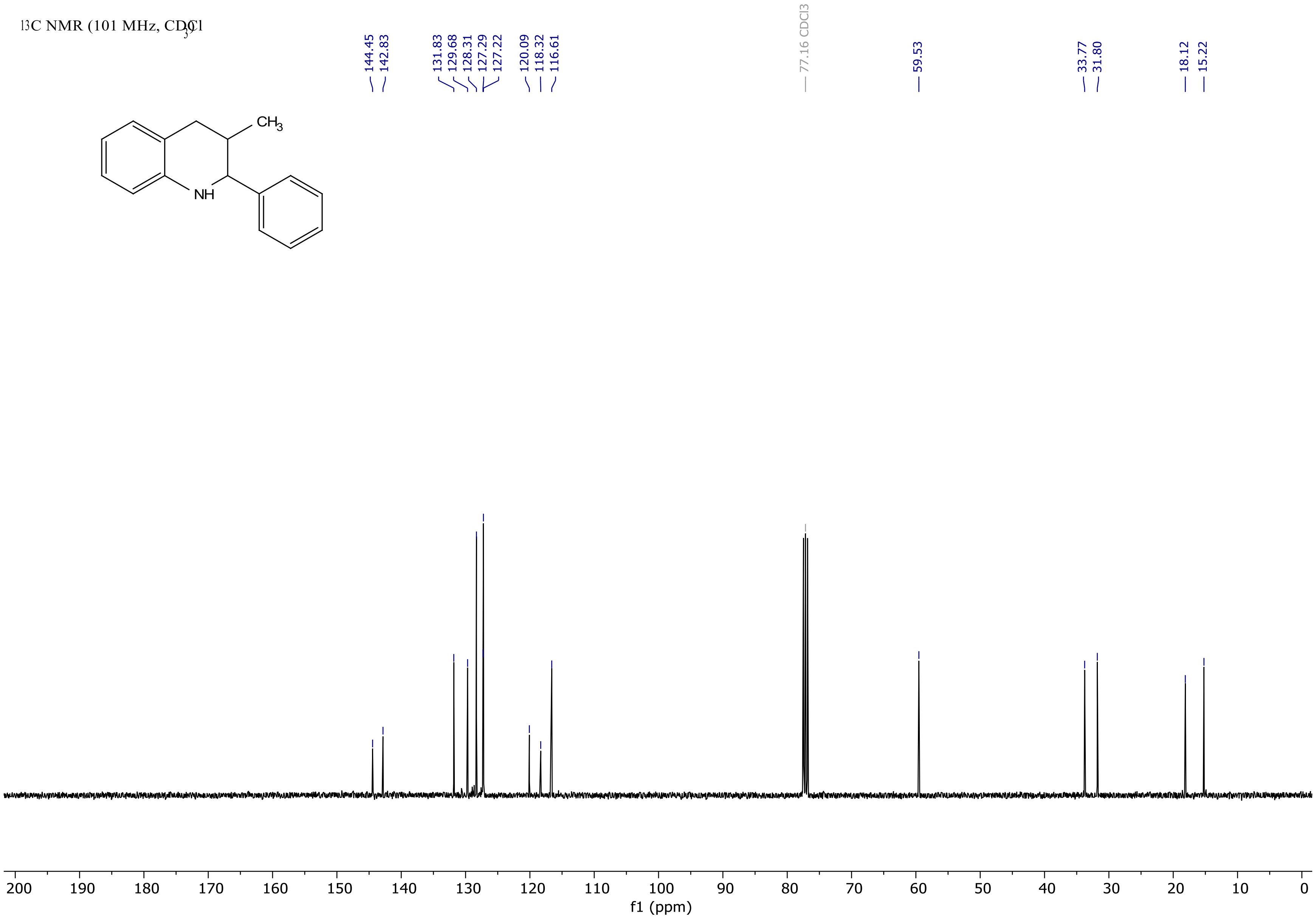

1H NMR (400 MHz, CDCl3)

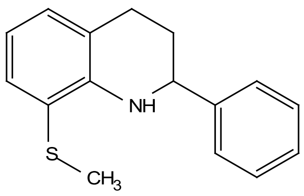

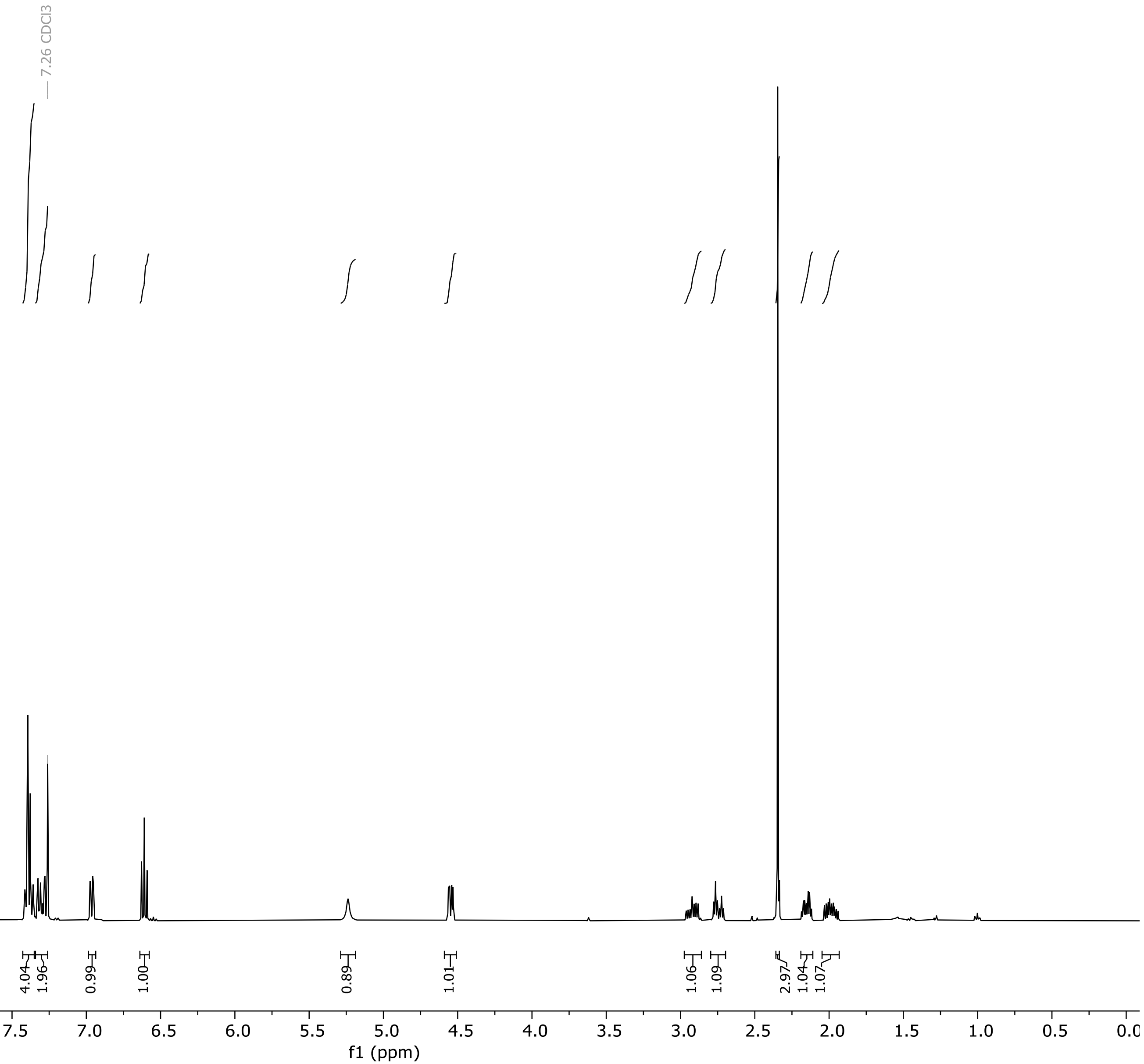

13C NMR (101 MHz, CDCl3)

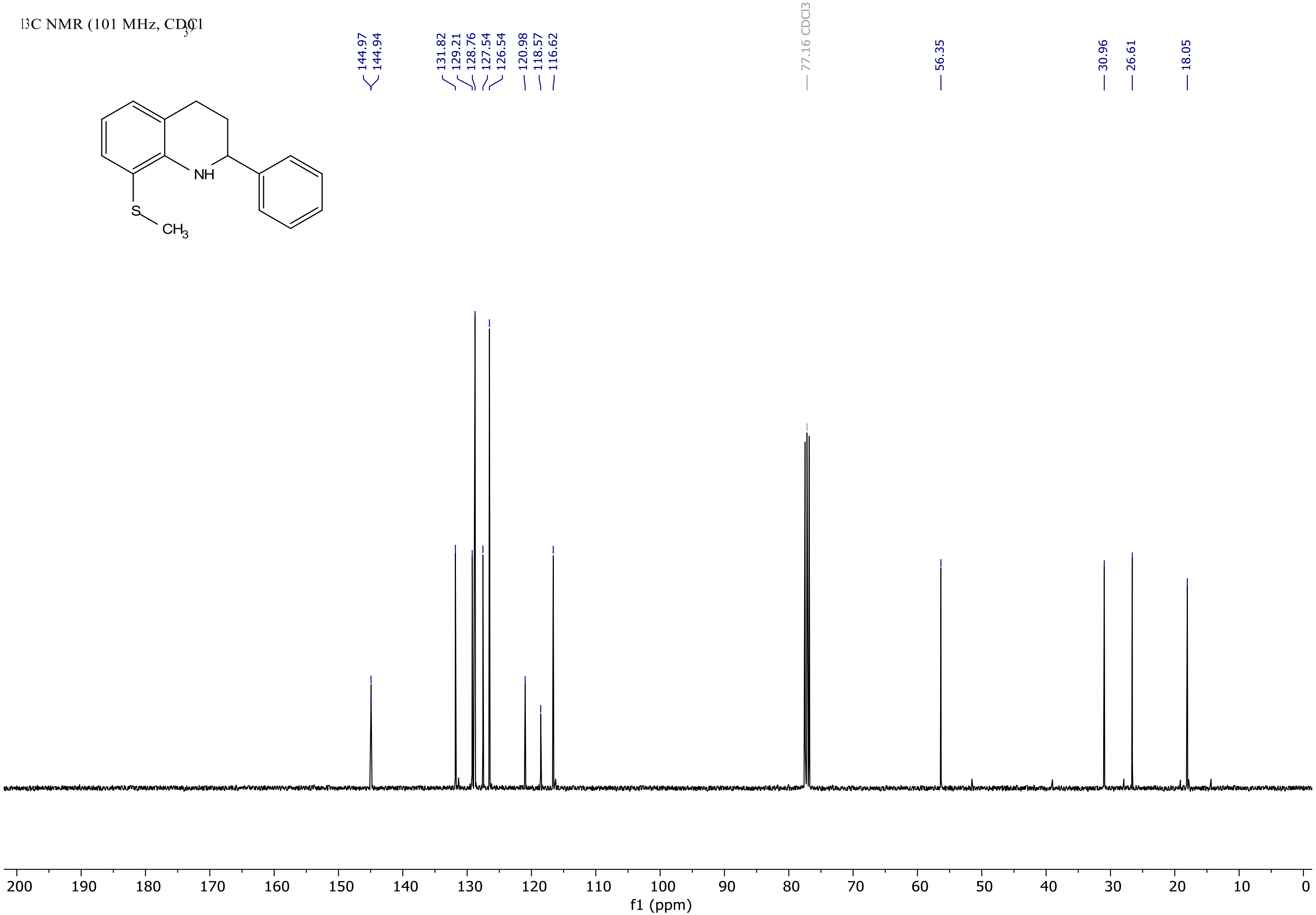

1H NMR (400 MHz, CDCl3)

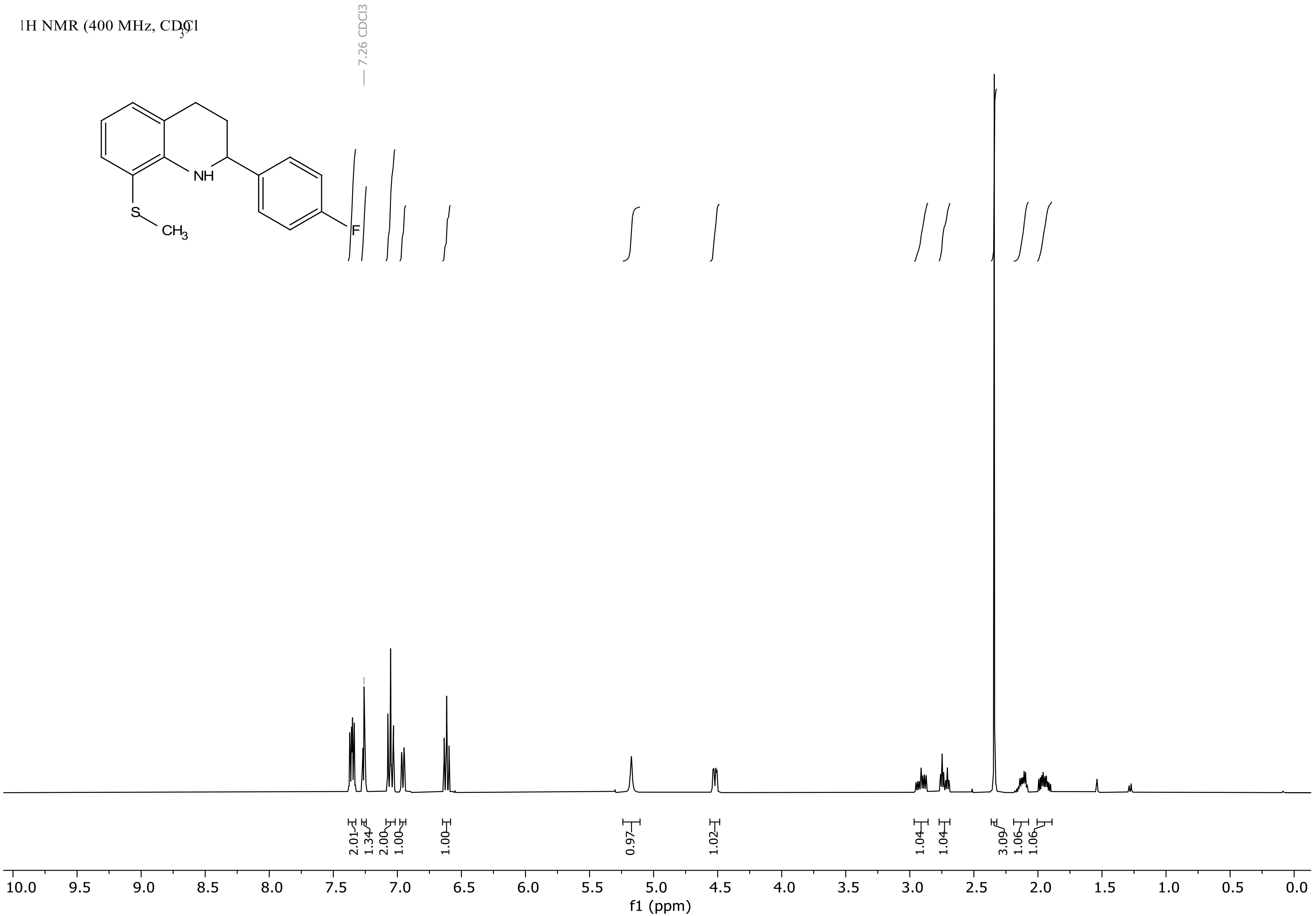

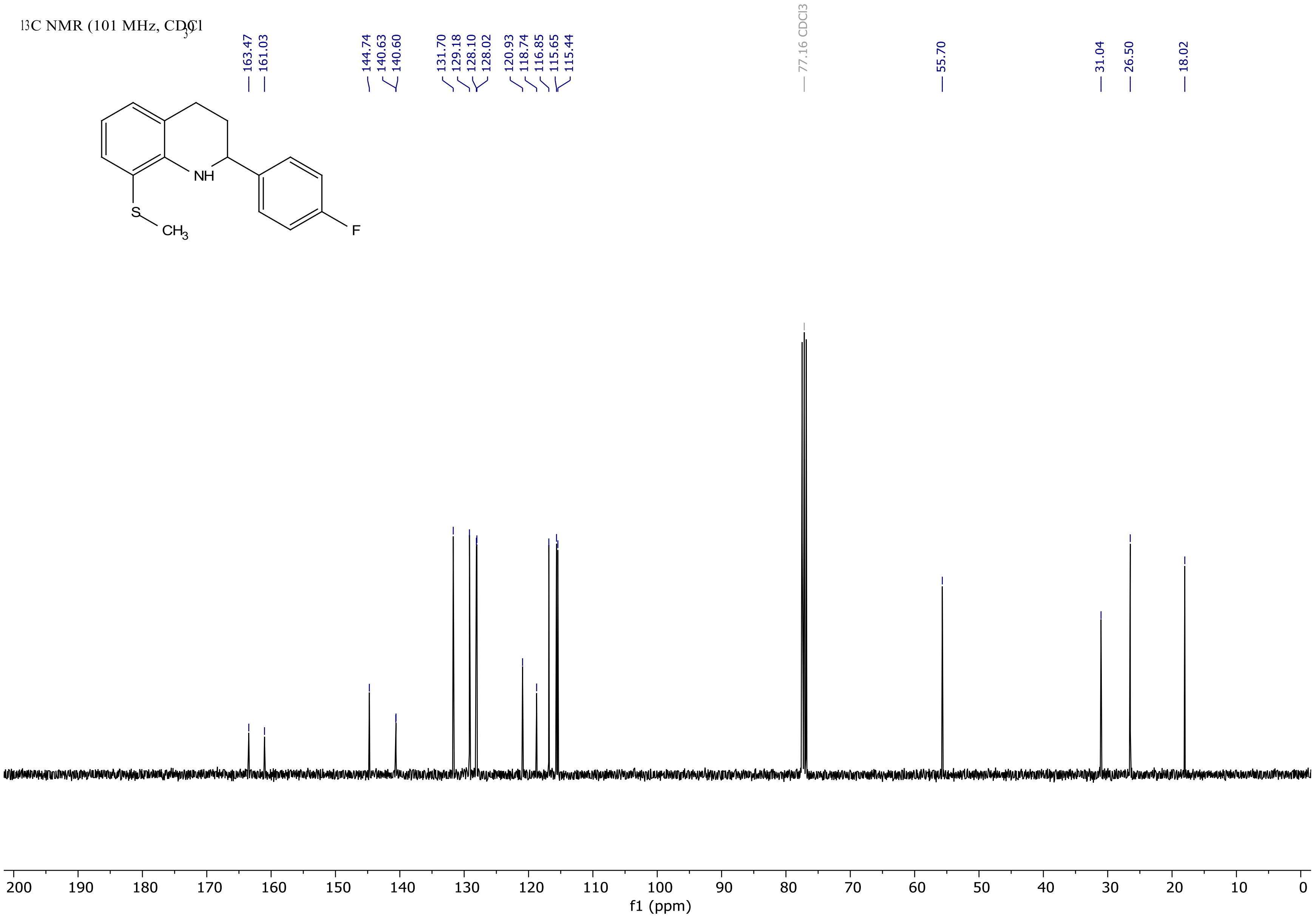
13C NMR (101 MHz, CDCl3)
163.47
161.03
144.74
140.63
140.60
131.70
129.18
128.10
128.02
120.93
118.74
116.85
115.65
115.44
77.16 CDCl3
55.70
31.04
26.50
18.02
NH
S
CH3
F
200 190 180 170 160 150 140 130 120 110 100 90 80 70 60 50 40 30 20 10 0
f1 (ppm)

19F NMR (376 MHz, $CDCl_3$)

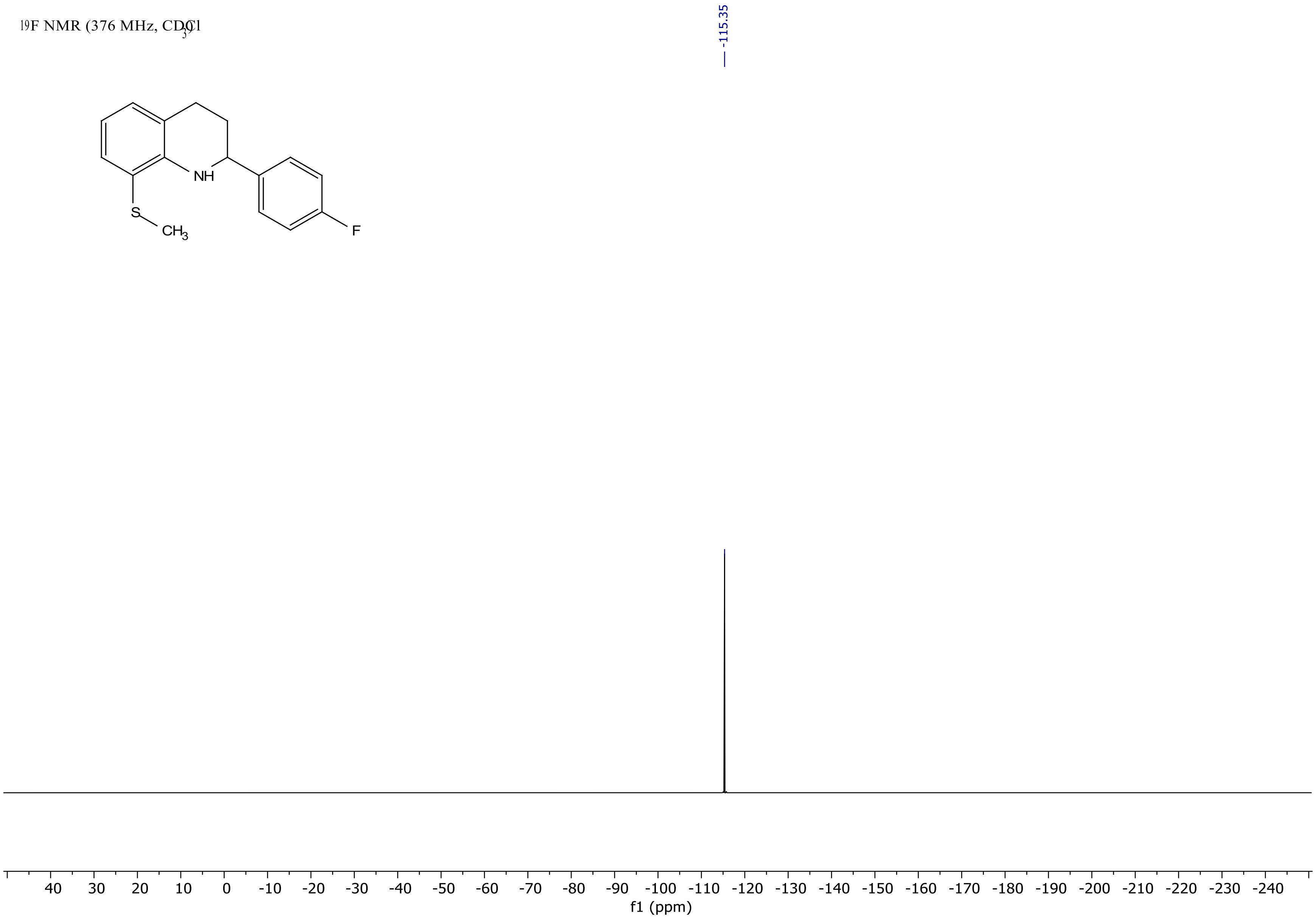

1H NMR (400 MHz, CDCl3)

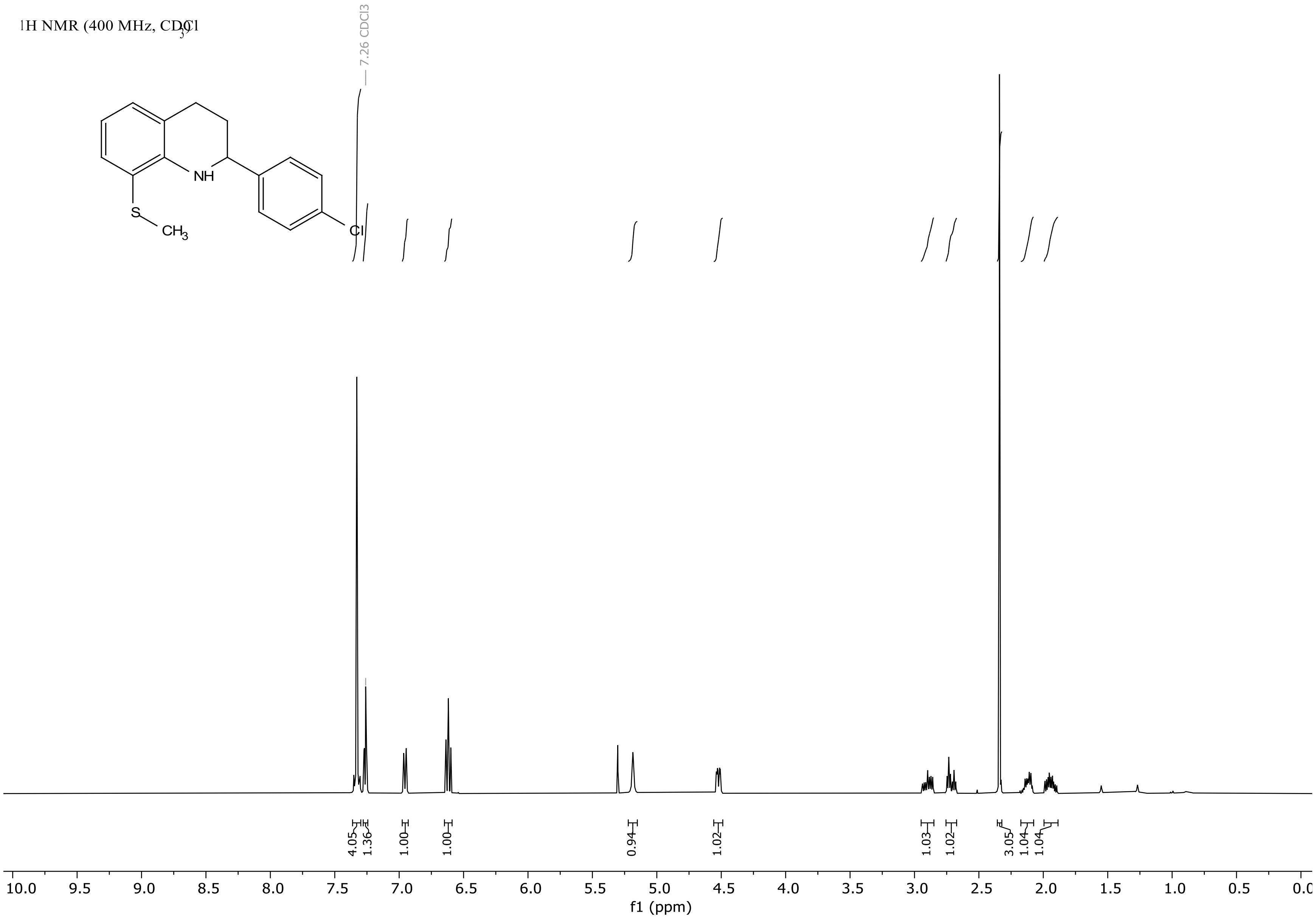

13C NMR (101 MHz, $CDCl_3$)

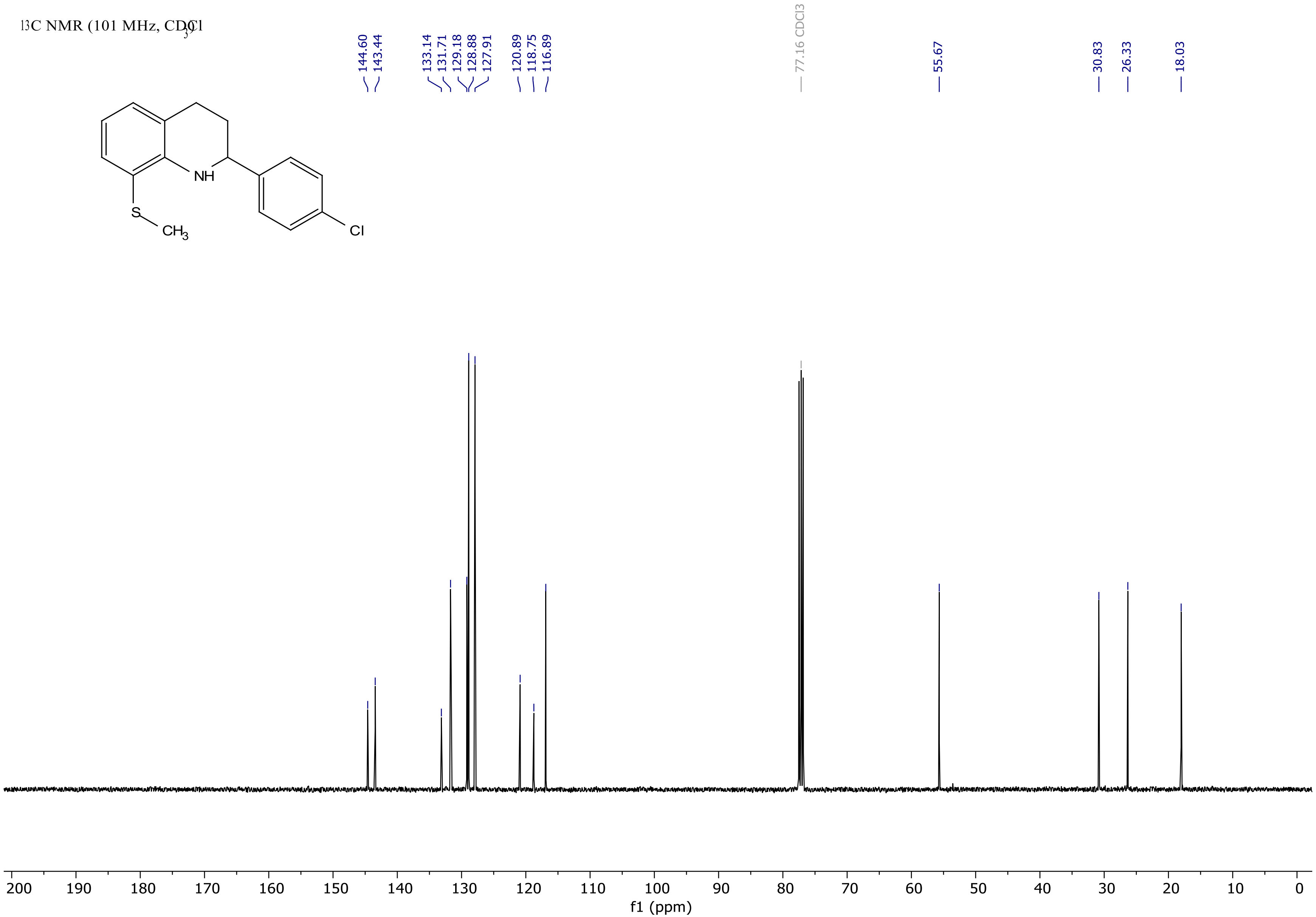

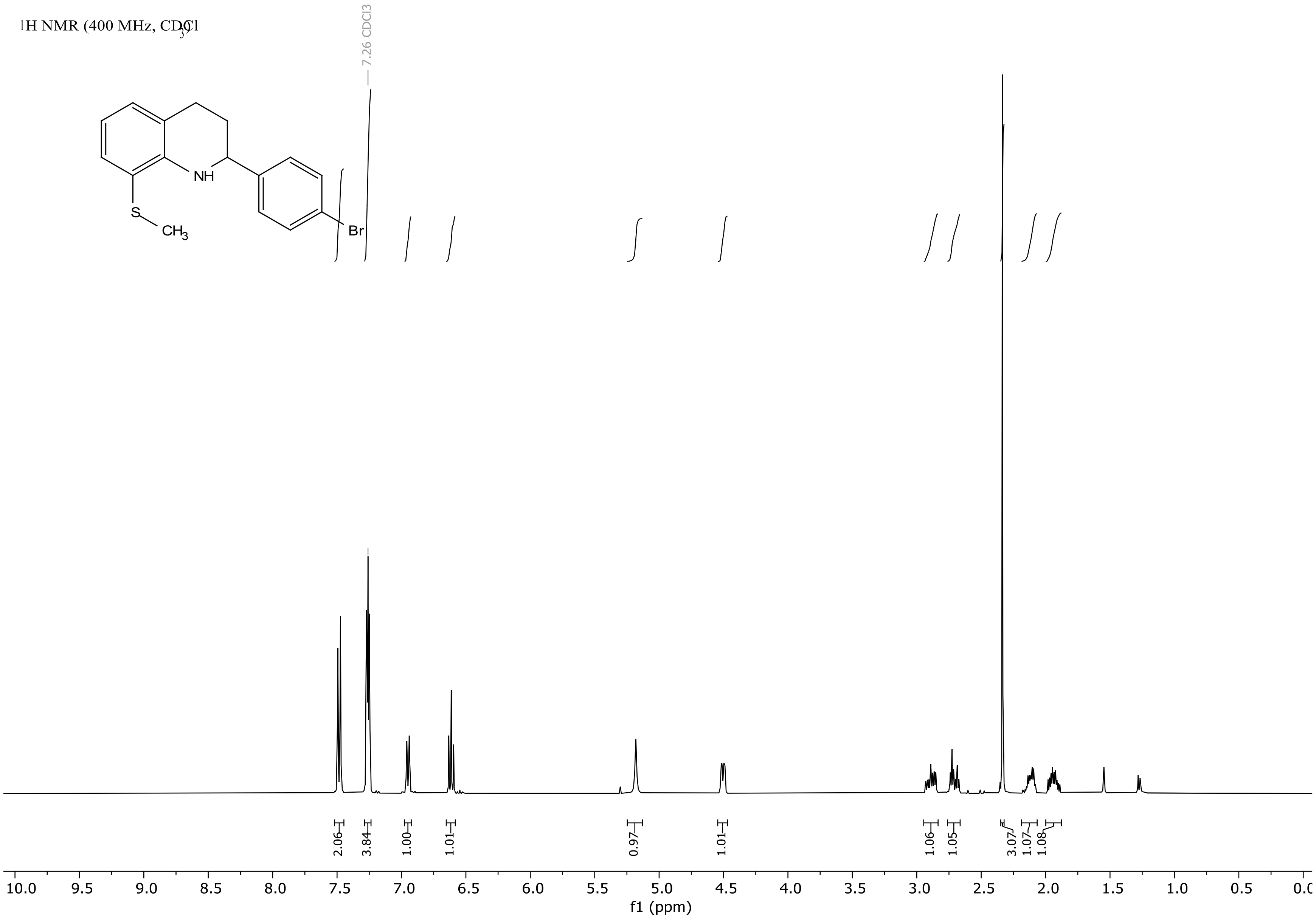
1H NMR (400 MHz, CDCl3)
7.26 CDCl3
NH
S
CH3
Br
2.06
3.84
1.00
1.01
0.97
1.01
1.06
1.05
3.07
1.07
1.08
10.0
9.5
9.0
8.5
8.0
7.5
7.0
6.5
6.0
5.5
5.0
4.5
4.0
3.5
3.0
2.5
2.0
1.5
1.0
0.5
f1 (ppm)

13C NMR (101 MHz, CDCl3)

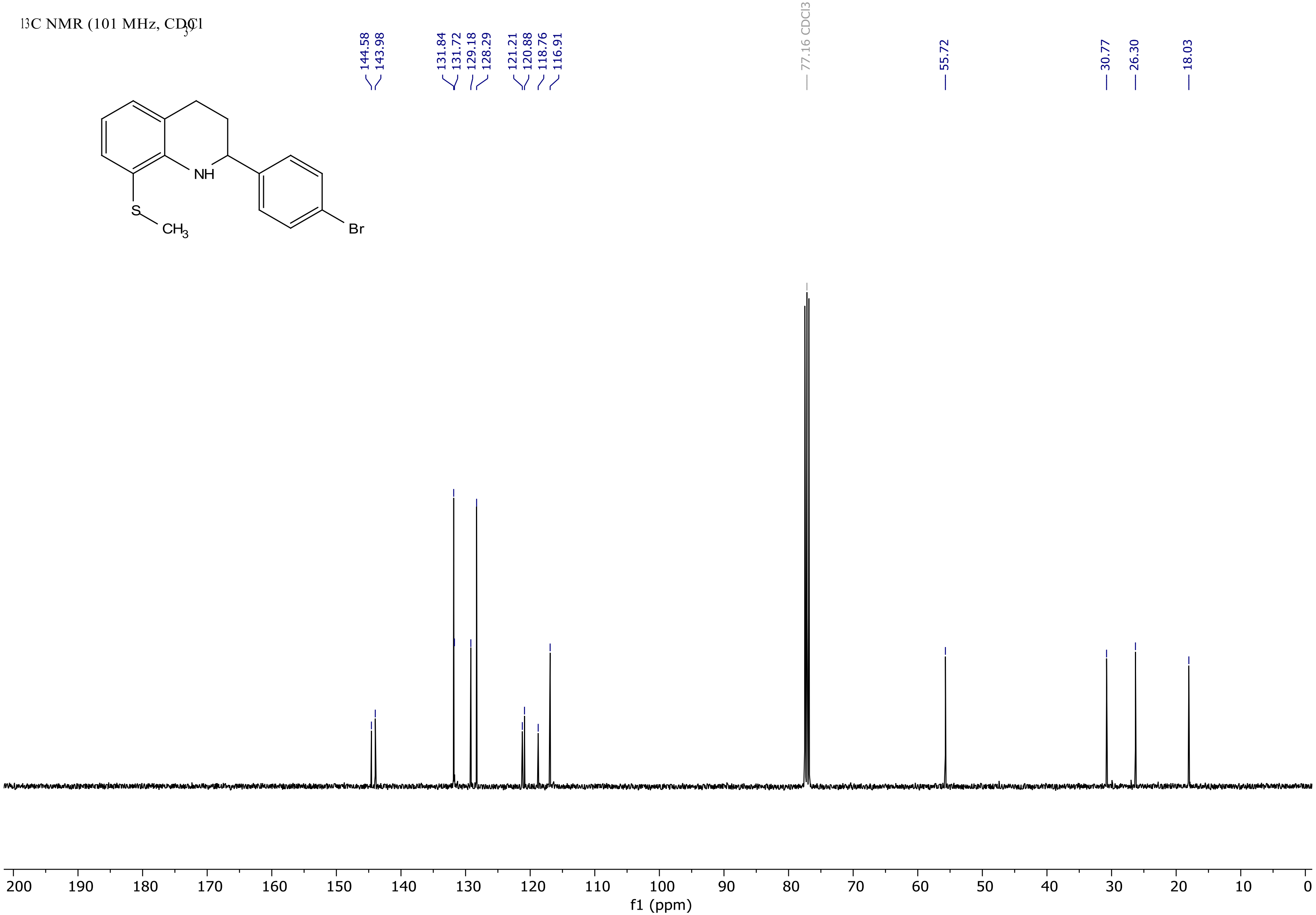

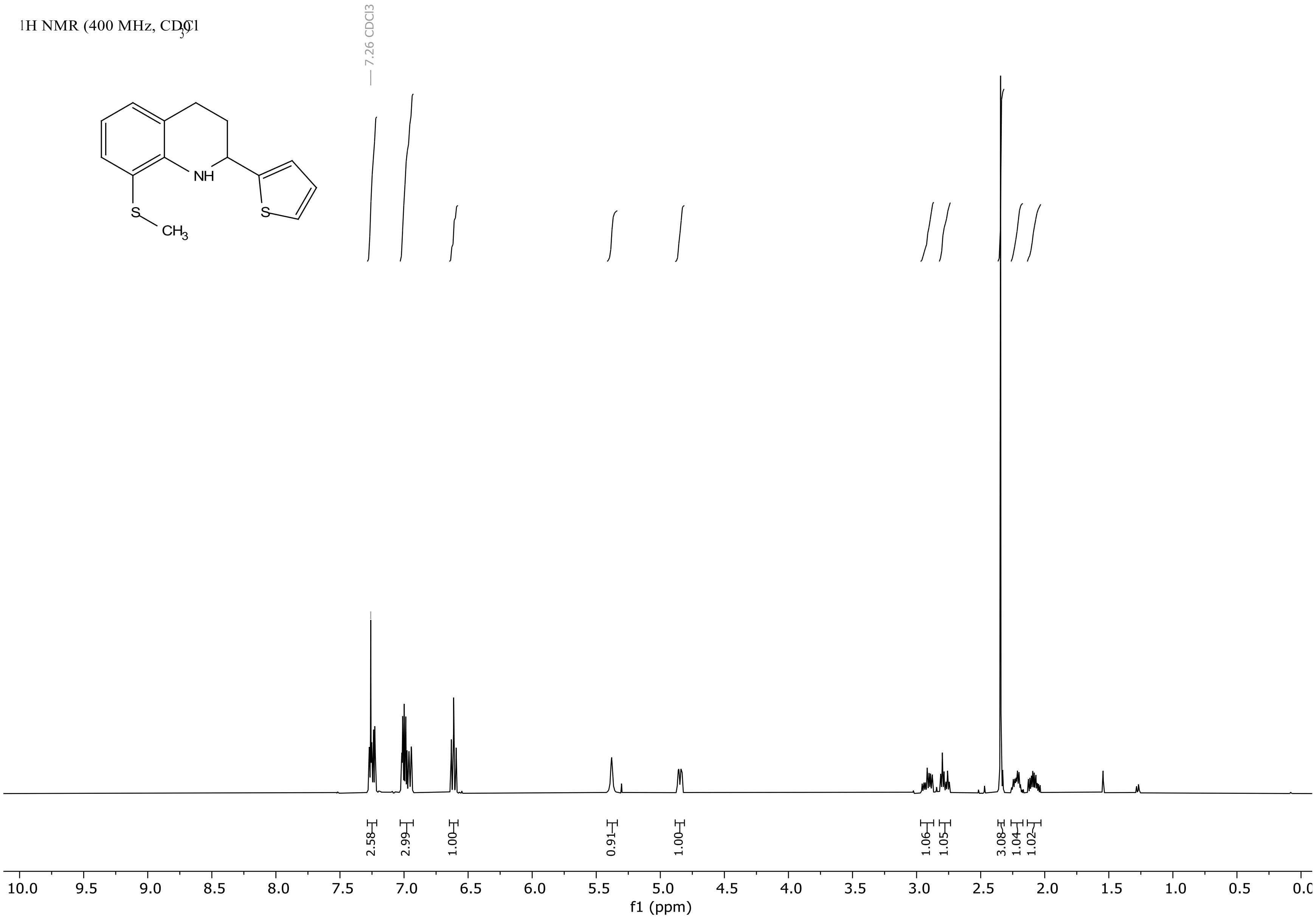
1H NMR (400 MHz, CDCl3)
7.26 CDCl3
NH
S
CH3
S
2.58
2.99
1.00
0.91
1.00
1.06
1.05
3.08
1.04
1.02
10.0 9.5 9.0 8.5 8.0 7.5 7.0 6.5 6.0 5.5 5.0 4.5 4.0 3.5 3.0 2.5 2.0 1.5 1.0 0.5 0.0
f1 (ppm)

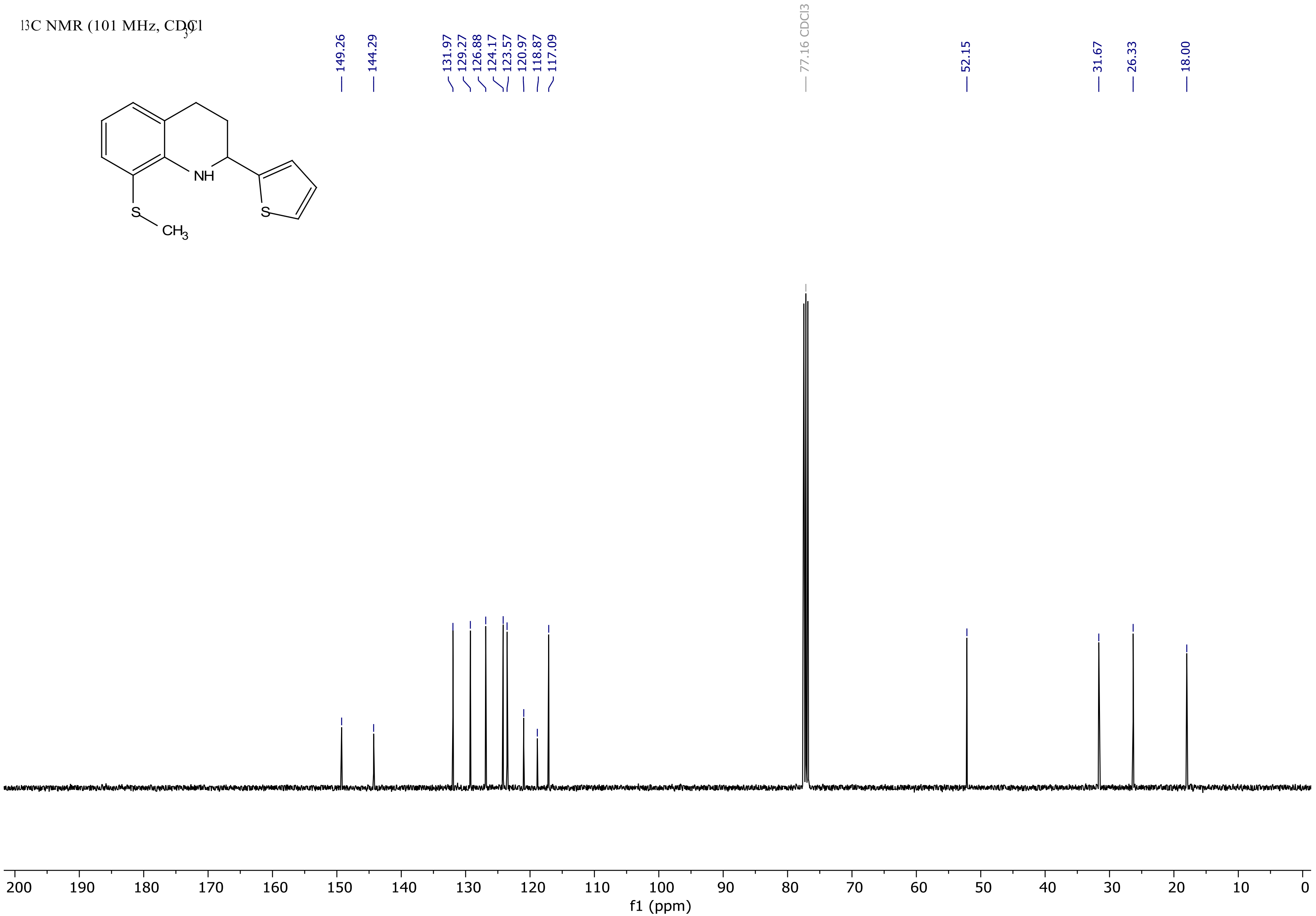
13C NMR (101 MHz, CDCl3)
149.26
144.29
131.97
129.27
126.88
124.17
123.57
120.97
118.87
117.09
77.16 CDCl3
52.15
31.67
26.33
18.00
NH
S
CH3
S
200
190
180
170
160
150
140
130
120
110
100
90
80
70
60
50
40
30
20
10
0
f1 (ppm)

1H NMR (400 MHz, CDCl3)

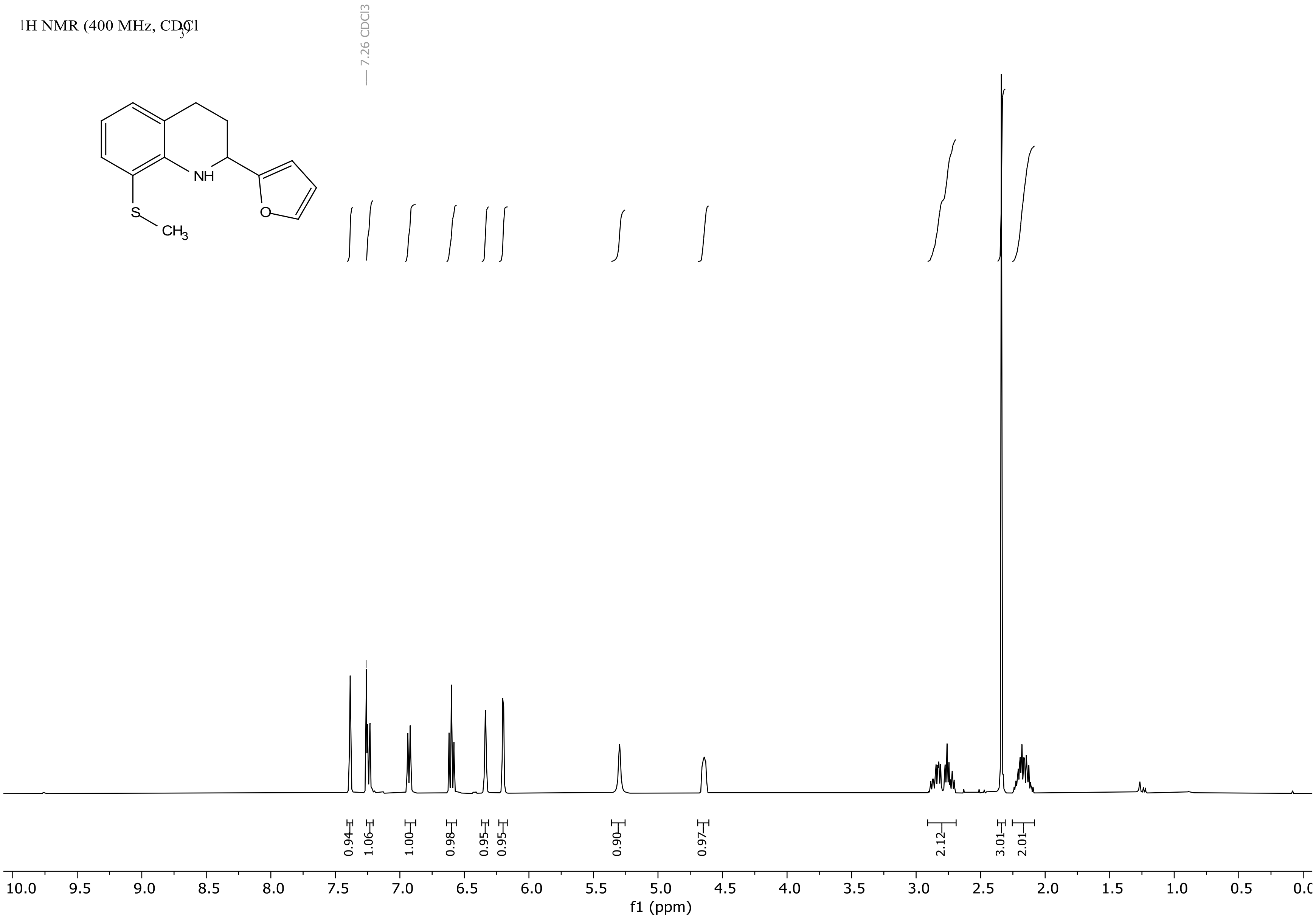

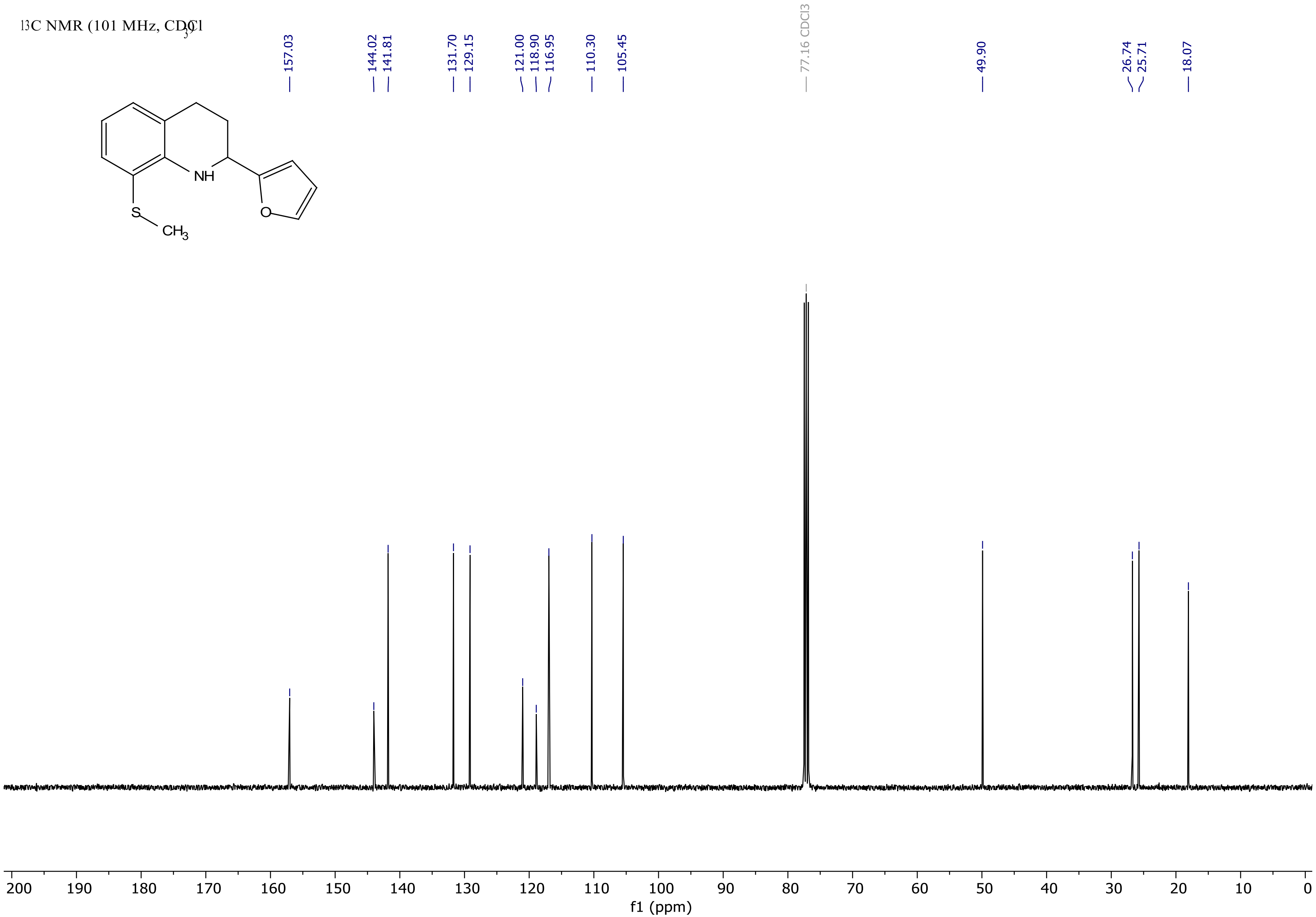
13C NMR (101 MHz, CDCl3)
157.03
144.02
141.81
131.70
129.15
121.00
118.90
116.95
110.30
105.45
77.16 CDCl3
49.90
26.74
25.71
18.07
NH
S
CH3
O
200 190 180 170 160 150 140 130 120 110 100 90 80 70 60 50 40 30 20 10 0
f1 (ppm)

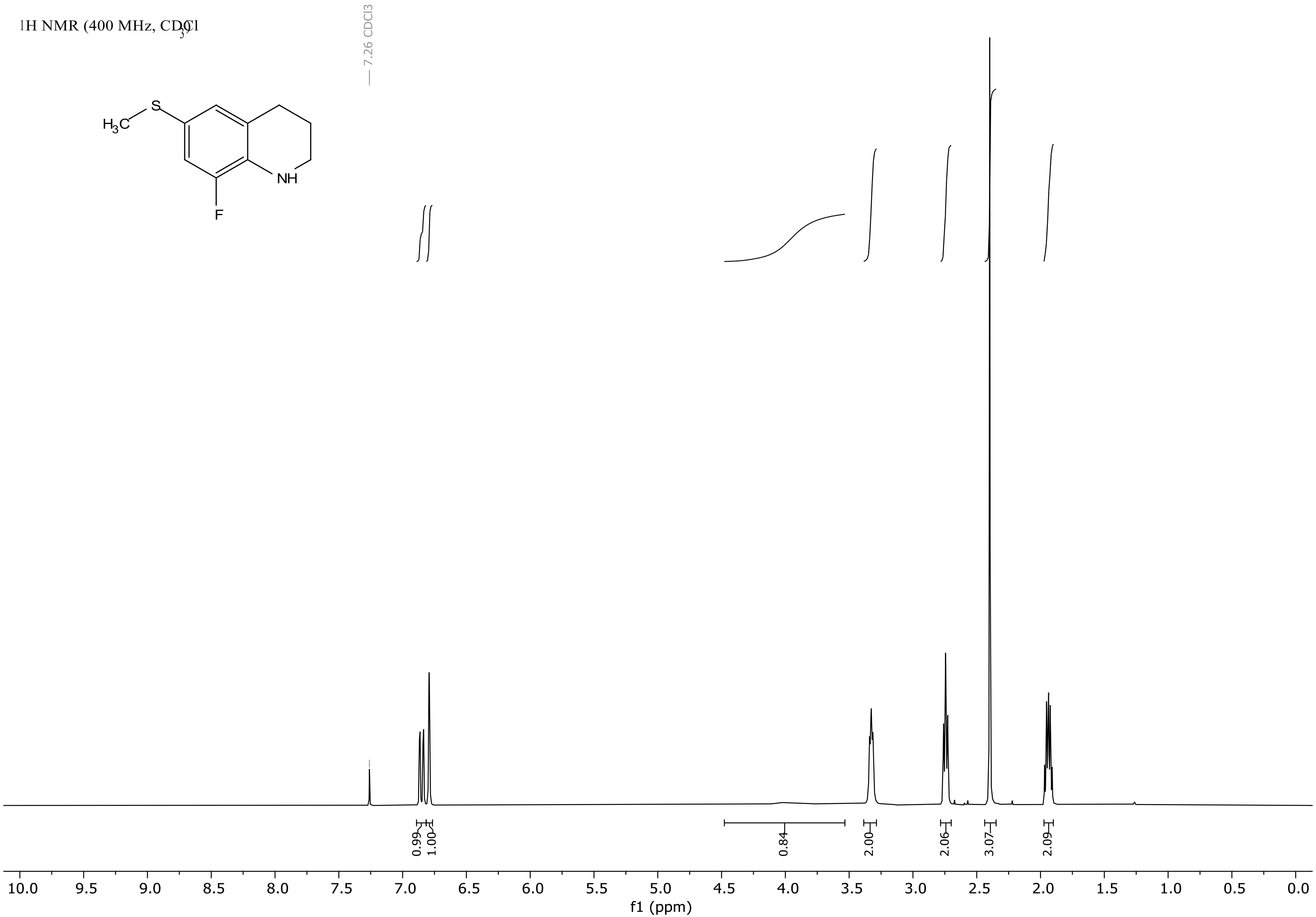
1H NMR (400 MHz, CDCl3)
7.26 CDCl3
H3C
S
NH
F
0.99
1.00
0.84
2.00
2.06
3.07
2.09
10.0
9.5
9.0
8.5
8.0
7.5
7.0
6.5
6.0
5.5
5.0
4.5
4.0
3.5
3.0
2.5
2.0
1.5
1.0
0.5
0.0
f1 (ppm)

13C NMR (101 MHz, CDCl3)

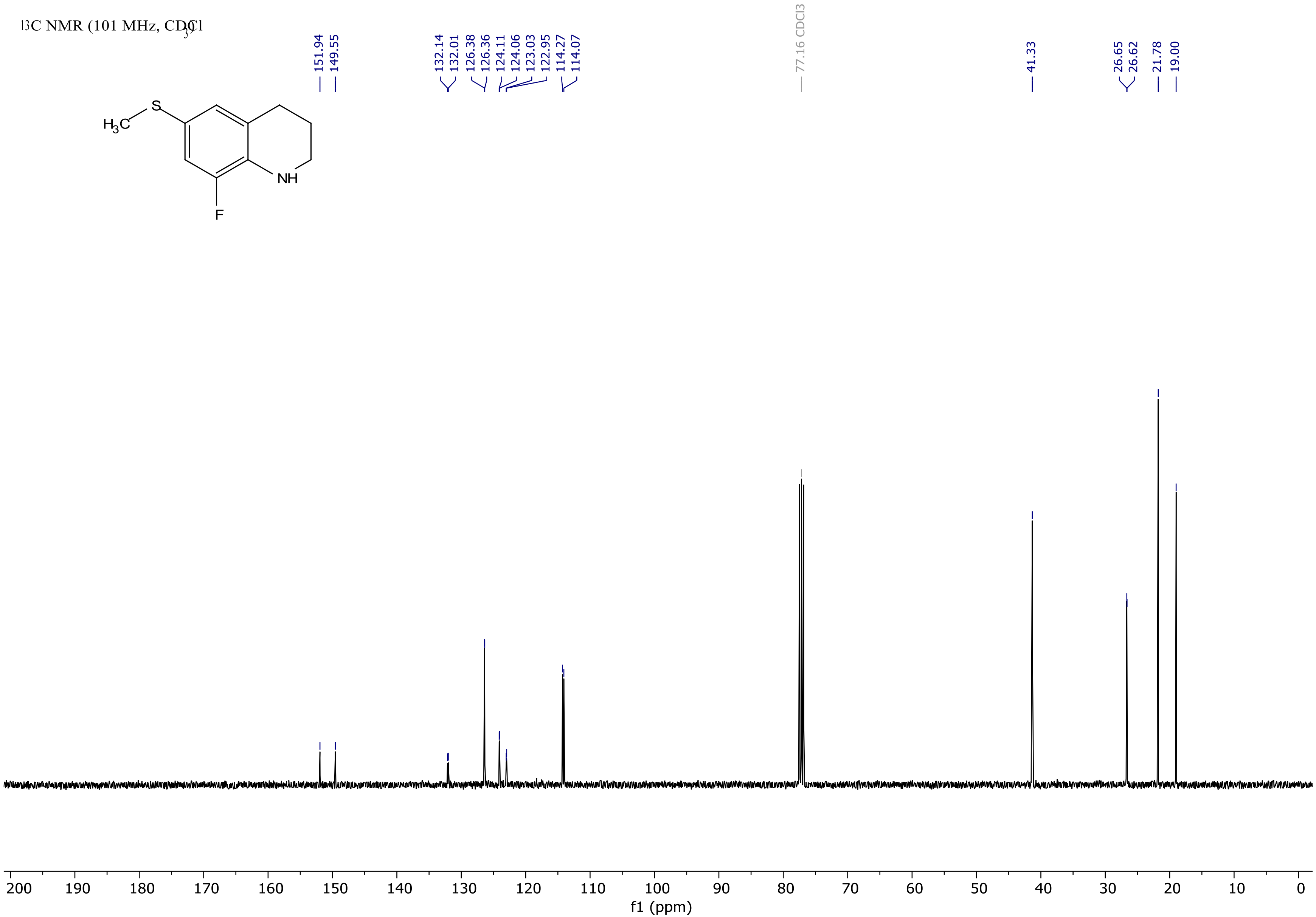

19F NMR (376 MHz, $CDCl_3$)

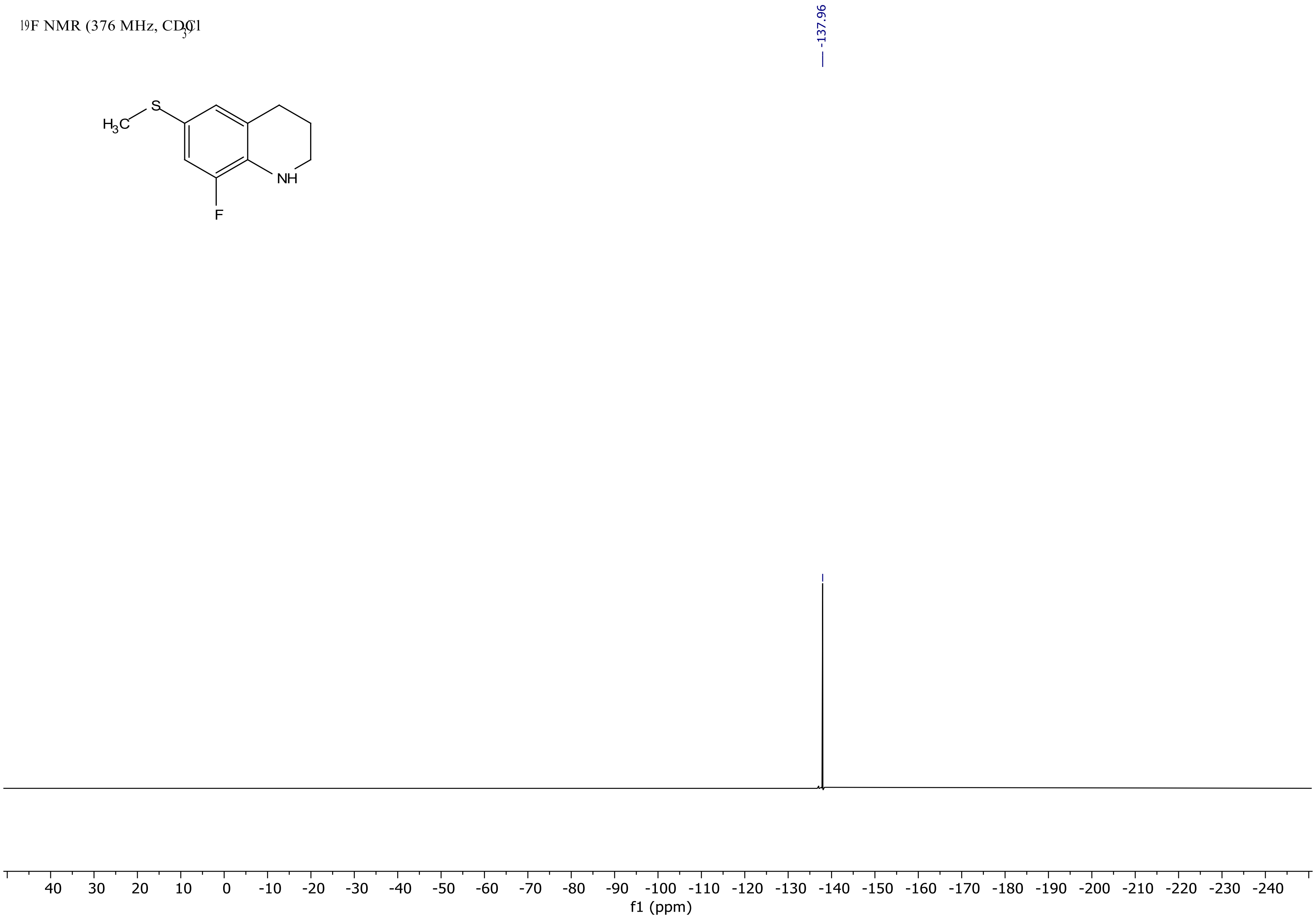

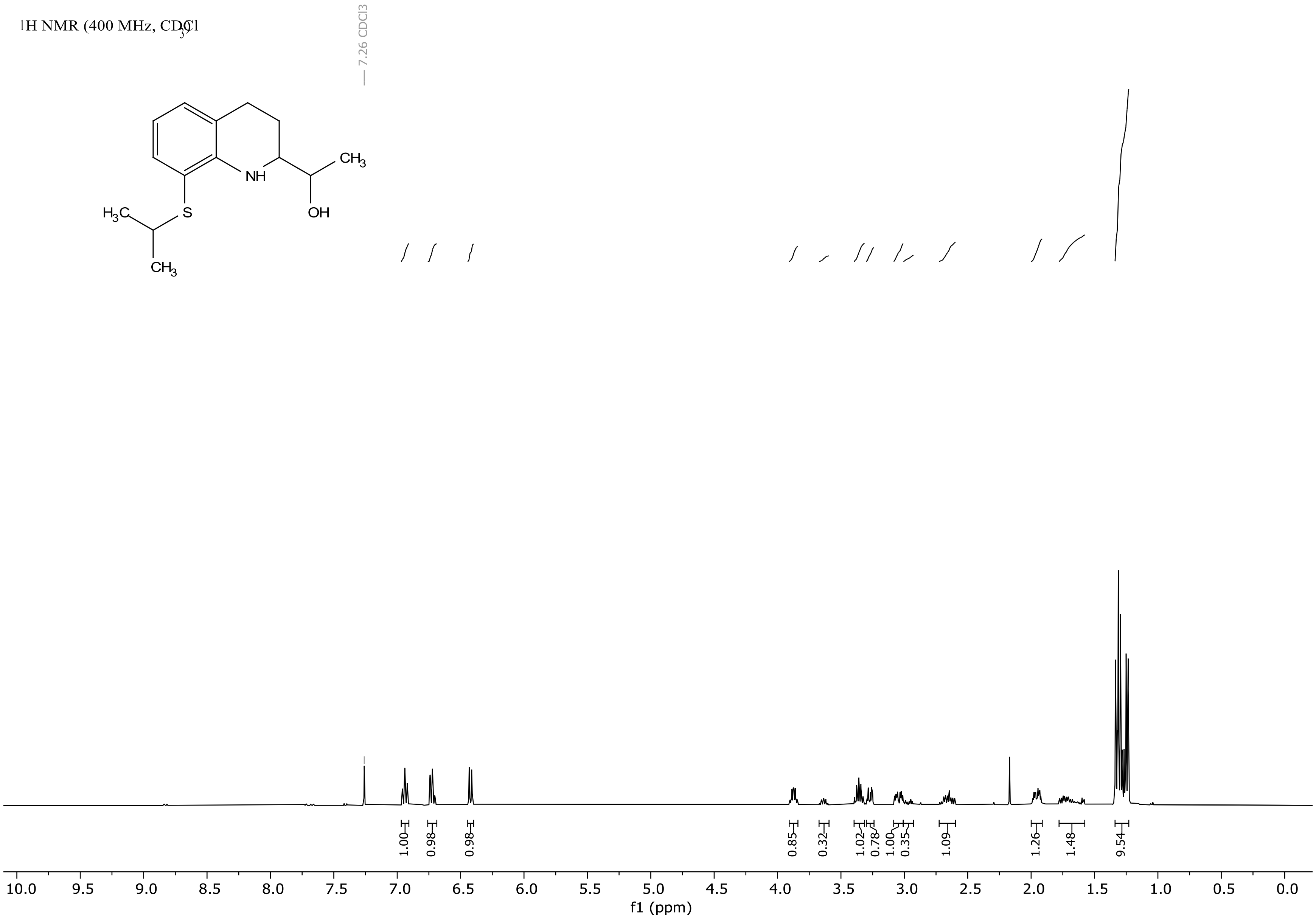
1H NMR (400 MHz, CDCl3)
7.26 CDCl3
CH3
NH
OH
H3C
S
CH3
1.00
0.98
0.98
0.85
0.32
1.02
0.78
1.00
0.35
1.09
1.26
1.48
9.54
10.0 9.5 9.0 8.5 8.0 7.5 7.0 6.5 6.0 5.5 5.0 4.5 4.0 3.5 3.0 2.5 2.0 1.5 1.0 0.5 0.0
f1 (ppm)

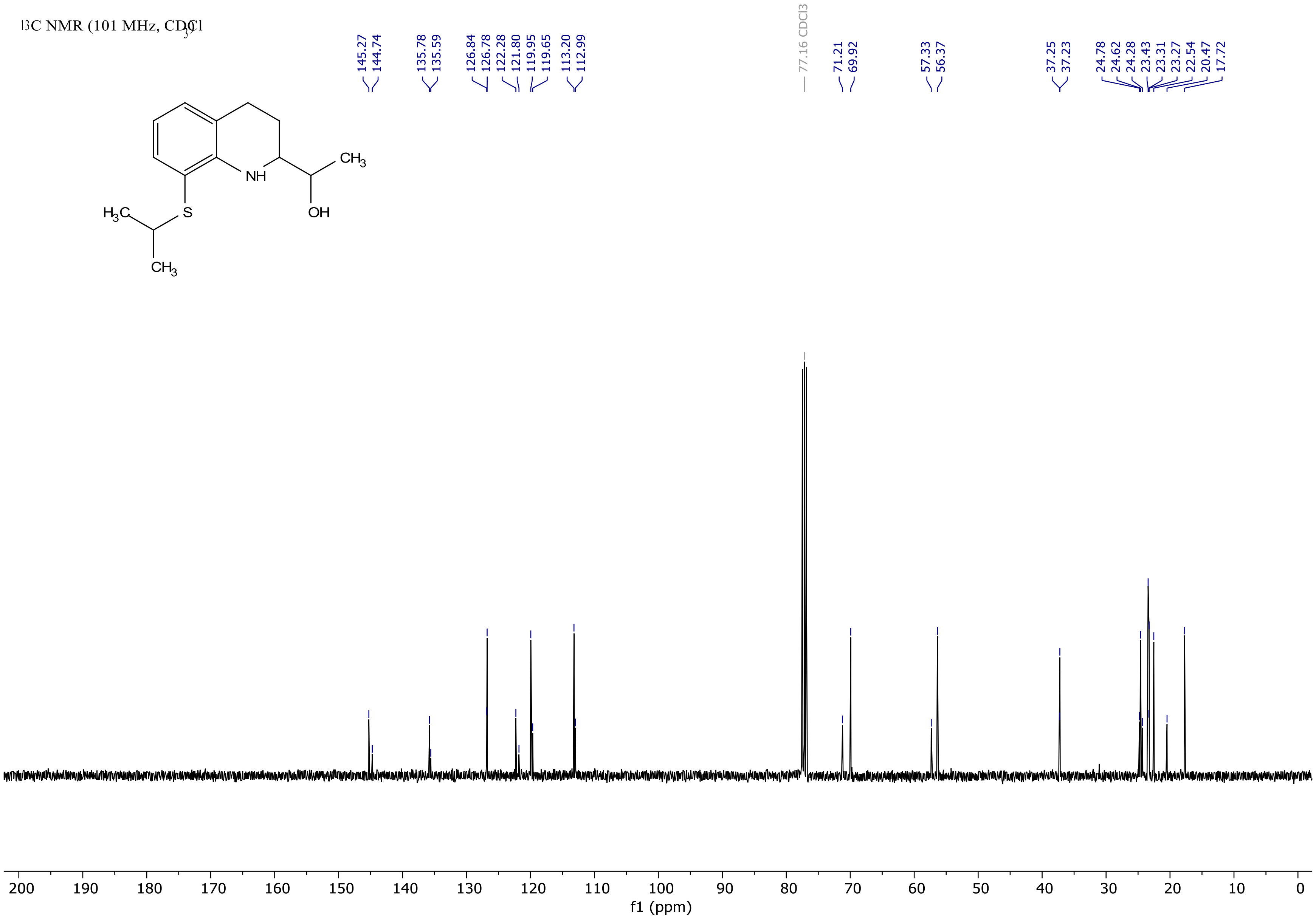
13C NMR (101 MHz, CDCl3)
145.27
144.74
135.78
135.59
126.84
126.78
122.28
121.80
119.95
119.65
113.20
112.99
77.16 CDCl3
71.21
69.92
57.33
56.37
37.25
37.23
24.78
24.62
24.28
23.43
23.31
23.27
22.54
20.47
17.72
CH3
NH
OH
S
H3C
CH3
200
190
180
170
160
150
140
130
120
110
100
90
80
70
60
50
40
30
20
10
0
f1 (ppm)

1H NMR (400 MHz, $CDCl_3$)

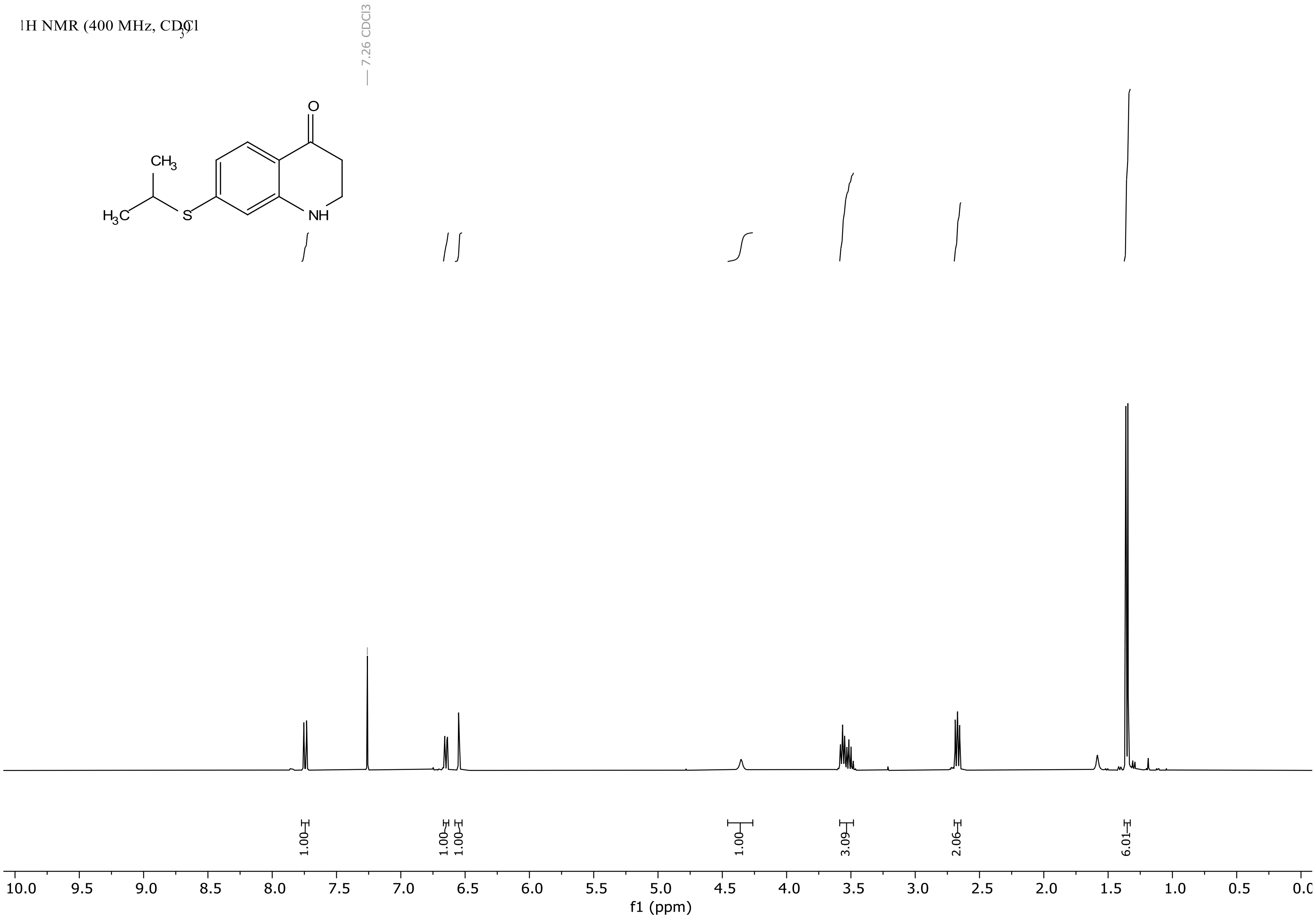

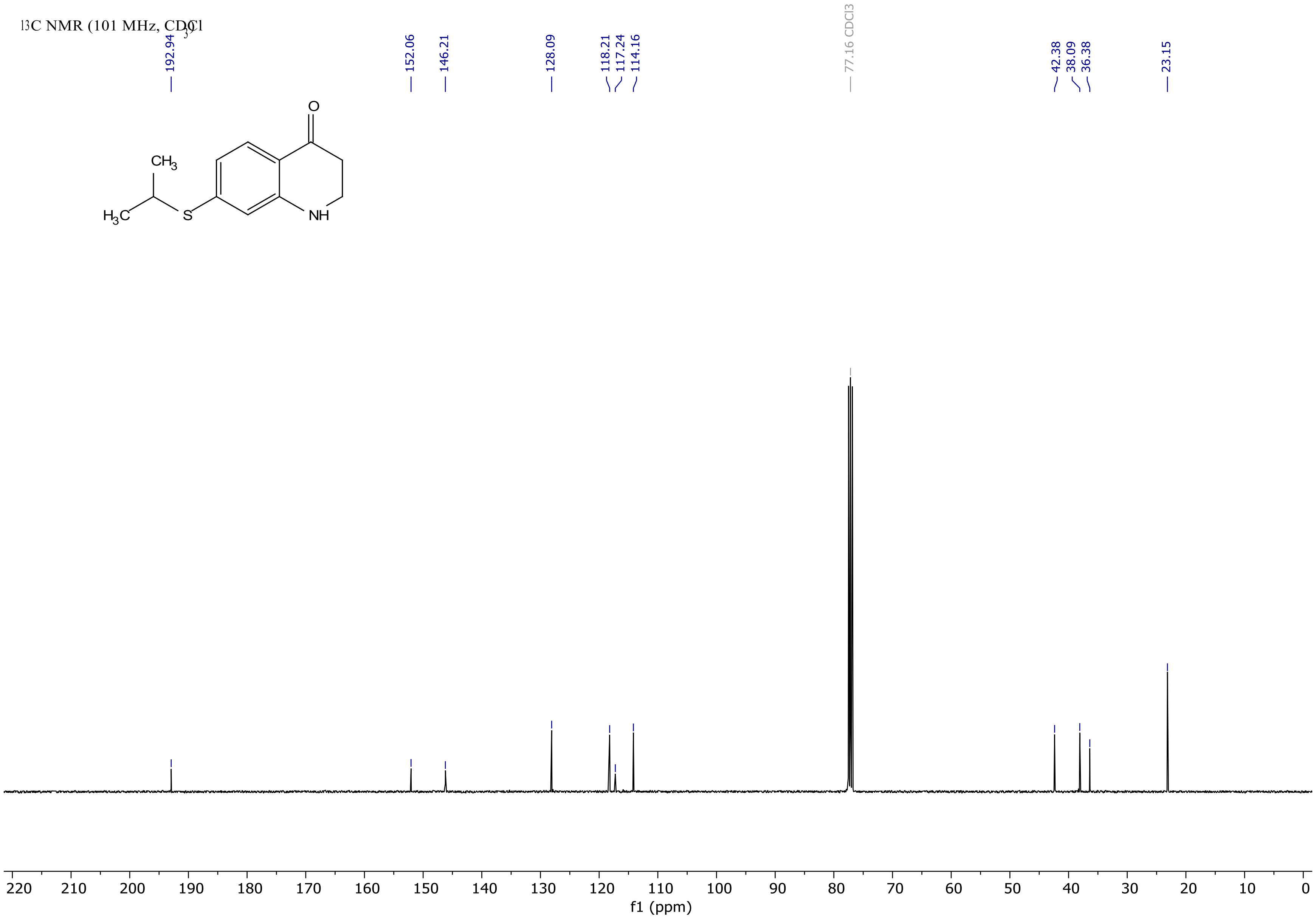
13C NMR (101 MHz, CDCl3)
192.94
152.06
146.21
128.09
118.21
117.24
114.16
77.16 CDCl3
42.38
38.09
36.38
23.15
O
CH3
H3C
S
NH
220 210 200 190 180 170 160 150 140 130 120 110 100 90 80 70 60 50 40 30 20 10 0
f1 (ppm)

1H NMR (400 MHz, CDCl3)

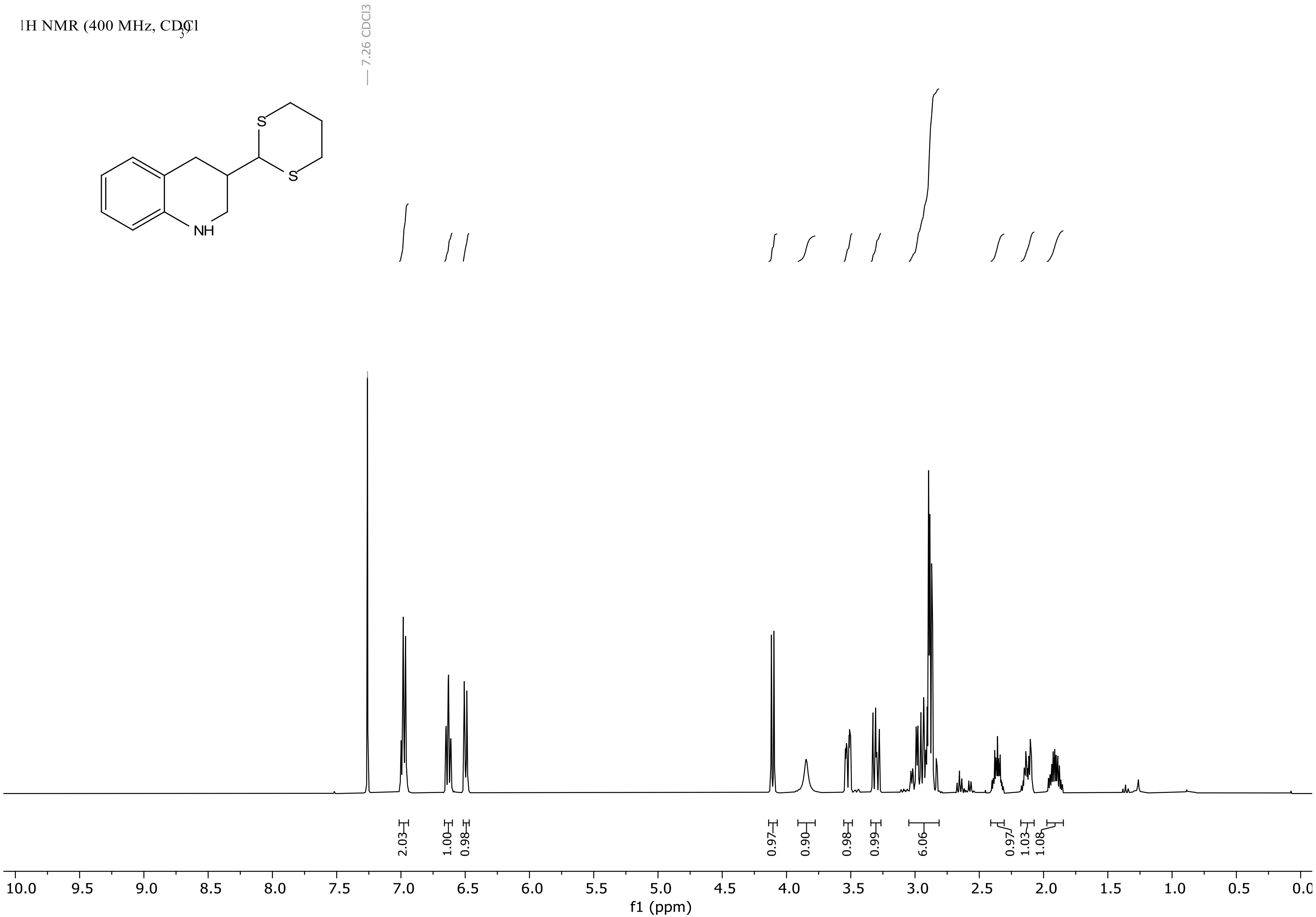

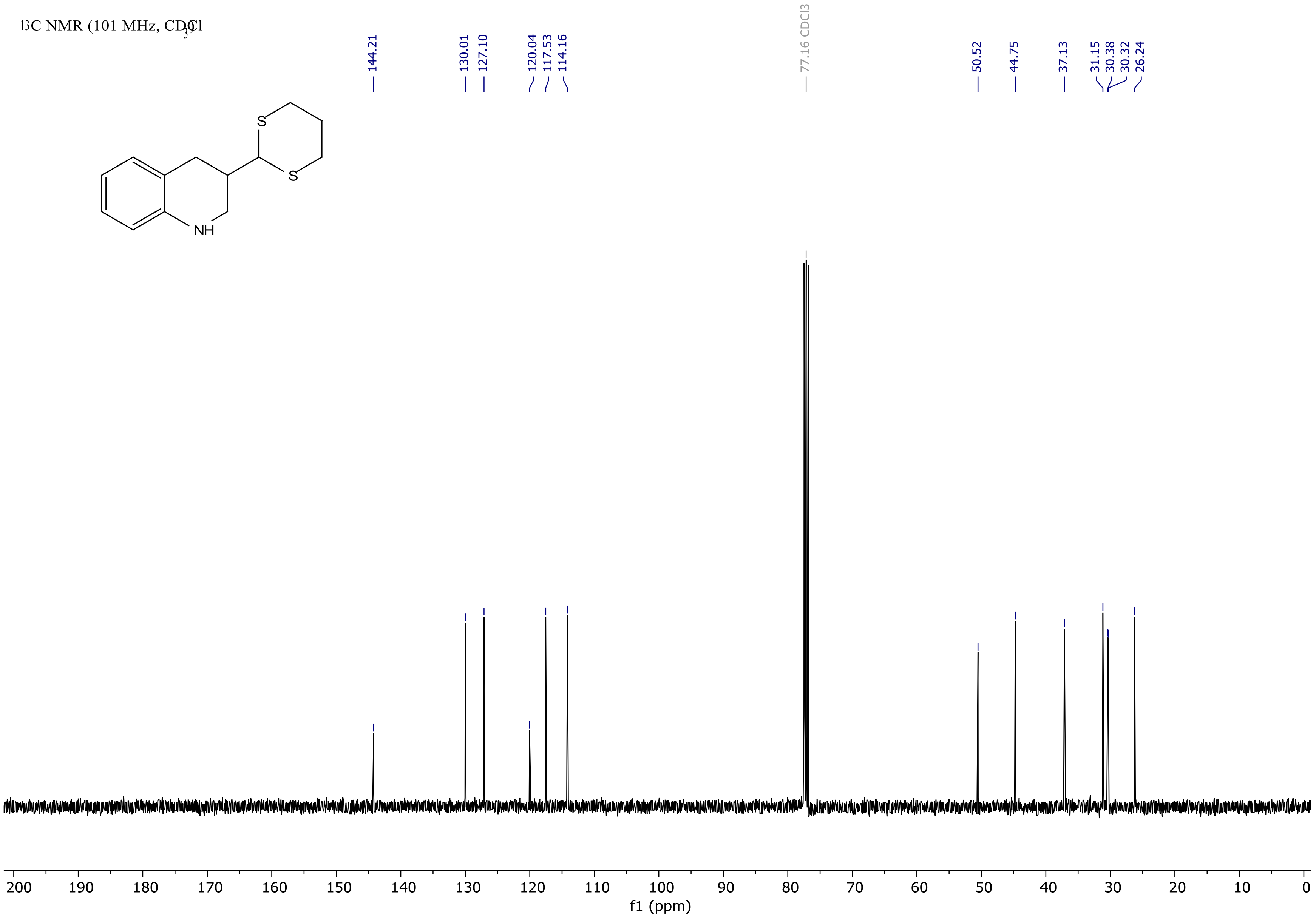
13C NMR (101 MHz, CDCl3)
144.21
130.01
127.10
120.04
117.53
114.16
77.16 CDCl3
50.52
44.75
37.13
31.15
30.38
30.32
26.24
S
S
NH
200 190 180 170 160 150 140 130 120 110 100 90 80 70 60 50 40 30 20 10 0
f1 (ppm)

1H NMR (400 MHz, CDCl3)

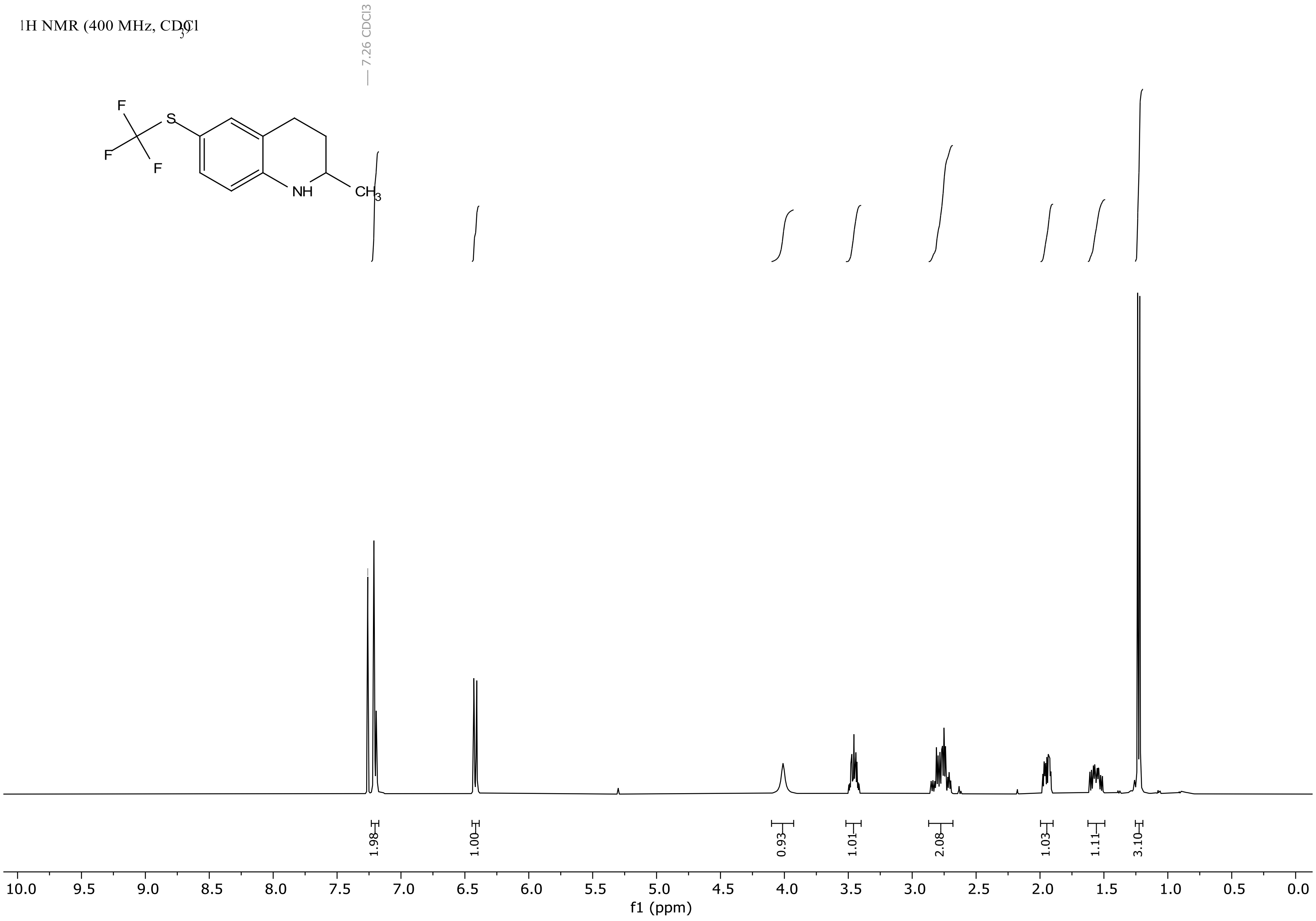

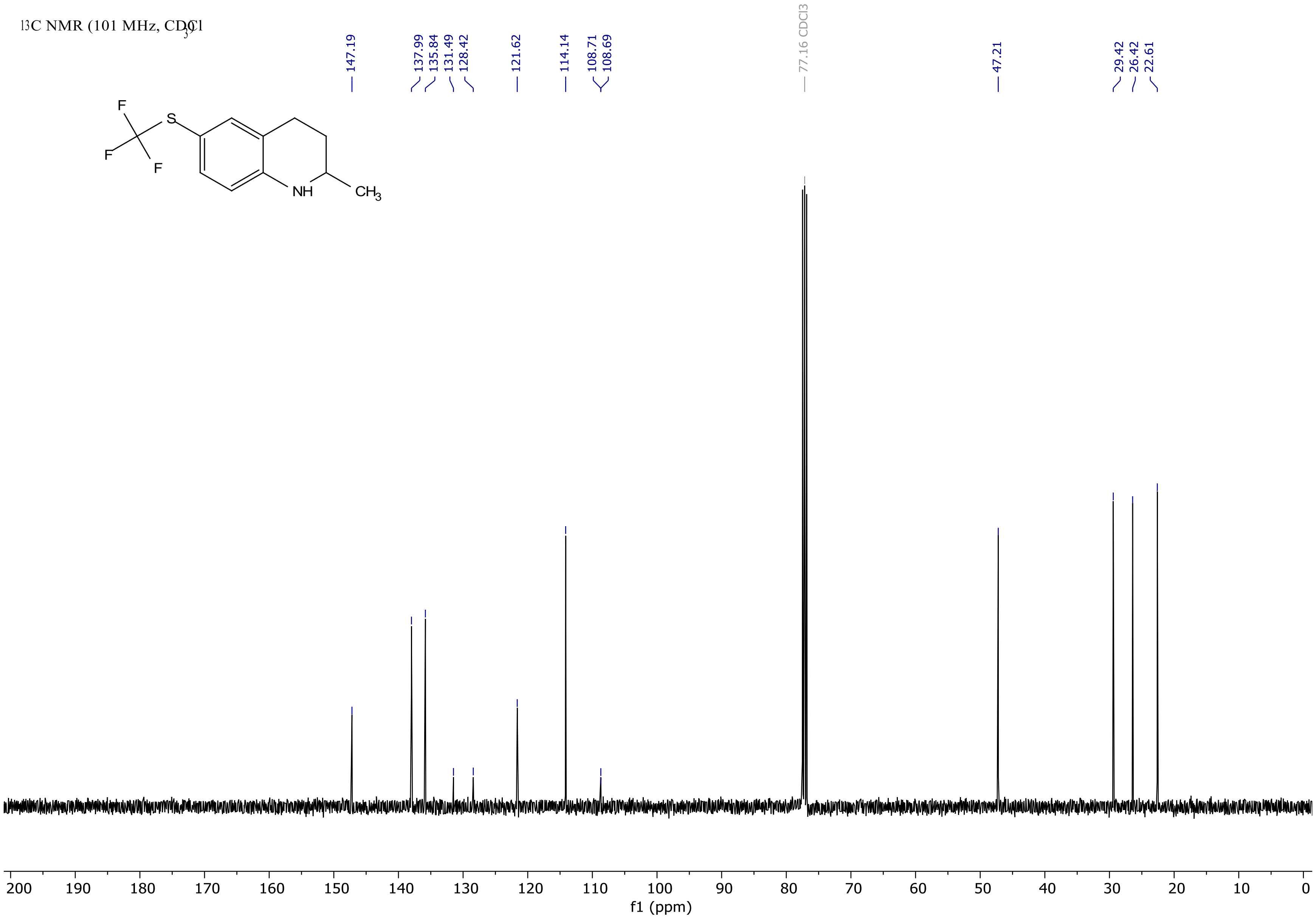
13C NMR (101 MHz, CDCl3)
147.19
137.99
135.84
131.49
128.42
121.62
114.14
108.71
108.69
77.16 CDCl3
47.21
29.42
26.42
22.61
F
F
F
S
NH
CH3
200 190 180 170 160 150 140 130 120 110 100 90 80 70 60 50 40 30 20 10 0
f1 (ppm)

19F NMR (376 MHz, $CDCl_3$)

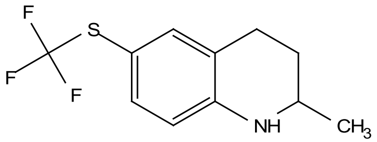

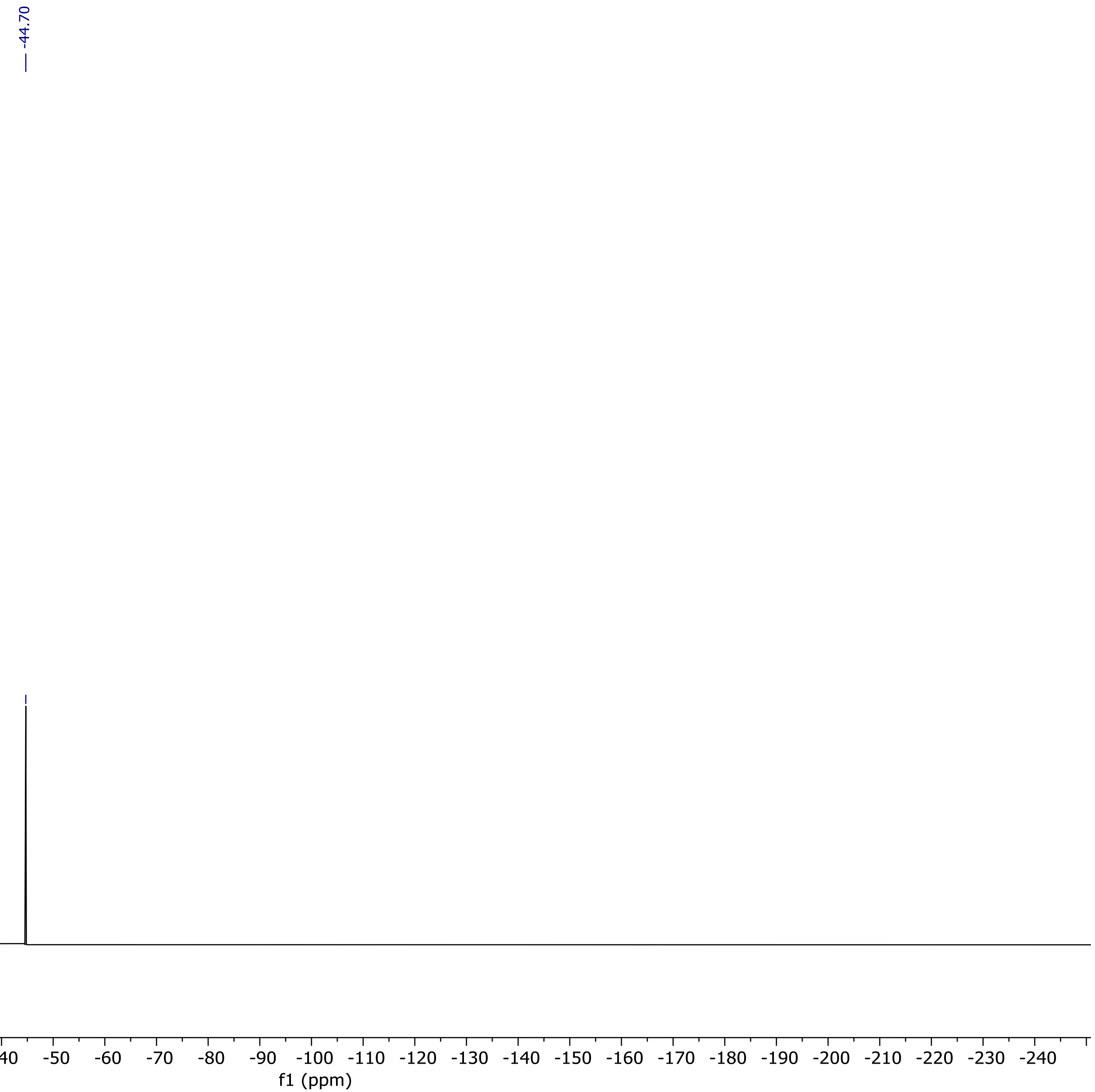

1H NMR (400 MHz, CDCl3)

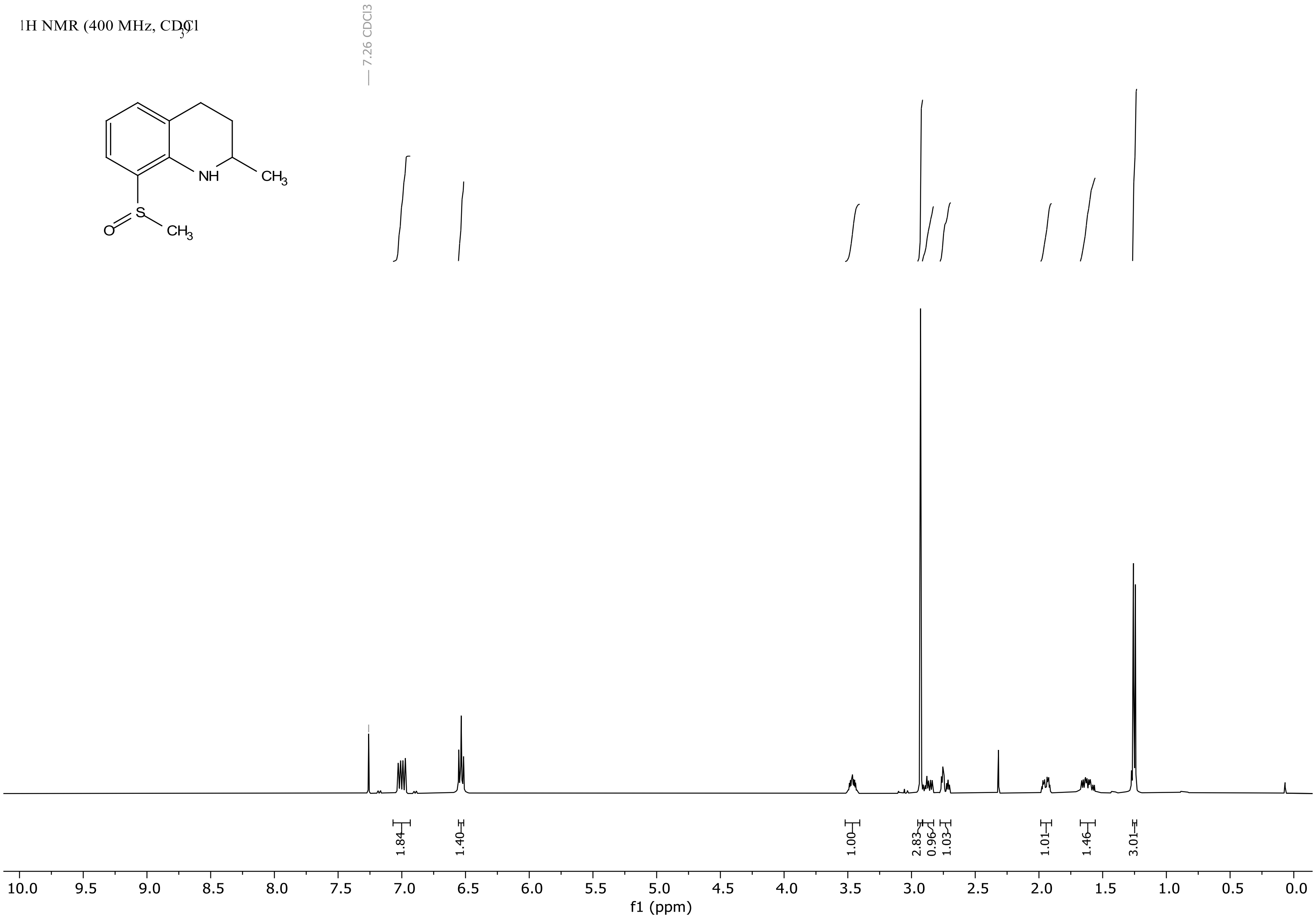

13C NMR (101 MHz, $CDCl_3$)

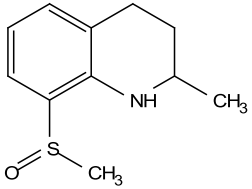

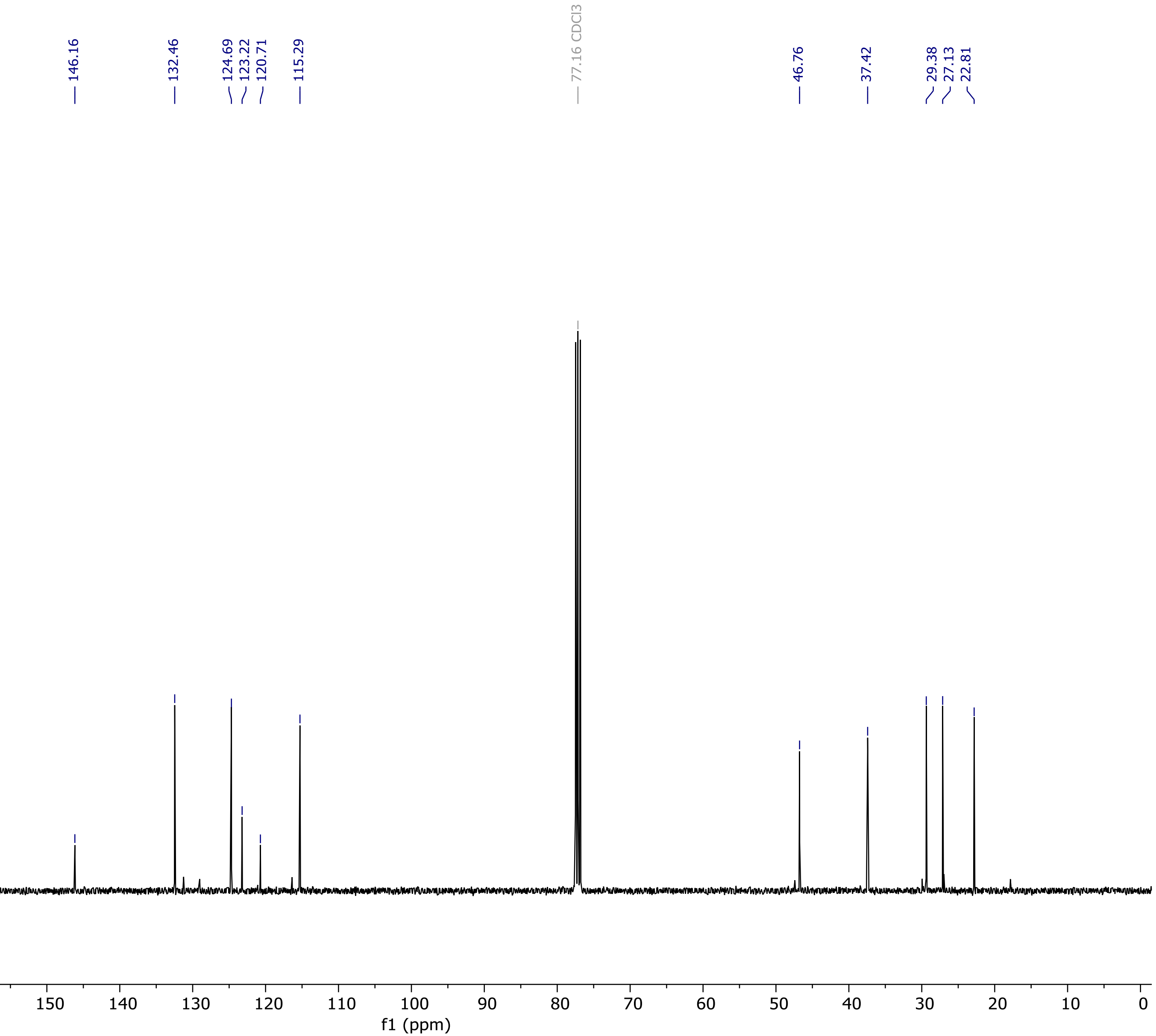

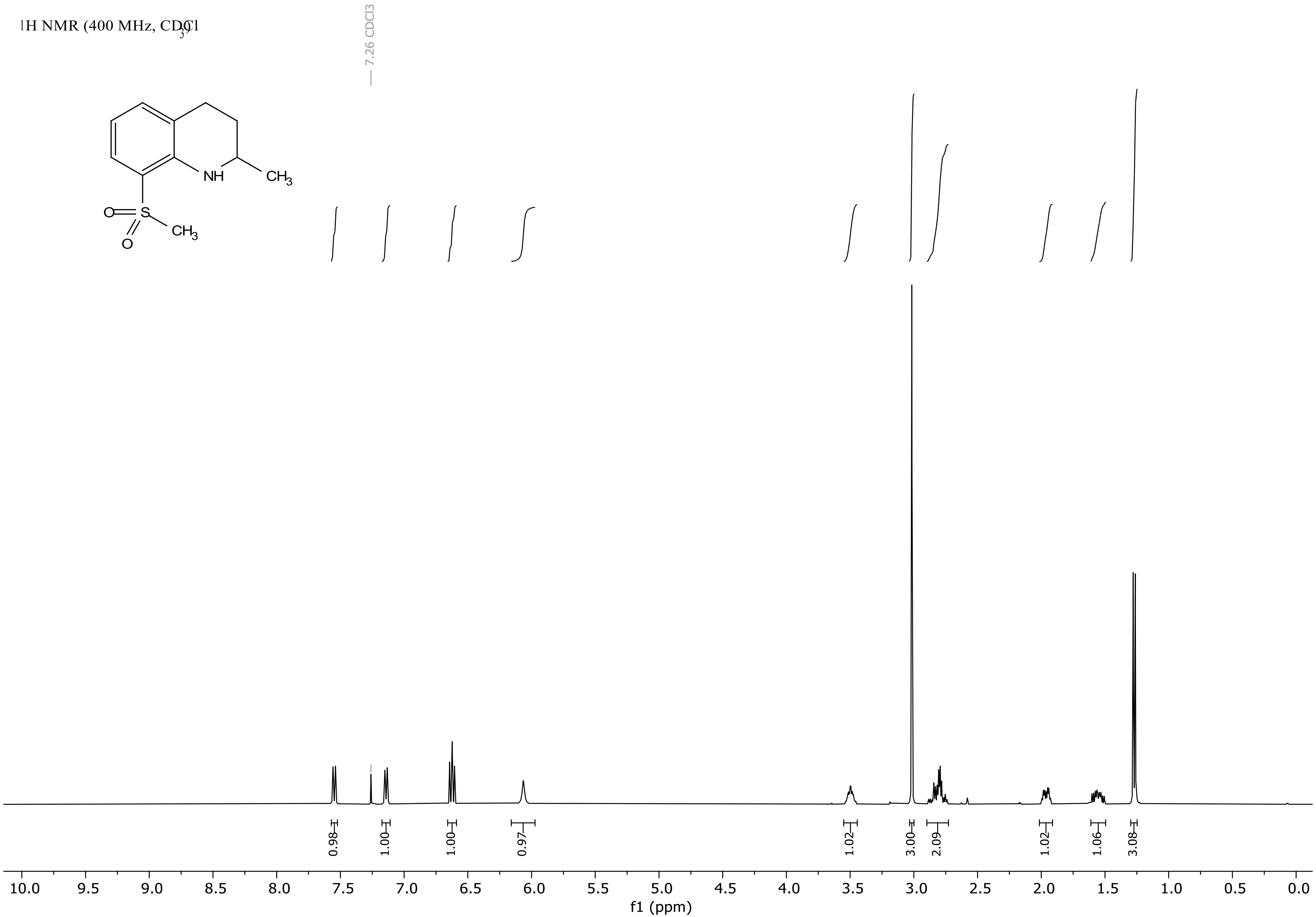
1H NMR (400 MHz, CDCl3)
7.26 CDCl3
NH
CH3
O
S
O
CH3
0.98
1.00
1.00
0.97
1.02
3.00
2.09
1.02
1.06
3.08
10.0 9.5 9.0 8.5 8.0 7.5 7.0 6.5 6.0 5.5 5.0 4.5 4.0 3.5 3.0 2.5 2.0 1.5 1.0 0.5 0.0
f1 (ppm)

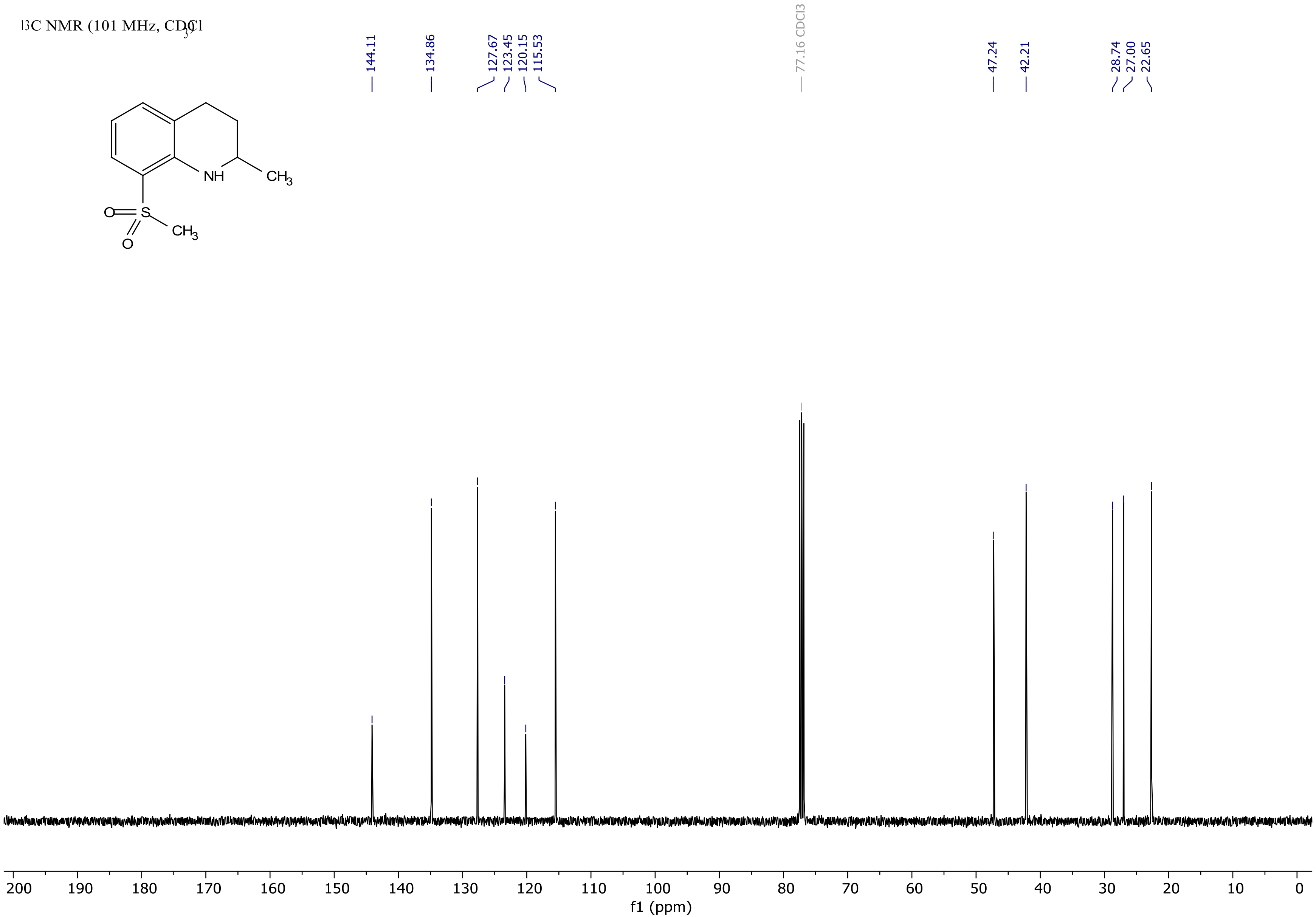

13C NMR (101 MHz, CDCl3)
144.11
134.86
127.67
123.45
120.15
115.53
77.16 CDCl3
47.24
42.21
28.74
27.00
22.65
NH
CH3
O
S
CH3
O
200
190
180
170
160
150
140
130
120
110
100
90
80
70
60
50
40
30
20
10
0
f1 (ppm)

1H NMR (400 MHz, $CDCl_3$)

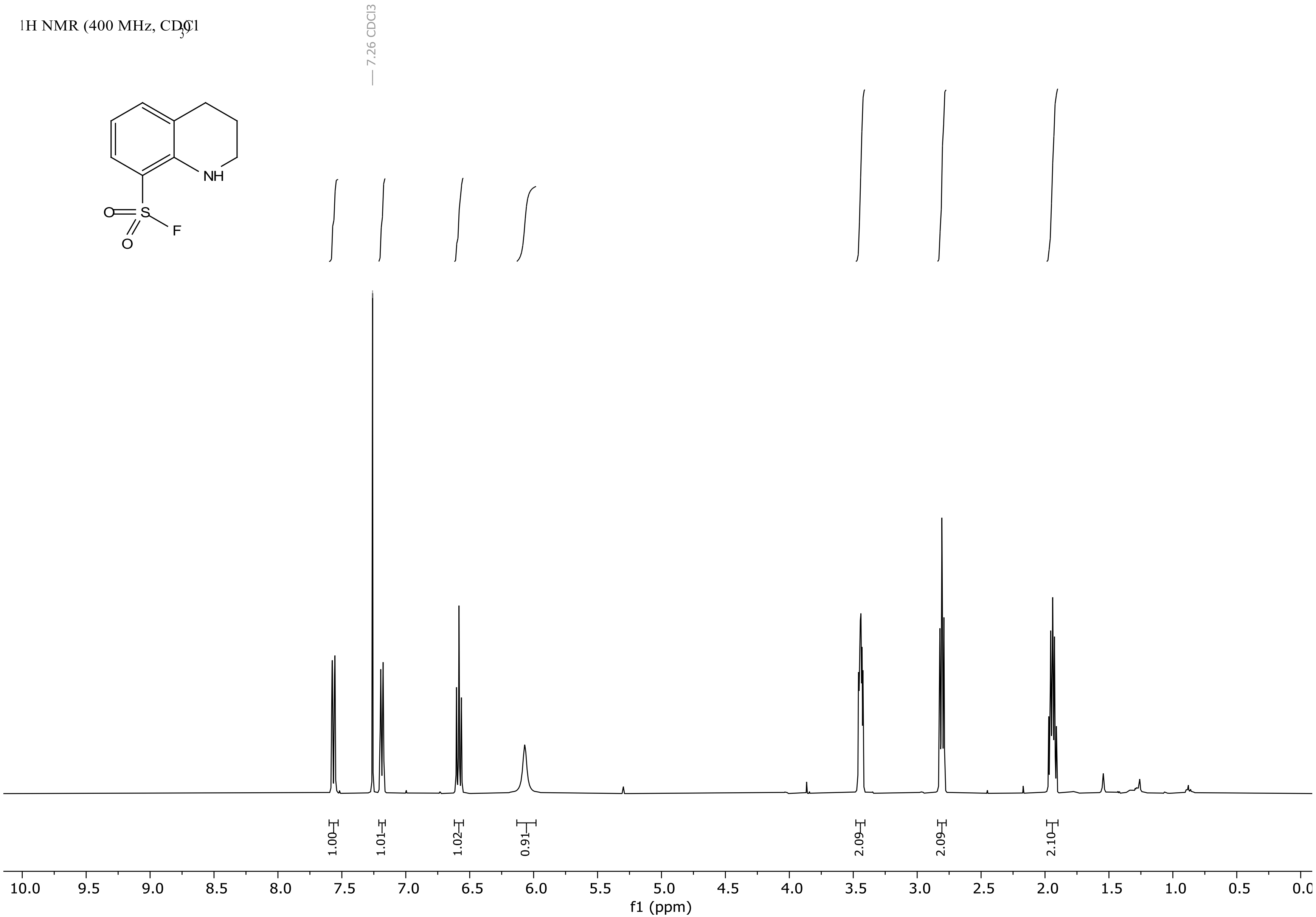

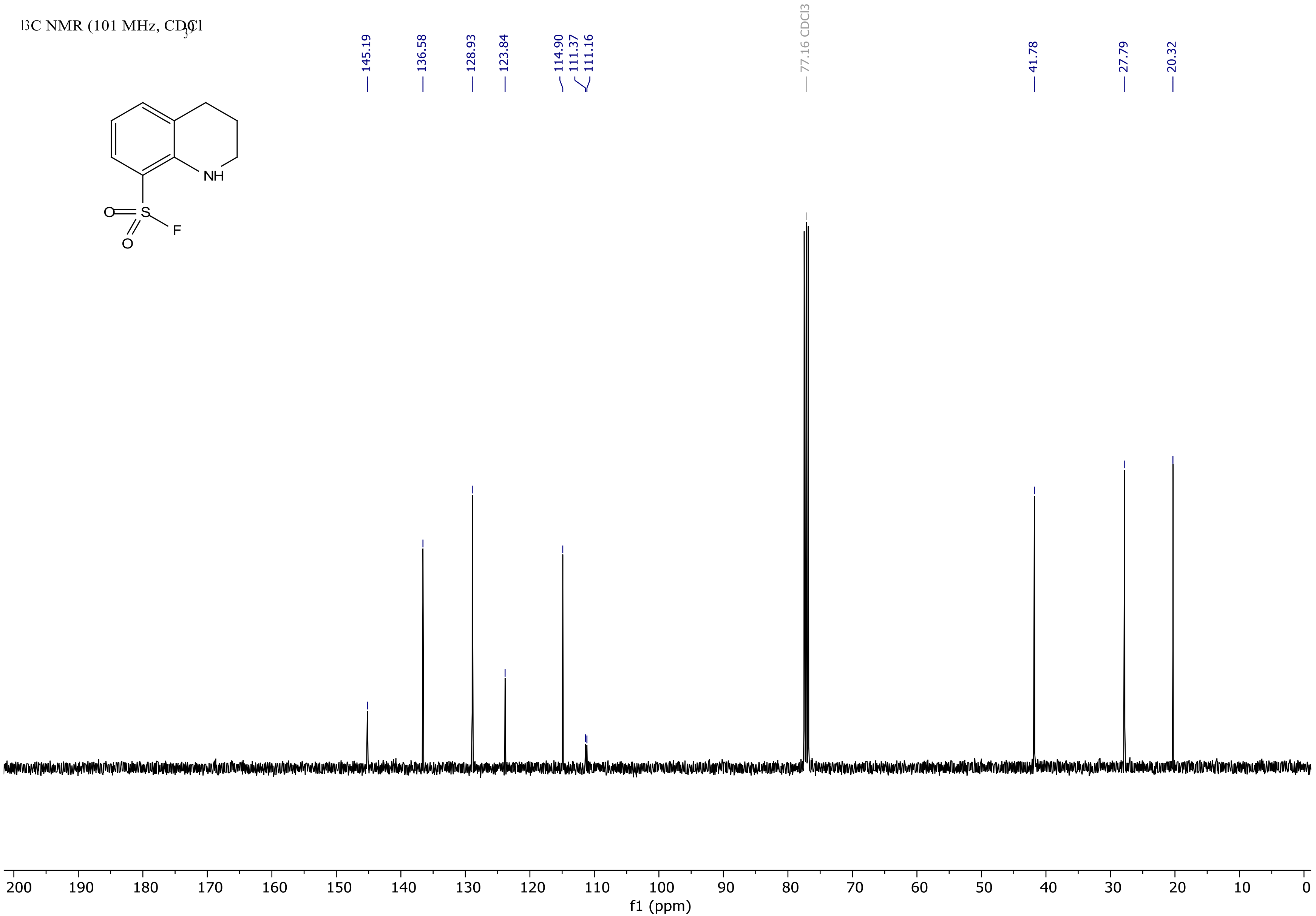
13C NMR (101 MHz, CDCl3)
145.19
136.58
128.93
123.84
114.90
111.37
111.16
77.16 CDCl3
41.78
27.79
20.32
NH
O
S
F
O
200 190 180 170 160 150 140 130 120 110 100 90 80 70 60 50 40 30 20 10 0
f1 (ppm)

19F NMR (376 MHz, $CDCl_3$)

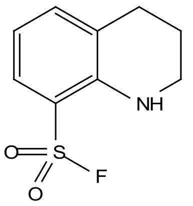

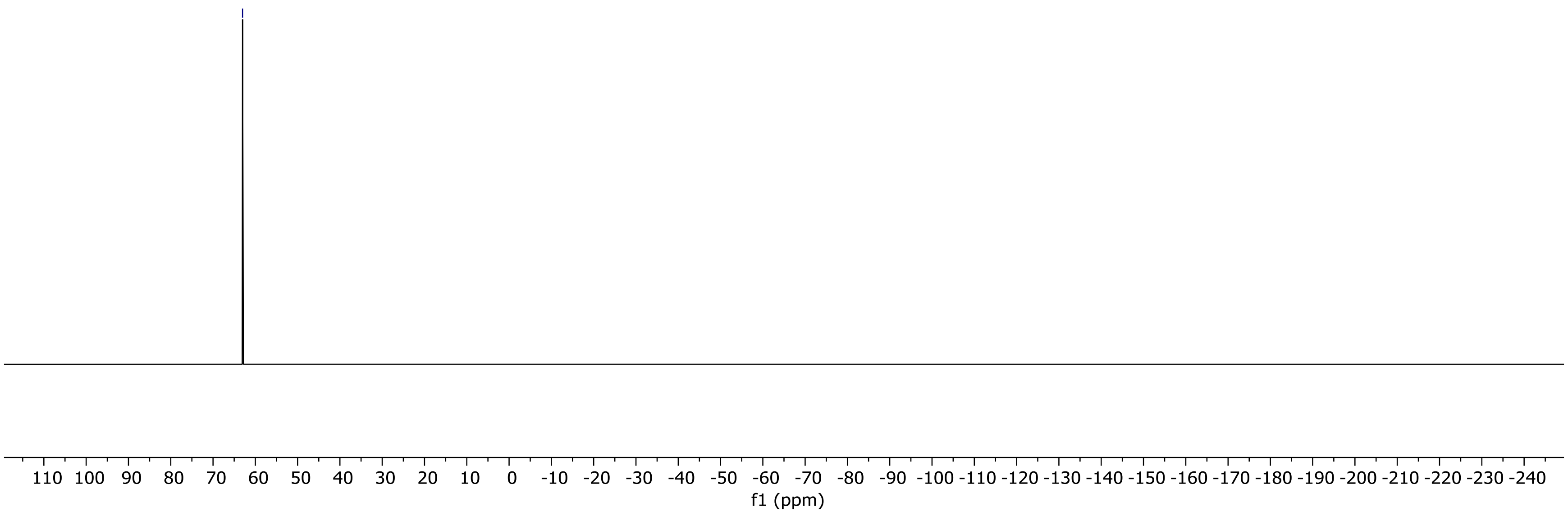

1H NMR (400 MHz, $CDCl_3$)

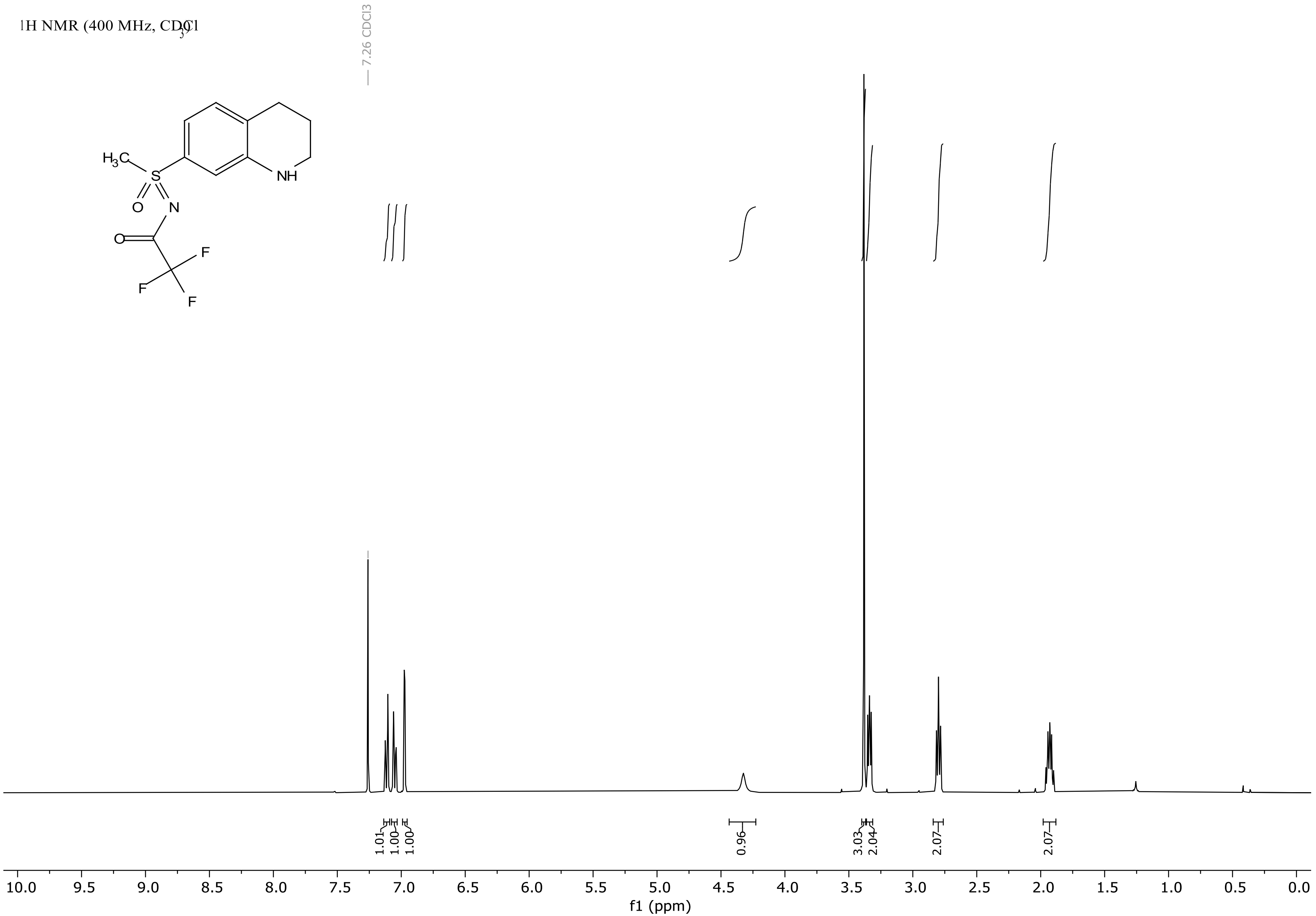

13C NMR (101 MHz, CDCl3)

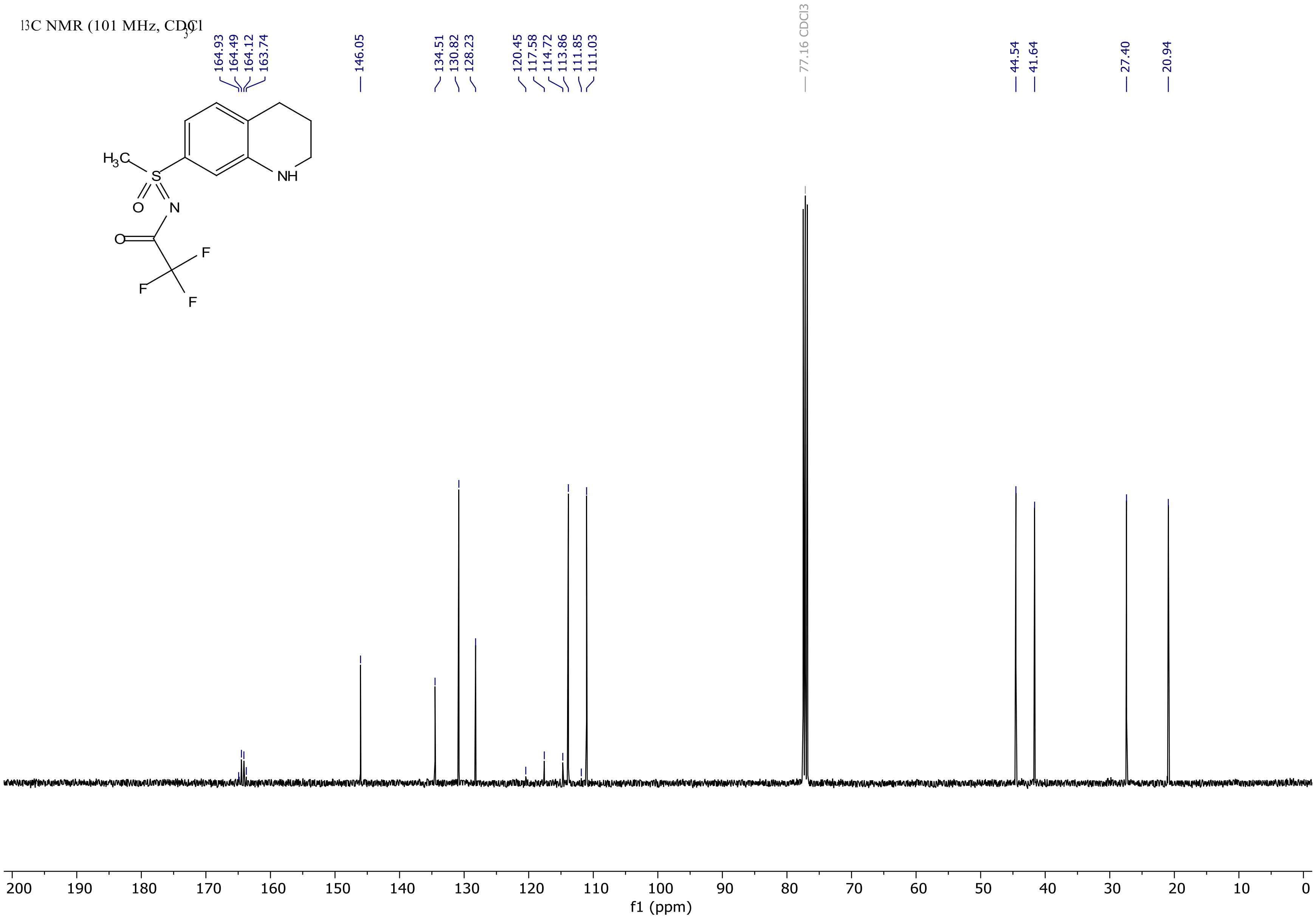

19F NMR (376 MHz, $CDCl_3$)

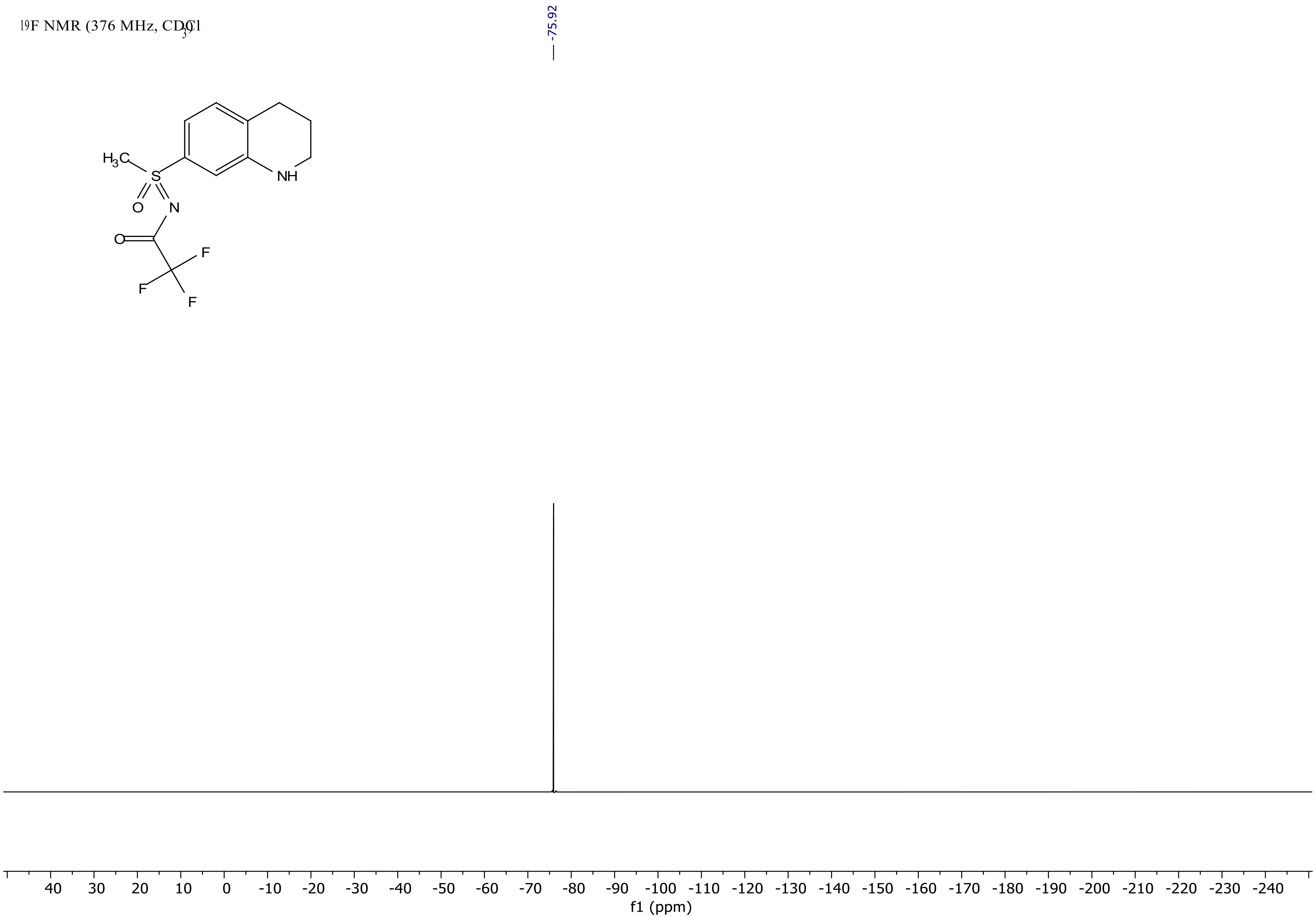

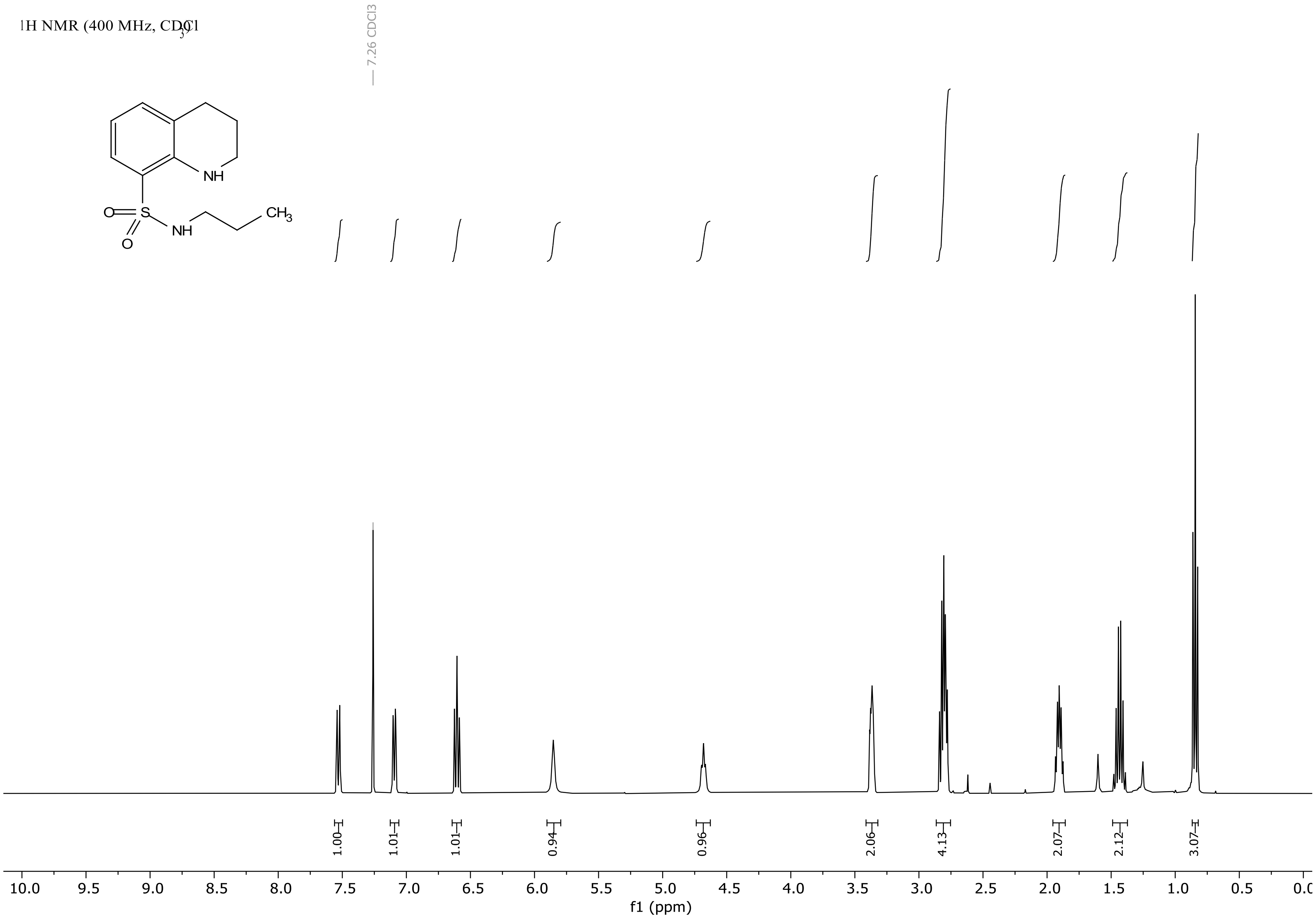
1H NMR (400 MHz, CDCl3)
7.26 CDCl3
NH
CH3
NH
O
S
O
1.00
1.01
1.01
0.94
0.96
2.06
4.13
2.07
2.12
3.07
f1 (ppm)

13C NMR (101 MHz, $CDCl_3$)

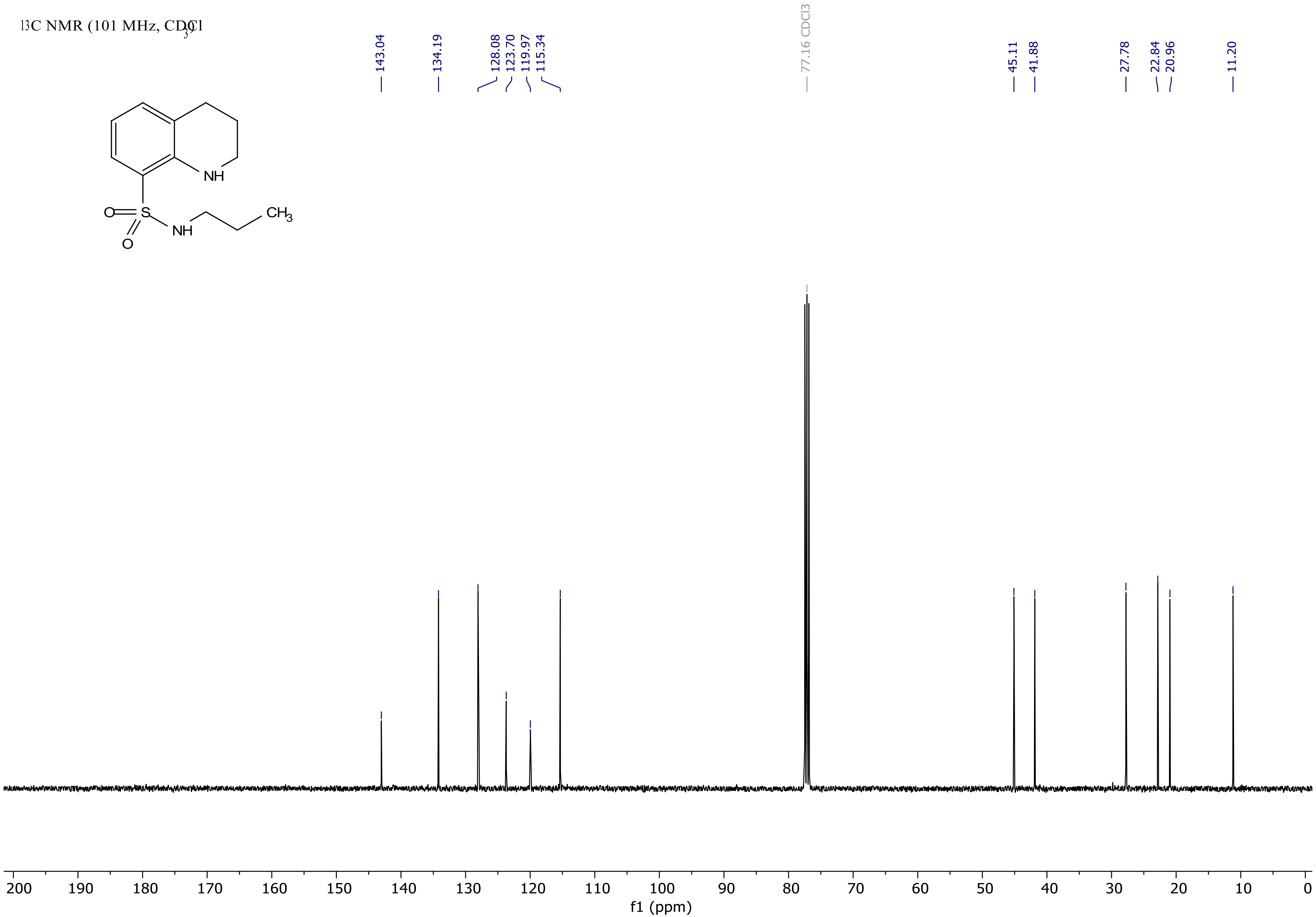

1H NMR (400 MHz, CDCl3)

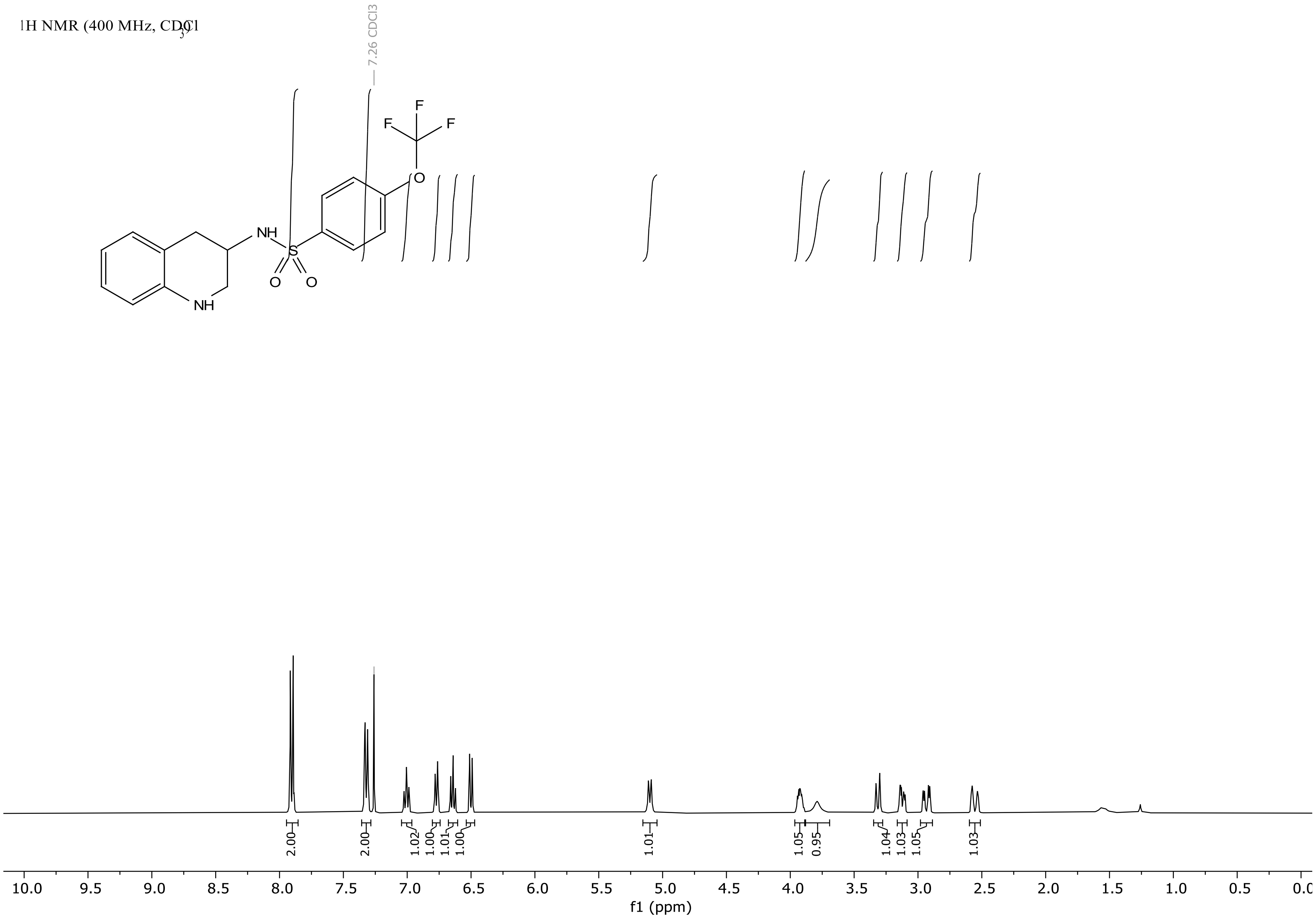

13C NMR (101 MHz, CDCl3)

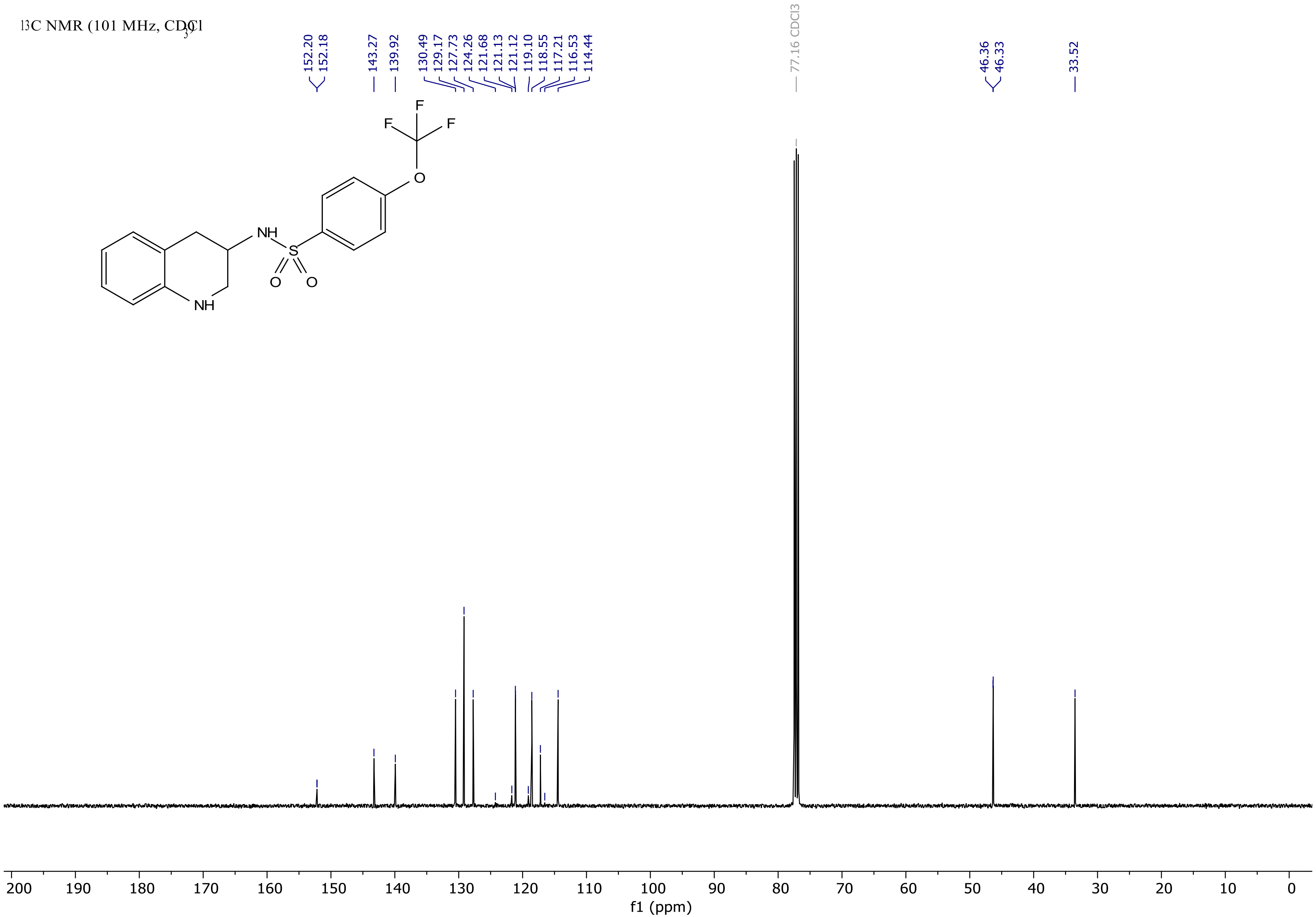

19F NMR (376 MHz, $CDCl_3$)

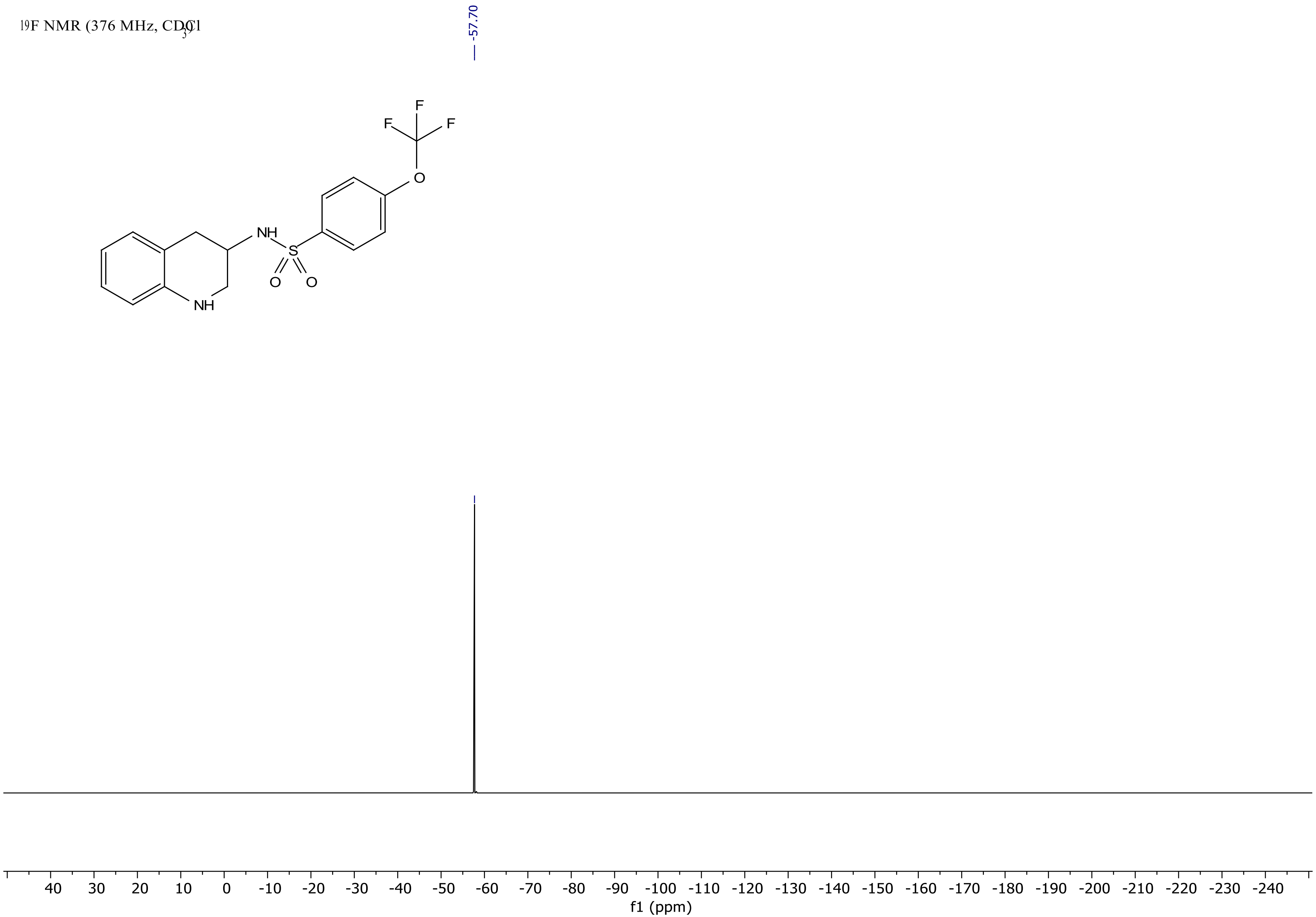

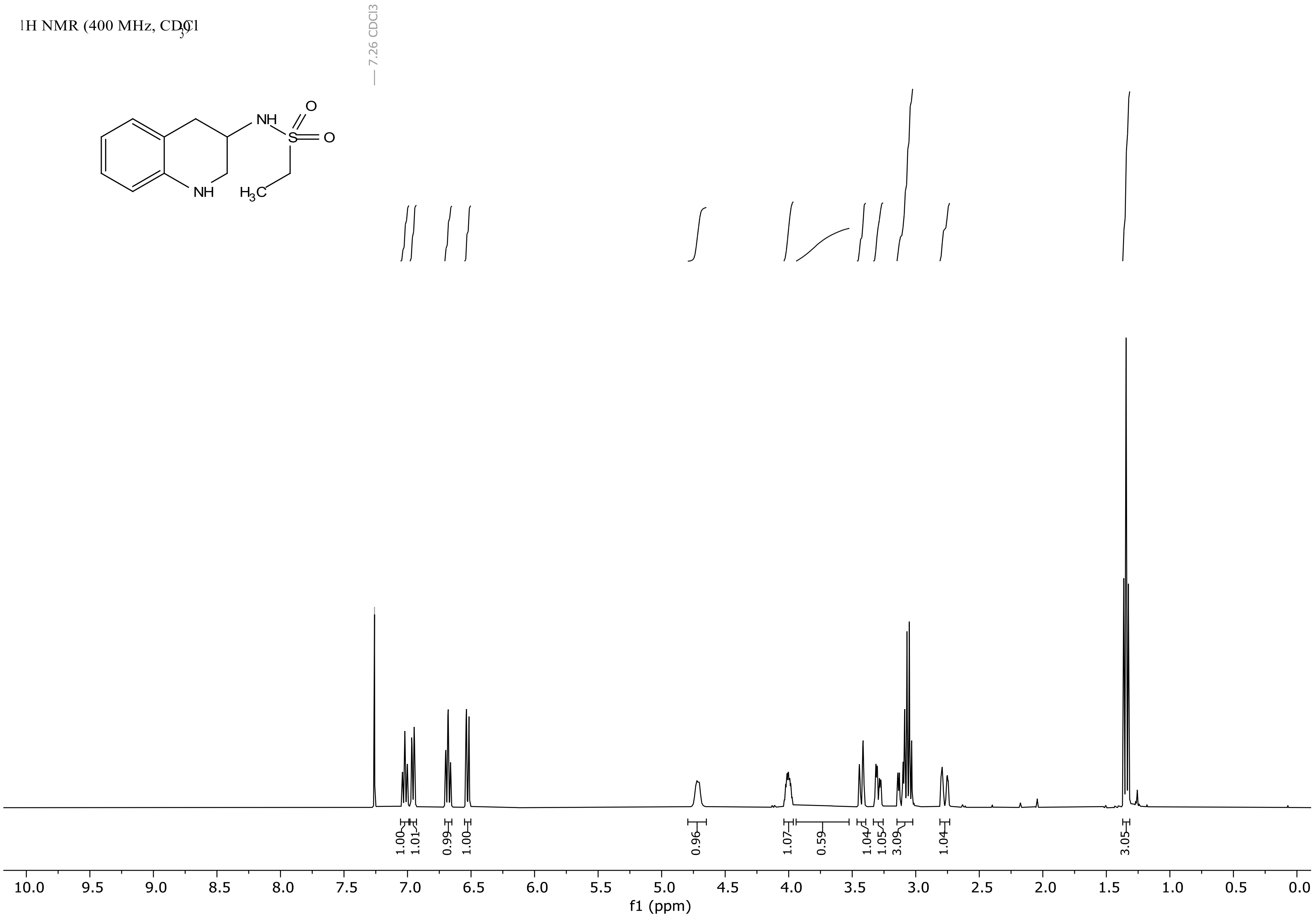
1H NMR (400 MHz, CDCl3)
7.26 CDCl3
NH
O
S
O
NH
H3C
1.00
1.01
0.99
1.00
0.96
1.07
0.59
1.04
1.05
3.09
1.04
3.05
10.0 9.5 9.0 8.5 8.0 7.5 7.0 6.5 6.0 5.5 5.0 4.5 4.0 3.5 3.0 2.5 2.0 1.5 1.0 0.5 0.0
f1 (ppm)

13C NMR (101 MHz, $CDCl_3$)

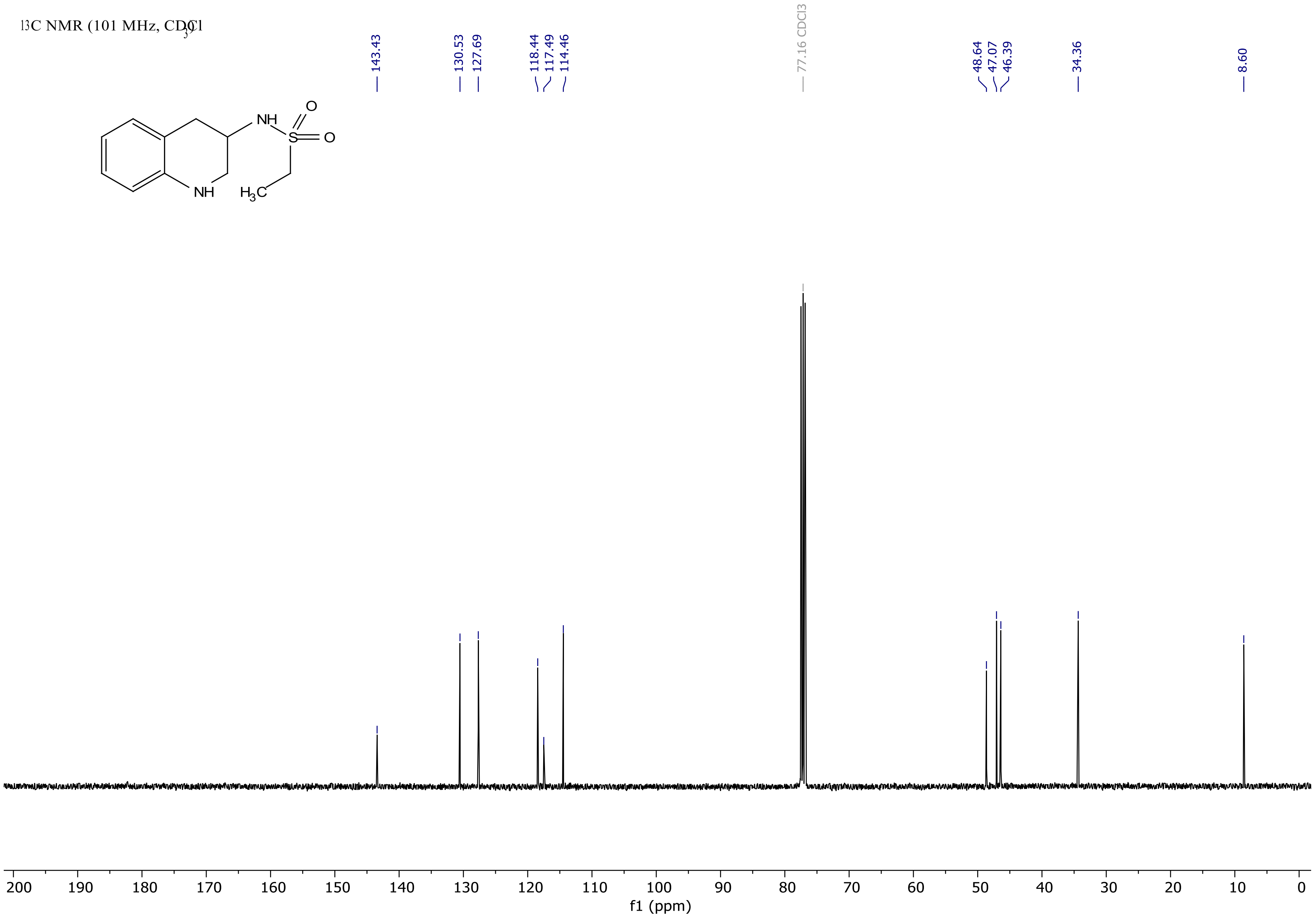

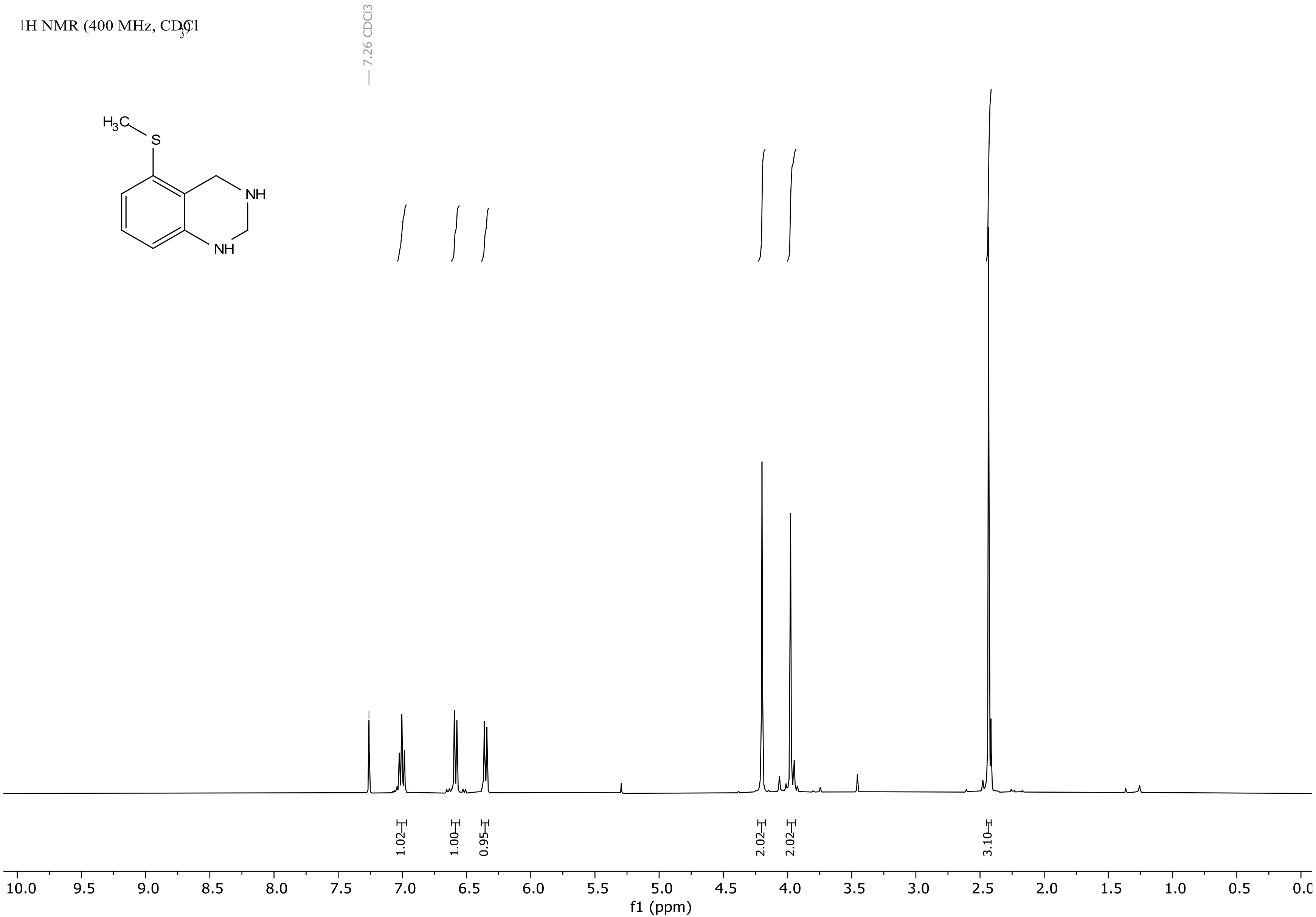
1H NMR (400 MHz, CDCl3)
7.26 CDCl3
H3C
S
NH
NH
1.02
1.00
0.95
2.02
2.02
3.10
10.0 9.5 9.0 8.5 8.0 7.5 7.0 6.5 6.0 5.5 5.0 4.5 4.0 3.5 3.0 2.5 2.0 1.5 1.0 0.5
f1 (ppm)

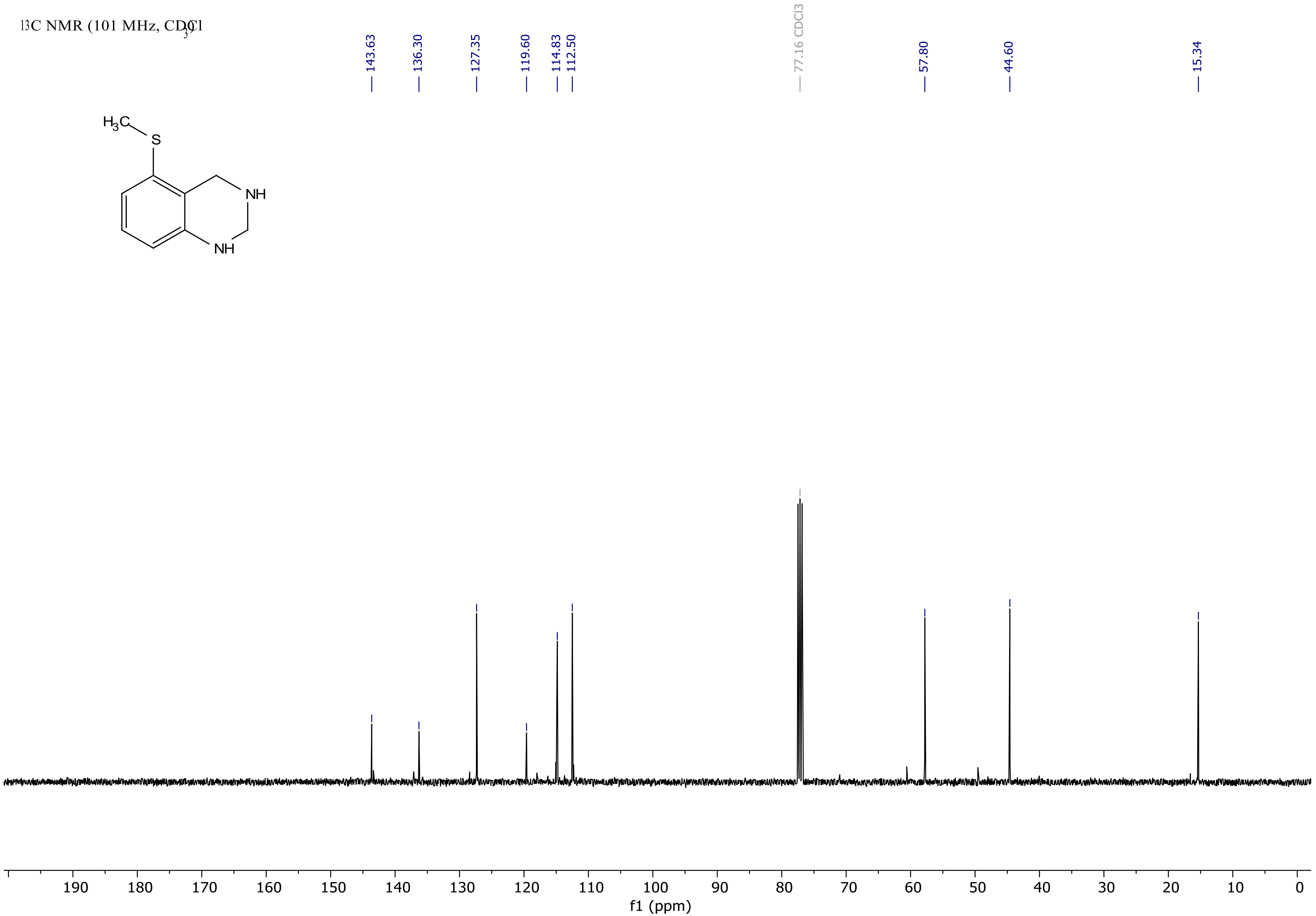
13C NMR (101 MHz, CDCl3)
143.63
136.30
127.35
119.60
114.83
112.50
77.16 CDCl3
57.80
44.60
15.34
H3C
S
NH
NH
190 180 170 160 150 140 130 120 110 100 90 80 70 60 50 40 30 20 10 0
f1 (ppm)

1H NMR (400 MHz, DMSO)

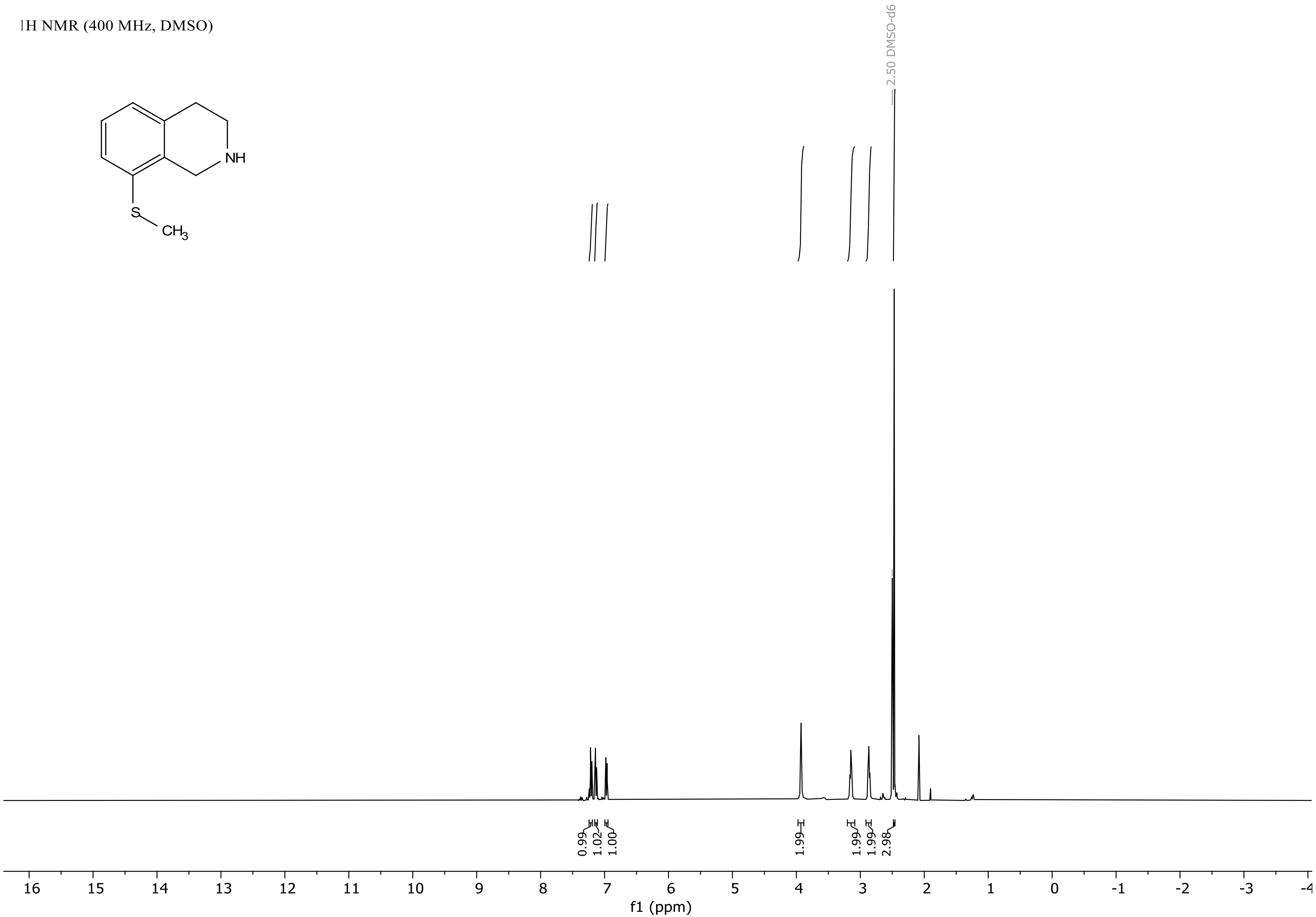

13C NMR (101 MHz, DMSO)

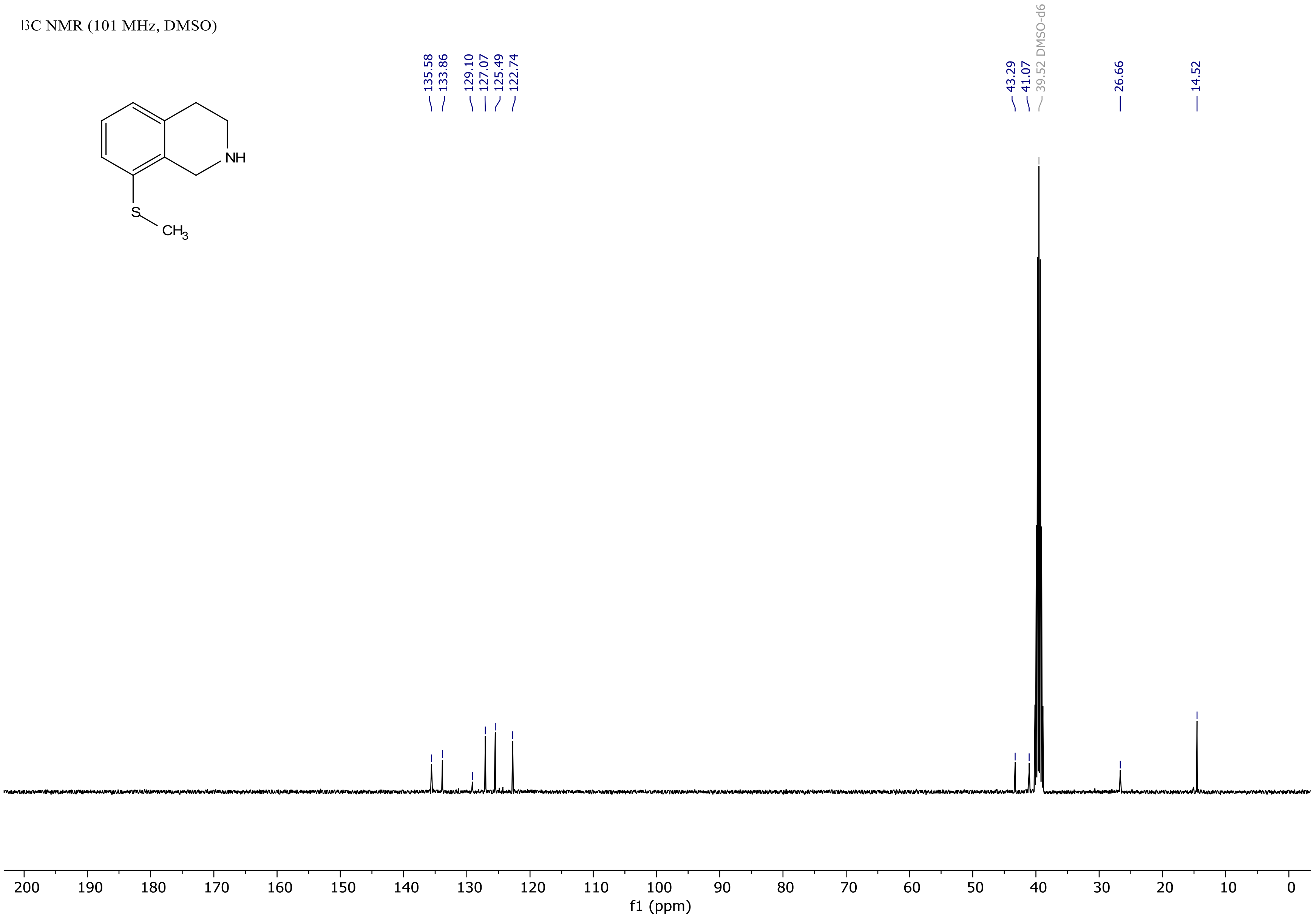

1H NMR (400 MHz, $CDCl_3$)

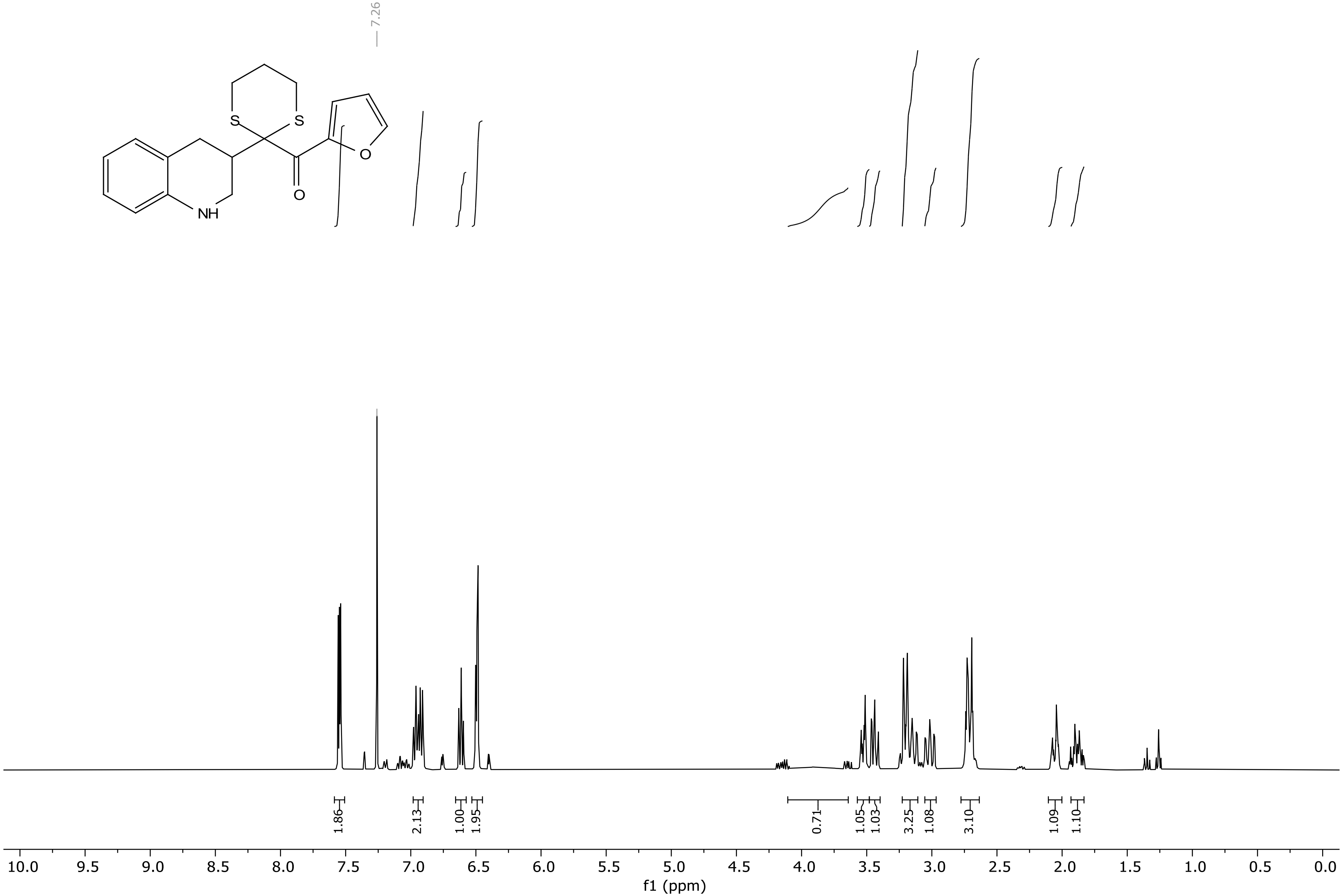

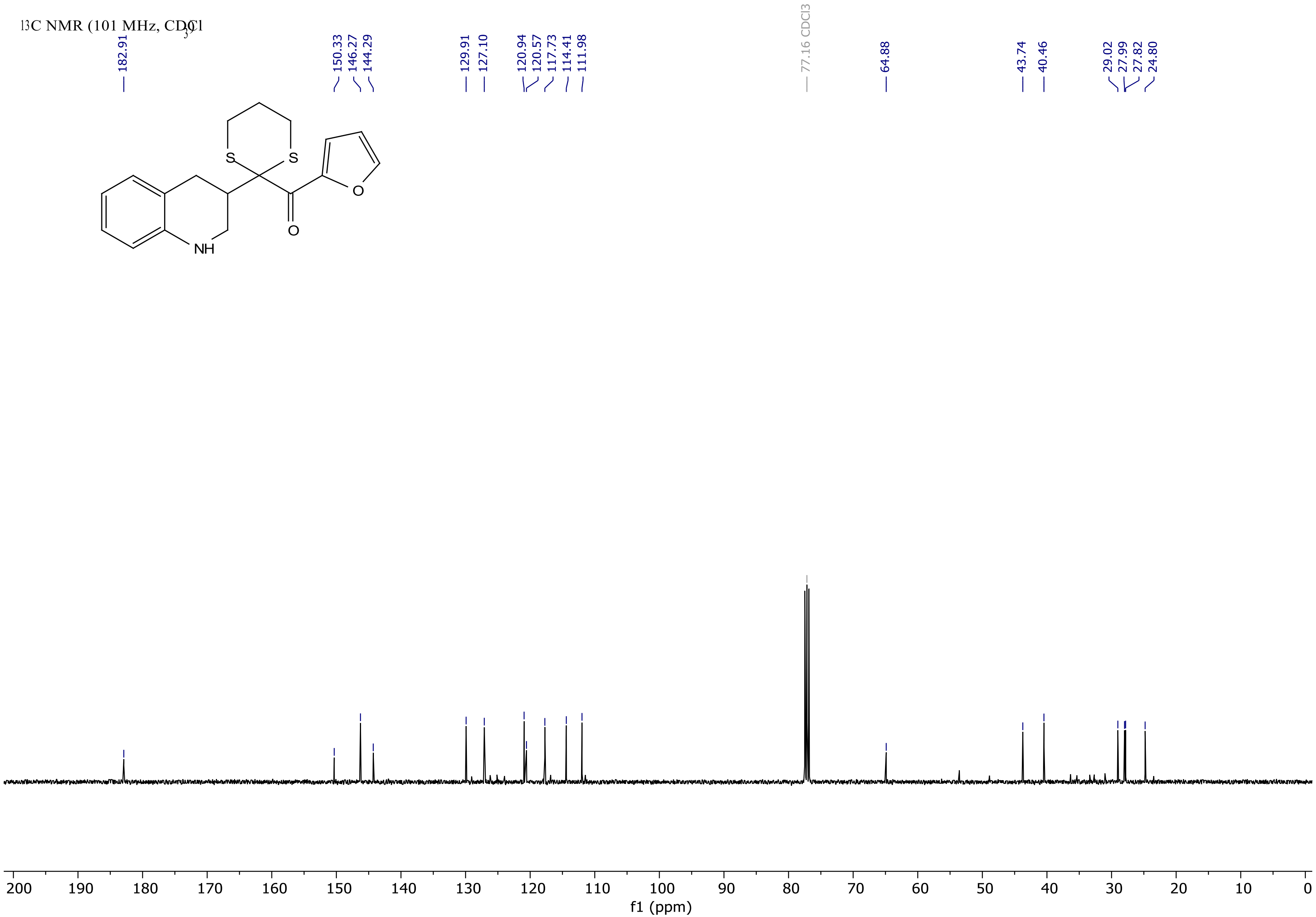
13C NMR (101 MHz, CDCl3)
182.91
150.33
146.27
144.29
129.91
127.10
120.94
120.57
117.73
114.41
111.98
77.16 CDCl3
64.88
43.74
40.46
29.02
27.99
27.82
24.80
S
S
O
O
NH
200
190
180
170
160
150
140
130
120
110
100
90
80
70
60
50
40
30
20
10
0
f1 (ppm)

1H NMR (400 MHz, CDCl3)

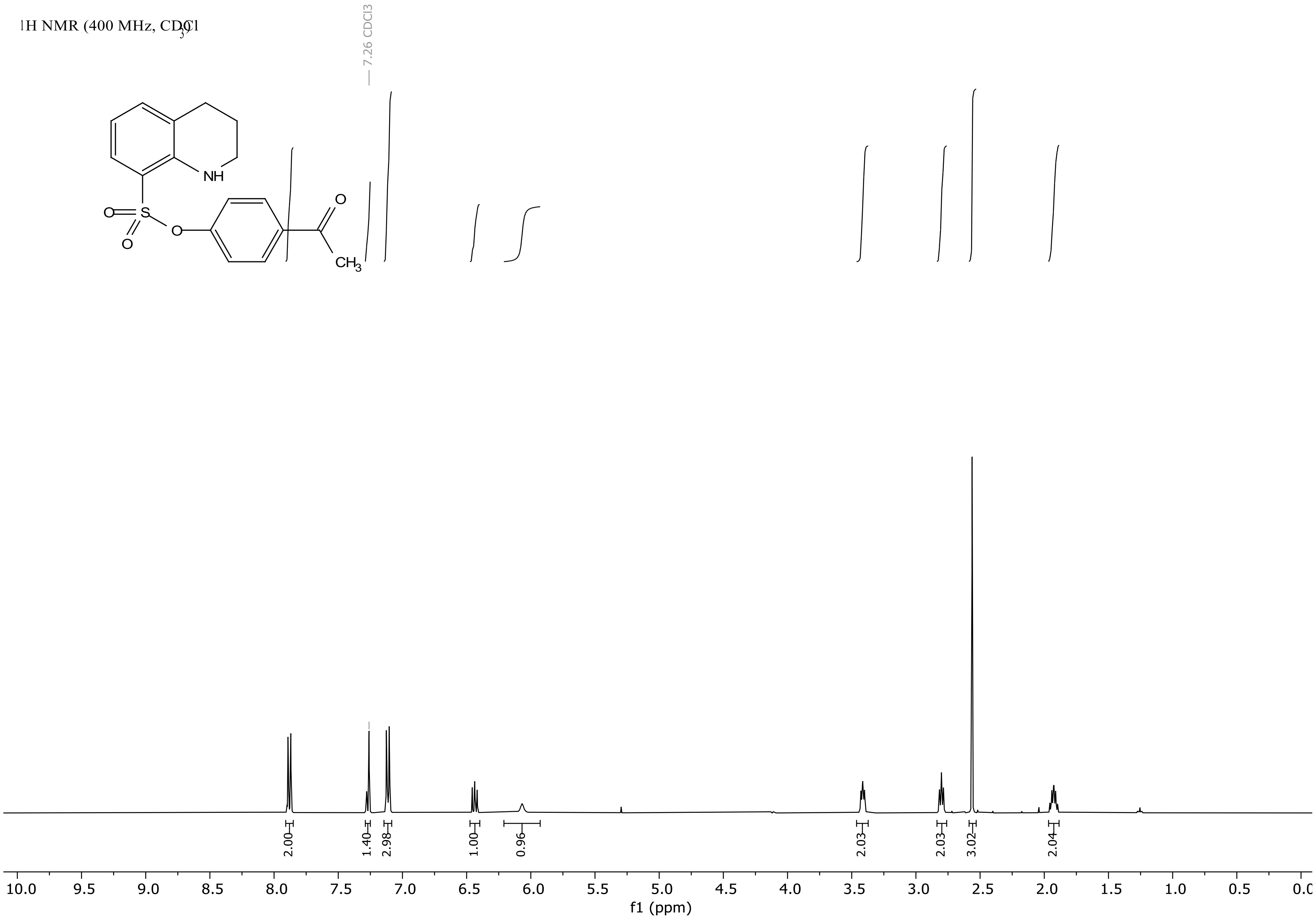

13C NMR (101 MHz, CDCl3)

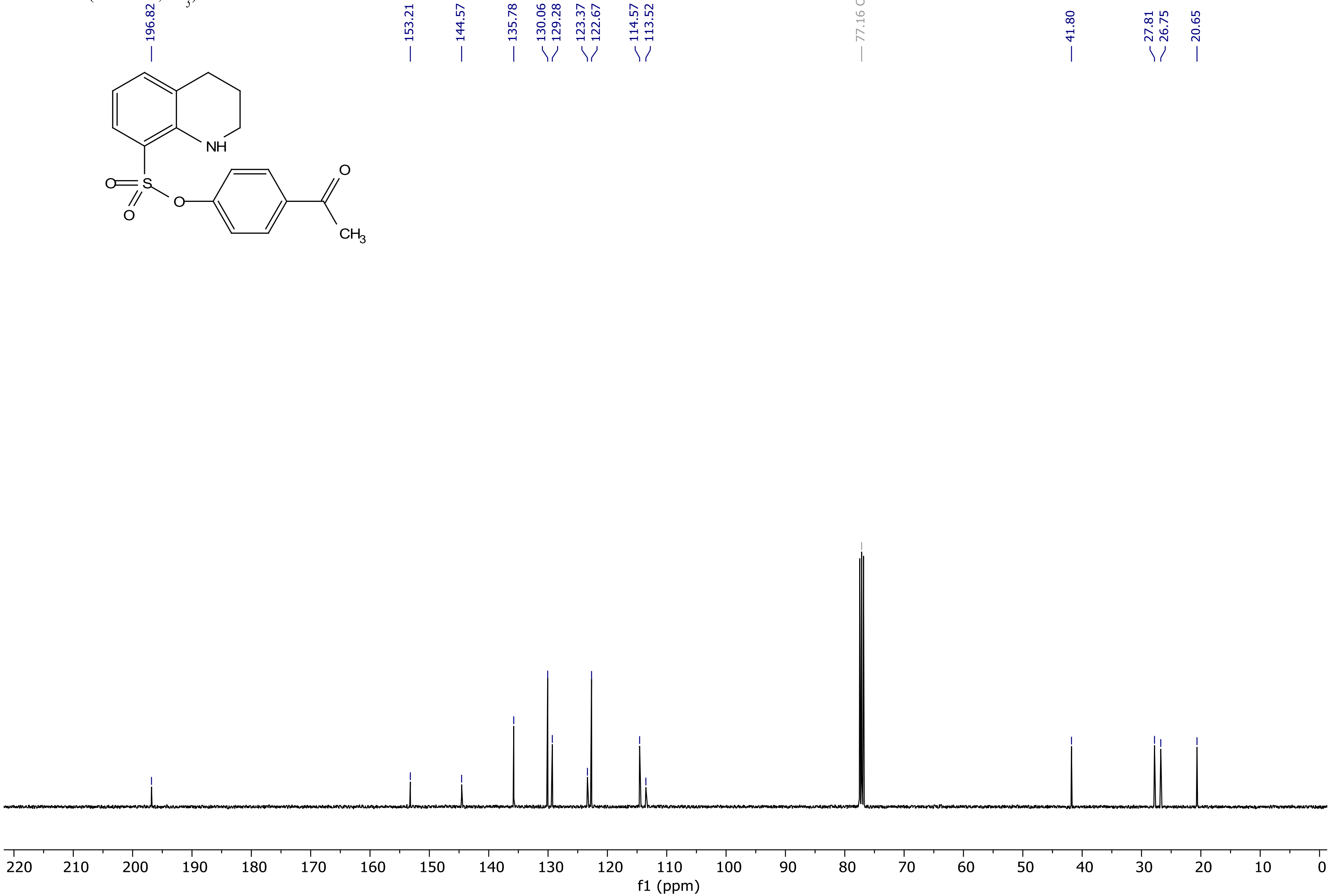

Supplement: Supplementary file 1 — ja3c11163_si_001.pdf [file ja3c11163_si_001.pdf]
